# Chirality Transfer in Gold(I)‐Catalysed Hydroalkoxylation of 1,3‐Disubstituted Allenes

**DOI:** 10.1002/chem.201603918

**Published:** 2016-11-11

**Authors:** Stacey Webster, Daniel R. Sutherland, Ai‐Lan Lee

**Affiliations:** ^1^ Institute of Chemical SciencesSchool of Engineering and Physical SciencesHeriot-Watt UniversityEdinburghEH14 4ASUK

**Keywords:** alcohols, allenes, chirality transfer, ether, gold

## Abstract

Gold(I)‐catalysed intermolecular hydroalkoxylation of enantioenriched 1,3‐disubstituted allenes was previously reported to occur with poor chirality transfer due to rapid allene racemisation. The first intermolecular hydroalkoxylation of allenes with efficient chirality transfer is reported here, exploiting conditions that suppress allene racemisation. A full substrate scope study reveals that excellent regio‐ and stereoselectivities are achieved when a σ‐withdrawing substituent is present.

## Introduction

**Scheme 1:**
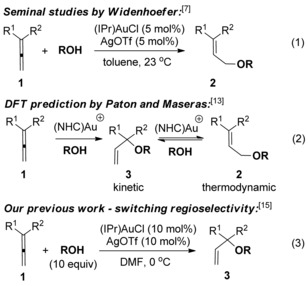
Previous work on intermolecular gold‐catalysed hydroalkoxylation of allenes.

The gold‐catalysed^1^ hydroalkoxylation of allenes^2^, ^3^ is one of the earliest homogenous gold(I)‐catalysed reactions to be reported.^4^ The ability of gold(I) to act as a soft, π‐Lewis acid makes it an ideal catalyst, and since the turn of the century, many excellent publications have emerged in the area.^5^ To date, however, much of the attention has been focused on *intra*molecular hydroalkoxylations to form cyclic ether products, including with excellent regio‐ and enantioselectivity.2e, 6 Far less attention has been paid to the *inter*molecular variant, and it was not until 2008 when Widenhoefer,^7^ Yamamoto^8^ and Horino^9^ revealed the first examples of the gold‐catalysed intermolecular hydroalkoxylation of allenes (e.g., reaction (1), Scheme 1).^10^, ^11^, ^12^ Following these seminal reports, Paton and Maseras proposed, based on DFT calculations, that the regioselectivity observed by Widenhoefer and Yamamoto is due to isomerisation of the kinetic tertiary allylic ether **3** to the thermodynamic primary allylic ether **2**, rather than preferential activation of **1** at the less‐hindered double bond, as originally assumed (reaction (2), Scheme 1).^13^ Drawing on previous success in controlling the regioselectivity of gold‐catalysed alcohol additions to cyclopropenes,^14^ we subsequently developed conditions to suppress isomerisation of **3** to **2**, thereby switching the regioselectivity to the kinetic, tertiary allylic ether product **3** (reaction (3), Scheme 1).^15^ The use of DMF as solvent was the crucial difference, possibly because it reduces the activity of the active cationic gold catalyst (IPr)Au^+^ (IPr=1,3‐bis(2,6‐diisopropylphenyl)imidazol‐2‐ylidene) by means of reversible coordination.^16^

**Scheme 2:**
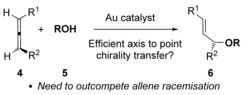
1,3‐Disubstituted allenes can in principle undergo chirality transfer.

**Scheme 3:**
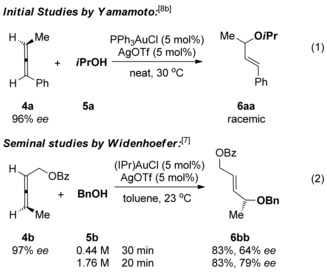
Previous reported attempts at chirality transfer in gold‐catalysed hydroalkoxylation of allenes.

In contrast to 1‐substituted and 1,1‐disubstituted allenes **1**, 1,3‐disubstituted allenes **4** have been far less studied, presumably due to regioselectivity issues when R^1^ and R^2^ are not electronically differentiated. 1,3‐Disubstituted allenes **4**, however, are extremely interesting due to the possibility of axis to point chirality transfer during the hydroalkoxylation reaction (Scheme 2).^17^ Surprisingly, Yamamoto et al. observed that, while the related gold‐catalysed hydramination reaction with allene **4 a** occurred with good chirality transfer,^18^, ^19^ the corresponding hydroalkoxylation, using less nucleophilic alcohols instead of anilines as nucleophiles, produced only racemic product (reaction (1), Scheme 3).8b In his seminal work, Widenhoefer et al. also reported chirality transfer reaction on one substrate, allene **4 b**, and obtained a much more promising result.^7^ Nevertheless, the reaction still proceeded with substantial erosion of enantiomeric excess (97→79 % *ee*, reaction (2), Scheme 3). In both cases, it is thought that rapid racemisation of the allene^20^ under the hydroalkoxylation conditions is to blame.^7^, ^8^ Thus far, this remains an unsolved problem and represents a clear limitation in the area.

**Scheme 4:**
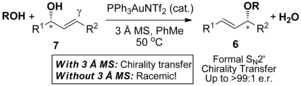
Addition of molecular sieves (MS) affects efficiency of chirality transfer.

Meanwhile, we recently carried out a full investigation into chirality transfer in gold‐catalysed allylic etherification reactions; this reaction is related to the title reaction by virtue of forming similar allylic ether products **6**, though with different substrate and mechanism. Surprisingly, we discovered that the addition of molecular sieves (MS) was crucial for both regioselectivity and efficient chirality transfer (Scheme 4).^[21**,** 22]^ In the absence of molecular sieves, only racemic allylic ether products **6** were observed. Inspired by this discovery, we decided to revisit the gold‐catalysed hydroalkoxylation of allenes (Scheme 2 and Scheme 3) in order to investigate whether a similar approach would allow us to finally realise efficient chirality transfers. In this full article, we describe our endeavours and disclose for the first time a method for intermolecular hydroalkoxylation of allenes with high degree of chirality transfer, along with full substrate scope studies.

## Results and Discussion

**Scheme 5:**
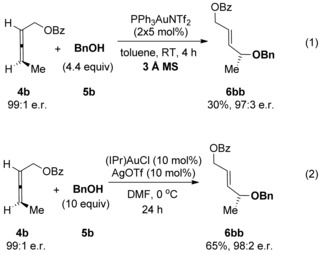
Initial attempts at increasing the levels of chirality transfer.

We initiated our investigations using the allene **4 b**, since regioselectivity is not an issue with this substrate^7^ (see below), and also in order to readily compare our results with previous studies (Scheme 3). Working on the assumption that any erosion of chirality transfer originates from rapid racemisation of the allene substrate, as suggested by Widenhoefer and Yamamoto, while not ruling out racemisation of the allylic ether product through isomerisation (see reaction (2), Scheme 1), it is clear in either case that the conditions need to be modified in order to retard the racemisation step while still actively catalysing the desired hydroalkoxylation reaction. Based on our previous related studies mentioned above, we hypothesised that our two previous sets of conditions could potentially be solutions: 1) conditions in reaction (3), Scheme 1 (DMF, 0 °C, excess alcohol), which was found to suppress any further isomerisation of the allylic ether products, and 2) conditions using molecular sieves, as shown in Scheme 4, which was also shown to favour the kinetic products.^23^ Pleasingly, our initial results were promising and are summarised in Scheme 5. Addition of molecular sieves did indeed result in higher chirality transfer (97:3 e.r.), however, at the cost of a drop in yield (30 %, reaction (1), Scheme 5). Unfortunately, further attempts at optimisation by increasing the time, temperature and catalyst loading did not significantly improve the yield without a subsequent drop in e.r. Therefore, we turned to our previous conditions shown in reaction (3), Scheme 1 instead: (IPr)AuCl/AgOTf (Tf=triflate) pre‐catalyst at 0 °C in DMF, with excess alcohol. To our delight, high chirality transfer (98:2 e.r.) is observed and in a much better 65 % yield of **6 bb** (reaction (2), Scheme 5).

**Scheme 6:**
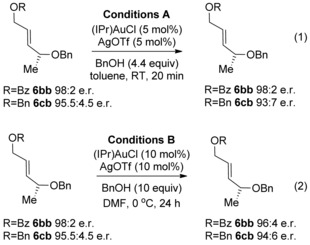
Control reaction to test whether the products **6** racemise under the reaction conditions.

Before proceeding to the substrate scope, we carried out control reactions to ascertain whether erosion in enantiomeric excess occurs through racemisation of the allylic ether product **6** or racemisation of the allene substrate **4**, or both. Towards this end, the allylic ether products **6 bb** and **6 cb** were subjected to two reaction conditions: the original conditions used by Widenhoefer in reaction (2) Scheme 3 (hereafter referred to as Conditions A) and also our conditions shown in reaction (2), Scheme 5 (hereafter referred to as Conditions B). As shown in Scheme 6, there was either no or very little erosion of enantiomeric excess, thereby suggesting that isomerisation of products **6** is not the major cause of *ee* erosion in the gold‐catalysed hydroalkoxylation reaction.

Next, the allene substrate **4 b** was subjected to similar controls (Table 1). This time, however, sterically hindered *t*BuOH (which is a sluggish nucleophile under these conditions, see below) was added to these control reactions in order to replicate the reaction conditions whilst avoiding hydroalkoxylation.^24^ As shown in Table 1, Conditions A clearly result in much faster racemisation of the allene **4 b** compared to Conditions B.

**Table 1:** Controls to test whether the allene substrate **4** racemises under the reaction conditions.

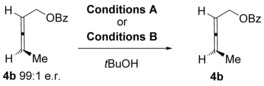			
Entry	Time	Conditions A^[a]^	Conditions B^[a]^
1	20 min	98:2 e.r.	98.5:1.5 e.r.
2	2 h	89:11 e.r.	96.5:3.5 e.r.
3	24 h	racemic	no allene remaining

[a] Determined by CSP‐HPLC.

**Scheme 7:**
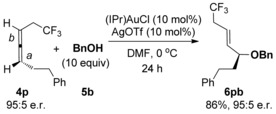
Effect of solvent and temperature on allene racemisation.

Having confirmed that the racemisation of the allene substrate is indeed the major culprit for *ee* erosion under Conditions A, we then proceeded to ascertain whether it is the solvent (DMF versus toluene), temperature (0 °C versus RT) or alcohol concentration that is causing the stark difference in results using Conditions A versus B as shown in Table 1. Towards this end, the racemisation control under Conditions B was investigated with toluene instead of DMF as the solvent (Scheme 7). The resulting enantiomeric ratios are very similar (within error): 96.5:3.5 e.r. in DMF and 97.5:2.5 e.r. in toluene, which initially suggests that the solvent difference between Conditions A and B is not the most crucial change with this substrate. The temperature, however, is crucial (Scheme 7); when the same toluene experiment is repeated at room temperature, the e.r. of **4 b** plummets to 81:19.

With these observations in hand, we then proceeded to study the effect of the solvent in the actual gold‐catalysed hydroalkoxylation reaction with allene **4 b** (Table 2). Indeed, the change of solvent from DMF and toluene has only a small effect on resulting enantiomeric ratios for substrate **4 b** (98:2 vs. 97:3), but the yield is slightly better with toluene as solvent (65 vs. 81 %, entries 1 and 2). Sticking with toluene, the alcohol equivalents was investigated next (entries 3–6). The enantiomeric ratio appears to drop slightly as the alcohol equivalents is decreased from 4 to 2 to 1.1 equivalents (entries 3–6). As a result of this screen, we initially decided to use the conditions shown in entry 4 (hereafter referred to as Conditions C) as the optimised reaction conditions, as it provided a good compromise between lower equivalents of alcohol nucleophile and good e.r./yield.

**Table 2:** Effect of solvent and alcohol concentration on the hydroalkoxylation of **4 b**

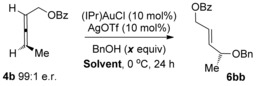				
Entry	Solvent	BnOH equiv	e.r.^[a]^	Yield [%]
1	DMF	10	98:2	65
2	toluene	10	97:3	81
3	toluene	4	97:3	85
4^[b]^	toluene	2	96:4	78
5	toluene	1.1	93:7	74

[a] Determined by CSP‐HPLC, >20:1 *E:Z* by ^1^H NMR analysis. [b] Conditions C.

However, we soon discovered to our surprise that Conditions C only gave good enantiomeric ratios specifically for substrate **4 b**, and consistently provided poorer enantiomeric ratios for all other allenes investigated (see below, Table 4). In fact, when applied to other allenes **4** during our substrate scope studies, our original Conditions B consistently outperform Conditions A and C in chirality transfer efficiency. For example, when **4 c** is used instead of **4 b**, Conditions B (95:5 e.r., entry 2, Table 3) outperform both A (81:19 e.r., entry 1) and C (87:13 e.r., entry 3). In this case, the switch of solvent alone from DMF (95:5 e.r., entry 2, Table 3) to toluene (92.5:7.5 e.r., entry 4) produces a worse result in enantiomeric ratio.

**Table 3:** Effect of solvent and alcohol concentration on the hydroalkoxylation of **4 c**.

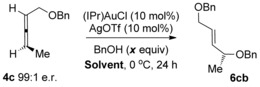						
Entry	Solvent	BnOH equiv	*T* [°C]	Conditions	e.r.^[a]^	Yield [%]
1^[b]^	toluene	4.4	RT	cond. A	81:19	37
2	DMF	10	0	cond. B	95:5	45
3	toluene	2	0	cond. C	87:13	61
4	toluene	10	0	–	92.5:7.5	65

[a] Determined by CSP‐HPLC. >20:1 *E:Z* by ^1^H NMR analysis. [b] 20 min.

We therefore conclude that, with the exception of the initially studied allene **4 b**, in general, the solvent, temperature and alcohol equivalents all cumulatively affect the efficiency of the chirality transfer reaction when comparing Conditions B to the previously reported Conditions A. Although the yield is lower using DMF for this particular combination of reactants (**4 c** with BnOH), the yields were pleasingly all good to excellent when Conditions B were applied to other substrates (see later, Table 4 and Table 5).

Having ascertained that Conditions B is the most general, we proceeded to investigate the allene substrate scope (Table 4).^25^ Suspecting that the OBz substituent plays a major role in the excellent regioselectivity observed, we first investigated the effect of various different protecting groups on the oxygen (entries 1–4). To our delight, removing the carbonyl (**4 c**, entry 2) or Ph (**4 d**, **4 e**, entries 3 and 4) does not seem to significantly affect enantiomeric ratios and a high degree of chirality transfer occurs in all cases. Replacing the Me in **4 b** with a longer *n*pentyl in **4 f** also results in high yield and chirality transfer (entry 5). Inserting an extra CH_2_ to place the OBz group further from the allene in **4 g**, however, does cause a more noticeable drop in chirality transfer, although **6 gb** is still formed in a good 79 % yield and 90:10 e.r. (entry 6).

**Table 4:** Allene scope.

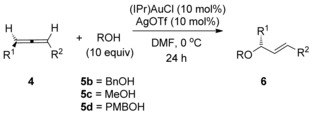			
Entry	Allene	Product	Result^[a]^
1	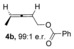	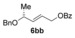	65 %
			98:2 e.r.^[b]^
2	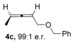	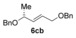	45 %
			95:5 e.r.^[b]^
3	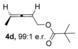	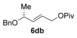	70 %
			93:7 e.r.^[c]^
4	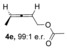	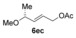	78 %
			94:6 e.r.^[c]^
5	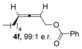	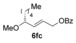	91 %
			97:3 e.r.^[b]^
6^[e]^	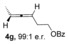	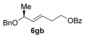	79 %
			90:10 e.r.^[b]^
7	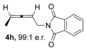	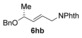	81 %
			97:3 e.r.^[b]^
8^[g]^	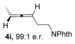	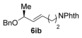	94 %
			81:19 e.r.
			9:1 regioselectivity^[f]^
9^[h]^	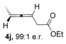	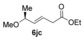	92 %
			97:3 e.r.^[b]^
10	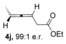	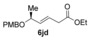	71 %
			95:5 e.r.^[b]^
11	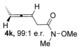	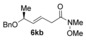	58 %
			91:9 e.r.^[b]^
12	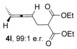	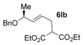	86 %
			90:10 e.r.^[b]^
13	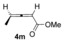	–	–^[i]^
14	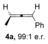	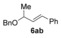	79 %^[j]^
			racemic
			10:1 regioselectivity
15	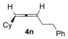	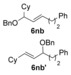	71 %,^[j]^
			1:0.7 regioselectivity
16	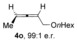	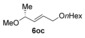	94 %
			87:13 e.r.^[d]^

[a] Isolated yields, >20:1 *E:Z* and regioselectivity by ^1^H NMR analysis unless otherwise stated**. 5 c** was used when the product using **5 b** is not separable by CSP‐HPLC, CSP‐GC or chiral shift reagents. [b] Determined by CSP‐HPLC. [c] Determined by ^1^H NMR using chiral shift reagent (*R*)‐(−)‐1‐(9‐anthryl)‐2,2,2‐trifluoroethanol. [d] Determined by CSP‐GC. [e] Conditions C gives 70 % yield and 78:22 e.r. [f] When (IPr)AuNTf_2_ used as catalyst instead, regioselectivity improves to>20:1; 82:18 e.r. [g] Conditions C gives 79 % yield, 7:1 regioselectivity, 78:22 e.r. [h] Conditions C gives 63 % yield, 97:3 e.r. [i] Mainly **4 m** and complex mixture of products. [j] Combined yield.

Next, we proceeded to replace the O in **4 b** with N (**4 h**) and pleasingly, this still gives a high 81 % yield and 97:3 e.r. of **6 hb** (Table 4, entry 7). Once again, placing the NPhtalate functionality further from the allene in **4 i** results in a more noticeable drop in enantiomeric ratio (81:19 e.r., entry 8). While all previous examples (entries 1–7) provided exclusively one regioisomer, the regioselectivity with **4 i** is lower, albeit a still very good 9:1 (entry 8), suggesting that the functionality on the substituent is indeed responsible for the excellent regioselectivities observed thus far. Nevertheless, when the silver free catalyst (IPr)AuNTf_2_ is used, the regioselectivity is restored to >20:1 (82:18 e.r.).

Next, the ester (**4 j**) and Weinreb amide (**4 k**) substituted allenes were investigated (Table 4, entries 9–11). These once again provide the expected products, with **6 jc** and **6 jd** being formed in excellent yield and e.r. (92 %, 97:3 e.r. and 71 %, 95:5 e.r. respectively) and **6 kb** in a slightly lower but still good 91:9 e.r. Once again, moving the ester functionality one carbon away (**4 l**) still provides excellent regioselectivity and 90:10 e.r. (entry 12). Having the ester directly conjugated to the allene (**4 m**), however, is surprisingly detrimental to the reaction (entry 13).

Having successfully demonstrated good regioselectivities and enantiomeric ratios with a wide range of functionalised allenes, we next turned our attention to unfunctionalised ones. Aryl substituted allene **4 a**, originally investigated by Yamamoto (reaction (1), Scheme 3), gave a good 10:1 regioselectivity but still racemised under these conditions (Table 4, entry 14). It is likely that the aryl substituent renders the allene isomerisation too rapid for successful chirality transfer under gold‐catalysed hydroalkoxylation conditions.20b Next, we investigated whether steric differentiation in a dialkyl 1,3‐substituted allene (**4 n**) could result in good regioselectivity. Disappointingly, this is not the case and **6 nb** and **6 nb′** is formed as an inseparable regioisomeric mixture (entry 15).^26^ The poor regioselectivity with double alkyl substituents (**4 n**) is not necessarily a major drawback for synthetic purposes, as functionalised substituents are much more useful as a handle for subsequent elaboration in synthesis.

In order to investigate the minimum amount of functionality required to achieve good regioselectivity, the ether allene **4 o** was investigated next (entry 16). Pleasingly, the reaction is regioselective, and a decent 87:13 e.r. is observed for product **6 oc**. It is clear that some functionality on one substituent is required for good regio‐ and stereoselectivities, and the heteroatom (**4 o**) and carbonyls (**4 b**, **4 d**–**l**) all seem to play a role in the observed selectivity.

The alcohol nucleophile scope was investigated next using allene substrate **4 b** (Table 5). Primary benzyl (**5 b**) and phenethyl alcohol (**5 f**), as well as alkyl alcohols MeOH (**5 c**) and *n*‐butanol (**5 e**) all react smoothly to furnish the desired allylic ethers in good yields and enantioselective ratios (entries 1–4). Primary alkyl alcohols **5 g** and **5 h**, with pendent electron withdrawing Cl and CF_3_ groups respectively, also react smoothly and in high enantioselective ratios, albeit with slightly lower yields (60 and 37 %, entries 5 and 6). To our delight, the more sterically hindered secondary alcohol *i*PrOH **5 a** reacts in good yield (88 %) and e.r. (97:3 e.r., entry 7). Homochiral secondary alcohols **5 j** and **5 k** also proceed with excellent e.r. and d.r., although the yield is higher for the less bulky **5 j** (78 %) versus **5 k** (51 %) (entries 8–9). Unsurprisingly, therefore, the bulky tertiary alcohol *t*BuOH **5 l** reacts sluggishly; nevertheless, 30 % of the desired product is obtained in a good 94:6 e.r. (entry 10). This difference in reactivity, however, allows for chemoselective reaction of unprotected diol **5 m** at the less‐hindered primary end (entry 11). Other potentially sensitive functional groups such as a pendent alkene (**5 n**) and a furan (**5 o**) are also pleasingly tolerated (entries 12 and 13). The less nucleophilic phenol (**5 p**), however, is not a viable nucleophile in this reaction (entry 14).

**Table 5:** Alcohol scope.

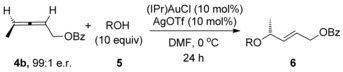			
Entry	Alcohol	Product	Result^[a]^
1	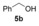	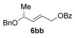	65 %
			98:2 e.r.^[b]^
2	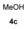	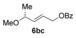	81 %
			>95:5 e.r.^[c]^
3	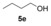	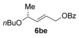	68 %
			97.5:2.5 e.r.^[b]^
4	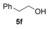	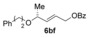	62 %
			98:2 e.r.^[c]^
5	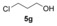	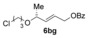	60 %
			97:3 e.r.^[b]^
6	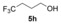	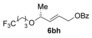	37 %
			99:1 e.r.^[b]^
7	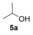	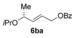	88 %
			97:3 e.r.^[b]^
8	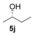	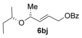	78 %
			81:19 e.r.^[b]^
9	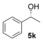	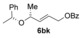	51 %
			97:3 e.r.^[b]^
10	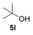	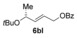	30 %
			94:6 e.r.^[b]^
11	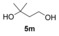	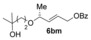	60 %
			98:2 e.r.^[b]^
12	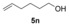	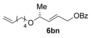	66 %
			99.8:0.2 e.r.^[b]^
13	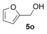	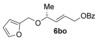	64 %
			99:1 e.r.^[b]^
14	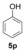	N/A	No reaction

[a] Isolated yields, >20:1 *E:Z* and regioselectivity by ^1^H NMR analysis unless otherwise stated. [b] Determined by CSP‐HPLC. [c] Determined by ^1^H NMR using chiral shift reagent (*R*)‐(−)‐1‐(9‐anthryl)‐2,2,2‐trifluoroethanol. [d] Determined on the THP deprotected product.

**Scheme 8:**
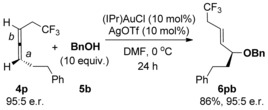
Reaction to ascertain that regioselectivity is due to inductive effects.

Finally, in order to ascertain whether the heteroatom and carbonyl groups in **4 b**–**l** and **4 o** impart excellent regioselectivity through a chelation effect or a simple inductive withdrawing effect, the reaction with allene **4 p** was investigated (Scheme 8). Allene **4 p** contains a non‐chelating electron‐withdrawing substituent (CF_3_) in place of the O, N or C=O withdrawing groups in **4 b**–**4 l**, **4 o**, but still produces a highly regioselective reaction (Scheme 8). This suggests that the preference for reaction at *a* is purely due to electronics: The inductive withdrawing effect of the functional group (CF_3_ in this case) results in electronically differentiated π‐bonds *a* and *b*, with the LAu^+^ catalyst preferring to coordinate to the more electron‐rich π‐bond at position *a*. It should be noted though that allene **4 m**, with electronically differentiated π‐bonds through a conjugated rather than inductive withdrawing group, performs very poorly in the reaction (entry 13, Table 4). Furthermore, because the unwanted and competing gold‐catalysed allene racemisation is thought to occur through achiral η^1^‐allylic cation intermediates,^20^ it is possible that allenes with substituents that stabilise these intermediates (such as Ph in **4 a**) undergo fast racemisation, and therefore provide poor chirality transfer in the reaction, whereas inductive withdrawing substituents have the opposite effect and allow for excellent transfer of chirality.

**Scheme 9:**
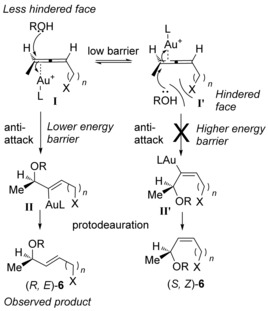
Postulated origin of stereoselectivity.

It should be noted that the reaction is highly stereoselective both in terms of chirality transfer as well as *E* selectivity (>20:1 *E:Z* by ^1^H NMR analysis). A plausible reason for the observed selectivity is shown in Scheme 9. The gold catalyst can coordinate to either face of the allene (**I** and **I′**), with a low barrier of interconversion between **I** and **I′**.^27^ Because nucleophiles typically approach *anti* to the Au^I^,^1^ the approach of the alcohol onto **I** should have the lower energy barrier as it approaches from the less hindered face of the allene. This leads to intermediate **II** and the observed (*R*,*E*)‐**6** product upon protodeauration. Conversely, nucleophilic attack onto **I′** is predicted to be kinetically unfavourable, and indeed (*S*,*Z*)‐**6** is never observed experimentally.^28^ Furthermore, subjecting a mixture of *E:Z* isomers of an allylic ether **6** to the gold‐catalysed hydroalkoxylation conditions (see Supporting Information) results in no change to the *E:Z* ratio, suggesting that the *E* selectivity is *not* due to thermodynamic control.

## Conclusion

Gold(I)‐catalysed intermolecular hydroalkoxylation of enantioenriched 1,3‐disubstituted allenes was previously reported to occur with poor chirality transfer due to rapid racemisation of the allene substrate. We have developed conditions to overcome this limitation and to successfully carry out gold(I)‐catalysed intermolecular hydroalkoxylation of allenes with high degree of chirality transfer (up to 99:1 e.r.), excellent *E* selectivity and good substrate scope. The combined use of the coordinating solvent DMF, lower temperatures and higher equivalents of alcohol nucleophile suppresses allene racemisation and thereby allows for the asymmetric hydroalkoxylation using a wide range of alcohol nuceophiles, including sterically hindered and acid sensitive ones. A variety of functional groups are tolerated on the 1,3‐disubstituted allene substrate, and the former are in fact necessary for excellent regioselectivities. Control experiments suggest that the excellent regioselectivity is determined by inductive withdrawing effects rather than chelation control or steric differentiation.

## Experimental Section

### General procedure

(IPr)AuCl (10 mol %), followed by AgOTf (10 mol %) were added to a solution of allene **4** (0.14 mmol, 1 equiv), alcohol nucleophile (1.4 mmol, 10 equiv) and DMF (0.14 mL) at 0 °C. The resulting reaction mixture was allowed to stir at 0 °C for 24 h. The crude mixture was then filtered through two plugs of silica, washing with Et_2_O. The filtrate was washed with water and brine, and the resulting organic layer was dried (MgSO_4_) and concentrated in vacuo. The crude product was purified by column chromatography to yield products **6**. Full experimental procedures, characterisation for all new compounds and copies of ^1^H and ^13^C NMR spectra are provided in the Supporting Information.

## Supporting information

As a service to our authors and readers, this journal provides supporting information supplied by the authors. Such materials are peer reviewed and may be re‐organized for online delivery, but are not copy‐edited or typeset. Technical support issues arising from supporting information (other than missing files) should be addressed to the authors.

Supplementary
