# Supplementary material for: Chirality Transfer in Gold(I)‐Catalysed Hydroalkoxylation of 1,3‐Disubstituted Allenes
Source: Chemistry. 2016 Nov 11;22(51):18593–600. doi: 10.1002/chem.201603918 (PMC5215423; doi:10.1002/chem.201603918)
Supplement: Supplementary file 1 — Supplementary [file CHEM-22-18593-s001.pdf]

CHEMISTRY
A European Journal

Supporting Information

Chirality Transfer in Gold(I)-Catalysed Hydroalkoxylation of 1,3-Disubstituted Allenes

Stacey Webster, Daniel R. Sutherland, and Ai-Lan Lee*[a]

chem_201603918_sm_miscellaneous_information.pdf

Supporting Information

Contents

1. **General Experimental……………………………………………….………….3**
2. **Optimisation and Controls…………………………..…………………...….….…4**
3. **Determination of Absolute Stereochemistry……………………..…………………7**
4. **Experimental…………………………………………………………….…......15**
5. **^{1}H-, ^{13}C-NMR and HPLC/GC traces………………………………………………50**
6. **References ……………………………………………………………………140**

1) General Experimental Section

^{1}H NMR spectra was recorded on Bruker AV 300 and AV 400 spectrometers at 300 and 400 MHz respectively and referenced to residual solvent. ^{13}C NMR spectra were recorded using the same spectrometers at 75 and 100 MHz respectively. Chemical shift data are quoted in parts per million (ppm) and are referenced to tetramethylsilane (TMS) or to residual solvent peaks ($CDCl_3$ at δ_H 7.26). *J* values are given in Hz and s, d, dd, dt, ddt, dtd, t, td, tt, q, qd, qt, qn and m abbreviations correspond to singlet, doublet, doublet of doublet, doublet of triplet, doublet of doublet of triplet, doublet of triplet of doublet, triplet, triplet of doublet, triplet of triplet, quartet, quartet of doublet, quartet of triplet, quintet and multiplet. Mass spectra were obtained at the EPSRC National Mass Spectrometry Service Centre in Swansea and APCI represents atmospheric pressure chemical ionisation. Infrared spectra were obtained on Perkin-Elmer Spectrum 100 FT-IR Universal ATR Sampling Accessory, deposited neat or as a chloroform solution to a diamond/ZnSe plate. Flash column chromatography was carried out using Matrix silica gel 60 from Fisher Chemicals and TLC was performed using Merck silica gel 60 F254 pre-coated sheets and visualised by UV (254 nm) or stained by the use of aqueous acidic $KMnO_4$ or aqueous acidic ceric ammonium molybdate as appropriate. Chemicals were purchased from Sigma-Aldrich, Acros, Fisher and Apollo chemical companies and used without further purification unless otherwise stated. THF, DCM and DMF were dried using an MBRAUN SPS-800 solvent purification system. Diethyl ether was purified by distilling over CaH. High performance liquid chromatography (HPLC) was carried out on Agilent Technologies 1120 Compact LC. Gas chromatography was carried out on a Shimadzu GC2014 with FID.

The gold(I)-catalysed reactions were carried out in screw cap 1 dram vials unless otherwise indicated. No special precautions to exclude air or moisture were taken unless otherwise indicated.

2) Optimisation and Controls

Initial control reactions without molecular sieves

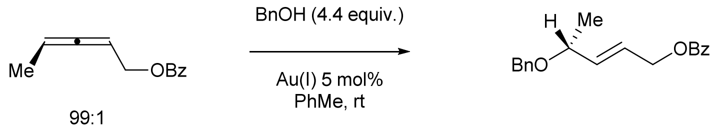

Entry	Catalyst	Time	Yield	e.r
1	IPrAuCl/AgOTf	20 min	83%	89.5:10.5 (79% ee)
2	IPrAuCl/AgOTf	20 min	95%	93:7 (86% ee)
3	PPh_3AuNTf_2	4 h	95%	92:8 (84% ee)

For comparison, Entry 1 is Widenhoefer's original results in *Org. Lett* **2008**, *10*, 2079. [1]

Entry 2 is a repeat of the conditions reported in *Org. Lett* **2008**, *10*, 2079.[1]

Varying amounts of powdered 4 Å molecular sieves (MS)

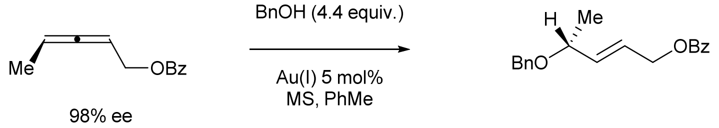

Entry	Catalyst	Time	Temp	MS	Yield	e.r
1	PPh_3AuNTf_2	4 h	rt	9 mg	Mainly SM	-
2	PPh_3AuNTf_2 (2 x 5 mol%) 7+17 h	24 h	rt	4 mg	30%	97:3
3	PPh_3AuNTf_2	4 + 1 h	rt + 60 ^{o}C	1 mg	43%	95:5
4	IPrAuCl/AgOTf	24 h	rt	7 mg	Mainly SM	-

For the following optimisations, powdered 4 Å MS were used unless otherwise stated.

Portionwise addition of Au(I) every 30 mins

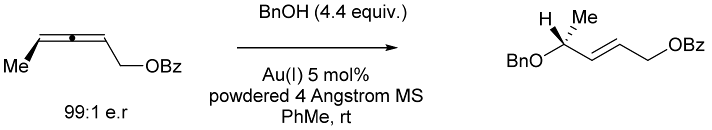

Entry	Catalyst	Time	Yield	e.r
1	PPh_3AuNTf_2	24 h	30%	97:3
2	PPh_3AuNTf_2 (4 x 5 mol%)	4.5 h	35%	95:5

Equiv. of BnOH

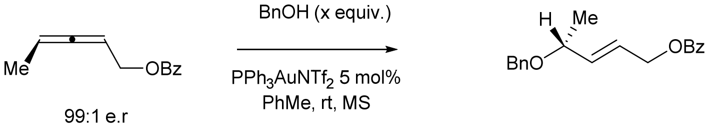

Entry	Equiv. of BnOH	Time	Yield	e.r
1	1.1	20 h	Mainly SM	-
2	4.4	24 h	30%	97:3
3	10	4 days	17%	98:2

Temperature

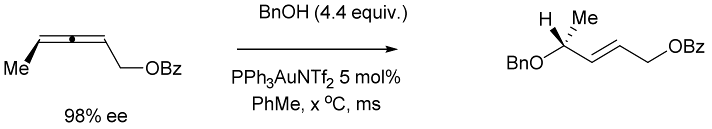

Entry	Temperature	Time	Yield	e.r
1	rt	24 h	30%	97:3
2	50	4 h	38%	92:8

Additives

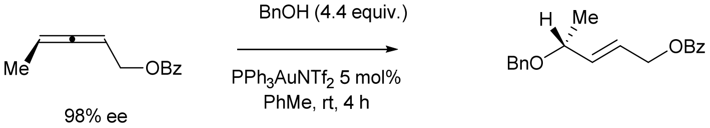

Entry	Time	Additive	Yield	e.r
1	20 h	4 mg of 4 Å MS + 1 equiv. of water	Mainly SM	-
2	4 days	4 mg of activated 4 Å MS	7%	98:2
3	4 h	4 mg of 4 Å MS (fine powder)	Only SM	-
4	4 h	4 mg of 4 Å MS (beads crushed into powder)	26%	97:3
5	4 h	4 mg of 5 Å MS (beads crushed into powder)	60%	83:17
6	4 h	4 mg of 5 Å MS (beads crushed into powder)	61%	95:5
7	4 h	4 mg of silica	50%	94:6
8	4 h	4 mg of neutral alumina	28%	97.5:2.5
9	4 h	2 mg of neutral alumina	58%	96:4
10	4 h	4 mg of basic alumina	41%	96:4
11	4 h	4 mg of acidic alumina	48%	97:3
12	4 h	1-5 mol% Ga(OTf)$_3$	34%	98:2

Although Entry 6 was promising, the results using molecular sieves were not always reproducible (e.g. Entry 5 vs. 6). Yields also tended to be low when ees are high, so this approach was abandoned in favour of Conditions B in the main article.

Control to ascertain whether *E* selectivity is due to thermodynamic control:

The allylic ether **SI-1** shown below, containing a 7:1 ratio of *E*:*Z* isomers was synthesised using an alternative procedure.[1] The *E*/*Z* mixture of **SI-1** Was then subjected to the gold-catalysed hydroalkoxylation reaction as shown below. No change to the *E*:*Z* ratio was observed, implying that the excellent *E*–selectivity in the hydroalkoxylation reaction is not due to thermodynamic control.

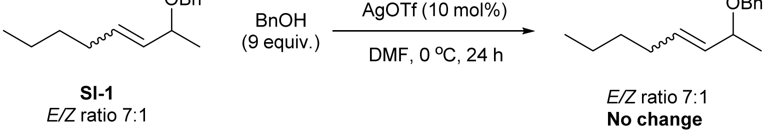

3) Determination of Absolute Configuration

Widenhoefer and co-workers have previously shown that under the conditions shown in Scheme 1 the *R*-enantiomer is produced. This was determined by comparing product **6bb** to known compounds.[2]

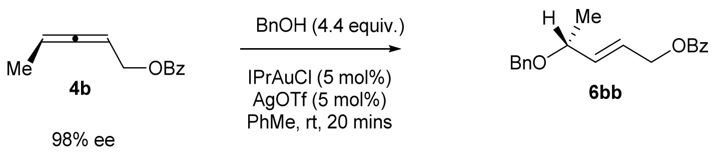

Their conditions to produce *R*-**6bb** were repeated and produced the following HPLC trace (Figure S1).

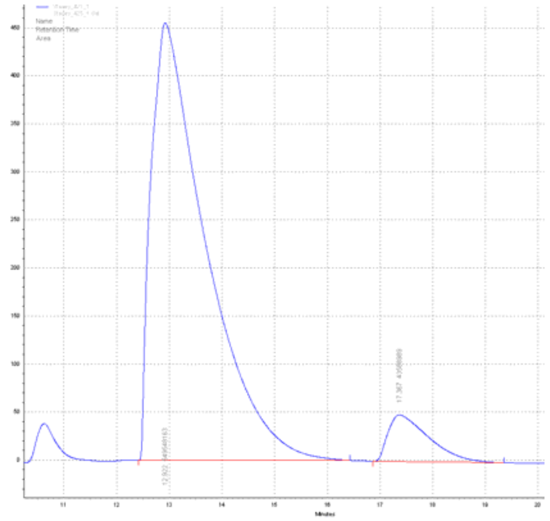

Figure S1: HPLC trace of product **6bb** obtained under Widenhoefer's conditions.[1]

Under our conditions, the following HPLC trace was obtained (Figure S2). This suggests that our conditions are also producing the *R*-enantiomer of **6bb**.

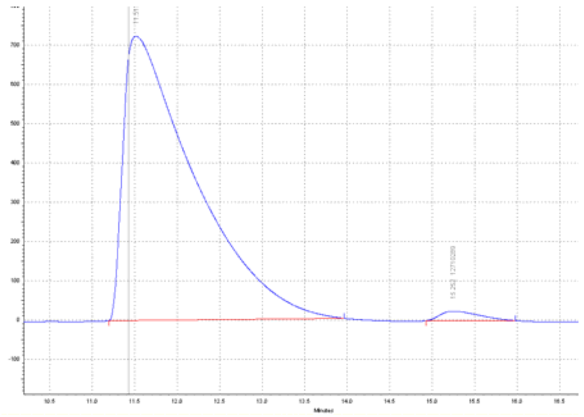

Figure S2: HPLC trace of product **6bb** under our conditions.

Determination of Absolute Configuration

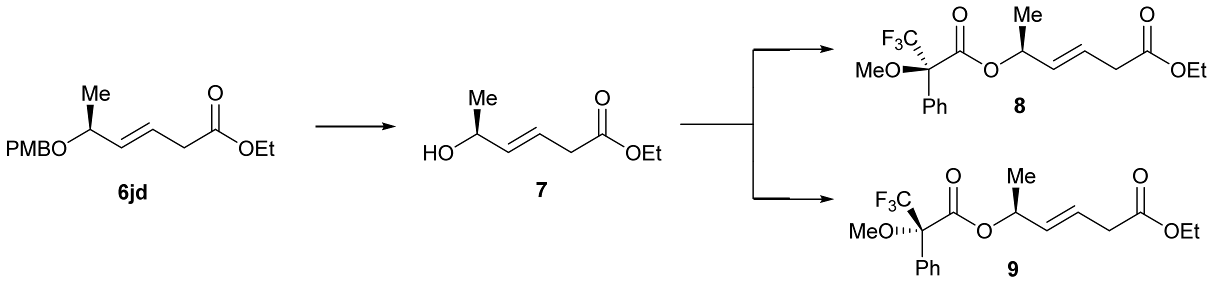

According to T. R. Hoye, C. S. Jeffrey, F. Shao, *Nat. Protoc.* **2007**, *2*, 2451-2458:[3] in their most represented configuration the CF_3 group, carbonyl and the H of the chiral carbon are in the same plane and the phenyl group is in close proximity to either the methyl group or alkene CH. The phenyl group shields the nuclei which it is close to and causes an upfield shift in the NMR. This means that if the alcohol is the *S*-enantiomer as expected the *S*-Moshers ester will have a more upfield methyl group signal than the *R*-Moshers ester and the *R*-Moshers ester will have a more upfield alkene CH signal than the *S*-Moshers ester. This can be represented as:

$$\Delta\delta^{SR}_{Me} = \delta_{Me}(S) - \delta_{Me}(R)$$

$$\Delta\delta^{SR}_{CHalkene} = \delta_{CHalkene}(S) - \delta_{CHalkene}(R)$$

where if $\Delta\delta^{SR}_{Me} < 0$ and $\Delta\delta^{SR}_{CHalkene} > 0$ the alcohol is the *S*-enantiomer.

$$\Delta\delta^{SR}_{Me} = \delta_{Me}(S) - \delta_{Me}(R)$$

^{1}H NMR $\Delta\delta^{SR}_{Me} = 1.39 - 1.46 = -\ 0.17$

^{13}C NMR $\Delta\delta^{SR}_{Me} = 19.79 - 20.02 = -\ 0.23$

$$\Delta\delta^{SR}_{CHalkene} = \delta_{CHalkene}(S) - \delta_{CHalkene}(R)$$

^{1}H NMR $\Delta\delta^{SR}_{Me} = 5.77^{*} - 5.69^{*} = +\ 0.08$

*most downfield point of multiplet

^{13}C NMR could not be assigned without ambiguity

From this analysis, alcohol **7**, and therefore **6jd** are both (*S*)-enantiomers.

drsh24c202
1H 300.1MHz Job 52835 Sutherland Daniel R 24C202 CDCl3 25.0°C
*

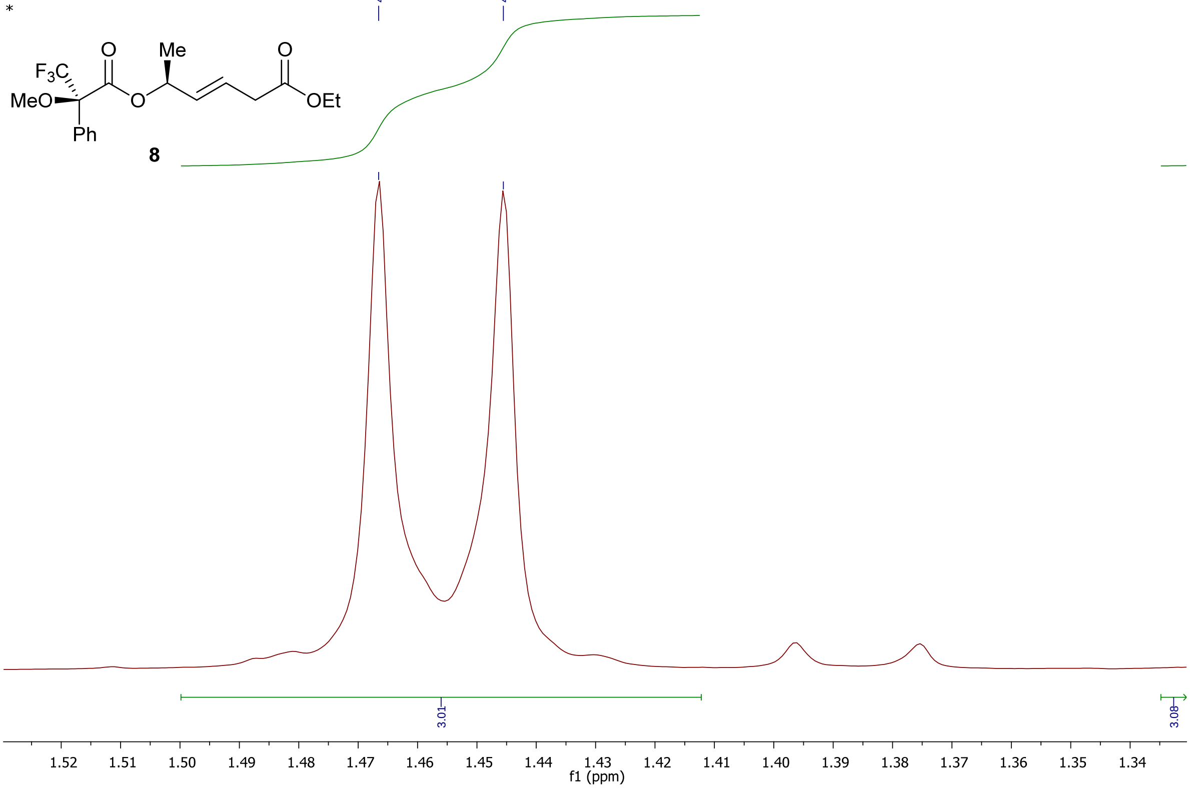

drsh24d101
1H 300.1MHz Job 52864 Sutherland Daniel R 24D101 CDCl3 25.0°C
*

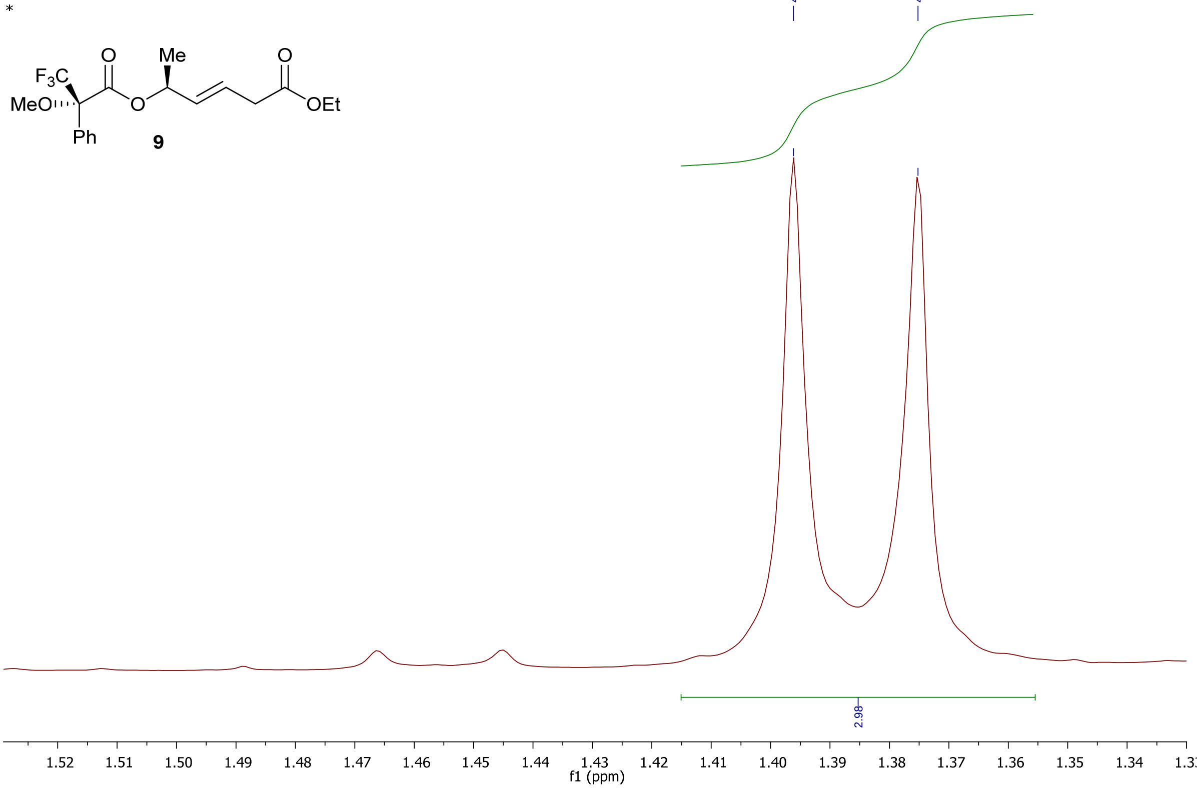

(*S*,*E*)-Ethyl 5-hydroxyhex-3-enoate 7

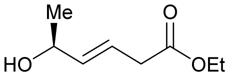

7

Following a general procedure by Takeda *et al.*[4] 2,3-dichloro-5,6-dicyano-1,4-benzoquinone (120 mg, 0.53 mmol, 1.2 equiv.) was added to a stirring solution of PMB ester **6jd** (123 mg, 0.44 mmol, 1 equiv.) in DCM (6 ml) and water (0.3 ml), and stirred for 1 h. A saturated solution of $NaHCO_3$ (20 ml) was added and the layers were separated. The aqueous layer was extracted with DCM, and the combined organic layers washed with brine, dried over $MgSO_4$ and concentrated. The mixture was purified by column chromatography (eluent: 2:1 hexane/EtOAc) to yield product **7** as a colourless oil (50.6 mg, 0.31 mmol, 71%)

R_f 0.23 (2:1 hexane/EtOAc); ν_{max}/cm^{-1} 3412 (OH), 2975 (C-H), 1733 (C=O); ^{1}H NMR (300 MHz, $CDCl_3$) δ 5.73-5.89 (1H, m, =C<u>H</u>CH_2), 5.62-5.73 (1H, m, =C<u>H</u>CH), 4.34 (1H, app. qn, *J* = 6.2 Hz, OCH), 4.17 (2H, q *J* = 7.1, Hz, OCH_2), 3.08 (2H, d, *J* = 6.6 Hz, CHC<u>H</u>$_2$), 1.75 (1H, br d, *J* = 14.7 Hz, OH), 1.23-1.35 (6H, m, CH_2C<u>H</u>$_3$ + CHC<u>H</u>$_3$); ^{13}C NMR (75 MHz, $CDCl_3$) δ 171.7 (C), 138.1 (CH), 122.1 (CH), 68.4 (CH), 60.7 (CH_2), 37.6 (CH_2), 23.1 (CH_3), 14.2 (CH_3).

(*S*,*E*)-Ethyl 5-(((R)-3,3,3-trifluoro-2-methoxy-2-phenylpropanoyl)oxy)hex-3-enoate 8

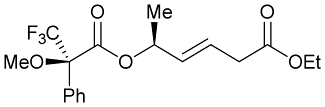

8

Following a general procedure by Renner *et al.*[5] to a stirring solution of alcohol **7** (19 mg, 0.12 mmol, 1.0 equiv.) in DCM (3.0 ml) (*R*)-(+)-α-methoxy-α-trifluoromethylphenylacetic acid (84 mg, 0.36 mmol, 3.0 equiv.), dicyclohexylcarbodiimide (74 mg, 0.36 mmol, 3.0 equiv.), and 4-dimethylaminopyridine (44 mg, 0.36 mmol, 3.0 equiv.) were added sequentially. The reaction mixture was stirred for 1 hour at room temperature, passed through a silica plug (DCM) dried over $MgSO_4$ and concentrated. The mixture was purified by column chromatography (eluent: 14:1 then 10:1 hexane/Et_2O) to yield product **8** as a colourless oil (34.2 g, 0.09 mmol, 73%).

*R*f 0.36 (9:1 hexane/EtOAc); ν_{max}/cm^{-1} 2984 (C-H), 1737 (C=O), 1164 (C-O-C); ^{1}H NMR (300 MHz, $CDCl_3$) δ 7.48-7.60 (2H, m, Ar-H), 7.36-7.48 (3H, m, Ar-H), 5.89 (1H , dt, *J* = 14.6, 7.0 Hz, =C<u>H</u>CH_2), 5.51-5.69 (2H, m, =C<u>H</u>CH + OCH), 4.17 (2H, q, *J* = 7.1 Hz, OC<u>H</u>$_2$$CH_3$), 3.59 (3H, s, OCH_3), 3.07 (2H, d, *J* = 7.0 Hz, =CHC<u>H</u>$_2$), 1.46 (3H, d, *J* = 6.3 Hz, CHC<u>H</u>$_3$), 1.28 (3H, t, *J* = 7.1 Hz, CH_2C<u>H</u>$_3$); ^{13}C NMR (75 MHz, $CDCl_3$) δ 171.0 (C), 165.6 (C), 132.4 (C), 131.9 (CH), 129.5 (CH), 128.3 (CH), 127.3 (CH), 126.1 (CH), 123.3 (q, *J* = 288.6 Hz, CF_3), 84.51 (q, *J* = 27.5 Hz, C), 73.1 (CH), 60.8 (CH_2), 55.4 (CH_3), 37.6 (CH_2), 20.0 (CH_3), 14.1 (CH_3); $[\alpha]_D^{22°C}$ = +36.0 (c = 1.11 in $CHCl_3$).

(*S*,*E*)-Ethyl 5-(((S)-3,3,3-trifluoro-2-methoxy-2-phenylpropanoyl)oxy)hex-3-enoate 9

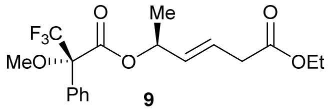

9

Following the same procedure as for Moshers ester **8** replacing (*R*)-(+)-α-methoxy-α-trifluoromethylphenylacetic acid with (*S*)-(+)-α-methoxy-α-trifluoromethylphenylacetic acid gave Moshers ester **9.**

*R*f 0.36 (9:1 hexane/EtOAc); ^{1}H NMR (300 MHz, $CDCl_3$) δ 7.49-7.62 (2H, m, Ar-H), 7.35-7.49 (3H, m, Ar-H), 5.96 (1H , dt, *J* = 14.6, 7.0 Hz, =C<u>H</u>CH_2), 5.58-5.77 (2H, m, =C<u>H</u>CH + OCH), 4.17 (2H, q, *J* = 7.1 Hz, OC<u>H</u>$_2$$CH_3$), 3.59 (3H, s, OCH_3), 3.10 (2H, d, *J* = 7.0 Hz, =CHC<u>H</u>$_2$), 1.39 (3H, d, *J* = 6.3 Hz, CHC<u>H</u>$_3$), 1.28 (3H, t, *J* = 7.1 Hz, CH_2C<u>H</u>$_3$); ^{13}C NMR (75 MHz, $CDCl_3$) δ 171.0 (C), 165.7 (C), 132.4 (C), 132.0 (CH), 129.5 (CH), 128.4 (CH), 127.3 (CH), 126.3 (CH), 123.3 (q, *J* = 288.6 Hz, CF_3), 84.51 (q, *J* = 27.5 Hz, C), 73.2 (CH), 60.8 (CH_2), 55.4 (CH_3), 37.6 (CH_2), 19.8 (CH_3), 14.1 (CH_3); $[\alpha]_D^{22°C}$ = -41.4 (c = 1.11 in $CHCl_3$).

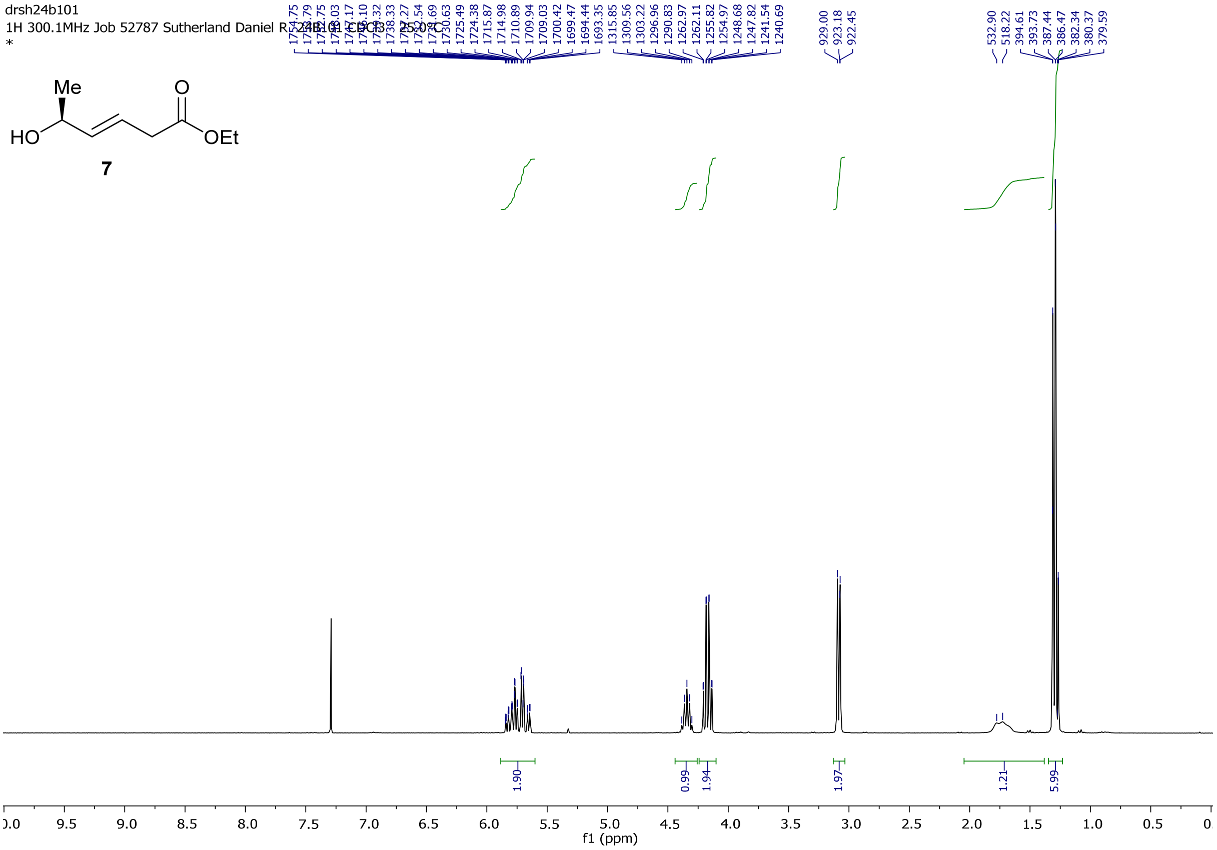
drsh24b101
Me
O
HO
OEt
7
1.90
0.99
1.94
1.97
1.21
5.99
f1 (ppm)

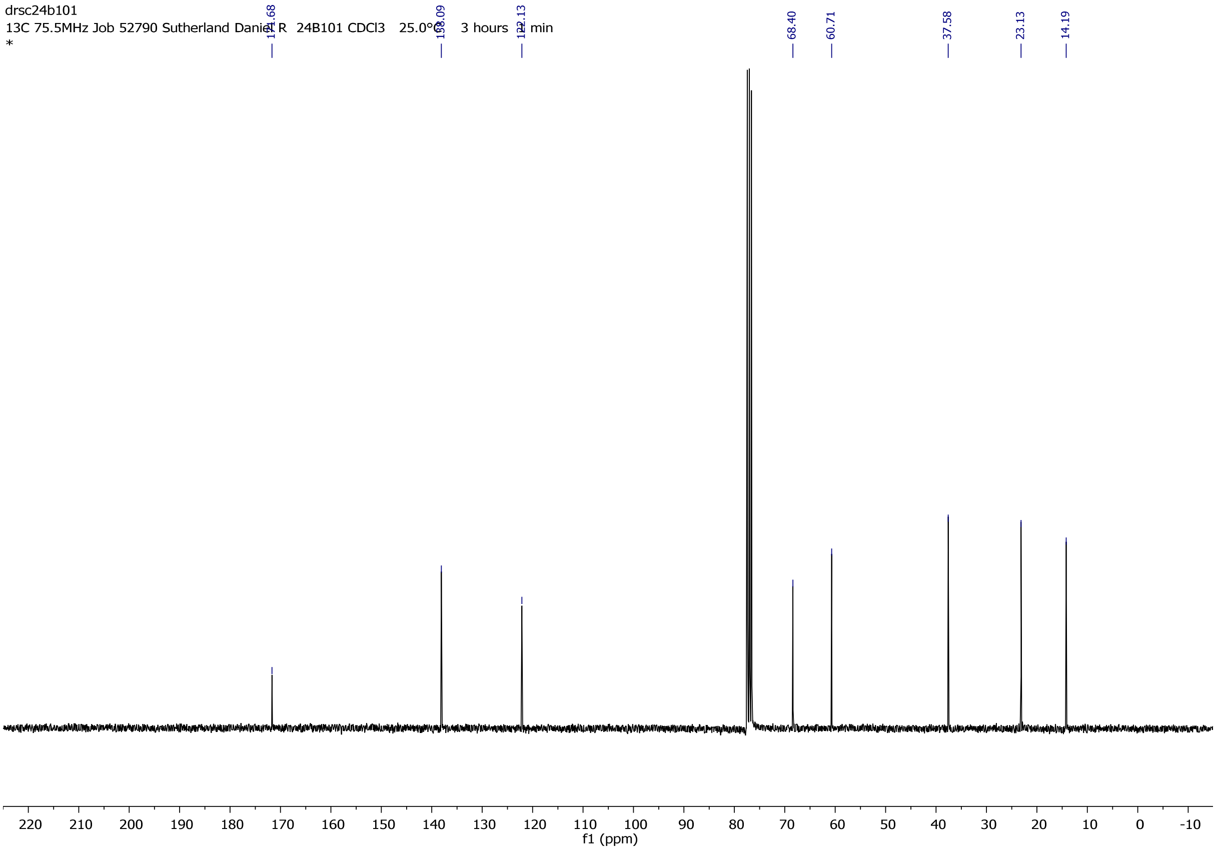
drsc24b101
68.40
60.71
37.58
23.13
14.19
f1 (ppm)

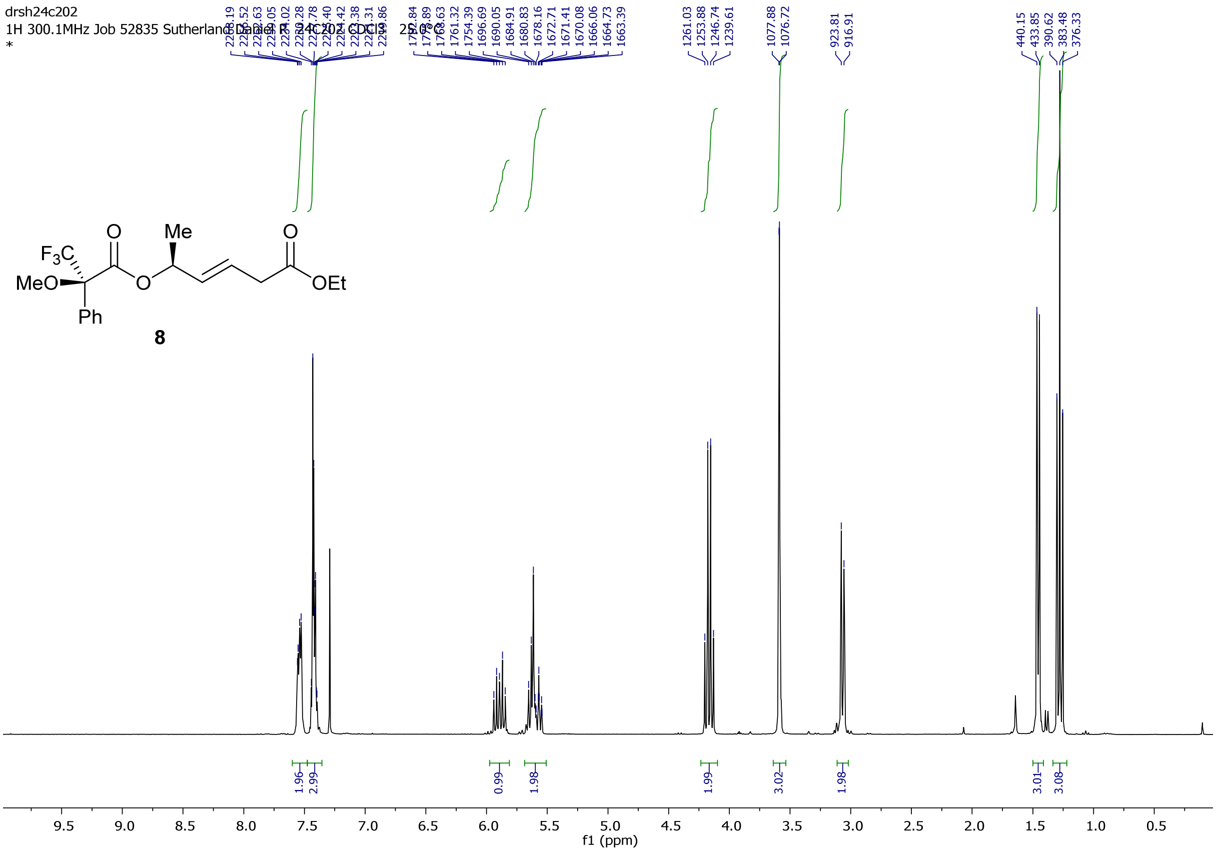
drsh24c202
1H 300.1MHz Job 52835 Sutherland
*
1261.03
1253.88
1246.74
1239.61
1077.88
1076.72
923.81
916.91
440.15
433.85
390.62
383.48
376.33
8
1.96
2.99
0.99
1.98
1.99
3.02
1.98
3.01
3.08
f1 (ppm)

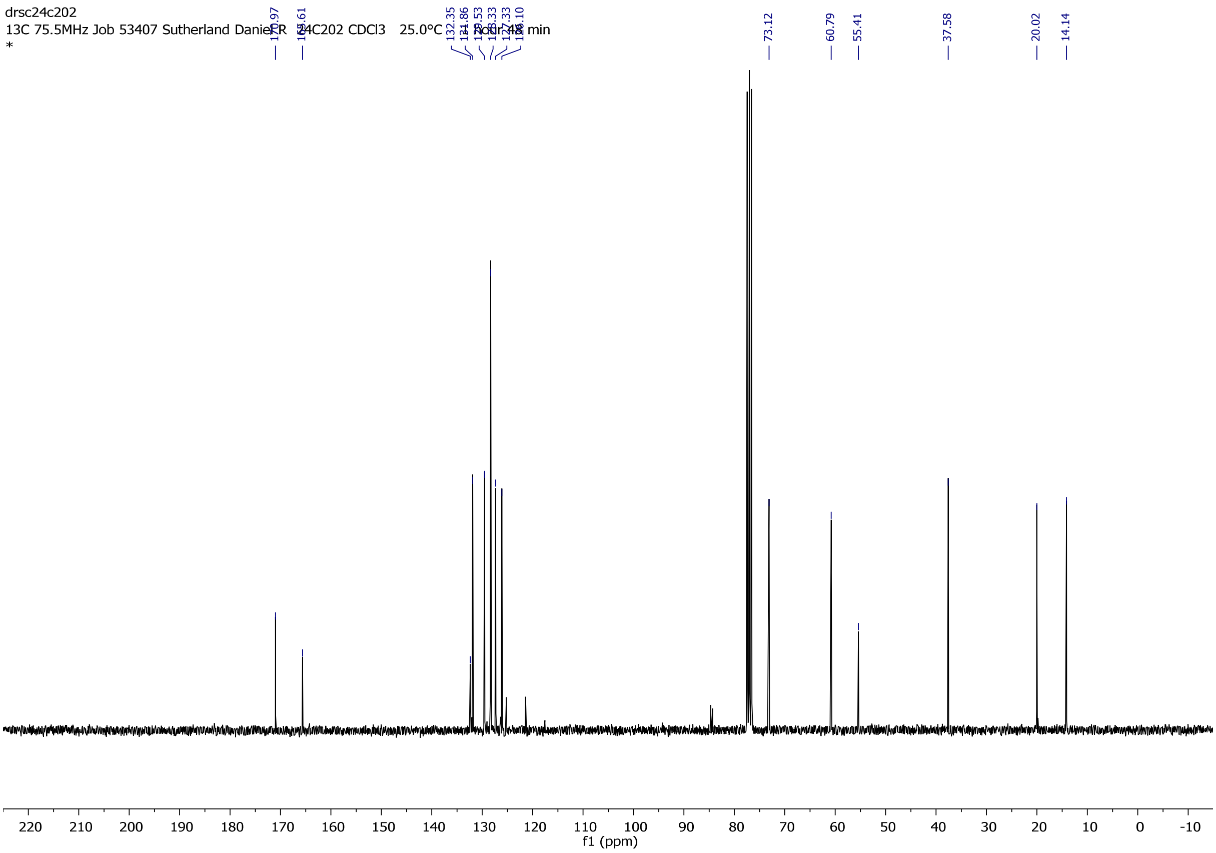
drsc24c202
25.0°C
*
73.12
60.79
55.41
37.58
20.02
14.14
f1 (ppm)

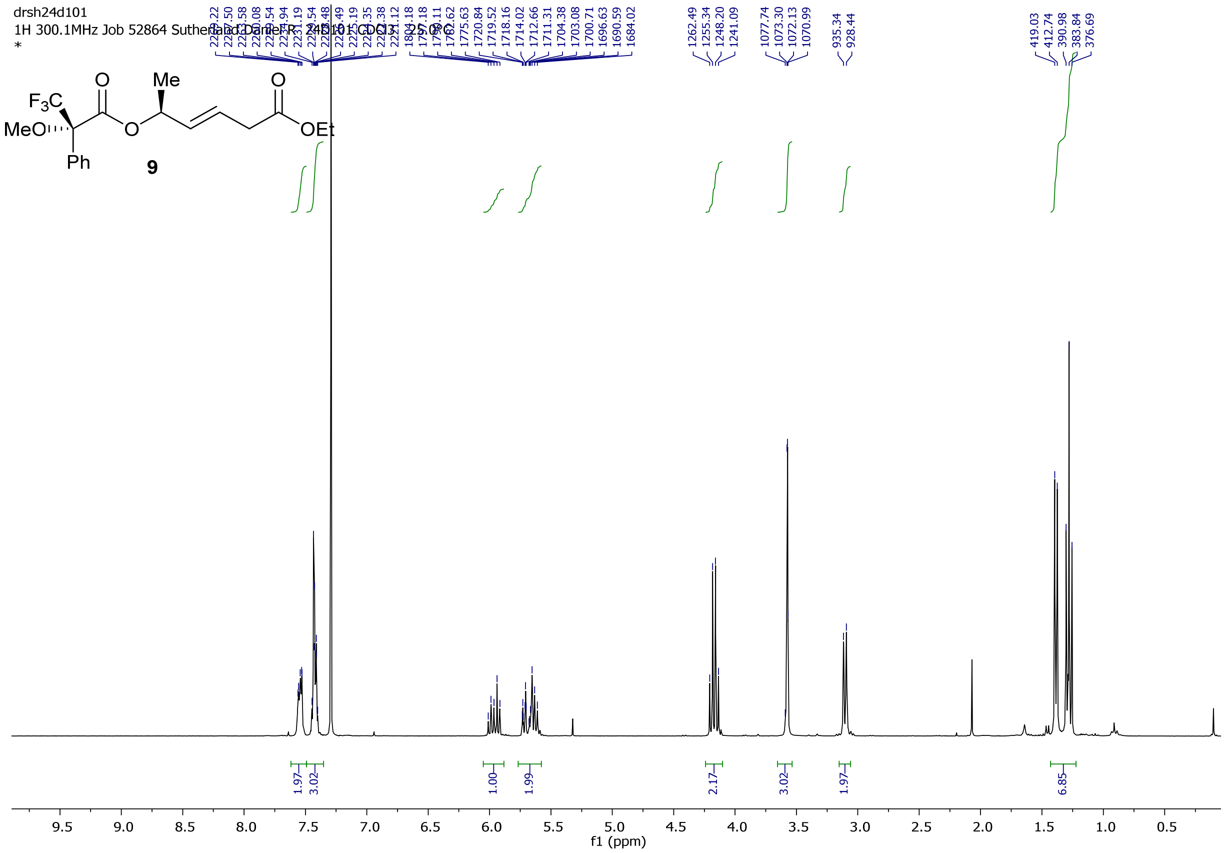
drsh24d101
*
Me
O
O
F_3C
MeO
O
O
OEt
Ph
9
1262.49
1255.34
1248.20
1241.09
1077.74
1073.30
1072.13
1070.99
935.34
928.44
419.03
412.74
390.98
383.84
376.69
1.97
3.02
1.00
1.99
2.17
3.02
1.97
6.85
f1 (ppm)

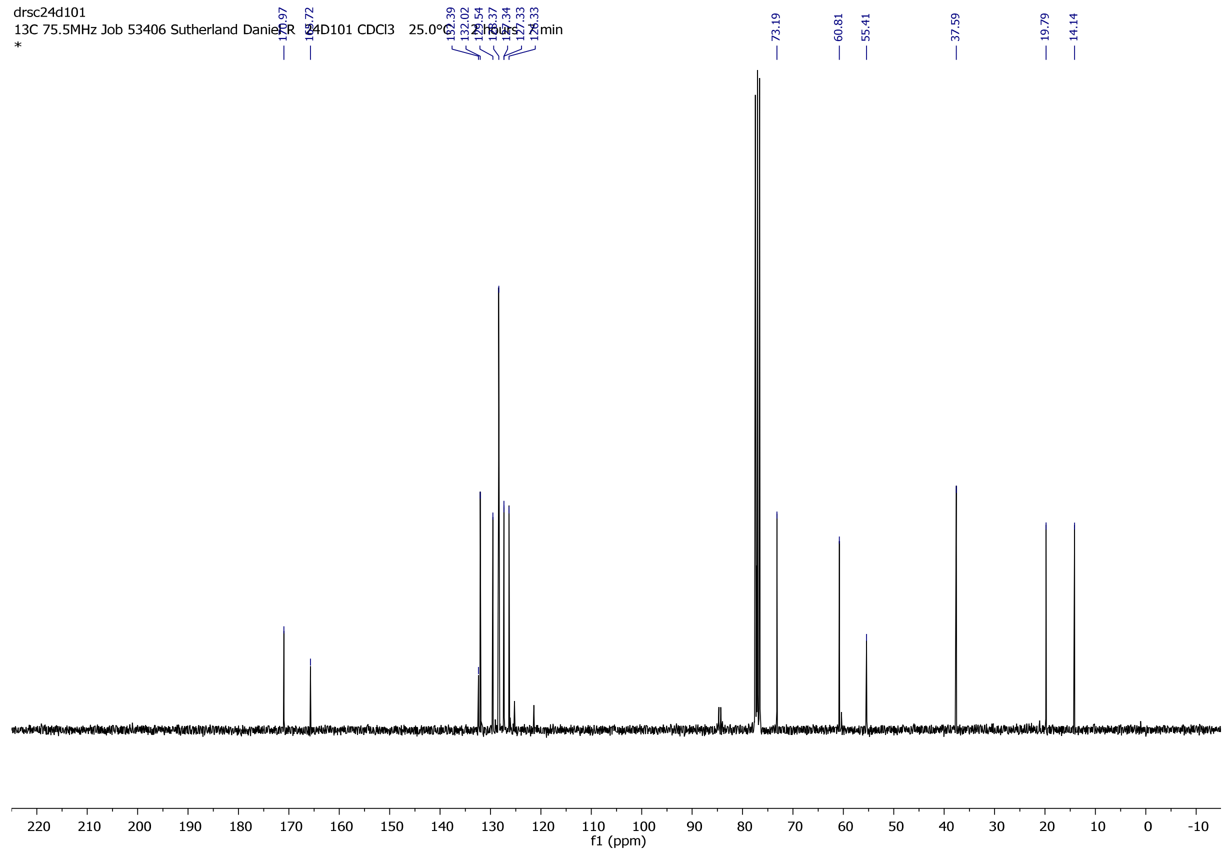
drsc24d101
13C 75.5MHz Job 53406 Sutherland Daniel R 24D101 CDCl3 25.0°C
*
73.19
60.81
55.41
37.59
19.79
14.14
f1 (ppm)

4) Experimental

Starting material synthesis

Note that although we followed a multistep procedure detailed below to access the enantiopure allenols (e.g. **12a-12c**), Ma has recently published a procedure to access these allenols in high ee *via* a one-step procedure from readily available starting materials.[6]

2-(((*R*)-But-3-yn-2-yl)oxy)tetrahydro-2*H*-pyran (10a)[2]

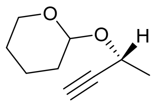

10a

To a solution of (*R*)-1-butyn-3-ol (1.83 g, 26.1 mmol, 1 equiv., 97% ee), 3,4-dihydro-2H-pyran (2.9 ml, 31.3 mmol, 1.2 equiv.) and DCM (20 ml) at 0 °C was added TsOH (69 mg, 1.5 mol%). The solution was stirred for 2 hours at 0 °C. The reaction was diluted with Et_2O and quenched with a saturated solution of $NaHCO_3$ and extracted with Et_2O. The combined organic layers were dried over $MgSO_4$ and concentrated. The mixture was purified by column chromatography (eluent 10:1 pentane:ether) to yield product **10a** as a colourless oil (3.56 g, 23.1 mmol, 89%) in a 1:0.9 mixture of diastereomers.

R_f 0.64 (15:1 pentane/Et_2O); ν_{max}/cm^{-1} 3291 (C≡C-H), 2940, 2870 (C-H), 1162 (C-O-C); ^1H NMR (300 MHz, $CDCl_3$) δ 4.93 (1H, dd, *J* = 4.3, 2.9 Hz, OC<u>H</u>O, major), 4.76 (1H', t, *J* = 3.2 Hz, OCHO, minor), 4.54 (1H, qd, *J* = 6.7, 2.0 Hz, $CH_3C\underline{H}$, major), 4.45 (1H', qd, *J* = 6.7, 2.2 Hz, $CH_3C\underline{H}$, minor), 3.93-4.03 (1H', m, $OCHC\underline{H}_2CH_2$, minor), 3.76-3.86 (1H, m, $OCHC\underline{H}_2CH_2$, major), 3.47-3.57 (1H + 1H', m, $OCHC\underline{H}_2CH_2$, major + minor), 2.41 (1H', d, *J* = 2.2 Hz, C≡CH, minor), 2.35 (1H, d, *J* = 2.0 Hz, C≡CH, major), 1.76-1.81 (1H + 1H', m, alkyl H's, major + minor), 1.64-1.76 (1H + 1H', m, alkyl H's, major + minor), 1.49-1.64 (4H + 4H', m, alkyl H's, major + minor), 1.46 (3H, d, *J* = 6.7 Hz, $C\underline{H}_3CH$, major), 1.43 (3H', d, *J* = 6.7 Hz, $C\underline{H}_3CH$, minor); ^{13}C NMR (75.5 MHz, $CDCl_3$) δ 97.3 (CH, minor), 96.1 (CH, major), 84.8 (C, minor), 83.8 (C, major), 72.6 (CH, major), 72.0 (CH, minor), 62.7 (CH, major), 62.4 (CH_2, minor), 62.3 (CH_2, major), 60.7 (CH, minor), 30.7 (CH_2, minor), 30.6 (CH_2, major), 25.6 (CH_2, major), 25.5 (CH_2, minor), 22.2 (CH_3, major), 21.9 (CH_3, minor), 19.6 (CH_2, major), 19.2 (CH_2, minor).

2-(((*R*)-Oct-3-yn-2-yl)oxy)tetrahydro-2*H*-pyran (10b)[2]

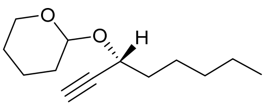

10b

To a solution of (*R*)-1-octyn-3-ol (1.13 g, 8.92 mmol, 1 equiv., 97% ee), 3,4-dihydro-2H-pyran (1.0 ml, 10.7 mmol, 1.2 equiv.) and DCM (6.9 ml) at 0 °C was added TsOH (23 mg, 1.5 mol%). The solution was stirred for 2 hours at 0 °C. The reaction was diluted with Et_2O and quenched with a saturated solution of $NaHCO_3$ and extracted with Et_2O. The combined organic layers were dried over $MgSO_4$ and concentrated. The mixture was purified by column chromatography (eluent 10:1 hexane:ether) to yield product **10b** as a colourless oil (1.76 g, 8.38 mmol, 94%) in a 1:0.3 mixture of diastereomers.

ν_{max}/cm^{-1} 3309 (C≡C-H), 2939, 2860 (C-H), 1157 (C-O-C); ^{1}H NMR (300 MHz, $CDCl_3$) δ 4.98 (1H, t, *J* = 2.9 Hz, OC<u>H</u>O, major), 4.75 (1H', t, *J* = 3.3 Hz, OC<u>H</u>O, minor), 4.41 (1H, td, *J* = 6.7, 2.0 Hz, OC<u>H</u>CH_2, major), 4.28 (1H', td, *J* = 6.7 Hz, 2.2 Hz, OC<u>H</u>CH_2, minor), 3.97-4.07 (1H', m, OC<u>H</u>$_2CH_2$, minor), 3.76-3.86 (1H, m, OC<u>H</u>$_2CH_2$, major), 3.49-3.59 (1H + 1H', m, OC<u>H</u>$_2CH_2$, major + minor), 2.43 (1H', d, *J* = 2.2 Hz, <u>H</u>C≡C, minor), 2.37 (1H, d, *J* = 2.0 Hz, <u>H</u>C≡C, major), 1.68-1.89 (4H + 4H', m, alkyl H's, major + minor), 1.42-1.67 (6H + 6H', m, alkyl H's, major + minor), 1.24-1.39 (4H + 4H', m, alkyl H's, major + minor), 0.87-0.97 (3H + 3H', m, CH_3, major + minor); ^{13}C NMR (75.5 MHz, $CDCl_3$) δ 98.3 (CH, minor), 95.7 (CH, major), 84.1 (C, minor), 83.1 (C, major), 73.2 (CH, major), 72.6 (CH, minor), 67.3 (CH, minor), 64.9 (CH, major), 62.5 (CH_2, major), 62.4 (CH_2, minor), 35.8 (CH_2, major), 35.7 (CH_2, minor), 31.7 (CH_2, major), 31.6 (CH_2, minor), 30.6 (CH_2, major + minor), 25.6 (CH_2, major), 25.5 (CH_2, minor), 25.2 (CH_2, major), 24.9 (CH_2, minor), 22.8 (CH_2, major), 22.7 (CH_2, minor), 19.5 (CH_2, major), 19.2 (CH_2, minor), 14.2 (CH_3, major), 14.1 (CH_3, minor).

(4*R*)-4-((Tetrahydro-2*H*-pyran-2-yl)oxy)pent-2-yn-1-ol (11a)[2]

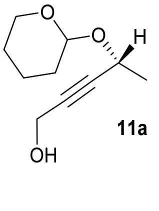

11a

n-BuLi (13 ml, 2.5 M in hexanes, 32.3 mmol, 1.4 equiv.) was added dropwise (over 20 mins) to a solution of **10a** (3.56 g, 23.1 mmol, 1 equiv.) in THF (77 ml) at -78 °C under Ar.

Allowed to warm to room temperature and paraformaldehyde (1.30 g, 43.0 mmol, 2.0 equiv.) was added. The reaction was stirred for a further 2 hours. The reaction was diluted with Et_2O and quenched with a saturated solution of NH_4Cl and extracted with Et_2O. The combined organic layers were dried over $MgSO_4$ and concentrated. The mixture was purified by column chromatography (eluent: 10:1 then 1:1 hexane/Et_2O) to yield product **11a** as a colourless oil as a 1:0.9 mixture of diastereomers (3.38 g, 18.3 mmol, 80%).

R_f 0.35 (1:1 hexane/Et_2O); ν_{max}/cm^{-1} 3416 (O-H), 2938, 2868 (C-H), 1114 (C-O-C); ^{1}H NMR (300 MHz, $CDCl_3$) δ 4.92 (1H, t, *J* = 3.0 Hz, OC<u>H</u>O, major), 4.76 (1H', t, *J* = 3.1 Hz, minor), 4.57 (1H, qt, *J* = 6.7, 1.6 Hz, C<u>H</u>CH_3, major), 4.49 (1H', qt, *J* = 6.6, 1.7 Hz, C<u>H</u>CH_3, minor), 4.29 (2H', d, *J* = 1.6 Hz, HOC<u>H</u>$_2$, minor), 4.27 (2H, d, *J* = 1.7 Hz, HOC<u>H</u>$_2$, major), 3.93-4.02 (1H', m, OC<u>H</u>$_2$$CH_2$, minor), 3.76-3.85 (1H, m, OC<u>H</u>$_2$$CH_2$, major), 3.47-3.57 (1H + 1H', m, OC<u>H</u>$_2$$CH_2$, major + minor), 2.04-2.18 (1H + 1H', m, OH, major + minor), 1.79-1.90 (1H + 1H', m, alkyl H's, major + minor), 1.65-1.77 (1H + 1H', m, alkyl H's, major + minor), 1.48-1.65 (4H + 4H', m, alkyl H's, major + minor), 1.45 (3H, d, *J* = 6.7 Hz, CH_3, major), 1.42 (3H', d, *J* = 6.6 Hz, CH_3, minor); ^{13}C NMR (75.5 MHz, $CDCl_3$) δ 97.3 (CH, minor), 95.9 (CH, major), 86.5 (C, minor), 85.5 (C, major), 83.1 (C, major), 82.4 (C, minor), 62.7 (CH, minor), 62.6 (CH_2, major), 62.5 (CH_2, minor), 60.9 (CH, major), 51.2 (CH_2, minor), 51.1 (CH_2, major), 30.7 (CH_2, minor), 30.6 (CH_2, major), 25.53 (CH_2, major), 25.46 (CH_2, minor), 22.2 (CH_3, major), 21.9 (CH_3, minor), 19.5 (CH_2, major), 19.3 (CH_2, minor).

(4*R*)-4-((Tetrahydro-2*H*-pyran-2-yl)oxy)non-2-yn-1-ol (11b)[2]

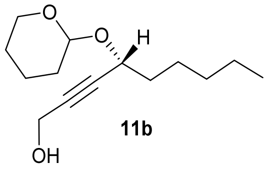

11b

n-BuLi (6.6 ml, 1.6 M in hexanes, 10.6 mmol, 1.4 equiv.) was added dropwise (over 20 mins) to a solution of **10b** (1.59 g, 7.57 mmol, 1 equiv.) in THF (20 ml) at -78 °C under Ar. Allowed to warm to room temperature and paraformaldehyde (462 mg, 15.4 mmol, 2.0 equiv.) was added. The reaction was stirred for a further 2 hours. The reaction was diluted with Et_2O and quenched with a saturated solution of NH_4Cl and extracted with Et_2O. The combined organic layers were dried over $MgSO_4$ and concentrated. The mixture was purified

by column chromatography (eluent: 1:1 hexane/Et_2O) to yield product **11b** as a colourless oil as a 1:0.1 mixture of diastereomers (1.42 g, 5.90 mmol, 78%).

ν_{max}/cm^{-1} 3407 (O-H), 2931, 2859 (C-H), 1113 (C-O-C); ^{1}H NMR (300 MHz, $CDCl_3$) δ 4.96 (1H, t, *J* = 2.9 Hz, OC<u>H</u>O), 4.44 (1H, tt, *J* = 6.7, 1.6 Hz, C<u>H</u>CH_2), 4.29 (2H, dd, *J* = 6.1, 1.6 Hz, HOC<u>H</u>$_2$), 3.75-3.85 (1H, m, OC<u>H</u>$_2$$CH_2$), 3.49-3.57 (1H, m, OC<u>H</u>$_2$$CH_2$), 1.67-1.86 (5H, m, alkyl H's), 1.42-1.64 (6H, m, alkyl H's), 1.26-1.36 (4H, m, alkyl H's), 0.89 (3H, d, *J* = 6.7 Hz, CH_3); ^{13}C NMR (75.5 MHz, $CDCl_3$) δ 95.5 (CH), 85.0 (C), 83.6 (C), 65.0 (CH), 62.3 (CH_2), 51.3 (CH_2), 35.9 (CH_2), 31.7 (CH_2), 30.6 (CH_2), 25.6 (CH_2), 25.3 (CH_2), 22.7 (CH_2), 19.4 (CH_2), 14.2 (CH_3).

(*S*)-Penta-2,3-dien-ol (12a)[2]

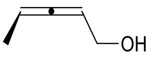

12a

A solution of **11a** (3.38 g, 18.3 mmol, 1.0 equiv.) in Et_2O (18 ml) was added to a solution of $LiAlH_4$ (1.26 g, 33.2 mmol, 1.8 equiv.) in Et_2O (30 ml) at 0 °C. The reaction was slowly warmed to room temperature and stirred for 18 hours. The reaction was then cooled to 0 °C and quenched with successive additions of water (4.0 ml), 15% NaOH (4.0 ml) and water (4.0 ml). The white suspension was filtered through celite and washed with Et_2O and then concentrated. The product **12a** was used without further purification.

(*S*)-Nona-2,3-dien-1-ol (12b)[2]

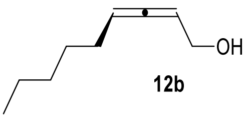

12b

A solution of **11b** (1.42 g, 5.92 mmol, 1.0 equiv.) in Et_2O (8 ml) was added to a solution of $LiAlH_4$ (472 mg, 12.4 mmol, 2.0 equiv.) in Et_2O (8 ml) at 0 °C. The reaction was slowly warmed to room temperature and stirred for 18 hours. The reaction was then cooled to 0 °C and quenched with successive additions of water (2.0 ml), 15% NaOH (2.0 ml) and water (2.0 ml). The white suspension was filtered through celite and washed with Et_2O and then concentrated. The product **12b** was used without further purification.

(*R*)-Hexa-3,4-dien-1-ol (12c)

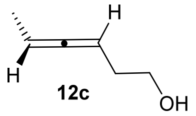

12c

(*R*)-Ethyl hexa-3,4-dienoate **4j** (491 mg, 3.5 mmol, 1 equiv.) in dry THF (8 ml) was added dropwise to stirring solution of lithium aluminium hydride (266 mg, 7.0 mmol, 2 equiv.) in THF (4 ml) at 0 °C and under argon. THF (4 ml) was added and the reaction mixture was stirred at room temperature for 1 h. 1 M NaOH solution was added dropwise until the solution turned viscous and then to a clear solution with white precipitate. The reaction mixture was passed through a Celite® plug and concentrated. The mixture was purified by column chromatography (eluent: 10:1 then 6:1 hexane/EtOAc) to yield product **12c** as a pale yellow oil (235 mg, 2.4 mmol, 69%).

R_f 0.35 (7:1 pentane/Et_2O); ν_{max}/cm^{-1} 3324 (OH), 2926 (C-H), 1965 (C=C=C); ^{1}H NMR (300 MHz, $CDCl_3$) δ 5.03-5.22 (2H, m, allene H), 3.73 (2H, t, *J* = 6.2 Hz, OCH_2), 2.28 (2H, app qd, *J* = 6.2, 3.0 Hz CHC$\underline{H}_2$), 1.70 (3H, dd, *J* = 6.9, 3.3 Hz CHC$\underline{H}_3$); ^{13}C NMR (75 MHz, $CDCl_3$) δ 205.5 (C), 86.6 (CH), 86.2 (CH), 62.0 (CH_2), 32.2 (CH_2), 14.5 (CH_3); $[\alpha]_D^{20°C}$ = -33.6 (c = 0.66 in $CHCl_3$) [lit.[7] $[\alpha]_D^{20°C}$ = -93.2 (c = 1.0 in MeOH)].

(*R*)-4-Phenylbut-3-yn-2-ol (13)[8]

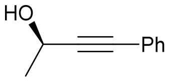

13

To a solution of iodobenzene (0.49 ml, 4.4 mmol, 1.1 equiv.) in diisopropylamine (5.3 ml, 38 mmol, 9.5 equiv.) was added bis(triphenylphosphine)palladium(II) dichloride (210 mg, 0.30 mmol, 0.7 mol %) and copper(I) iodide (195 mg, 1.03 mmol, 1.4 mol %). To the resultant mixture was added but-3- yn-2-ol (283 mg, 4 mmol, 1.0 equiv.) dropwise at 0 °C. The mixture was allowed to warm to room temperature and the reaction mixture stirred for 2 h and then concentrated. The mixture was purified by column chromatography (eluent: DCM) to yield product **13** as an orange oil (549 mg, 3.9 mmol, 98%).

R_f 0.45 (DCM); ν_{max}/cm^{-1} 3321 (OH), 2981 (C-H), 1598, 1489 (C-C Ar); ^{1}H NMR (300 MHz, $CDCl_3$) δ 7.31-7.50 (5H, m, Ar-H), 4.80 (1H, m, OCH), 2.04 (1H, br d, *J* = 4.9 Hz, OH), 1.59 (3H, d, *J* = 6.6 Hz, CH_3); ^{13}C NMR (75 MHz, $CDCl_3$) δ 131.7 (CH), 128.4 (CH), 128.3 (CH),

122.6 (C), 90.9 (C), 84.0 (C), 58.9 (CH), 24.4 (CH_3); $[\alpha]_D^{20°C}$ = +27.2 (c = 1.03 in $CHCl_3$) [lit. $[\alpha]_D^{25°C}$ = +37 (c = 0.8 in $CHCl_3$)].

1-(1,1,1-Trifluoro-7-phenylhept-4-yn-3-yl)pyrrolidine (14a)

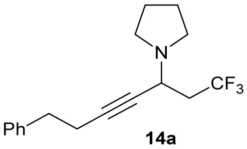

14a

Following a general procedure by Periasamy *et al.*[9] CuBr (226 mg, 20 mol%), pyrrolidine (0.74 ml, 8.9 mmol, 1.0 equiv.) and dry toluene (14 ml) were added to a flask. 3,3,3-trifluoropropanal (1.0 g, 8.9 mmol, 1.0 equiv.), 4 Å molecular sieves (4.5 g) and 4-phenyl-1-butyne (1.38 ml, 9.8 mmol, 1.1 equiv.) were added to the flask and stirred under Ar at rt for 36 hours. The molecular sieves were removed *via* filtration and washed with Et_2O. The crude product was purified by column chromatography (eluent 30:1 then 10:1 hexane/EtOAc) to yield product **14a** as a yellow oil (498 mg, 1.7 mmol, 19%).

R_f 0.68 (10:1 hexane/EtOAc); ν_{max}/cm^{-1} 2961 (C-H), 1496, 1455 (C-C Ar); ^{1}H NMR (300 MHz, $CDCl_3$) δ 7.09-7.26 (5H, m, Ar-H), 3.78 (1H, t, *J* = 5.8 Hz, NC<u>H</u>CH_2), 2.75 (2H, t, *J* = 7.4 Hz, ≡CH_2), 2.26-2.53 (8H, m, alkyl-H), 1.59-1.71 (4H, m, alkyl-H); ^{13}C NMR (75.5 MHz, $CDCl_3$) δ 140.6 (C), 128.5 (CH), 128.3 (CH), 126.2 (CH), 125.8 (q, *J* = 277.4 Hz, CF_3), 85.6 (C), 76.1 (C), 48.8 (CH_2), 48.0 (q, *J* = 3.6 Hz, C<u>H</u>CH_2CF_3), 39.7 (q, *J* = 27.3 Hz, <u>C</u>H_2CF_3), 35.1 (CH_2), 23.4 (CH_2), 20.6 (CH_2); ^{19}F NMR (282 MHz, $CDCl_3$) δ -64.07 (t, *J* = 10.4 Hz); Found (FTMS p NSI+) $[M + H]^+$ 296.1620, $C_{17}H_{21}F_3N$ requires 296.1621.

(S)-2-(Pyrrolidin-1-ylmethyl)-1-((S)-1,1,1-trifluoro-7-phenylhept-4-yn-3-yl)pyrrolidine (14b)

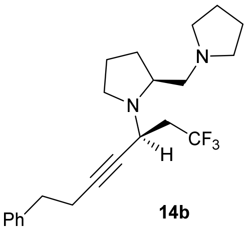

14b

Following a general procedure by Periasamy *et al.*[9] CuBr (187 mg, 20 mol%), (*S*)-(+)-1-(2-pyrrolidinylmethyl)pyrrolidine (1.0 g, 6.5 mmol, 1.0 equiv.) and dry toluene (13 ml) were added to a flask. 3,3,3-Trifluoropropanal (0.56 mL, 6.5 mmol, 1.0 equiv.), 4 Å molecular sieves (4.5 g) and 4-phenyl-1-butyne (1.00 ml, 7.1 mmol, 1.1 equiv.) were added to the flask and stirred under N_2 at rt for 42 hours. The molecular sieves were removed *via* filtration and washed with Et_2O. The crude product was purified by graduated column chromatography using basic alumina (eluent 50:1 to 10:1 hexane/EtOAc) to yield product **14b** as a yellow oil (55 mg, 0.17 mmol, 2.7%).

R_f 0.39 (25:1 hexane/EtOAc neutral alumina plate); ν_{max}/cm^{-1} 2960, 2787 (C-H), 1454, (C-C Ar); ^{1}H NMR (300 MHz, $CDCl_3$) δ 7.09-7.25 (5H, m, Ar-H), 4.20 (1H, t, *J* = 7.4 Hz, NC<u>H</u>CH$_2$), 2.74 (2H, t, *J* = 7.3 Hz, ≡CCH$_2$), 2.61-2.72 (2H, m, alkyl-H), 2.17-2.50 (11H, m, alkyl-H), 1.37-1.84 (8H, m, alkyl-H); ^{13}C NMR (100.6 MHz, $CDCl_3$) δ 140.6 (C), 128.4 (CH), 128.3 (CH), 126.2 (CH), 125.8 (q, *J* = 277.3 Hz, CF_3), 85.2 (C), 61.7 (C), 59.4 (CH), 54.8 (CH_2) 47.0 (CH_2), 46.9 (q, *J* = 4.1 Hz, C<u>H</u>CH$_2$CF$_3$), 39.8 (q, *J* = 27.2 Hz, <u>C</u>H$_2$CF$_3$), 35.2 (CH_2), 30.3 (CH_2), 23.5 (CH_2), 22.9 (CH_2), 20.5 (CH_2); ^{19}F NMR (282 MHz, $CDCl_3$) δ -64.09 (t, *J* = 10.7 Hz); Found (FTMS p NSI+) $[M + H]^+$ 379.2353 $C_{22}H_{30}F_3N_2$ requires 379.2356.

(*S*)-Penta-2,3-dien-1-yl benzoate (4b)[2]

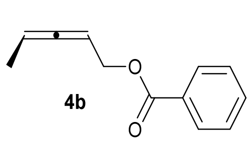

4b

To a solution of **12a** (1.02 g, 12.2 mmol, 1.0 equiv.) in DCM (50 ml) was added BzCl (3.0 ml, 24.6 mmol, 2.0 equiv.), pyridine (7.0 ml, 86.1 mmol, 7.0 equiv.) and 4-(dimethylamino)pyridine (306 mg, 2.5 mmol, 0.2 equiv.) at 0 ^{o}C. The solution was stirred for 6 hours. The reaction was quenched with 6N HCl and extracted with Et_2O. The combined organic layers were washed with brine, dried over $MgSO_4$ and concentrated. The mixture was purified by column chromatography (eluent: 7:1 hexane:Et_2O) to give product (*S*)-**4b** as a yellow oil (870.5 mg, 4.63 mmol, 38%, 99:1 er).

R_f 0.4 (3:1 Hexane/Et_2O); ν_{max}/cm^{-1} 2948 (C-H), 1970 (C=C=C), 1716 (C=O), 1601, 1584, 1491,1451 (C-C Ar); ^{1}H NMR (300 MHz, $CDCl_3$) δ 8.03-8.09 (2H, m, Ar-H), 7.53-7.59 (1H, m, Ar-H), 7.41-7.47 (2H, m, Ar-H), 5.30-5.40 (1H, m, allene H), 5.21-5.30 (1H, m, allene H),

4.80 (2H, dd, J = 6.7, 2.4 Hz, CHC<u>H</u>$_2$O), 1.69 (3H, dd, J = 7.0, 3.2 Hz, CHC<u>H</u>$_3$); ^{13}C NMR (75.5 MHz, $CDCl_3$) δ 206.4 (C), 166.5 (C), 133.0 (CH), 130.4 (C), 129.8 (CH), 128.4 (CH), 87.9 (CH), 86.5 (CH), 63.4 (CH_2), 14.0 (CH_3); $[\alpha]_D^{22°C}$ = +33.6 (c = 1.07 in $CHCl_3$); CSP-GC (β-Dex, 120 °C, 35 cm s^{-1}) (*R*)-**4b** 73.5 min and (*S*)-**4b** 74.4 min.

(*S*)-((Penta-2,3-dien-1-yloxy)methyl)benzene (4c)

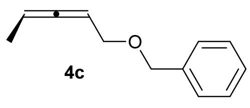

Compound **12a** (311 mg, 3.70 mmol, 1.0 equiv.) was added dropwise to a solution of NaH (60% in oil, 216 mg, 5.55 mmol, 1.5 equiv.) in THF (3.7 ml) at room temperature followed by addition of BnCl (0.64 ml, 5.55, 1.5 equiv.). The reaction was then stirred at 50 °C for 18 hours. The reaction was quenched with water and extracted with Et_2O. The organic layer was dried over $MgSO_4$ and concentrated. The mixture was purified by column chromatography (eluent: 50:1 then 25:1 hexane/Et_2O) to yield product (*S*)-**4c** as a yellow oil (313.2 mg, 1.8 mmol, 49%, 99:1 er).

R_f 0.37 (20:1 hexane/ Et_2O); ν_{max}/cm^{-1} 3029, 2924, 2855 (C-H), 1966 (C=C=C), 1495, 1453, 1410 (C-C Ar), 1093 (C-O-C); ^{1}H NMR (300 MHz, $CDCl_3$) δ 7.31-7.39 (5H, m, Ar-H), 5.13-5.28 (2H, m, allene-H), 4.54 (2H, s, OC<u>H</u>$_2$Ph), 4.05 (2H, dd, J = 6.4, 2.6 Hz, CHC<u>H</u>$_2$O), 1.69 (3H, dd, J = 6.7, 3.5 Hz, CH_3); ^{13}C NMR (75.5 MHz, $CDCl_3$) δ 206.0 (C), 138.4 (C), 128.5 (CH), 128.0 (CH), 127.7 (CH), 87.9 (CH), 86.7 (CH), 71.8 (CH_2), 68.6 (CH_2), 14.3 (CH_3); $[\alpha]_D^{21°C}$ = +31.9 (c = 1.06 in $CHCl_3$); CSP-GC (β-Dex, 110 °C, 35 cm s^{-1}) (*R*)-**4c** 65.5 min and (*S*)-**4c** 66.1 min.

(*S*)-Penta-2,3-dien-1-yl pivalate (4d)

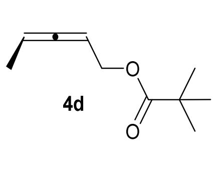

Compound **12a** (309 mg, 3.68 mmol, 1.0 equiv.) was added dropwise to a solution of NaH (60% in oil, 222 mg, 5.55 mmol, 1.5 equiv.) in THF (3.7 ml) at room temperature followed by addition of PivCl (0.68 ml, 5.55, 1.5 equiv.). The reaction was then stirred at 50 °C for 18

hours. The reaction was quenched with water and extracted with Et_2O. The organic layer was dried over $MgSO_4$ and concentrated. The mixture was purified by column chromatography (eluent: 25:1 hexane/Et_2O) to yield product **4d** as a yellow oil (303 mg, 1.8 mmol, 49%, 99:1 er (Er assumed by analogy to other allenes formed by esterification of allenol **12a**, as this allene could not be separated by CSP-GC or HPLC).

R_f 0.39 (25:1 hex/Et_2O); ν_{max}/cm^{-1} 2972 (C-H), 1971 (C=C=C), 1730 (C=O), 1032 (C-O-C); ^{1}H NMR (300 MHz, $CDCl_3$) δ 5.14-5.28 (2H, m, allene-H), 4.53 (2H, dd, *J* = 6.1, 3.0 Hz, C<u>H</u>$_2$O), 1.67 (3H, dd, *J* = 6.7, 3.5 Hz, CH_3), 1.20 (9H, s, ^{t}Bu); ^{13}C NMR (75.5 MHz, $CDCl_3$) δ 205.9 (C), 178.4 (C), 87.9 (CH), 86.8 (CH), 62.4 (CH_2), 38.9 (C), 27.3 (CH_3), 14.1 (CH_3); Found (GC/MS EI+) $[M]^+$ 168.1152, $C_{10}H_{16}O_2$ requires 168.1150. $[\alpha]_D^{21°C}$ = +21.4 (c = 1.12 in $CHCl_3$).

(*S*)-Penta-2,3-dien-1-yl acetate (4e)

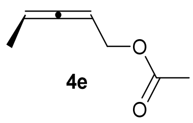

Compound **12a** (299 mg, 3.56 mmol, 1.0 equiv.) was added dropwise to a solution of NaH (60% in oil, 180 mg, 5.07 mmol, 1.5 equiv.) in THF (3.4 ml) at 0 °C followed by addition of AcCl (0.36 ml, 5.07, 1.5 equiv.). The reaction was then stirred at 35 °C for 18 hours. The reaction was quenched with water and extracted with Et_2O. The organic layer was dried over $MgSO_4$ and concentrated. The mixture was purified by column chromatography (eluent: 10:1 hexane/Et_2O) to yield product **4e** as a yellow oil (92.3 mg, 0.73 mmol, 21%, 99:1 er).

R_f 0.33 (10:1 hex/Et_2O); ν_{max}/cm^{-1} 2949 (C-H), 1970 (C=C=C), 1738 (C=O), 1022 (C-O-C); ^{1}H NMR (300 MHz, $CDCl_3$) δ 5.15-5.30 (2H, m, allene H), 4.52-4.57 (2H, m, OCH_2), 2.06 (3H, s, O=CCH_3), 1.65-1.71 (3H, m, CHC<u>H</u>$_3$); ^{13}C NMR (75.5 MHz, $CDCl_3$) δ 206.3 (C), 170.9 (C), 87.7 (CH), 86.4 (CH), 63.0 (CH_2), 21.1 (CH_3), 14.0 (CH_3); Found (GC/MS EI+) $[M]^+$ 126.0682, $C_7H_{10}O_2$ requires 126.0681; $[\alpha]_D^{21°C}$ = +45.5 (c = 1.10 in $CHCl_3$); CSP-GC (β-Dex, 100 °C, 35 cm s^{-1}) (*R*)-**4e** 5.7 min and (*S*)-**4e** 5.8 min.

(*S*)-Nona-2,3-dien-1-yl benzoate (4f)[2]

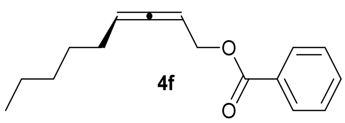

To a solution of **12b** (481 mg, 3.45 mmol, 1.0 equiv.) in DCM (8.0 ml) was added BzCl (0.46 ml, 3.94 mmol, 1.1 equiv.), pyridine (1.1 ml, 13.8 mmol, 4.0 equiv.) and 4-(dimethylamino)pyridine (47.6 mg, 0.38 mmol, 0.1 equiv.) at 0 °C. The solution was stirred for 6 hours. The reaction was quenched with 6N HCl and extracted with Et_2O. The combined organic layers were washed with brine and then dried over $MgSO_4$ and concentrated. The mixture was purified by column chromatography (eluent: 10:1 then 7:1 hexane:Et_2O) to give product (*S*)-**4f** as a yellow oil (436 mg, 1.79 mmol, 52%, 98.5::1.5 er).

This allene was prepared using propargylic alcohol (*R*)-1-Octyn-3-ol (98.5:1.5 e.r) in a similar procedure to that used to prepare allene **4b**. Since this procedure shows no erosion of ee, the e.r of allene **4f** can be assumed from the propargylic alcohol as this allene could not be separated *via* CSP-GC or HPLC.

R_f 0.6 (5:1 Hexane/Et_2O); ν_{max}/cm^{-1} 2927, 2856 (C-H), 1965 (C=C=C), 1718 (C=O), 1601, 1584,1451 (C-C Ar); ^{1}H NMR (300 MHz, $CDCl_3$) δ 8.03-8.09 (2H, m, Ar-H), 7.52-7.59 (1H, m, Ar-H), 7.40-7.48 (2H, m, Ar-H), 5.23-7.42 (2H, m, allene H), 4.80 (2H, dd, *J* = 6.6, 2.4 Hz, CHC$\underline{H}_2$O), 2.02 (2H, app. qd, *J* = 6.9, 3.0 Hz, =CHC$\underline{H}_2$$CH_2$), 1.38-1.46 (2H, m, alkyl H's), 1.26-1.33 (4H, m, alkyl H's), 0.87 (3H, t, *J* = 7.0 Hz, CH_3); ^{13}C NMR (75.5 MHz, $CDCl_3$) δ 205.6 (C), 166.5 (C), 133.0 (CH), 130.5 (C), 129.8 (CH), 128.4 (CH), 93.3 (CH), 87.1 (CH), 63.5 (CH_2), 31.4 (CH_2), 28.8 (CH_2), 28.5 (CH_2), 22.6 (CH_2), 14.2 (CH_3); $[\alpha]_D^{20°C}$ = +35.2 (c = 1.02 in $CHCl_3$).

(*R*)-Hexa-3,4-dien-1-yl benzoate (4g)[10]

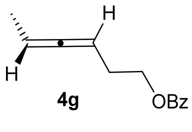

To a solution of benzoyl chloride (2.94 ml, 23 mmol, 3.0 equiv.) in DCM (15 ml), pyridine (6.19 ml, 77 mmol, 10 equiv.), and 4-(dimethylamino)pyridine (281 mg, 2.3 mmol, 0.3 equiv.) were added sequentially to a solution of (R)-hexa-3,4-dien-1-ol **12c** (745 mg, 7.7 mmol, 1 equiv.) in DCM (30 ml) at 0 °C. After stirring for 6 h at 0 °C the reaction was quenched by addition of 6 M HCl solution, the layers were separated, and the aqueous layer

was extracted with Et_2O (3 × 40 ml). The combined organic extracts were washed with brine, dried over $MgSO_4$ and concentrated. The mixture was purified by column chromatography (eluent: 15:1 hexane/Et_2O) to yield product **4g** as a thin colourless oil (1.41 g, 7.0 mmol, 91%, 99:1 e.r).

*R*f 0.40 (14:1 hexane/Et_2O); ν_{max}/cm^{-1} 2953 (C-H), 1967 (C=C=C), 1716 (C=O), 1602, 1584, 1451 (C-C Ar); ^{1}H NMR (300 MHz, $CDCl_3$) δ 8.03-8.11 (2H, m, Ar-H), 7.41-7.65 (3H, m, Ar-H), 5.03-5.25 (2H, m, allene H), 4.42 (2H, t, *J* = 6.7 Hz, OCH_2), 2.48 (2H, m, CH_2), 1.66 (3H, dd, *J* = 5.7, 4.5 Hz, $CH\underline{H}_3$); ^{13}C NMR (75 MHz, $CDCl_3$) δ 205.5 (C), 166.6 (C), 132.9 (CH), 130.4 (C), 129.6 (CH), 128.3 (CH), 86.5 (CH), 86.1 (CH), 64.1 (CH_2), 28.4 (CH_2), 14.4 (CH_3); Found (FTMS p NSI+) $[M + Na]^+$ 225.0884, $C_{13}H_{14}O_2Na$ requires 225.0886; $[\alpha]_D^{21°C}$ = -23.1 (c = 1.04 in $CHCl_3$); CSP-GC (β-Dex, 115 °C, 35 cm s^{-1}) (*R*)-**4g** 145.8 min and (*S*)-**4g** 148.2 min.

(*S*)-2-(Penta-2,3-dien-1-yl)isoindoline-1,3-dione (4h)

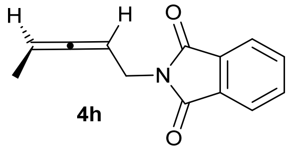

4h

Following a general procedure by Krause *et al.*[11] allenol **12a** (50.5 mg, 0.6 mmol, 1 equiv.), phthalimide (132 mg, 0.9 mmol, 1.5 equiv.) and PPh_3 (236 mg, 0.9 mmol, 1.5 equiv.) in THF (4.8 ml) were stirred at 0 °C. Diisopropyl azodicarboxylate (DIAD) (0.18 ml, 0.9 mmol, 1.5 equiv.) was added dropwise and the reaction mixture was stirred for a further 1 h at 0 °C and then concentrated. The mixture was purified by column chromatography (eluent: 12:1 hexane/EtOAc) to yield product **4h** as an oily white paste (63 mg, 0.29 mmol, 49%, 99:1 e.r).

*R*f 0.41 (9:1 hexane/EtOAc); ν_{max}/cm^{-1} 2927 (C-H), 1968 (C=C=C), 1710 (C=O), 1612, 1466 (C-C Ar); ^{1}H NMR (300 MHz, $CDCl_3$) δ 7.80-7.93 (2H, m, Ar-H), 7.66-7.79 (2H, m, Ar-H), 5.10-5.25 (2H, m, allene H), 4.28 (2H, app. t, *J* = 4.1 Hz, CH_2), 1.58 (3H, dd, *J* = 7.6, 3.0 Hz, CH_3); ^{13}C NMR (75 MHz, $CDCl_3$) δ 204.8 (C), 167.8 (C), 133.9 (CH), 132.2 (C), 123.2 (CH), 89.0 (CH), 86.1 (CH), 36.8 (CH_2), 13.9 (CH_3); Found (FTMS p NSI+) $[M + H]^+$ 214.0864, $C_{13}H_{12}NO_2$ requires 214.0863; $[\alpha]_D^{19°C}$ = +38.9 (c = 0.98 in $CHCl_3$); CSP-HPLC (Chiralcel OD-H, 98.2:1.8 hexane:IPA, 1 ml min^{-1}) (*R*)-**4h** 9.0 min and (*S*)-**4h** 9.6 min.

(*R*)-2-(Hexa-3,4-dien-1-yl)isoindoline-1,3-dione (4i)

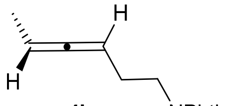

4i

Prepared in the same manner as **4h** from (*R*)-hexa-3,4-dien-1-ol **12c** (87.4 mg, 0.9 mmol) The mixture was purified by column chromatography (eluent: 12:1 hexane/EtOAc) to give product **4i** as an oily white paste (196 mg, 0.86 mmol, 96%, 99:1 e.r). E.r assumed from starting material allene **12c** (and the fact that the Mitsunobu reaction is shown not to erode the er in the related reaction **4h** above), as no separation conditions were found.

R_f 0.41 (9:1 hexane/EtOAc); ν_{max}/cm^{-1} 2947 (C-H), 1961 (C=C=C), 1707 (C=O); ^{1}H NMR (300 MHz, $CDCl_3$) δ 7.81-7.95 (2H, m, Ar-H), 7.67-7.80 (2H, m, Ar-H), 5.05 (2H, m, allene H), 3.80 (2H, t, J = 7.2 Hz, NCH_2), 2.39 (2H, dtd, J = 14.3, 7.2, 3.2 Hz, =CHC$\underline{H}_2$$CH_2$N), 1.53 (3H, dd, J = 6.6, 3.6 Hz, CH_3); ^{13}C NMR (75 MHz, $CDCl_3$) δ 205.6 (C), 168.3 (C), 133.9 (CH), 132.2 (C), 123.2 (CH), 86.5 (CH), 86.3 (CH), 37.5 (CH_2), 27.9 (CH_2), 14.3 (CH_3); Found (TOF MS) $[M+H]^+$ 228.1030, $C_{14}H_{14}NO_2$ requires 228.1024; $[\alpha]_D^{21°C}$ = -28.9 (c = 1.04 in $CHCl_3$).

(*R*)-Ethyl hexa-3,4-dienoate (4j)[7]

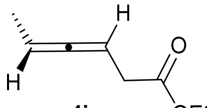

4j

(*R*)-But-3-yn-2-ol (1.23 g, 17.5 mmol, 1 equiv. 97% ee) and triethyl orthoacetate (10.9 ml, 59.5 mmol, 3.4 equiv.) were added to an oven dried, argon flushed round-bottom flask equipped with a Dean-Stark trap and heated to 170 °C (oil bath). Propionic acid (0.22 ml, 3.0 mmol, 0.17 equiv.) was added and the reaction mixture stirred at 170 °C. After 2.5 h, additional propionic acid (0.16 ml, 2.1 mmol, 0.21 equiv.) was added and the reaction mixture was stirred for a further 2.5 h under these conditions. After cooling to room temperature the reaction mixture was concentrated, passed through a silica plug with 10:1 hexane:EtOAc and concentrated again. The mixture was purified by column chromatography (eluent: 15:1 then 12:1 hexane/Et_2O) to yield product **4j** as a pale yellow oil (1.95 g, 13.8 mmol, 79%, 99:1 e.r)

R_f 0.61 (10:1 hexane/Et_2O); ν_{max}/cm^{-1} 2981 (C-H), 1967 (C=C=C), 1734 (C=O); ^{1}H NMR (300 MHz, $CDCl_3$) δ 5.09-5.31 (2H, m, allene H), 4.19 (2H, q, *J* = 7.1 Hz, OCH_2), 3.04 (2H, dd, *J* = 7.0, 2.9 Hz, =CHC$\underline{H}_2$), 1.69 (3H, dd, *J* = 6.3, 3.2 Hz, =CHC$\underline{H}_3$), 1.30 (3H, t, *J* = 7.1 Hz, $CH_2C\underline{H}_3$); ^{13}C NMR (75 MHz, $CDCl_3$) δ 205.8 (C), 171.7 (C), 86.8 (CH), 83.5 (CH), 60.7 (CH_2), 34.9 (CH_2), 14.2 (CH_3), 14.1 (CH_3); $[\alpha]_D^{21°C}$ = -27.0 (c = 1.11 in $CHCl_3$); CSP-GC (β-Dex, 90 °C, 35 cm s^{-1}) (*R*)-**4j** 11.6 min and (*S*)-**4j** 11.9 min.

(*R*)-N-Methoxy-N-methylhexa-3,4-dienamide (4k)

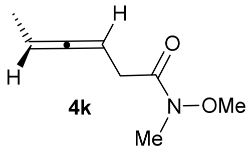

4k

Following a general procedure by Rathi *et al.*[12] 2M Isopropylmagnesium chloride in THF (5.25 ml, 10.5 mmol, 3 equiv.) was added dropwise over 30 minutes to a stirring solution of ester **4j** (491 mg, 3.5 mmol, 1 equiv.) and N,O-Dimethylhydroxylamine hydrochloride (527 mg, 5.4 mmol, 1.55 equiv.) in THF (7 ml) at – 20 °C. The reaction mixture was warmed slowly to – 10 °C and stirred for a further 30 minutes. 1 M HCl solution (35 ml) was added and the organic layer separated off. The aqueous layer was extracted with EtOAc (3 x 50 ml) and the combined organic layers dried over Na_2SO_4 and concentrated. The mixture was purified by column chromatography (eluent: 4:1 hexane/EtOAc) to yield product **4k** as an orange oil (286 mg, 1.9 mmol, 53%, 99:1 e.r).

R_f 0.36 (3:1 hexane/EtOAc); ν_{max}/cm^{-1} 2939 (C-H), 1968 (C=C=C), 1659 (C=O); ^{1}H NMR (300 MHz, $CDCl_3$) δ 5.22-5.39 (1H, m, allene H), 5.09-5.22 (1H, m, allene H), 3.72 (3H, s, OCH_3), 3.21 (3H, s, NCH_3), 3.14 – 3.20 (2H, m, CH_2), 1.68 (3H, dd, *J* = 7.0, 3.2 Hz, CH_3); ^{13}C NMR (75 MHz, $CDCl_3$) δ 205.8 (C), 172.4 (C), 86.4 (CH), 84.1 (CH), 61.3 (CH_3), 33.0 (CH_2), 32.3 (CH_3), 14.2 (CH_3); Found (FTMS p NSI+) $[M + H]^+$ 156.1016, $C_8H_{14}NO_2$ requires 156.1019; $[\alpha]_D^{21°C}$ = -30.8 (c = 1.04 in $CHCl_3$); CSP-GC (β-Dex, 100 °C, 35 cm s^{-1}) (*R*)-**4k** 39.8 min and (*S*)-**4k** 40.4 min.

(*S*)-Diethyl 2-(penta-2,3-dien-1-yl)malonate (4l)

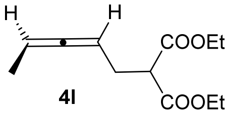

4l

Following a procedure by Krantz *et al.*[13] to a stirring solution of allenol **12a** (210 mg, 2.5 mmol, 1 equiv.) and triethylamine (0.54 ml, 3.9 mmol, 1.55 equiv.) in DCM (14 ml) at -25 °C mesyl chloride (0.23 ml, 3.0 mmol, 1.2 equiv.) was added dropwise over 20 minutes. The reaction mixture was warmed slowly to 0 °C and stirred for 2 ½ h. Chilled DCM (40 ml) was added and the reaction mixture washed with chilled water (30 ml), 2M HCl solution (2 x 35 ml) and a saturated solution of $NaHCO_3$ (30 ml) then dried over $MgSO_4$ and concentrated to give the crude mesylated allenol which was used without further purification.

Following a procedure by Dinh *et al.*[14] Diethyl malonate (0.50 ml, 3.1 mmol, 1.25 equiv.) was added to a stirring solution of NaH (60 % dispersion in oil) (125 mg, 3.1 mmol, 1.25 equiv.) in THF (30 ml) at room temperature room temperature. After 30 minutes, all of the crude mesylated allenol and tetrabutylammonium iodide (92 mg, 0.25 mmol, 0.1 equiv.) in THF (20 ml) was added and the reaction mixture was diluted with THF (10 ml) and stirred for 40 h at 50 °C. Diethyl ether (50 ml) was added and the solution washed with water (2 x 30 ml). The combined aqueous layers were extracted with the diethyl ether (40 ml) and the combined organic layers were dried over $MgSO_4$ and concentrated. The mixture was purified by column chromatography (eluent: 36:1 hexane/EtOAc) to yield product **4l** as a colourless oil (202 mg, 0.9 mmol, 36%, 99:1 e.r).

R_f 0.46 (15:1 pentane/Et_2O); ν_{max}/cm^{-1} 2983 (C-H), 1962 (C=C=C), 1730 (C=O); ^{1}H NMR (300 MHz, $CDCl_3$) δ 4.98-5.31 (2H, m, allene H), 4.13-4.30 (4H, m, OCH_2), 3.49 (1H, t, J = 7.1 Hz, C<u>H</u>CH_2), 2.53-2.65 (2H, m, CH_2), 1.65 (3H, dd, J = 9.6, 3.7 Hz, CHC<u>H</u>$_3$), 1.30 (6H, t, J = 7.1 Hz, CH_2C<u>H</u>$_3$); ^{13}C NMR (75 MHz, $CDCl_3$) δ 204.89 (C), 169.0 (C), 87.5 (CH), 87.0 (CH), 61.4 (CH_2), 51.6 (CH), 27.9 (CH_2), 14.3 (CH_3), 14.1 (CH_3); $[\alpha]_D^{21°C}$ = +34.1 (c = 0.94 in $CHCl_3$); CSP-HPLC (Chiralpak IA, 99.2:0.8 hexane:IPA, 1 ml min^{-1}) (*R*)-**4l** 6.9 min and (*S*)-**4l** 7.2 min.

(*S*)-Buta-1,2-dien-1-ylbenzene (4a)[15]

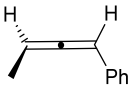

4a

Diethyl azodicarboxylate (DEAD) (0.70 ml, 4.4 mmol, 1.3 equiv.) was added to a solution of PPh_3 (1.15 g, 4.4 mmol, 1.3 equiv.) in THF (13 ml) at -15 °C. After 10 min, a solution of (R)-4-phenylbut-3-yn-2-ol **13** (497 mg, 3.4 mmol, 1 equiv.) in THF (10 ml) was added to the yellow reaction mixture, followed 10 min later by a solution of 2-nitrobenzenesulfonylhydrazide (NBSH) (1.04 g, 4.8 mmol, 1.4 equiv.) in THF (13 ml). The resulting suspension was held at -15 °C for 1 h, warmed to room temperature and left to stand overnight and then concentrated. The mixture was purified by column chromatography (eluent: hexane) to yield product **4a** as an orange oil (168 mg, 1.3 mmol, 38%, 99:1 e.r).

*R*f 0.74 (hexane); ν_{max}/cm^{-1} 3028, 2982, 2920 (C-H), 1946 (C=C=C), 1598, 1496, 1463 (C-C Ar); ^{1}H NMR (300 MHz, $CDCl_3$) δ 7.15-7.40 (5H, m, Ar-H), 6.13 (1H, dq, *J* = 6.4, 3.2 Hz, allene CH), 5.57 (1H, app. qn, *J* = 7.1 Hz, allene CH), 1.82 (3H, dd, *J* = 7.1, 3.2 Hz, CH_3); ^{13}C NMR (75 MHz, $CDCl_3$) δ 206.0 (C), 135.1 (C), 128.5 (CH), 126.6 (2xCH), 94.0 (CH), 89.6 (CH), 14.1 (CH_3); $[\alpha]_D^{21°C}$ = -32.7 (c = 0.98 in $CHCl_3$) [lit.[16] $[\alpha]_D^{20°C}$ = -238 (c = 1.33 in Acetone)]; CSP-GC (β-Dex, 93 °C, 35 cm s^{-1}) (*R*)-**4a** 20.4 min and (*S*)-**4a** 20.8 min.

(*S*)-(5-Cyclohexylpenta-3,4-dien-1-yl)benzene (4n)[17]

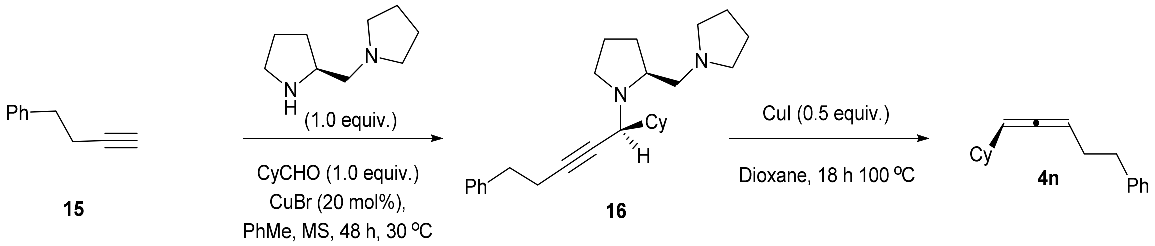

CuBr (58.0 mg, 20 mol%), (*S*)-(+)-1-(2-pyrrolidinylmethyl)pyrrolidine (0.33 ml, 2.0 mmol, 1.0 equiv.) and dry toluene (3.0 ml) were added to a flask. Distilled cyclohexanecarboxaldehyde (0.24 ml, 2.0 mmol, 1.0 equiv.), 4 Å molecular sieves (1 g) and 4-phenyl-1-butyne (0.31 ml, 2.2 mmol, 1.1 equiv.) were added to the flask and stirred under Ar at 30 °C for 48 hours. The molecular sieves were removed *via* filtration and washed with Et_2O. The crude product was purified by column chromatography using basic alumina (eluent 40:1 hexane/EtOAc) to yield product **16** as an impure yellow oil (271 mg, 0.71 mmol 36%). Compound **16** (271 mg, 0.72 mmol, 1.0 equiv.), freshly distilled dioxane (2.9 ml) and CuI (72.0 mg, 0.38 mmol, 0.5 equiv.) was added to a flask and refluxed for 18 hours. The crude product was purified by column chromatography (eluent: hexane) to yield product **4n** as a yellow oil (57.4 mg, 0.25 mmol 35%).

R_f 0.48 (hexane); ν_{max}/cm^{-1} 2920, 2848 (C-H), 1959 (C=C=C), 1495, 1447 (C-C Ar); ^{1}H NMR (300 MHz, $CDCl_3$) δ 7.15-7.25 (3H, m, Ar-H), 7.07-7.15 (2H, m, Ar-H), 5.04-5.13 (1H, m, Allene-H), 4.97-5.04 (1H, m, Allene-H), 2.59-2.70 (2H, m, alkyl-H), 2.17-2.29 (2H, m, alkyl-H), 1.76-1.91 (1H, m, alkyl-H), 1.49-1.68 (5H, m, alkyl-H), 1.07-1.26 (3H, m, alkyl-H), 0.87-1.07 (2H, m, alkyl-H); ^{13}C NMR (75.5 MHz, $CDCl_3$) δ 202.9 (C), 142.1 (C), 128.7 (CH), 128.4 (CH), 125.9 (CH), 97.7 (CH), 91.3 (CH), 37.4 (CH), 35.7 (CH_2), 33.23 (CH_2), 33.20 (CH_2), 31.0 (CH_2), 26.3 (CH_2), 26.21 (CH_2), 26.20 (CH_2).

(*S*)-1-((3-Penta-2,3-dien-1-yl)oxy)hexane (4o)

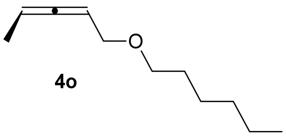

4o

Compound **12a** (290 mg, 3.46 mmol, 1.0 equiv.) was added dropwise to a solution of NaH (60% in oil, 193 mg, 5.19 mmol, 1.5 equiv.) in THF (3.5 ml) followed by addition of iodohexane (0.56 ml, 3.80, 1.1 equiv.). The reaction was then stirred at 50 °C for 18 hours. The reaction was quenched with water and extracted with Et_2O. The organic layer was dried over $MgSO_4$ and concentrated. The mixture was purified by column chromatography (eluent: 20: then 10:1 hexane/Et_2O) to yield product **4o** as a colourless oil (165 mg, 0.98 mmol, 28%, 99:1 er).

R_f 0.81 (3:1 hex/Et_2O); ν_{max}/cm^{-1} 2928, 2856 (C-H), 1967 (C=C=C), 1738 (C=O); ^{1}H NMR (300 MHz, $CDCl_3$) δ 5.10-5.20 (2H, m, allene H), 3.93-3.99 (2H, m, =CHC<u>H</u>$_2$OCH$_2$), 3.43 (2H, t, *J* = 7.7 Hz, CH$_2$OC<u>H</u>$_2$CH$_2$), 1.67 (3H, dd, *J* = 5.7, 4.4 Hz, CHC<u>H</u>$_3$), 1.51-1.62 (2H, m, alkyl H), 1.22-1.40 (6H, m, alkyl H), 0.88 (3H, t, *J* = 5.8 Hz, CH_3); ^{13}C NMR (75.5 MHz, $CDCl_3$) δ 205.7 (C), 88.1 (CH), 86.5 (CH), 70.2 (CH_2), 69.3 (CH_2), 31.9 (CH_2), 29.9 (CH_2), 26.0 (CH_2), 22.8 (CH_2), 14.27 (CH_3), 14.20 (CH_3); Found (TOF MS) $[M+H]^+$ 169.1591, $C_{11}H_{21}O$ requires 169.1592; $[\alpha]_D^{20°C}$ = +40.1 (c = 1.02 in $CHCl_3$); CSP-GC (β-Dex, 100 °C, 35 cm s^{-1}) (*R*)-**4o** 22.6 min and (*S*)-**4o** 23.0 min.

(7,7,7-Trifluoro-4l5-hepta-3,4-dien-1-yl)benzene (4p)[9]

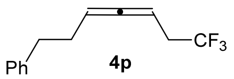

4p

Following a general procedure by Periasamy *et al.*[9] ZnI_2 (265 mg, 0.83 mmol, 0.5 equiv.) and NaI (136 mg, 0.91 mmol, 0.55 equiv.) were added to a flask and dried by heating under vacuum. The flask was flushed with argon and a solution of compound **14a** (488 mg, 1.7 mmol, 1 equiv.) in toluene (8 mL) was added. The solution was refluxed for 2 hours, allowed to cool to rt and passed through a silica plug with Et_2O. The crude product was purified by column chromatography (eluent: petrol 40-60°C) to yield product **4p** as a colourless oil (45 mg, 0.20 mmol, 12%). See below for characterisation.

(*R*)-(7,7,7-Trifluoro-4l5-hepta-3,4-dien-1-yl)benzene (4p)

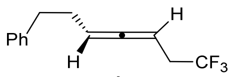

4p

Following a general procedure by Periasamy *et al.*[9] compound **14b** (93 mg, 0.3 mmol, 1.0 equiv.), dry dioxane (1.2 ml) and CuI (29 mg, 0.15 mmol, 0.5 equiv.) was added to a flask and refluxed for 20 hours. The crude was purified by column chromatography (eluent: petrol 40-60°C) to yield product **4p** as a yellow oil (7.0 mg, 0.03 mmol, 10%, 95:5 e.r).

R_f 0.45 (hexane); ν_{max}/cm^{-1} 3028, 2927, 2858 (C-H), 1969 (C=C=C), 1496, 1454 (C-C Ar); ^{1}H NMR (300 MHz, $CDCl_3$) δ 7.18-7.28 (2H, m, Ar-H), 7.05-7.17 (3H, m, Ar-H), 5.13-5.28 (1H, m, Allene-H), 4.89-5.05 (1H, m, Allene-H), 2.49-2.77 (4H, m, alkyl-H), 2.16-2.37 (2H, m, alkyl-H); ^{13}C NMR (75.5 MHz, $CDCl_3$) δ 207.0 (C), 141.4 (C), 128.5 (CH), 128.3 (CH), 126.0 (CH), 125.9 (q, *J* = 276.9 Hz, CF_3), 91.8 (CH), 80.9 (q, *J* = 4.3 Hz, C<u>H</u>CH_2CF_3), 35.0 (CH_2), 34.5 (q, *J* = 29.7 Hz, <u>C</u>H_2CF_3), 29.9 (CH_2); ^{19}F NMR (282 MHz, $CDCl_3$) δ -66.93 (t, *J* = 10.8 Hz); Found (TOF MS ASAP+) $[M + H]^+$ 227.1049, $C_{13}H_{14}F_3$ requires 227.1048; $[\alpha]_D^{21°C}$ = -12.0 (c = 0.5 in $CHCl_3$); CSP-GC (β-Dex, 135 °C, 35 cm s^{-1}) (*R*)-**4a** 12.5 min and (*S*)-**4a** 12.8 min.

Gold Catalysed Hydroalkoxylation Reactions

General procedure

Allene (0.14 mmol, 1.0 equiv.), alcohol nucleophile (1.4 mmol, 10.0 equiv.) and DMF (0.14 ml) were added to a vial and stirred at 0 °C. IPrAuCl (8.7 mg, 10 mol%) was added followed by AgOTf (3.6 mg, 10 mol%). The reaction was stirred at 0 °C for 24 h. The crude was then passed through two silica plugs and washed with Et_2O. The solution was washed with water and brine and the organic layer was dried over $MgSO_4$, then concentrated. The crude mixture was purified by column chromatography to yield products **6**.

(*R*,*E*)-4-(Benzyloxy)pent-2-en-1-yl benzoate (6bb)[2]

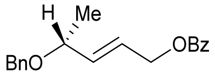

6bb

General procedure followed on a 0.28 mmol scale and the crude mixture was purified by column chromatography (eluent 20:1 hexane/EtOAc) to yield product **6bb** as a colourless oil (54.1 mg, 0.18 mmol, 65%, 98:2 e.r).

R_f 0.48 (5:1 hexane/EtOAc); ν_{max}/cm^{-1} 3031, 2973, 2864 (C-H), 1717 (C=O), 1601, 1584, 1494 (C-C Ar), 1268 (C-O); ^{1}H NMR (300 MHz, $CDCl_3$) δ 8.05-8.11 (2H, m, Ar-H), 7.54-7.61 (1H, m, Ar-H), 7.41-7.49 (2H, m, Ar-H), 7.26-7.37 (5H, m, Ar-H), 5.77-5.96 (2H, m, alkene-H), 4.85 (2H, d, *J* = 4.6 Hz, C<u>H</u>$_2$OBz), 4.58 (1H, d, *J* = 11.9 Hz, PhC<u>H</u>$_2$O), 4.43 (1H, d, *J* = 11.9 Hz, PhC<u>H</u>$_2$O), 4.05 (1H, app. q, *J* = 6.3 Hz, BnOC<u>H</u>CH_3), 1.32 (3H, d, *J* = 6.3 Hz, CH_3); ^{13}C NMR (75.5 MHz, $CDCl_3$) δ 166.4 (C), 138.7 (C), 136.5 (CH), 133.1 (CH), 130.3 (C), 129.8 (CH), 128.50 (CH), 128.49 (CH), 127.8 (CH), 127.6 (CH), 125.9 (CH), 75.1 (CH), 70.3 (CH_2), 64.8 (CH_2), 21.5 (CH_3); $[\alpha]_D^{21°C}$ = + 27.8 (c = 1.23 in $CHCl_3$); CSP-HPLC (Chiralcel OD-H, 99:1 hexane:IPA, 1 ml min^{-1}) (*R*)-**6bb** 11.5 min and (*S*)-**6bb** 15.2 min.

(*R*,*E*)-((Pent-2-ene-1,4-diylbis(oxy)bis(methylene))dibenzene (6cb)

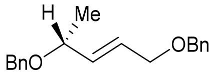

6cb

General procedure followed on a 0.30 mmol scale and the crude mixture was purified by column chromatography (eluent 25:1 hexane/EtOAc) to yield product **6cb** as a yellow oil (45%, 41 mg, 0.15 mmol, 95:5 e.r).

R_f 0.51 (5:1 hexane/EtOAc); ν_{max}/cm^{-1} 3063, 3029, 2973, 2855 (C-H), 1495, 1453, 1536 (C-C Ar), 1092 (C-O); ^{1}H NMR (300 MHz, $CDCl_3$) δ 7.23-7.40 (10H, m, Ar-H), 5.81 (1H, dt, *J* = 15.6, 5.1 Hz, CH=C<u>H</u>CH_2O), 5.67-5.77 (1H, m, C<u>H</u>=$CHCH_2$O), 4.58 (1H, d, *J* = 11.9 Hz, benzylic CH_2), 4.55 (2H, s, benzylic CH_2), 4.41 (1H, d, *J* = 11.9 Hz, benzylic CH_2), 4.07 (2H, d, *J* = 5.1 Hz, =CHC<u>H</u>$_2$O), 3.99 (1H, app. qn, *J* = 6.5 Hz, BnOC<u>H</u>CH_3), 1.31 (3H, d, *J* = 6.5 Hz, CH_3); ^{13}C NMR (75.5 MHz, $CDCl_3$) δ 138.9 (C), 138.4 (C), 135.2 (CH), 128.54 (CH), 128.47 (CH), 127.9 (CH), 127.8 (CH), 127.5 (CH), 75.3 (CH), 72.3 (CH_2), 70.3 (CH_2), 70.2

(CH_2), 21.6 (CH_3); Found (FTMS p NSI+) $[M + NH_4]^+$ 300.1957, $C_{19}H_{26}O_2N$ requires 300.1958. $[\alpha]_D^{21℃}$ = +35.8 (c = 0.95, $CHCl_3$); CSP-HPLC (ChiralPak IC, 99.3:0.7 hexane:IPA, 0.5 ml min^{-1}) (*S*)-**6cb** 12.8 min and (*R*)-**6cb** 14.9 min.

(*R*,*E*)-4-(Benzyloxy)pent-2-en-1-yl pivalate (6db)

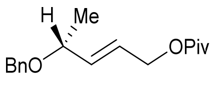

6db

General procedure followed and crude purified by column chromatography (eluent 20:1 hexane/EtOAc) to yield product **6db** as a yellow oil (27.1 mg, 0.10 mmol, 70%, 93:7 e.r). E.r was determined using ^1H NMR (400 MHz) analysis with chiral shift reagent (*R*)-(-)-1-(9-anthryl)-2,2,2-trifluroethanol.

R_f 0.24 (20:1 hexane/EtOAc); ν_{max}/cm^{-1} 3030, 2972, 2869 (C-H), 1727 (C=O), 1496, 1479, 1454 (C-C Ar), 1147 (C-O); ^1H NMR (300 MHz, $CDCl_3$) δ 7.31-7.38 (5H, m, Ar-H), 5.73-5.81 (1H, m, $CH=C\underline{H}CH_2O$), 5.70 (1H, dd, *J* = 16.5, 5.5 Hz, $C\underline{H}=CHCH_2O$), 4.60 (2H, d, *J* = 5.5 Hz, $=CHC\underline{H_2}O$), 4.56 (1H, d, *J* = 11.9 Hz, $PhC\underline{H_2}O$), 4.40 (1H, d, *J* = 11.9 Hz, $PhC\underline{H_2}O$), 3.97 (1H, app. qn, *J* = 6.4 Hz, $BnOC\underline{H}CH_3$), 1.29 (3H, d, *J* = 6.4 Hz, CH_3), 1.23 (9H, s, ^tBu); ^{13}C NMR (75.5 MHz, $CDCl_3$) δ 178.3 (C), 138.7 (C), 135.8 (CH), 128.5 (CH), 127.8 (CH), 127.6 (CH), 126.3 (CH), 75.0 (CH), 70.1 (CH_2), 64.1 (CH_2), 38.9 (C), 27.3 (CH_3), 21.5 (CH_3); Found (FTMS p NSI+) $[M + NH_4]^+$ 294.2062, $C_{17}H_{28}O_3N$ requires 294.2064. $[\alpha]_D^{21℃}$ = +16.5 (c = 0.97, $CHCl_3$).

(*R*,*E*)-4-Methoxypent-2-en-1-yl acetate (6ec)

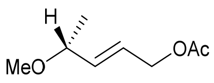

6ec

General procedure followed to yield product **6ec** as a colourless oil (16.9 mg, 0.11 mmol, 78%, 93:7 e.r). E.r was determined using ^1H NMR (400 MHz) analysis with chiral shift reagent (*R*)-(-)-1-(9-anthryl)-2,2,2-trifluroethanol.

ν_{max}/cm^{-1} 2974, 2932, 2820 (C-H), 1741 (C=O), 1447 (C-C Ar), 1097 (C-O); ^1H NMR (300 MHz, $CDCl_3$) δ 5.74 (1H, dt, *J* = 15.8, 5.7 Hz, $=C\underline{H}CH_2O$), 5.64 (1H, m, $CHC\underline{H}=CHCH_2$),

4.56 (2H, d, J = 5.7 Hz, =CHC$\underline{H}_2$O), 3.75 (1H, app. qn, J = 6.6 Hz, MeOC$\underline{H}$CH=CH), 3.24 (3H, s, OMe), 2.07 (3H, s, O=CCH$_3$), 1.23 (3H, d, J = 6.4 Hz, CH$_3$); ^{13}C NMR (75.5 MHz, CDCl$_3$) δ 170.9 (C), 136.2 (CH), 125.8 (CH), 77.4 (CH), 64.4 (CH$_2$), 56.2 (CH$_3$), 21.10 (CH$_3$), 21.09 (CH$_3$); Found (FTMS p CI) [M + NH$_4$]$^+$ 176.1, $C_8H_{18}O_3N$ requires 176.1; $[\alpha]_D^{21°C}$ = +18.4 (c = 1.09, CHCl$_3$).

(*R*,*E*)-4-Methoxyoct-2-en-1-yl benzoate (6fc)

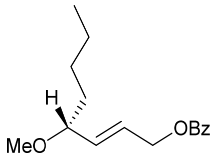

6fc

General procedure followed to yield product **6fc** as a colourless oil (34.6 mg, 0.13 mmol, 91%, 97:3 e.r).

ν_{max}/cm^{-1} 2930, 2859 (C-H), 1719 (C=O), 1602, 1451 (C-C Ar), 1097 (C-O); ^{1}H NMR (300 MHz, CDCl$_3$) δ 8.04-8.09 (2H, m, Ar-H), 7.56 (1H, tt, J = 6.5, 1.4 Hz, Ar-H), 7.41-7.48 (2H, m, Ar-H), 5.86 (1H, dtd, J = 15.6, 5.8, 0.5 Hz, =C$\underline{H}$CH$_2$O), 5.69 (1H, ddt, J = 15.6, 7.4, 1.1 Hz, MeOCHC$\underline{H}$=CH), 4.83 (2H, dd, J = 5.8, 1.1 Hz, =CHC$\underline{H}_2$O), 3.57 (1H, app. q, J = 6.8 Hz, MeOC$\underline{H}$CH=CH), 3.28 (3H, s, OMe), 1.53-1.67 (1H, m, alkyl-H), 1.21-1.43 (7H, m, alkyl-H), 0.88 (3H, t, J = 6.9 Hz, CH$_3$); ^{13}C NMR (75.5 MHz, CDCl$_3$) δ 166.4 (C), 135.3 (CH), 133.1 (CH), 130.3 (C), 129.8 (CH), 128.5 (CH), 126.7 (CH), 81.9 (CH), 64.8 (CH$_2$), 56.5 (CH$_3$), 35.4 (CH$_2$), 31.9 (CH$_2$), 25.1 (CH$_2$), 22.7 (CH$_2$), 14.2 (CH$_3$); Found (FTMS p NSI+) [M + NH$_4$]$^+$ 294.2064, $C_{17}H_{28}O_3N$ requires 294.2064; $[\alpha]_D^{20°C}$ = +7.3 (c = 1.09, CHCl$_3$); CSP-HPLC (ChiralPak IC, 99.3:0.7 hexane:IPA, 1 ml min^{-1}) (*R*)-**6fc** 9.5 min and (*S*)-**6fc** 9.7 min.

(*E*)-5-(Benzyloxy)hex-3-en-1-yl benzoate (6gb)

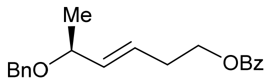

6gb

General procedure followed and the crude mixture was purified by column chromatography (eluent 7:1 hexane/Et_2O) to yield product **6gb** as a colourless oil (35 mg, 0.11 mmol, 79%, 90:10 e.r).

*R*f 0.17 (8:1 hexane:Et_2O); ν_{max}/cm^{-1} 2972, 2861 (C-H), 1716 (C=O), 1602, 1584, 1494, 1452 (C-C Ar); ^{1}H NMR (300 MHz, $CDCl_3$) δ 8.03-8.11 (2H, m, Ar-H), 7.53-7.62 (1H, m, Ar-H), 7.18-7.52 (2H, m, Ar-H), 7.23-7.39 (5H, m, Ar-H), 5.72 (1H, dt, *J* = 16.0, 6.5 Hz, CH_2HC=), 5.60 (1H, dd, *J* = 16.0, 8.0 Hz, OCHHC=), 4.56 (1H, d, *J* = 12.0 Hz, $PhCH_2O$), 4.43 (2H, t, *J* = 6.5, $BzOCH_2$), 4.38 (1H, d, *J* = 12.0 Hz, $PhCH_2O$), 3.87-4.02 (1H, m, OCH), 2.59 (2H, q, *J* = 6.5 Hz, CH_2), 1.30 (3H, d, *J* = 6.4 Hz, $CHCH_3$); ^{13}C NMR (75 MHz, $CDCl_3$) δ 166.53 (C), 138.8 (C), 135.1 (CH), 132.9 (CH), 130.3 (C), 129.6 (CH), 128.4 (CH), 128.3 (CH), 128.0 (CH), 127.6 (CH), 127.4 (CH), 75.6 (CH), 69.8 (CH_2), 64.0 (CH_2), 31.8 (CH_2), 21.7 (CH_3); Found (FTMS p NSI+) $[M + NH_4]^+$ 328.1910, $C_{20}H_{26}O_3N$ requires 328.1907; $[\alpha]_D^{20°C}$ = -19.1 (c = 0.84 in $CHCl_3$); CSP-HPLC (Chiralpak IA, 99.5:0.5 hexane:IPA, 1 ml min^{-1}) (*R*)-**6gb** 10.9 min and (*S*)-**6gb** 11.4 min.

(*R*,*E*)-2-(4-(Benzyloxy)pent-2-en-1-yl)isoindoline-1,3-dione (6hb)

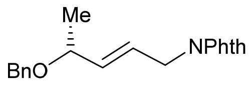

6hb

General procedure followed and the crude mixture was purified by column chromatography (eluent 9:1 hexane/EtOAc) to yield product **6hb** as a colourless oil (23 mg, 0.13 mmol, 50%, 97:3 e.r).

*R*f 0.41 (6:1 hexane/EtOAc); ν_{max}/cm^{-1} 2973, 2863 (C-H), 1709 (C=O), 1614, 1467, 1453 (C-C Ar); ^{1}H NMR (300 MHz, $CDCl_3$) δ 7.83-7.96 (2H, m, Phth-H), 7.68-7.82 (2H, m, Phth-H), 7.20-7.44 (5H, m, Ar-H), 5.62-5.83 (2H, m, alkene H), 4.55 (1H, d, *J* = 11.9 Hz, $PhOCH_2$), 4.38 (1H, d, *J* = 11.9 Hz, $PhOCH_2$), 4.30-4.36 (2H, m, NCH_2), 3.96 (1H, app. qn, *J* = 6.3 Hz, OCH), 1.29 (3H, d, *J* = 6.4 Hz, CH_3); ^{13}C NMR (75 MHz, $CDCl_3$) δ 167.9 (C), 138.6 (C), 135.7 (CH), 134.0 (CH), 132.1 (C), 128.3 (CH), 127.7 (CH), 127.4 (CH), 125.2 (CH), 123.3 (CH), 74.9 (CH), 70.1 (CH_2), 39.0 (CH_2), 21.4 (CH_3); Found (FTMS p NSI+) $[M + NH_4]^+$ 346.2743, $C_{22}H_{36}O_2N$ requires 346.2741; $[\alpha]_D^{21°C}$ = -24.0 (c = 1.00 in $CHCl_3$); CSP-HPLC (Chiralpak IC, 98.3:1.7 hexane:IPA, 1 ml min^{-1}) (*R*)-**6hb** 20.5 min and (*S*)-**6hb** 22.1 min.

(*S*,*E*)-2-(5-(Benzyloxy)hex-3-en-1-yl)isoindoline-1,3-dione (6ib)

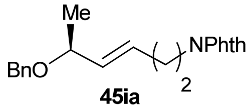

45ia

General procedure followed and the crude mixture was purified by column chromatography (eluent 9:1 hexane/EtOAc) to yield product **6ib** as a colourless oil (44 mg, 0.13 mmol, 94%, 81:19 e.r).

*R*f 0.25 (9:1 hexane/EtOAc); ν_{max}/cm^{-1} 2928 (C-H), 1708 (C=O), 1615, 1465, 1495, 1467 (C-C Ar); ^{1}H NMR (300 MHz, $CDCl_3$) δ 7.75-7.99 (2H, m, Phth-H), 7.55-7.75 (2H, m, Phth-H), 7.15-7.42 (5H, m, Ar-H), 5.64 (1H, dt, *J* = 15.4, 7.0 Hz, CH_2HC=), 5.45 (1H, dd, *J* = 15.4, 7.7 Hz, OCHHC=), 4.41 (1H, d, *J* = 11.9 Hz, PhOCHH), 4.26 (1H, d, *J* = 11.9 Hz, PhOCHH), 3.68-3.97 (3H, m, OCH + NCH_2), 2.42-2.60 (2H, m, CH_2), 1.18 (3H, d, *J* = 6.4 Hz, CH_3); ^{13}C NMR (75 MHz, $CDCl_3$) δ 168.3 (C), 138.8 (C), 135.4 (CH), 133.9 (CH), 132.0 (C), 128.4 (CH), 128.3 (CH), 127.6 (CH), 127.3 (CH), 123.2 (CH), 75.4 (CH), 69.7 (CH_2), 37.4 (CH_2), 31.4 (CH_2), 21.5 (CH_3); $[\alpha]_D^{21°C}$ = -21.9 (c = 1.28 in $CHCl_3$); Found (FTMS p NSI+) $[M + NH_4]^+$ 353.1857, $C_{21}H_{25}N_2O_3$ requires 353.1860; CSP-HPLC (Chiralpak IC, 98.3:1.7 hexane:IPA, 1 ml min^{-1}) (*R*)-**6ib** 14.7 min and (*S*)-**6ib** 16.7 min.

(*S*,*E*)-Ethyl 5-methoxyhex-3-enoate (6jc)

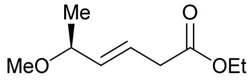

6jc

General procedure followed and the crude mixture was purified by column chromatography (eluent 10:1 hexane/EtOAc) to yield product **6jc** as a colourless oil (23 mg, 0.13 mmol, 92%, 97:3 e.r).

*R*f 0.22 (10:1 hexane/EtOAc); ν_{max}/cm^{-1} 2978, 2932, 2820 (C-H), 1735 (C=O); ^{1}H NMR (300 MHz, $CDCl_3$) δ 5.77 (1H, dtd, *J* = 15.5, 6.9, 0.6 Hz, =CHCH_2), 5.49 (1H, ddt, *J* = 15.5, 7.6, 1.4 Hz, =CHCHO), 4.18 (2H, q, *J* = 7.1 Hz, OCH_2), 3.75 (1H, app. qn, *J* = 6.6 Hz, CHO), 3.29 (3H, s, OCH_3), 3.11 (2H, d, *J* = 6.9 Hz, CH_2), 1.29 (3H, t, *J* = 7.1 Hz, CH_2CH_3), 1.29 (3H, d, *J* = 6.4 Hz, $OCHCH_3$); ^{13}C NMR (75 MHz, $CDCl_3$) δ 171.6 (C), 135.7 (CH), 124.3 (CH), 77.5 (CH), 60.7 (CH_2), 55.9 (CH_3), 37.7 (CH_2), 21.1 (CH_3), 14.2 (CH_3); Found (FTMS

p NSI+) [M + NH_4]$^+$ 190.1436, $C_9H_{20}O_3N$ 190.1436; $[\alpha]_D^{20°C}$ = -26.0 (c = 1.00 in $CHCl_3$); CSP-HPLC (Chiralpak IC, 99.3:0.7 hexane:IPA, 1 ml min^{-1}) (*R*)-**6jc** 13.0 min and (*S*)-**6jc** 15.0 min.

(*S,E*)-Ethyl 5-((4-methoxybenzyl)oxy)hex-3-enoate (6jd)

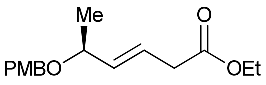

6jd

General procedure followed and the crude mixture was purified by column chromatography (eluent 9:1 hexane/EtOAc) to yield product **6jd** as a colourless oil (44 mg, 0.13 mmol, 94%, 95:5 e.r).

*R*f 0.31 (9:1 hexane/EtOAc); ν_{max}/cm^{-1} 2976 (C-H), 1732 (C=O), 1578, 1512, 1464 (C-C Ar); ^{1}H NMR (300 MHz, $CDCl_3$) δ 7.23-7.36 (2H, m, Ar-H), 6.84-6.96 (2H, m, Ar-H), 5.78 (1H, dtd, *J* = 15.4, 6.9, 0.6 Hz, =CHCH$_2$), 5.56 (1H, ddt, *J* = 15.4, 7.1, 1.3 Hz, =CHCH), 4.52 (1H, d, *J* = 11.5 Hz, ArOCHH), 4.35 (1H, d, *J* = 11.5 Hz, ArOCHH), 4.19 (2H, q, *J* = 7.1 Hz, CH$_2$CH$_3$), 3.95 (1H, app. qn, *J* = 7.1 Hz, OCH), 3.83 (3H, s, OCH$_3$), 3.13 (2H, app. dt, *J* = 6.9, 1.3 Hz, =CHCH$_2$), 1.30 (3H, t, *J* = 7.1 Hz, CH$_2$CH$_3$), 1.29 (3H, d, *J* = 6.4 Hz, CHCH$_3$); ^{13}C NMR (75 MHz, $CDCl_3$) δ 171.6 (C), 159.1 (C), 136.0 (CH), 130.8 (C), 129.3 (CH), 124.3 (CH), 113.8 (CH), 74.9 (CH), 69.5 (CH$_2$), 60.7 (CH$_2$), 55.3 (CH$_3$), 37.7 (CH$_2$), 21.5 (CH$_3$), 14.2 (CH$_3$); $[\alpha]_D^{18°C}$ = -34.4 (c = 0.99 in $CHCl_3$); CSP-HPLC (Chiralpak IC, 95:5 hexane:IPA, 1 ml min^{-1}) (*R*)-**6jd** 9.7 min and (*S*)-**6jd** 10.4 min.

(*S,E*)-5-(Benzyloxy)-*N*-methoxy-*N*-methylhex-3-enamide (6kb)

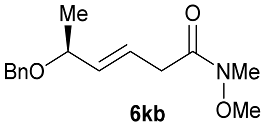

6kb

General procedure followed and the crude mixture was purified by column chromatography (eluent 4:1 then 3:1 hexane/EtOAc) to yield product **6kb** as a colourless oil (22 mg, 0.08 mmol, 58%, 91:9 e.r).

ν_{max}/cm^{-1} 2971, 2932, 2865 (C-H), 1660 (C=O), 1495, 1453 (C-C Ar); ^{1}H NMR (300 MHz, $CDCl_3$) δ 7.13-7.51 (5H, m, Ar-H), 5.85 (1H, dt, *J* = 15.5, 6.8 Hz, =C<u>H</u>CH_2), 5.57 (1H, ddt, *J* = 15.5, 7.7, 1.4 Hz, =C<u>H</u>CHO), 4.59 (1H, d, *J* = 11.9 Hz, PhOC<u>H</u>H), 4.42 (1H, d, *J* = 11.9 Hz, PhOCH<u>H</u>), 3.98 (1H, m, OCH), 3.73 (3H, s, OCH_3), 3.28 (2H, d, *J* = 6.8 Hz, =CHC<u>H</u>$_2$), 3.23 (3H, s, NCH_3), 1.32 (3H, d, *J* = 6.4 Hz, OCHC<u>H</u>$_3$); ^{13}C NMR (75 MHz, $CDCl_3$) δ 172.4 (C), 138.8 (C), 135.5 (CH), 128.3 (CH), 127.7 (CH), 127.4 (CH), 125.3 (CH), 75.4 (CH), 69.8 (CH_2), 61.3 (CH_3), 35.6 (CH_2), 32.3 (CH_3), 21.5 (CH_3); Found (FTMS p NSI+) [M + NH_4]$^+$ 264.1592, $C_{15}H_{22}O_3N$ requires 264.1594; $[\alpha]_D^{20°C}$ = -34.7 (c = 1.21 in $CHCl_3$); CSP-HPLC (Chiralpak IC, 95:5 hexane:IPA, 1 ml min^{-1}) (*R*)-**6kb** 25.7 min and (*S*)-**6kb** 30.9 min.

(*R*,*E*)-Diethyl 2-(4-(benzyloxy)pent-2-en-1-yl)malonate (6lb)

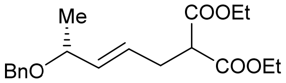

6lb

General procedure followed and the crude mixture was purified by column chromatography (eluent 12:1 hexane/EtOAc) to yield product **6lb** as a colourless oil (40 mg, 0.12 mmol, 86%, 90:10 e.r).

R_F 0.24 (10:1 hexane:EtOAc); ν_{max}/cm^{-1} 2980, 2931 (C-H), 1730 (C=O), 1496, 1454 (C-C Ar); ^{1}H NMR (300 MHz, $CDCl_3$) δ 7.19-7.53 (5H, m, Ar-H), 5.64 (1H, dt, *J* = 15.4, 6.4 Hz, =C<u>H</u>CH_2), 5.54 (1H, dd, *J* = 15.4, 7.2 Hz, =C<u>H</u>CH), 4.55 (1H, d, *J* = 11.9 Hz, PhOC<u>H</u>H), 4.36 (1H, d, *J* = 11.9 Hz, PhOCH<u>H</u>), 4.22 (4H, m, OC<u>H</u>$_2$$CH_3$), 3.90 (1H, m, OCH), 3.46 (1H, t, *J* = 7.5 Hz, C<u>H</u>CH_2), 2.69 (2H, app. t, *J* = 6.9 Hz, =CHC<u>H</u>$_2$), 1.23-1.35 (9H, m, CH_2CH_3 + $CHCH_3$); ^{13}C NMR (75 MHz, $CDCl_3$) δ 168.9 (C), 138.8 (C), 135.2 (CH), 128.3 (CH), 128.0 (CH), 127.7 (CH), 127.4 (CH), 75.3 (CH), 69.7 (CH_2), 61.4 (CH_2) , 52.0 (CH), 31.4 (CH_2), 21.6 (CH_3), 14.1 (CH_3); Found (FTMS p NSI+) [M + NH_4]$^+$ 352.2122, $C_{19}H_{30}O_5N$ requires 352.2118; $[\alpha]_D^{21°C}$ = +29.1 (c = 1.17 in $CHCl_3$); CSP-HPLC (Chiralpak IC, 98:2 hexane:IPA, 1 ml min^{-1}) (*R*)-**6lb** 13.1 min and (*S*)-**6lb** 14.3 min.

(*E*)-(3-(Benzyloxy)but-1-en-1-yl)benzene (6ab) + (E)-(1-(benzyloxy)but-2-en-1-yl)benzene (6ab')

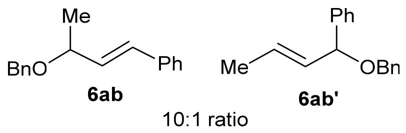

General procedure followed and the crude mixture was purified by column chromatography (eluent 20:1 hexane/EtOAc) to yield products **6ab** and **6ab'** in a 10:1 ratio as a colourless oil (26.2 mg, 0.11 mmol, 79% combined yield, racemic).

R_f 0.34 (20:1 hexane/EtOAc); ν_{max}/cm^{-1} 3028, 2972, 2859 (C-H), 1494, 1452 (C-C Ar); ^{1}H NMR (300 MHz, $CDCl_3$) δ 7.09-7.61 (10H + 10H', m, Ar-H, major + minor), 6.59 (1H, d, *J* = 16.0 Hz, =C<u>H</u>Ph, major), 6.21 (1H, dd, *J* = 16.0, 7.7 Hz, =C<u>H</u>CH, major), 5.49-5.89 (2H' m, =CH, minor), 4.83 (1H', d, *J* = 6.3 Hz, OCH, minor), 4.66 (1H, d, *J* = 11.9 Hz, $PhOCH_2$), 4.48 (1H, d, *J* = 11.9 Hz, $PhOCH_2$), 4.54 (2H', d, *J* = 0.9 Hz, OCH_2, minor), 4.16 (1H, m, OCH, major), 1.76 (3H', d, *J* = 4.9 Hz, CH_3, minor), 1.43 (3H, d, *J* = 6.4 Hz, CH_3, major); ^{13}C NMR (75 MHz, $CDCl_3$) Major only δ 138.8 (C), 136.7 (C), 131.7 (CH), 131.4 (CH), 128.6 (CH), 128.41 (CH), 128.38 (CH), 127.7 (CH), 127.4 (CH), 126.5 (CH), 75.9 (CH), 70.1 (CH_2), 21.8 (CH_3); CSP-HPLC (Chiralpak IB, hexane 1 ml min^{-1}) (*R*)-**6ab** 14.9 min and (*S*)-**6ab** 17.6 min.

(*E*)-(5-(Benzyloxy)-5-cyclohexylpent-3-en-1-yl) benzene (6nb)

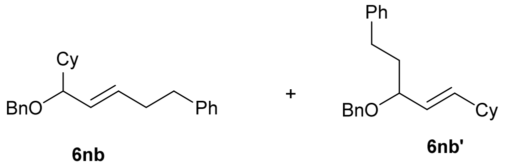

General procedure followed and the crude product was purified by column chromatography (eluent: 25:1 hexane/ether) to yield products **6nb** and **6nb'** as a colourless oil (32.6 mg, 0.10 mmol, 72%) in a 1:0.7 ratio.

R_f 0.43 (20:1 hexane/Et_2O); ν_{max}/cm^{-1} 3062, 3026, 2921, 2850 (C-H), 1603, 1495, 1451 (C-C Ar), 1093 (C-O); ^{1}H NMR (300 MHz, $CDCl_3$) δ 7.03-7.42 (10H + 10H', m, Ar-H, major + minor), 5.42-5.55 (1H + 1H', m, alkene H, major + minor), 5.24-5.29 (1H, m, alkene-H, major), 5.18-5.24 (1H', m, alkene-H, minor), 4.50 (1H', d, *J* = 11.9 Hz, PhC<u>H</u>$_2$O, minor), 4.40

(1H, d, *J* = 12.1 Hz. PhCH_2O, major), 4.25 (1H', d, *J* = 11.9 Hz, PhCH_2O, minor), 4.10 (1H, d, *J* = 12.1 Hz. PhCH_2O, major), 3.61 (1H', app. q, *J* = 6.23 Hz, OCHCH=CH, minor), 3.26, (1H, app t, *J* = 7.4 Hz, OCHCH=CH, major), 2.51-2.73 (2H + 2H', m, alkyl H, major + minor), 2.30-2.40 (2H, m, alkyl H, major), 1.80-2.00 (1H + 2H', m, alkyl H, major + minor), 1.49-1.78 (5H + 5H', m, alkyl H, major + minor), 1.27-1.42 (1H + 1H', m, alkyl H, major + minor), 0.94-1.26 (2H + 5H', alkyl H, major + minor), 0.7-0.9 (2H, m, alkyl H, major + minor); ^{13}C NMR (75.5 MHz, $CDCl_3$) δ 142.4 (C, minor), 141.8 (C, major), 140.7 (CH, minor), 139.4 (C, major), 139.2 (C, minor), 133.9 (CH, major), 130.5 (CH, major), 128.7 (CH, major), 128.6 (CH, minor), 128.5 (CH, minor), 128.44 (CH, major), 128.40 (CH, minor), 128.3 (CH, minor), 128.0 (CH, minor), 127.9 (CH, major), 127.8 (CH, major), 127.5 (CH, minor), 127.3 (CH, major), 126.0 (CH, major), 125.8 (CH, minor), 84.8 (CH, major), 79.7 (CH, minor), 69.79 (CH_2, major), 69.77 (CH_2, minor), 42.7 (CH, major), 40.6 (CH, minor), 37.6 (CH_2, minor), 35.9 (CH_2, major), 34.1 (CH_2, major), 33.2 (CH_2, major), 33.1 (CH_2, minor), 31.9 (CH_2, minor), 29.5 (CH_2, major + minor), 29.2 (CH_2, major + minor), 26.8 (CH_2, major), 26.3 (CH_2, major), 26.2 (CH_2, minor), 26.1 (CH_2, minor); Found (FTMS p NSI+) $[M + NH_4]^+$ 352.2636, $C_{24}H_{34}ON$ requires 352.2635.

(*R*,*E*)-1-((4-Methoxypent-2-en-1-yl)oxy)hexane (6oc)

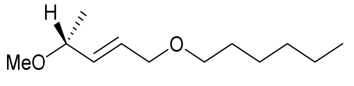

6oc

General procedure followed to yield product **6oc** as a colourless oil (26.8 mg, 0.13 mmol, 94%, 87:13 e.r).

ν_{max}/cm^{-1} 2956, 2929, 2855 (C-H), 1103 (C-O); ^{1}H NMR (300 MHz, $CDCl_3$) δ 5.72 (1H, dtd, *J* = 15.6, 5.7, 0.6 Hz, =CHCH$_2$O), 5.58 (1H, ddt, *J* = 15.6, 7.1, 1.1 Hz, CHCH=CHCH$_2$), 3.97 (2H, dd, *J* = 5.7, 1.1 Hz =CHCH_2O), 3.73 (1H, app. qn, *J* = 6.5 Hz, MeOCHCH=CH), 3.41 (2H, t, *J* = 6.7 Hz, CH$_2$OCH_2CH$_2$), 3.27 (3H, s, OMe), 1.52-1.63 (2H, m, alkyl H), 1.25-1.37 (6H, m, alkyl H), 1.23 (3H, d, *J* = 6.5 Hz, MeOCHCH_3), 0.88 (3H, t, *J* = 6.7 Hz, CH$_2$CH_3); ^{13}C NMR (75.5 MHz, $CDCl_3$) δ 134.4 (CH), 129.0 (CH), 77.6 (CH), 70.9 (CH_2), 70.6 (CH_2), 56.1 (CH_3), 31.8 (CH_2), 29.9 (CH_2), 26.0 (CH_2), 22.8 (CH_2), 21.3 (CH_3), 14.2 (CH_3); Found (FTMS p NSI) $[M + NH_4]^+$ 218.2114, $C_{12}H_{28}O_2N$ requires 218.2115; $[\alpha]_D^{21°C}$ = +22.5 (c =

1.07, $CHCl_3$) +22.5; CSP-GC (β-Dex, 80 °C, 35 cm s^{-1}) (*R*)-**6oc** 125.2 min and (*S*)-**6oc** 127.8 min.

(*S*,*E*)-(3-(Benzyloxy)-7,7,7-trifluorohept-4-en-1-yl)benzene (6pb)

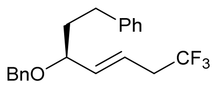

6pb

General procedure followed and crude purified by column chromatography (eluent 80:1 hexane/EtOAc) to yield impure product **6pb** as a colourless oil (40 mg, 0.12 mmol, 86%, 76% purity 95:5 e.r).

*R*f 0.47 (50:1 hexane/EtOAc); ν_{max}/cm^{-1} 3029, 2929, 2863 (C-H), 1496, 1454, 1429 (C-C Ar); ^{1}H NMR (400 MHz, $CDCl_3$) δ 7.05-7.29 (5H, m, Ar-H), 5.61 (1H, dd, *J* = 15.6, 7.3 Hz, =C<u>H</u>CH), 5.52 (1H, dt, *J* = 15.6, 6.6 Hz, =C<u>H</u>CH_2), 4.50 (1H, d, *J* = 11.8 Hz, PhOC<u>H</u>H), 4.26 (1H, d, *J* = 11.8 Hz, PhOCH<u>H</u>), 3.70 (1H, td, *J* = 7.3, 5.5 Hz, HCO) 2.72 – 2.87 (2H, m, CH_2CF_3), 2.52 – 2.72 (2H, m, CH_2Ph), 1.86 – 1.99 (1H, m, OCHC<u>H</u>$_2$), 1.67 – 1.79 (1H, m, OCHC<u>H</u>$_2$) Assigned using COSY 2D NMR; ^{13}C NMR (75.5 MHz, $CDCl_3$) δ 141.8 (C), 138.4 (CH), 128.44 (CH), 128.41 (CH), 128.36 (CH), 128.3 (q, *J* = 212.8 Hz, CF_3), 127.8 (CH), 127.6 (CH), 125.8 (CH), 121.0 (q, *J* = 3.7 Hz, C<u>H</u>CH_2CF_3), 78.4 (CH), 70.3 (CH_2), 37.08 (q, *J* = 29.9 Hz, <u>C</u>H_2CF_3), 37.0 (CH_2), 31.5 (CH_2); ^{19}F NMR (282 MHz, $CDCl_3$) δ -66.41 (t, *J* = 10.6 Hz); Found (FTMS p NSI+) $[M + NH_4]^+$ 352.1884, $C_{20}H_{25}F_3ON$ requires 352.1883; $[\alpha]_D^{22°C}$ = -10.0 (c = 0.28 in $CHCl_3$); CSP-HPLC (Chiralpak IA, hexane, 0.5 ml min^{-1}) (*R*)-**6pb** 22.0 min and (*S*)-**6pb** 24.7 min.

(*R*,*E*)-4-Methoxypent-2-en-1-yl benzoate (6bc)

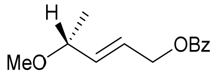

6bc

General procedure followed to yield product **6bc** as a colourless oil (25.2 mg, 0.11 mmol, 81%, >95:5 e.r). E.r was determined using ^{1}H NMR (400 MHz) analysis with chiral shift reagent (*R*)-(-)-1-(9-anthryl)-2,2,2-trifluroethanol.

ν_{max}/cm^{-1} 2974, 2930, 2820 (C-H), 1717 (C=O), 1601, 1584, 1451 (C-C Ar), 1097 (C-O); ^{1}H NMR (300 MHz, $CDCl_3$) δ 8.03-8.08 (2H, m, Ar-H), 7.56 (1H, tt, *J* = 7.0, 1.3 Hz, Ar-H), 7.40-7.48 (2H, m, Ar-H), 5.87 (1H, dtd, *J* = 15.6, 5.7, 0.7 Hz, CH=C<u>H</u>CH$_2$O), 5.74 (1H, ddt, *J* = 15.6, 6.7, 0.9 Hz, C<u>H</u>=CHCH$_2$O), 4.80-4.85 (2H, m, Ar-H), 3.78 (1H, app, qn, *J* = 6.7 Hz, MeOC<u>H</u>CH=CH), 3.29 (3H, s, OMe), 1.26 (3H, d, *J* = 6.7 Hz, CHC<u>H</u>$_3$); ^{13}C NMR (75.5 MHz, $CDCl_3$) δ 166.4 (C), 136.2 (CH), 133.1 (CH), 130.3 (C), 129.8 (CH), 128.5 (CH), 125.9 (CH), 77.3 (CH), 64.8 (CH_2), 56.2 (CH_3), 21.1 (CH_3); Found (FTMS p NSI+) [M + NH_4]$^+$ 238.1438, $C_{13}H_{20}O_3N$ requires 238.1438; $[\alpha]_D^{21°C}$ = +17.6 (c = 1.02, $CHCl_3$).

(*R*,*E*)-4-Butoxypent-2-en-1-yl benzoate (6be)

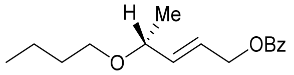

6be

General procedure followed and the crude mixture was purified by column chromatography (eluent 10:1 hexane/Ether) to yield product **6be** as a colourless oil (25.1 mg, 0.09 mmol, 68%, 97.5:2.5 e.r).

R_f 0.32 (10:1 hexane/Et_2O); ν_{max}/cm^{-1} 2958, 2931, 2869 (C-H), 1718 (C=O), 1601, 1584, 1451 (C-C Ar), 1094 (C-O); ^{1}H NMR (300 MHz, $CDCl_3$) δ 8.03-8.09 (2H, m, Ar-H), 7.56 (1H, tt, *J* = 6.5, 1.4 Hz, Ar-H), 7.41-7.48 (2H, m, Ar-H), 5.84 (1H, dt, *J* = 15.7, 5.4 Hz, CH=C<u>H</u>CH$_2$O), 5.78 (1H, dd, *J* = 15.7, 6.4 Hz, C<u>H</u>=CHCH$_2$O), 4.82 (2H, d, *J* = 5.4 Hz, CH=CHC<u>H</u>$_2$O), 3.87 (1H, app. qn, *J* = 6.4 Hz, OC<u>H</u>CH=CH), 3.44 (1H, dt, *J* = 9.2, 6.6 Hz, CH$_2$C<u>H</u>$_2$O), 3.33 (1H, dt, *J* = 9.2, 6.6 Hz, CH$_2$CH$_2$O), 1.49-1.60 (2H, m, ^{n}Bu-H), 1.32-1.44 (2H, m, ^{n}Bu-H), 1.25 (3H, d, *J* = 6.4 Hz, OCHC<u>H</u>$_3$), 0.91 (3H, t, *J* = 7.3 Hz, C<u>H</u>$_3$CH$_2$); ^{13}C NMR (75.5 MHz, $CDCl_3$) δ 166.4 (C), 137.0 (CH), 133.1 (CH), 130.3 (C), 129.8 (CH), 128.5 (CH), 125.1 (CH), 75.6 (CH), 68.4 (CH_2), 64.9 (CH_2), 32.2 (CH_2), 21.4 (CH_3), 19.5 (CH_2), 14.1 (CH_3); Found (FTMS p NSI+) [M + NH_4]$^+$ 280.1907, $C_{16}H_{26}O_3N$ requires 208.1907; $[\alpha]_D^{20°C}$ = +15.6 (c = 1.02, $CHCl_3$); CSP-HPLC (ChiralPak IA, 99:1 hexane:IPA, 1 ml min^{-1}) (*S*)-**6be** 8.7 min and (*R*)-**6be** 9.7 min.

(*R*,*E*)-4-Phenylethoxypent-2-en-1-yl benzoate (6bf)

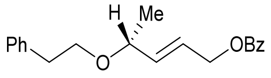

6bf

General procedure followed and the crude mixture was purified by column chromatography (eluent 7:1 hexane/Ether) to yield product **6bf** as a colourless oil (27.1 mg, 0.08 mmol, 62%, 98:2 e.r).

R_f 0.40 (5:1 hexane/Et_2O); ν_{max}/cm^{-1} 3027, 2930, 2863 (C-H), 1716 (C=O), 1601, 1584, 1451 (C-C Ar), 1094 (C-O); ^{1}H NMR (300 MHz, $CDCl_3$) δ 7.95-8.00 (2H, m, Ar-H), 7.49 (1H, tt, *J* = 6.5, 1.3 Hz, Ar-H), 7.33-7.40 (2H, m, Ar-H), 7.10-7.22 (5H, m, Ar-H), 5.75 (1H, dt, *J* = 15.6, 5.2 Hz, CH=C$\underline{H}$CH_2O), 5.66 (1H, dd, *J* = 15.6, 6.3 Hz, C$\underline{H}$=CHCH_2O), 4.72 (2H, d, *J* = 5.2 Hz, CH=CHC$\underline{H}_2$O), 3.82 (1H, app. qn, *J* = 6.3 Hz, OC$\underline{H}$CH=CH), 3.59 (1H, dt, *J* = 9.2, 7.5 Hz, PhCH_2C$\underline{H}_2$O), 3.48 (1H, dt, *J* = 9.2, 7.5 Hz, PhCH_2C$\underline{H}_2$O), 2.80 (2H, t, *J* = 7.5 Hz, PhC$\underline{H}_2$CH_2O), 1.18 (3H, d, *J* = 6.3 Hz, OCHC$\underline{H}_3$); ^{13}C NMR (75.5 MHz, $CDCl_3$) δ 166.4 (C), 139.1 (C), 136.7 (CH), 133.1 (CH), 130.3 (C), 129.8 (CH), 129.1 (CH), 128.5 (CH), 128.4 (CH), 126.3 (CH), 125.4 (CH), 75.9 (CH), 69.8 (CH_2), 64.8 (CH_2), 36.7 (CH_2), 21.3 (CH_3); Found (FTMS p NSI+) [M + NH_4]$^+$ 328.1907, $C_{20}H_{26}O_3N$ requires 328.1907; $[\alpha]_D^{20°C}$ = +23.2 (c = 0.95, $CHCl_3$); CSP-HPLC (ChiralPak IC, 99:1 hexane:IPA, 1 ml min^{-1}) (*S*)-**6bf** 15.6 min and (*R*)-**6bf** 18.0 min.

(*R*,*E*)-4-(3-Chloropropoxy)pent-2-en-1-yl benzoate (6bg)

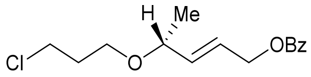

6bg

General procedure followed and the crude mixture was purified by column chromatography (eluent 10:1 hexane/Ether) to yield product **6bg** as a colourless oil (23.2 mg, 0.08 mmol, 60%, 97:3 e.r).

R_f 0.27 (10:1 hexane/Et_2O); ν_{max}/cm^{-1} 2971, 2868 (C-H), 1717 (C=O), 1601, 1451 (C-C Ar), 1097 (C-O); ^{1}H NMR (300 MHz, $CDCl_3$) δ 8.04-8.10 (2H, m, Ar-H), 7.57 (1H, tt, *J* = 6.5, 1.4 Hz, Ar-H), 7.40-7.49 (2H, m, Ar-H), 5.82-5.92 (1H, dtd, *J* = 15.6, 5.7, 0.5 Hz, CH=C$\underline{H}$CH_2O), 5.72-5.80 (1H, ddt, *J* = 15.6, 6.7, 0.9 Hz, C$\underline{H}$=CHCH_2O), 4.82 (2H, d, *J* =

5.7 Hz, CH=CHC<u>H</u>$_2$O), 3.90 (1H, app. qn, *J* = 6.6 Hz, OC<u>H</u>CH=CH), 3.64 (2H, t, *J* = 6.5 Hz, ClC<u>H</u>$_2$CH$_2$), 3.60 (1H, dt, *J* = 9.6, 5.9 Hz, CH$_2$C<u>H</u>$_2$O), 3.48 (1H, dt, *J* = 9.6, 5.9 Hz, CH$_2$C<u>H</u>$_2$O), 2.00 (2H, qn, *J* = 6.2 Hz, ClCH$_2$C<u>H</u>$_2$CH$_2$O), 1.25 (3H, d, *J* = 6.5 Hz, CH$_3$); ^{13}C NMR (75.5 MHz, CDCl$_3$) δ 166.4 (C), 136.5 (CH), 133.1 (CH), 130.3 (C), 129.8 (CH), 128.5 (CH), 125.5 (CH), 75.9 (CH), 64.8 (2 x CH$_2$), 42.2 (CH$_2$), 33.0 (CH$_2$), 21.3 (CH$_3$); Found (FTMS p NSI+) [M + NH$_4$]$^+$ 300.1362, $C_{15}H_{23}ClO_3N$ requires 300.1361; $[\alpha]_D^{19°C}$ = +16.3 (c = 0.98, CHCl$_3$); CSP-HPLC (ChiralPak IA, 98.8:1.2 hexane:IPA, 1 ml min^{-1}) (*S*)-**6bg** 8.1 min and (*R*)-**6bg** 11.2 min.

(*R*,*E*)-4-(4,4,4-Trifluorobutoxy)pent-2-en-1-yl benzoate (6bh)

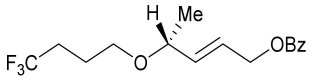

6bh

General procedure followed and the crude mixture was purified by column chromatography (eluent 10:1 hexane/Ether) to yield product **6bh** as a colourless oil (16.1 mg, 0.05 mmol, 37%, 99:1 e.r).

R$_f$ 0.40 (5:1 hexane/Et$_2$O); ν_{max}/cm^{-1} 2975, 2868 (C-H), 1719 (C=O), 1601, 1585, 1451 (C-C Ar), 1096 (C-O); ^{1}H NMR (300 MHz, CDCl$_3$) δ 8.03-8.09 (2H, m, Ar-H), 7.57 (1H, tt, *J* = 6.5, 1.4 Hz, Ar-H), 7.41-7.49 (2H, m, Ar-H), 5.84 (1H, dt, *J* = 15.6, 5.7 Hz, CH=C<u>H</u>CH$_2$O), 5.74 (1H, dd, *J* = 15.6, 6.7 Hz, C<u>H</u>=CHCH$_2$O), 4.82 (2H, d, *J* = 5.7 Hz, CH=CHC<u>H</u>$_2$O), 3.88 (1H, app. qn, *J* = 6.7 Hz, OC<u>H</u>CH=CH), 3.50 (1H, dt, *J* = 9.3, 6.2 Hz, CH$_2$C<u>H</u>$_2$O), 3.37 (1H, dt, *J* = 9.3, 6.2 Hz, CH$_2$C<u>H</u>$_2$O), 2.10-2.29 (2H, m, alkyl-H), 1.75-1.87 (2H, m, alkyl-H), 1.25 (3H, d, *J* = 6.7 Hz, CH$_3$); ^{13}C NMR (75.5 MHz, CDCl$_3$) δ 166.4 (C), 136.4 (CH), 133.1 (CH), 130.2 (C), 129.8 (CH), 129.2 (C, q, *J* = 279.8 Hz), 128.5 (CH), 125.7 (CH), 75.9 (CH), 66.6 (CH$_2$), 64.7 (CH$_2$), 31.1 (CH$_2$, q, *J* = 28.8 Hz), 22.7 (CH$_2$, q, *J* = 1.5 Hz), 21.3 (CH$_3$); Found (FTMS p NSI+) [M + NH$_4$]$^+$ 334.1624, $C_{16}H_{23}F_3O_3N$ requires 334.1625; $[\alpha]_D^{20°C}$ = +12.5 (c = 0.96, CHCl$_3$); CSP-HPLC (ChiralPak IA, hexane, 1 ml min^{-1}) (*S*)-**6bh** 21.1 min and (*R*)-**6bh** 26.3 min.

(*R*,*E*)-4-Isopropoxypent-2-en-1-yl benzoate (6ba)

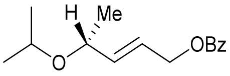

6ba

General procedure followed and the crude mixture was purified by column chromatography (eluent 10:1 hexane/Ether) to yield product **6ba** as a colourless oil (30.9 mg, 0.12 mmol, 88%, 97:3 e.r).

R_f 0.24 (10:1 hexane/Et_2O); ν_{max}/cm^{-1} 2971 (C-H), 1719 (C=O), 1601, 1585, 1451 (C-C Ar), 1096 (C-O); ^{1}H NMR (300 MHz, $CDCl_3$) δ 8.03-8.09 (2H, m, Ar-H), 7.56 (1H, tt, *J* = 6.5, 1.4 Hz, Ar-H), 7.41-7.47 (2H, m, Ar-H), 5.85 (1H, dt, *J* = 15.6, 5.2 Hz, CH=C<u>H</u>CH_2O), 5.78 (1H, dd, *J* = 15.6, 5.5 Hz, C<u>H</u>=CHCH_2O), 4.81 (2H, d, *J* = 5.2 Hz, CH=CHC<u>H</u>$_2$O), 4.00 (1H, app. qn, *J* = 6.4 Hz, OC<u>H</u>CH=CH), 3.65 (1H, sept, *J* = 6.1 Hz, CH_3C<u>H</u>CH_3), 1.23 (3H, d, *J* = 6.4 Hz, CH_3), 1.15 (3H, d, *J* = 6.1 Hz, CH_3C<u>H</u>CH_3), 1.12 (3H, d, *J* = 6.1 Hz, CH_3C<u>H</u>CH_3); ^{13}C NMR (75.5 MHz, $CDCl_3$) δ 166.4 (C), 137.6 (CH), 133.1 (CH), 130.3 (C), 129.8 (CH), 128.5 (CH), 124.5 (CH), 72.6 (CH), 68.7 (CH), 65.0 (CH_2), 23.3 (CH_3), 22.1 (CH_3), 21.9 (CH_3); Found (FTMS p NSI+) $[M + NH_4]^+$ 266.1753, $C_{15}H_{24}O_3N$ requires 266.1751; $[\alpha]_D^{20°C}$ = +10.0 (c = 1.00, $CHCl_3$); CSP-HPLC (ChiralPak IA, 99:1 hexane:IPA, 0.5 ml min^{-1}) (*S*)-**6ba** 13.3 min and (*R*)-**6ba** 15.2 min.

(*R*,*E*)-4-((*R*)-*sec*-Butoxy)pent-2-en-1-yl benzoate (6bj)

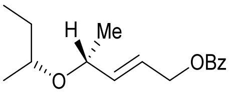

6bj

General procedure followed and the crude mixture was purified by column chromatography (eluent 10:1 hexane/Ether) to yield product **6bj** as a colourless oil (28.3 mg, 0.11 mmol, 78%, 81:19 e.r).

R_f 0.33 (10:1 hexane/Et_2O); ν_{max}/cm^{-1} 2969, 2929, 2875 (C-H), 1719 (C=O), 1601, 1451 (C-C Ar), 1175 (C-O); ^{1}H NMR (300 MHz, $CDCl_3$) δ 8.04-8.09 (2H, m, Ar-H), 7.56 (1H, tt, *J* = 6.5, 1.4 Hz, Ar-H), 7.41-7.47 (2H, m, Ar-H), 5.76-5.90 (2H, m, alkene-H), 4.87 (2H, d, *J* = 4.1 Hz, CH=CHC<u>H</u>$_2$O), 3.99 (1H, app. qn, *J* = 6.4 Hz, OC<u>H</u>CH=CH), 3.41 (1H, sex, *J* = 6.1 Hz, CH_3CH_2C<u>H</u>CH_3), 1.38-1.58 (2H, m, CH_3C<u>H</u>$_2$CHCH_3), 1.25 (3H, d, *J* = 6.4 Hz,

$OCHC\underline{H}_3$), 1.10 (3H, d, *J* = 7.2 Hz, $CH_3CH_2CHC\underline{H}_3$), 0.89 (3H, t, *J* = 7.4 Hz, $C\underline{H}_3CH_2CHCH_3$); ^{13}C NMR (75.5 MHz, $CDCl_3$) δ 166.4 (C), 137.9 (CH), 133.1 (CH), 130.4 (C), 129.8 (CH), 128.5 (CH), 124.3 (CH), 74.5 (CH), 73.4 (CH), 65.0 (CH_2), 29.2 (CH_2), 21.6 (CH_3), 20.6 (CH_3), 9.9 (CH_3); Found (FTMS p NSI+) $[M + Na]^+$ 285.1458, $C_{16}H_{22}O_3Na$ requires 285.1461; $[\alpha]_D^{22\,^{\circ}C}$ = +8.5 (c = 0.47, $CHCl_3$); CSP-HPLC (ChiralPak IC, 99:1 hexane:IPA, 1 ml min^{-1}) (*R*)-**6bj** 7.6 min and (*S*)-**6bj** 10.2 min.

(*R*,*E*)-4-((*R*)-1-Phenylethoxy)pent-2-en-1-yl benzoate (6bk)

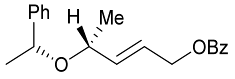

6bk

General procedure followed and the crude mixture was purified by column chromatography (eluent 10:1 hexane/Ether) to yield product **6bk** as a colourless oil (21.8 mg, 0.07 mmol, 51%, 97:3 e.r).

R_f 0.27 (10:1 hexane/Et_2O); ν_{max}/cm^{-1} 2973, 2928, 2875 (C-H), 1718 (C=O), 1584, 1492, 1450 (C-C Ar), 1175 (C-O); ^1H NMR (300 MHz, $CDCl_3$) δ 7.97-8.03 (2H, m, Ar-H), 7.50 (1H, tt, *J* = 6.5, 1.4 Hz, Ar-H), 7.35-7.43 (2H, m, Ar-H), 7.16-7.29 (5H, m, Ar-H), 5.60-5.75 (2H, m, alkene-H), 4.76 (2H, dd, *J* = 4.3, 1.1 Hz, $CH{=}CHC\underline{H}_2O$), 4.43 (1H, q, *J* = 6.5 Hz, $PhC\underline{H}CH_3$), 3.68 (1H, app. qn, *J* = 6.4 Hz, $OC\underline{H}CH{=}CH$), 1.33 (3H, d, *J* = 6.5 Hz, $PhCHC\underline{H}_3$), 1.13 (3H, d, *J* = 6.4 Hz, $OCHC\underline{H}_3$); ^{13}C NMR (75.5 MHz, $CDCl_3$) δ 166.4 (C), 144.2 (C), 136.5 (CH), 133.1 (CH), 130.3 (C), 129.8 (CH), 128.6 (CH), 128.5 (CH), 127.5 (CH), 126.4 (CH), 125.7 (CH),74.7 (CH), 72.7 (CH), 64.8 (CH_2), 24.8 (CH_3), 22.0 (CH_3); Found (FTMS p NSI+) $[M + NH_4]^+$ 328.1901, $C_{20}H_{26}O_3N$ requires 328.1907; $[\alpha]_D^{20\,^{\circ}C}$ = +125.6 (c = 0.43, $CHCl_3$); CSP-HPLC (Chiralcel OD-H, 99:1 hexane:IPA, 1 ml min^{-1}) (*R*)-**6bk** 35.4 min and (*S*)-**6bk** 59.0 min.

(*R*,*E*)-4,5,5-Trimethylhex-2-en-1-yl benzoate (6bl)

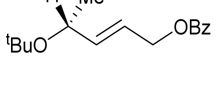

6bl

General procedure followed and the crude mixture was purified by column chromatography (eluent 10:1 hexane/Ether) to yield product **6bl** as a colourless oil (11.1 mg, 0.04 mmol, 30%, 94:6 e.r).

R_f 0.24 (10:1 hexane/Et_2O); ν_{max}/cm^{-1} 2973 (C-H), 1720 (C=O), 1601, 1451 (C-C Ar), 1175 (C-O); ^1H NMR (300 MHz, $CDCl_3$) δ 8.03-8.07 (2H, m, Ar-H), 7.56 (1H, tt, *J* = 6.5, 1.4 Hz, Ar-H), 7.40-7.47 (2H, m, Ar-H), 5.89-5.94 (1H, m, CH=C<u>H</u>CH_2O), 5.80 (1H, dd, *J* = 15.6, 0.9 Hz, C<u>H</u>=$CHCH_2O$), 4.80 (2H, d, *J* = 4.6 Hz, CH=CHC<u>H</u>$_2$O), 4.17 (1H, app. qn, *J* = 6.4 Hz, OC<u>H</u>CH=CH), 1.22 (3H, d, *J* = 6.4 Hz, OCHC<u>H</u>$_3$), 1.21 (9H, s, ^{t}Bu); ^{13}C NMR (75.5 MHz, $CDCl_3$) δ 166.5 (C), 139.9 (CH), 133.0 (CH), 130.5 (C), 129.8 (CH), 128.5 (CH), 122.6 (CH), 74.2 (C), 67.4 (CH), 65.3 (CH_2), 28.6 (CH_3), 23.5 (CH_3); Found (FTMS p NSI+) $[M + NH_4]^+$ 280.1902, $C_{16}H_{26}O_3N$ requires 280.1907; $[\alpha]_D^{21°C}$ = +3.6 (c = 1.11, $CHCl_3$); CSP-HPLC (ChiralPak IA, 99:1 hexane:IPA, 1 ml min^{-1}) (*S*)-**6bl** 6.8 min and (*R*)-**6bl** 9.7 min.

(*R*,*E*)-4-(3-Hydroxy-3-methylbutoxy)pent-2-en-1-yl benzoate (6bm)

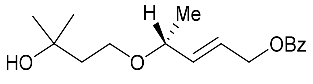

6bm

General procedure followed and the crude mixture was purified by column chromatography (eluent 1:1 hexane/Ether) to yield product **6bm** as a colourless oil (24.2 mg, 0.08 mmol, 60%, 98.2 e.r).

R_f 0.25 (1:1 hexane/Et_2O); ν_{max}/cm^{-1} 3465 (O-H), 2970 (C-H), 1717 (C=O), 1601, 1584, 1451 (C-C Ar), 1095 (C-O); ^1H NMR (300 MHz, $CDCl_3$) δ 8.03-8.09 (2H, m, Ar-H), 7.56 (1H, tt, *J* = 6.5, 1.3 Hz, Ar-H), 7.40-7.47 (2H, m, Ar-H), 5.82-5.91 (1H, m, CH=C<u>H</u>CH_2O), 5.75 (1H, ddt, *J* = 15.6, 7.1, 1.0 Hz, C<u>H</u>=$CHCH_2O$), 4.81 (2H, d, *J* = 5.6 Hz, CH=CHC<u>H</u>$_2$O), 3.89 (1H, app. qn, *J* = 6.5 Hz, OC<u>H</u>CH=CH), 3.72 (1H, dt, *J* = 9.5, 5.7 Hz, CH_2C<u>H</u>$_2$O), 3.56 (1H, dt, *J* = 9.5, 5.7 Hz, CH_2C<u>H</u>$_2$O), 3.36 (1H, br s, OH), 1.75 (2H, t, *J* = 5.7 Hz, HOCC<u>H</u>$_2$$CH_2O$), 1.27 (3H, d, *J* = 6.5 Hz, OCHC<u>H</u>$_3$), 1.23 (3H, s, HOCC<u>H</u>$_3$), 1.22 (3H, s, HOCC<u>H</u>$_3$); ^{13}C NMR (75.5 MHz, $CDCl_3$) δ 166.3 (C), 135.9 (CH), 133.1 (CH), 130.2 (C), 129.8 (CH), 128.5 (CH), 126.1 (CH), 76.5 (CH), 70.6 (C), 65.9 (CH_2), 64.6 (CH_2), 41.5 (CH_2), 29.44 (CH_3), 29.42 (CH_3), 21.4 (CH_3); Found (FTMS p NSI+) $[M + NH_4]^+$ 293.1749, $C_{17}H_{25}O_4N$ requires

293.1747; $[\alpha]_D^{20°C}$ = +20.6 (c = 0.58, $CHCl_3$); CSP-HPLC (ChiralPak IA, 97:3 hexane:IPA, 1 ml min^{-1}) (*R*)-**6bm** 14.4 min and (*S*)-**6bm** 16.0 min.

(*R*,*E*)-4-(Hex-5-en-1-yloxy)pent-2-en-1-yl benzoate (6bn)

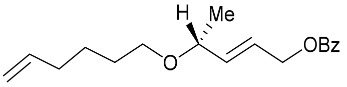

6bn

General procedure followed and the crude mixture was purified by column chromatography (eluent 7:1 hexane/Ether) to yield product **6bn** as a colourless oil (26.9 mg, 0.09 mmol, 66%, 99.8:0.2 e.r).

R_f 0.20 (10:1 hexane/Et_2O); ν_{max}/cm^{-1} 2974, 2932, 2859 (C-H), 1719 (C=O), 1601, 1584, 1451 (C-C Ar), 1096 (C-O); ^{1}H NMR (300 MHz, $CDCl_3$) δ 8.03-8.09 (2H, m, Ar-H), 7.56 (1H, tt, *J* = 6.5, 1.4 Hz, Ar-H), 7.40-7.49 (2H, m, Ar-H), 5.72-5.90 (3H, m, $CH_3CHC\underline{H}=C\underline{H}CH_2$ and $CH_2=C\underline{H}CH_2$), 4.90-5.04 (2H, m, $C\underline{H_2}=CHCH_2$), 4.82 (2H, d, *J* = 5.1 Hz, $CH=CHC\underline{H_2}O$), 3.87 (1H, app. qn, *J* = 6.4 Hz, $OC\underline{H}CH=CH$), 3.54 (1H, dt, *J* = 9.2, 6.7 Hz, $CH_2C\underline{H_2}O$), 3.33 (1H, dt, *J* = 9.2, 6.7 Hz, $CH_2C\underline{H_2}O$), 2.01-2.10 (2H, m, alkyl-H), 1.51-1.65 (2H, m, alkyl-H), 1.40-1.51 (2H, m, alkyl-H), 1.25 (3H, d, *J* = 6.4 Hz, $OCHC\underline{H_3}$); ^{13}C NMR (75.5 MHz, $CDCl_3$) δ 166.4 (C), 138.9 (CH), 137.0 (CH), 133.1 (CH), 130.3 (C), 129.8 (CH), 128.5 (CH), 125.2 (CH), 114.6 (CH_2), 75.6 (CH), 68.5 (CH_2), 64.9 (CH_2), 33.7 (CH_2), 29.5 (CH_2), 25.7 (CH_2), 21.4 (CH_3); Found (FTMS p NSI+) [M + NH_4]$^+$ 306.2065, $C_{18}H_{28}O_3N$ requires 306.2064; $[\alpha]_D^{21°C}$ = +16.1 (c = 0.99, $CHCl_3$); CSP-HPLC (ChiralPak IA, 97:3 hexane:IPA, 1 ml min^{-1}) (*S*)-**6bn** 24.8 min and (*R*)-**6bn** 34.0 min.

(*R*,*E*)-4-(Furan-2-ylmethoxy)pent-2-en-1-yl benzoate (6bo)

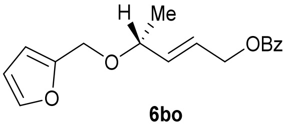

6bo

General procedure followed and the crude mixture was purified by column chromatography (eluent 5:1 hexane/Ether) to yield product **6bo** as a colourless oil (25.4 mg, 0.09 mmol, 64%, 99.1 e.r).

R_f 0.28 (5:1 hexane/Et_2O); ν_{max}/cm^{-1} 2974 (C-H), 1716 (C=O), 1601, 1584, 1451 (C-C Ar), 1069 (C-O); ^{1}H NMR (300 MHz, $CDCl_3$) δ 8.05-8.11 (2H, m, Ar-H), 7.57 (1H, tt, *J* = 6.5, 1.4 Hz, Ar-H), 7.42-7.48 (2H, m, Ar-H), 7.39 (1H, dd, *J* = 1.8, 0.9 Hz, furan-H), 6.32 (1H, dd, *J* = 3.2, 1.8 Hz, furan-H), 6.28 (1H, dd, *J* = 3.2, 0.9 Hz, furan-H), 5.90 (1H, dt, *J* = 16.1, 5.9 Hz, CH=C<u>H</u>CH_2O), 5.79 (1H, ddt, *J* = 16.1, 7.0, 1.1 Hz, C<u>H</u>=CHCH_2O), 4.84 (2H, dd, *J* = 5.9, 1.0 Hz, CH=CHC$\underline{H}_2$O), 4.50 (1H, d, *J* = 12.7 Hz, furan-C$\underline{H}_2$O), 4.37 (1H, d, *J* = 12.7 Hz, furan-CH_2O), 4.02 (1H, app. qn, *J* = 6.5 Hz, OC<u>H</u>CH=CH), 1.29 (3H, d, *J* = 6.5 Hz, OCHC$\underline{H}_3$); ^{13}C NMR (75.5 MHz, $CDCl_3$) δ 166.4 (C), 152.1 (C), 142.8 (CH), 136.0 (CH), 133.1 (CH), 130.3 (C), 129.8 (CH), 128.5 (CH), 126.2 (CH), 110.3 (CH), 109.2 (CH), 75.1 (CH), 64.7 (CH_2), 62.3 (CH_2), 21.4 (CH_3); Found (FTMS p NSI+) $[M + NH_4]^+$ 304.1543, $C_{17}H_{22}O_4N$ requires 304.1543; $[\alpha]_D^{22°C}$ = +41.4 (c = 1.06, $CHCl_3$); CSP-HPLC (ChiralPak IA, 98:2 hexane:IPA, 1 ml min^{-1}) (*R*)-**6bo** 6.56 min and (*S*)-**6bo** 8.29 min.

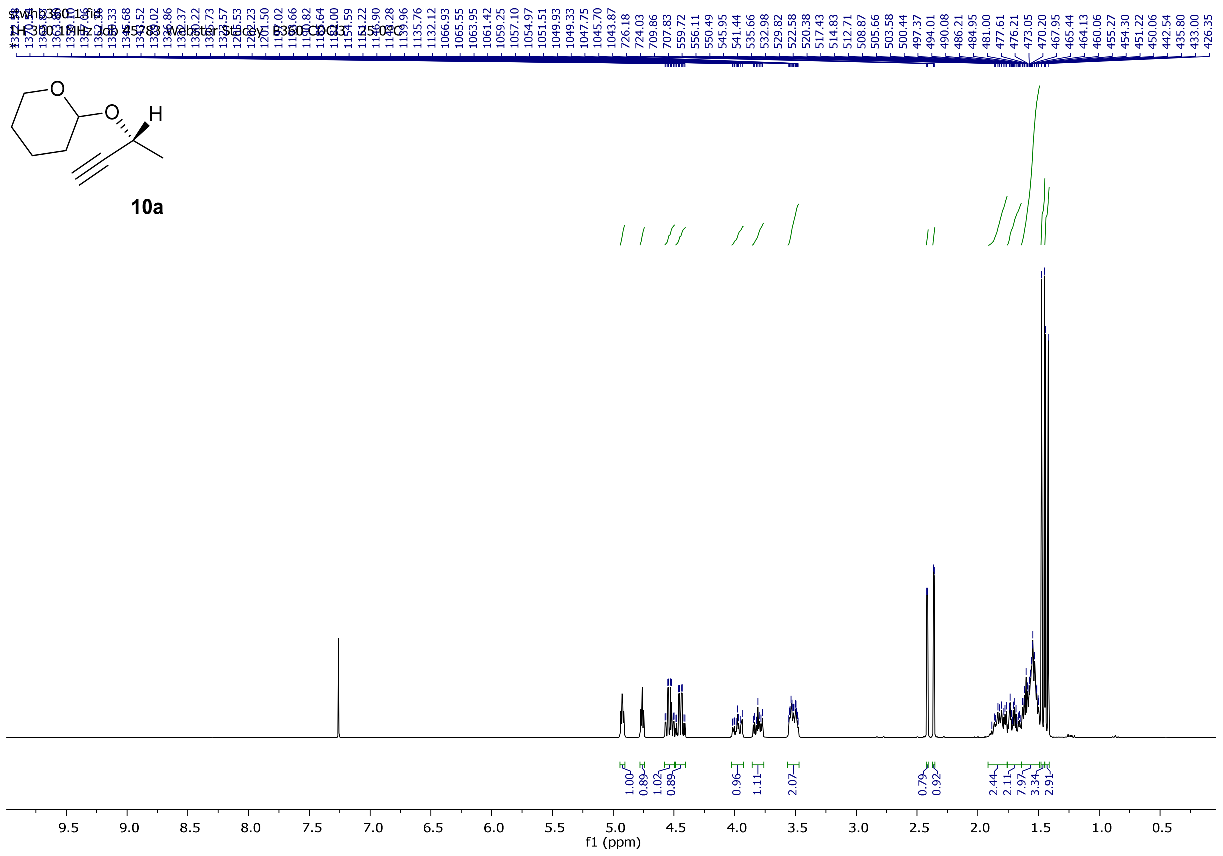

10a

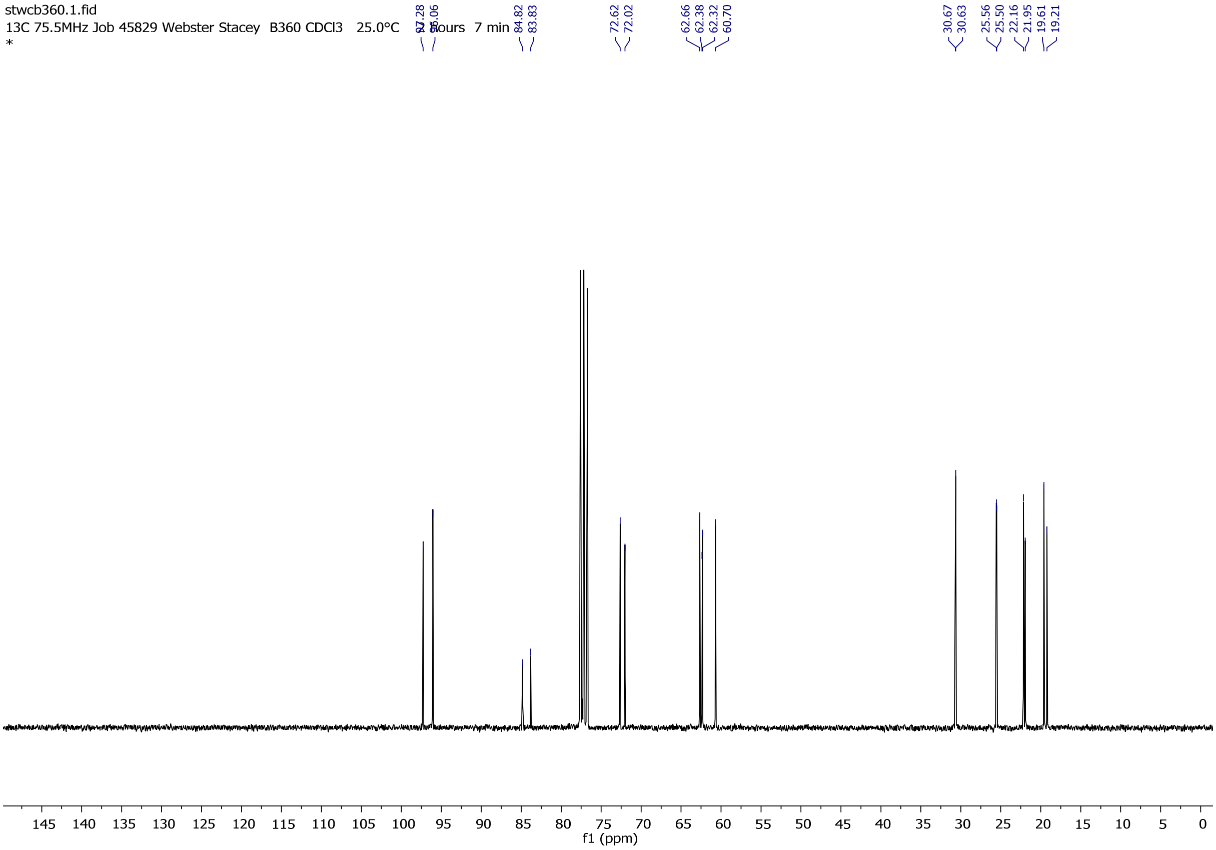
stwcb360.1.fid
13C 75.5MHz Job 45829 Webster Stacey B360 CDCl3 25.0°C 2 hours 7 min
*
97.28
96.06
84.82
83.83
72.62
72.02
62.66
62.38
62.32
60.70
30.67
30.63
25.56
25.50
22.16
21.95
19.61
19.21
f1 (ppm)

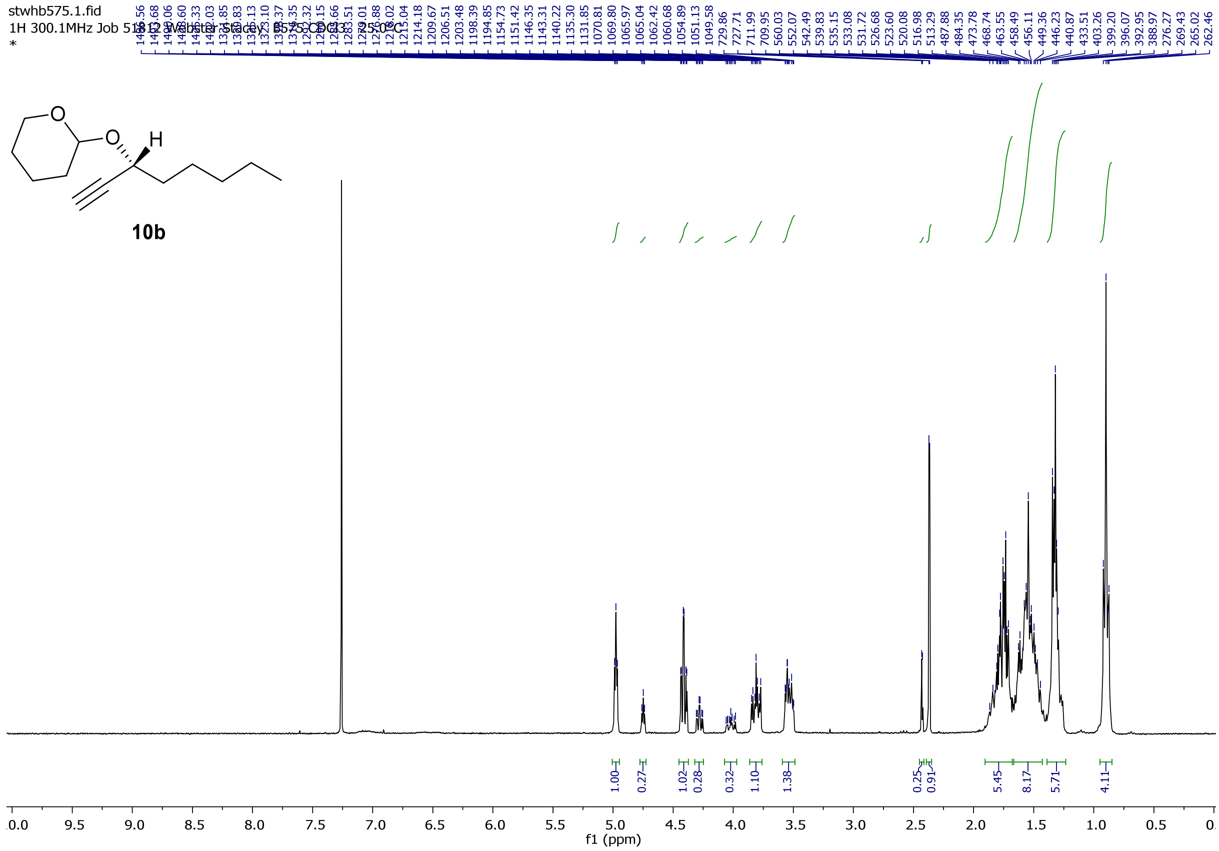
stwhb575.1.fid
1H 300.1MHz Job Webster Stacey B575 CDCl3 25.0°C
*
10b
1.00
0.27
1.02
0.28
0.32
1.10
1.38
0.25
0.91
5.45
8.17
5.71
4.11
f1 (ppm)

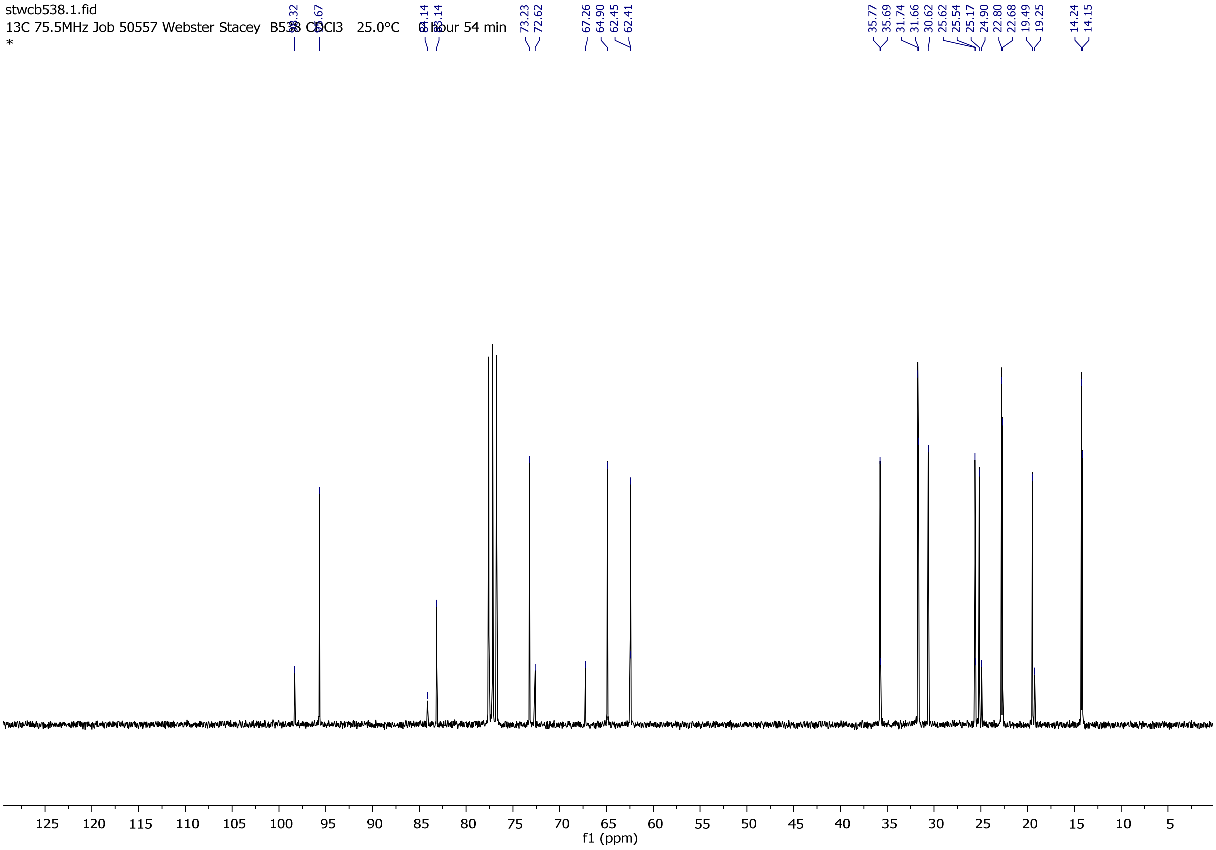
stwcb538.1.fid
13C 75.5MHz Job 50557 Webster Stacey B538 CDCl3 25.0°C
98.32
95.67
84.14
83.14
73.23
72.62
67.26
64.90
62.45
62.41
35.77
35.69
31.74
31.66
30.62
25.62
25.54
25.17
24.90
22.80
22.68
19.49
19.25
14.24
14.15
f1 (ppm)

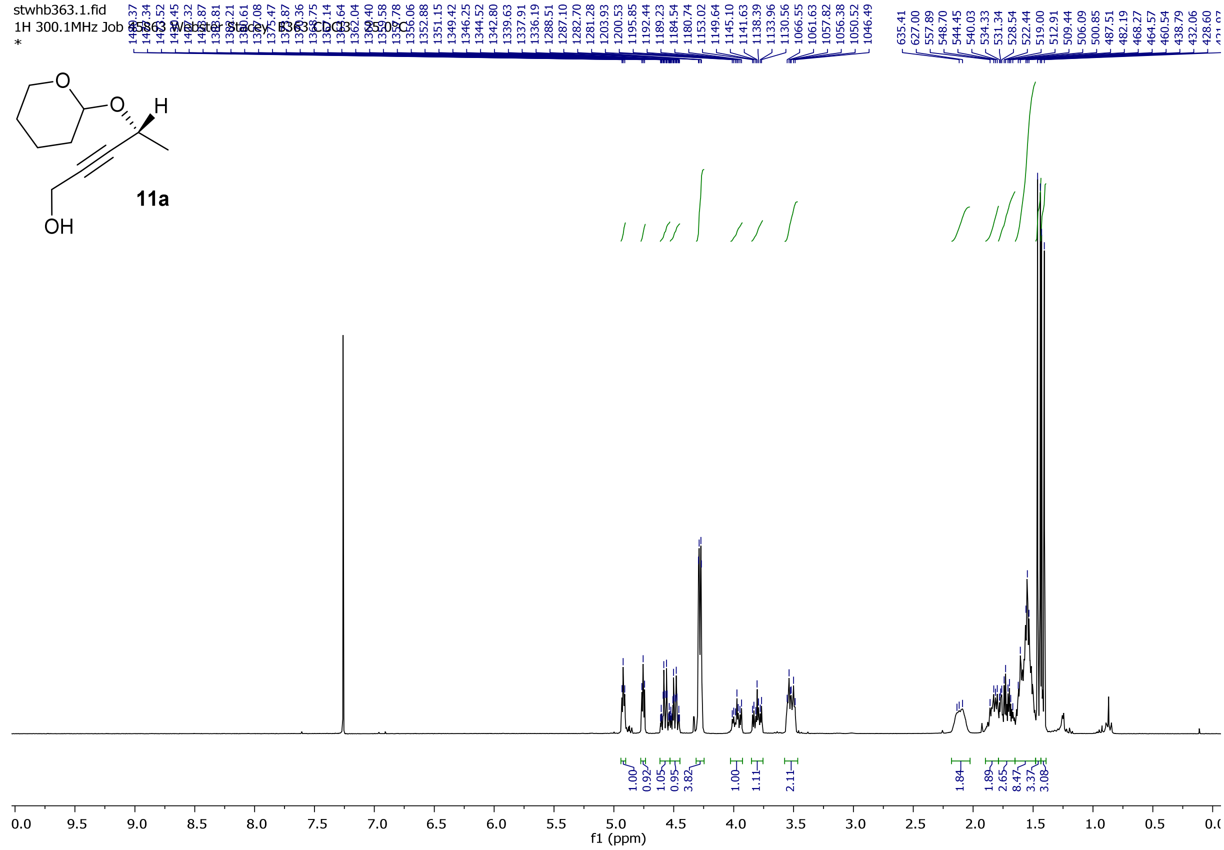
stwhb363.1.fid
1H 300.1MHz Job
1.00
0.92
1.05
0.95
3.82
1.00
1.11
2.11
1.84
1.89
2.65
8.47
3.37
3.08
f1 (ppm)

11a

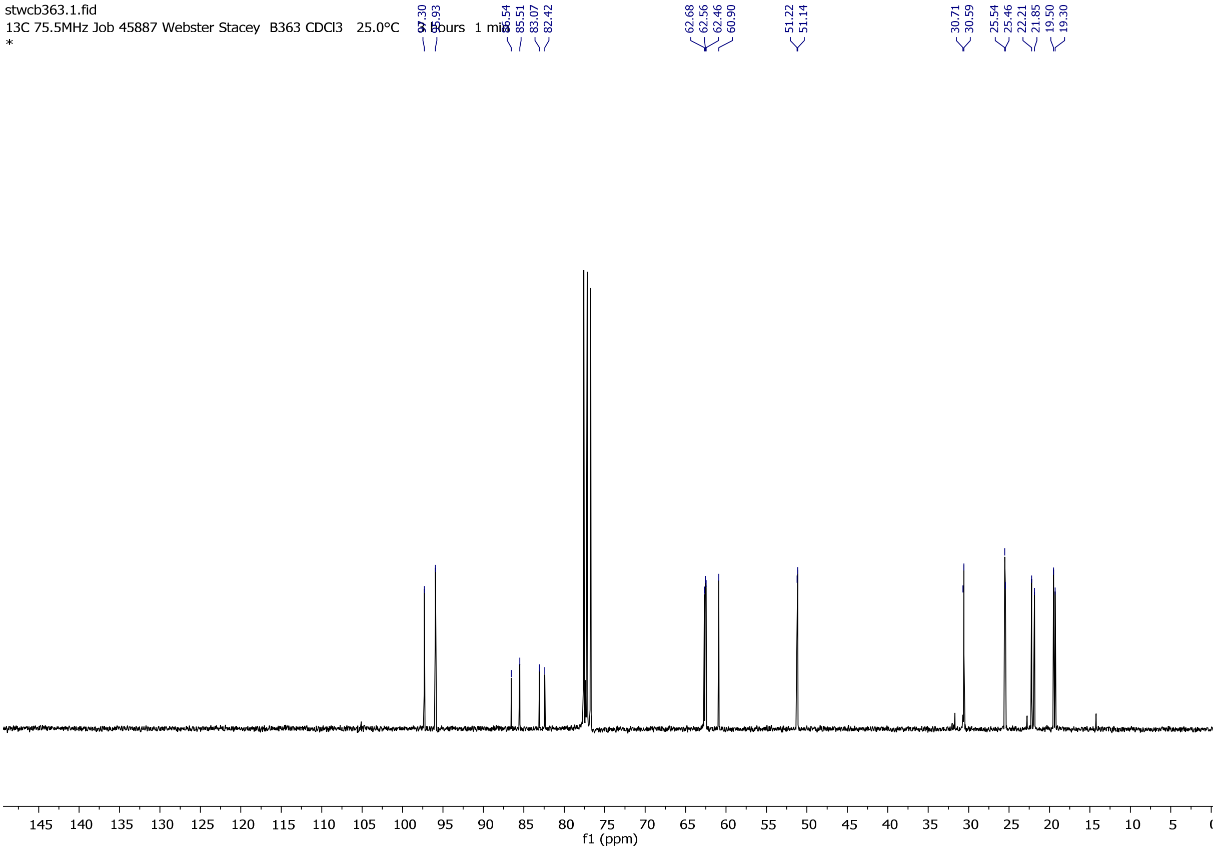
stwcb363.1.fid
13C 75.5MHz Job 45887 Webster Stacey B363 CDCl3 25.0°C 9 hours 1 min
97.30
95.93
86.54
85.51
83.07
82.42
62.68
62.56
62.46
60.90
51.22
51.14
30.71
30.59
25.54
25.46
22.21
21.85
19.50
19.30
f1 (ppm)

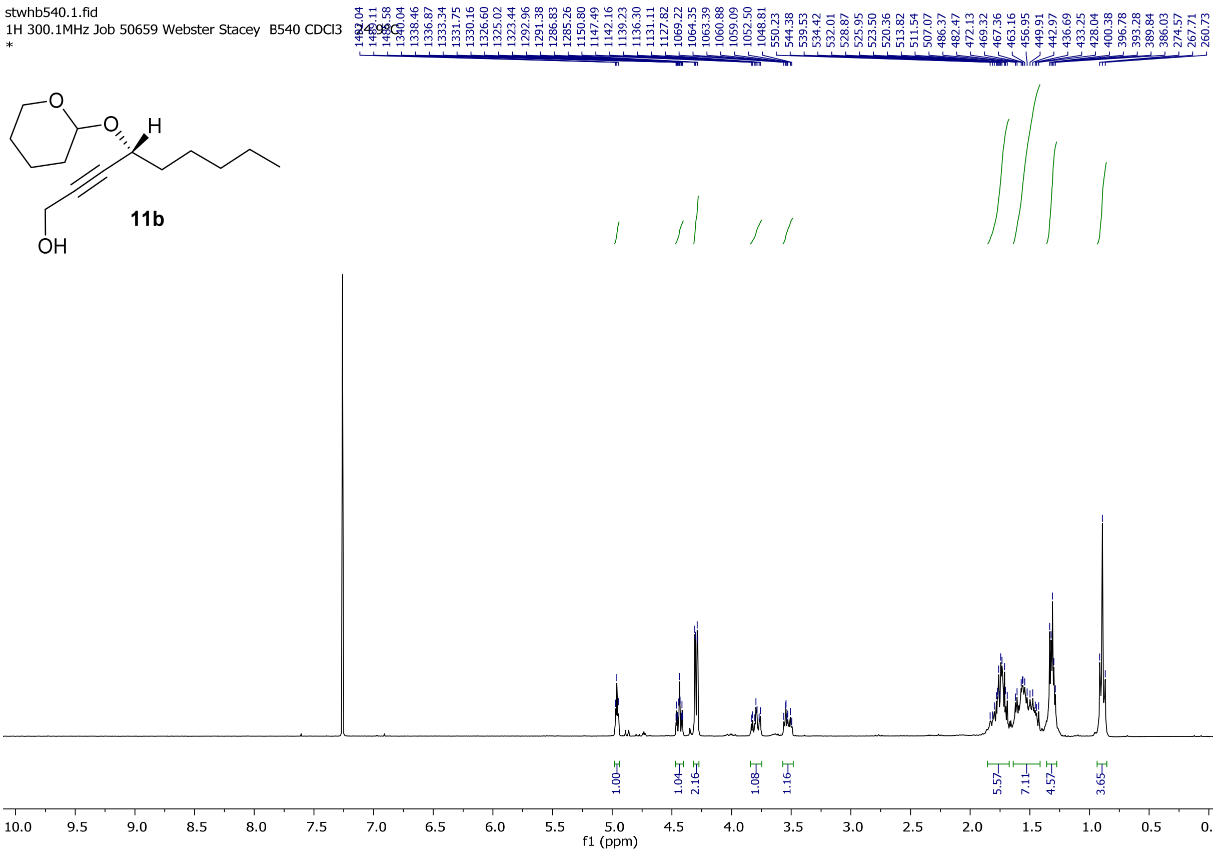
stwhb540.1.fid
1H 300.1MHz Job 50659 Webster Stacey B540 CDCl3 24.9°C
OH
11b
1.00
1.04
2.16
1.08
1.16
5.57
7.11
4.57
3.65
f1 (ppm)

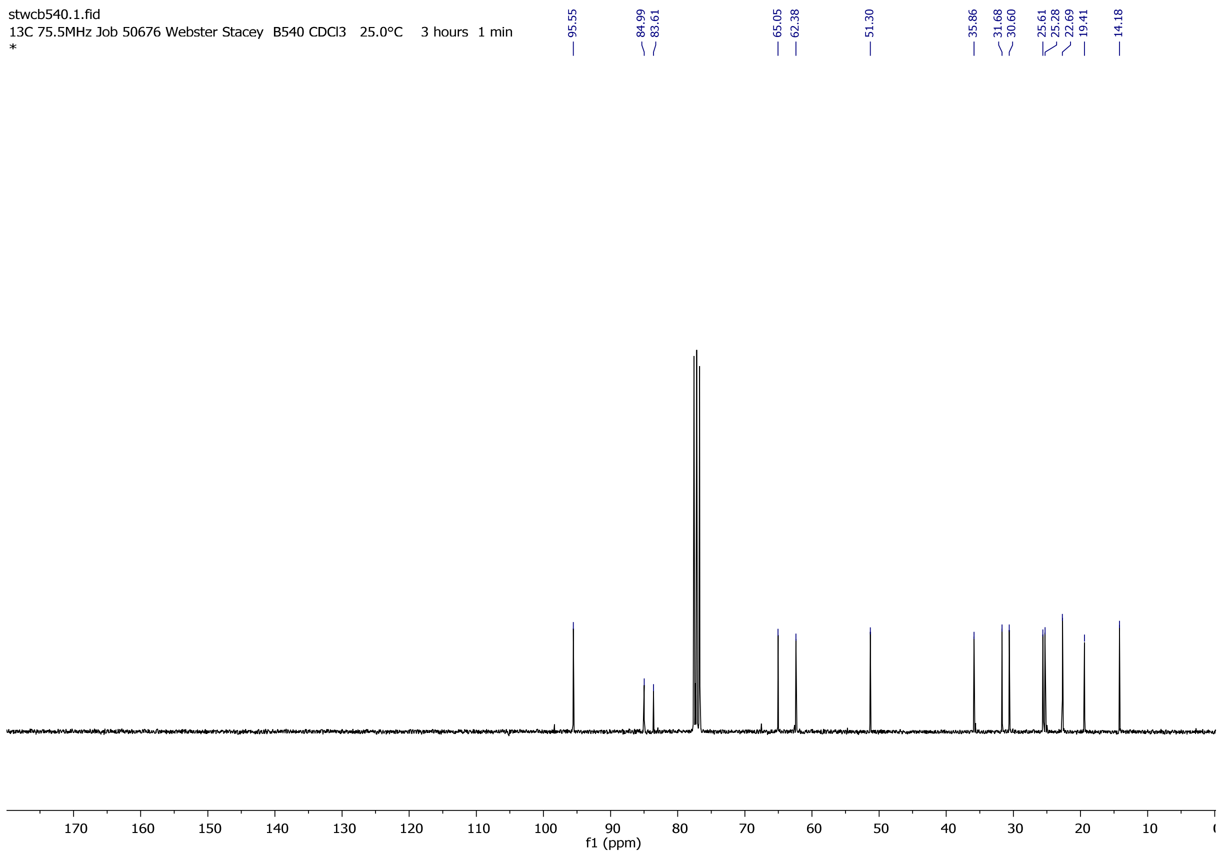

stwcb540.1.fid
13C 75.5MHz Job 50676 Webster Stacey B540 CDCl3 25.0°C 3 hours 1 min
*
95.55
84.99
83.61
65.05
62.38
51.30
35.86
31.68
30.60
25.61
25.28
22.69
19.41
14.18
170
160
150
140
130
120
110
100
90
80
70
60
50
40
30
20
10
f1 (ppm)

drsh3b201
1H 300.1MHz Job 51900 Sutherland Daniel R 3B201 CDCl3 25.0°C
*

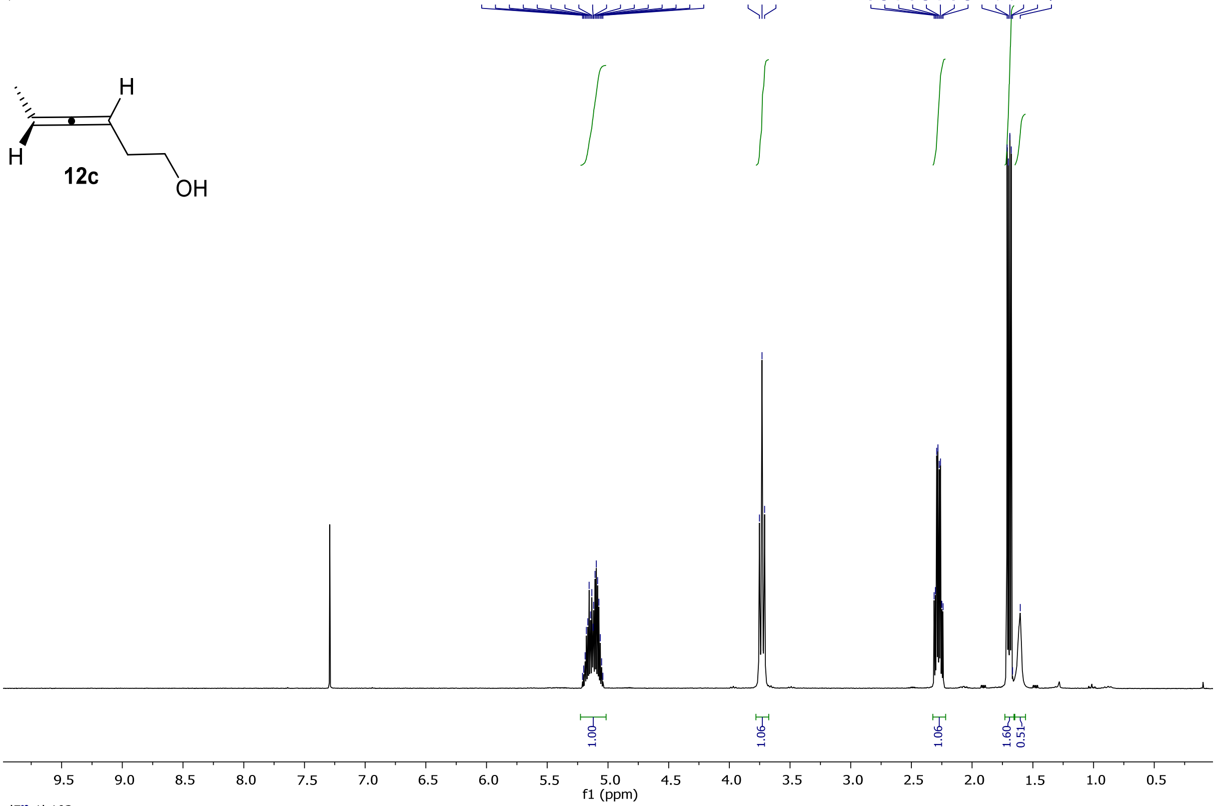

d5[illegible]c1b102
13C 75.5MHz Job 49056 Sutherland Daniel R 1B102 CDCl3 24.9°C 3 hours 1 min
*

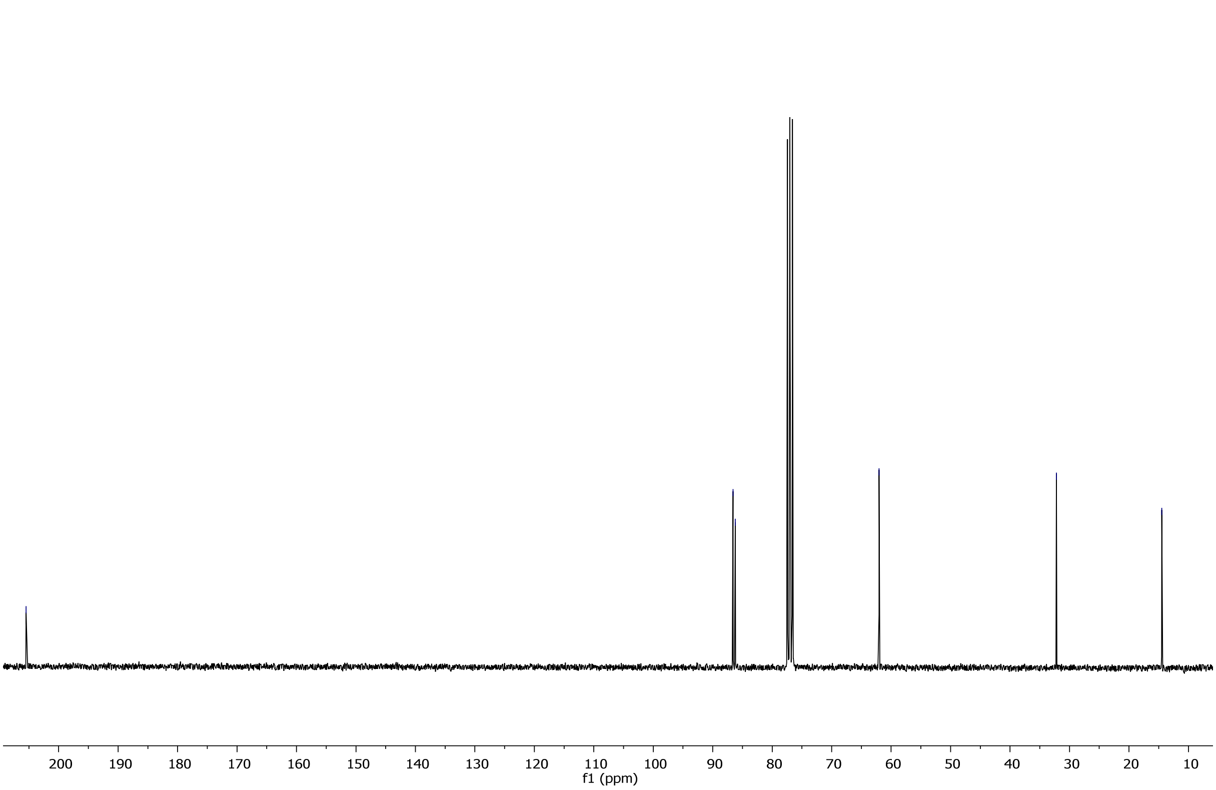

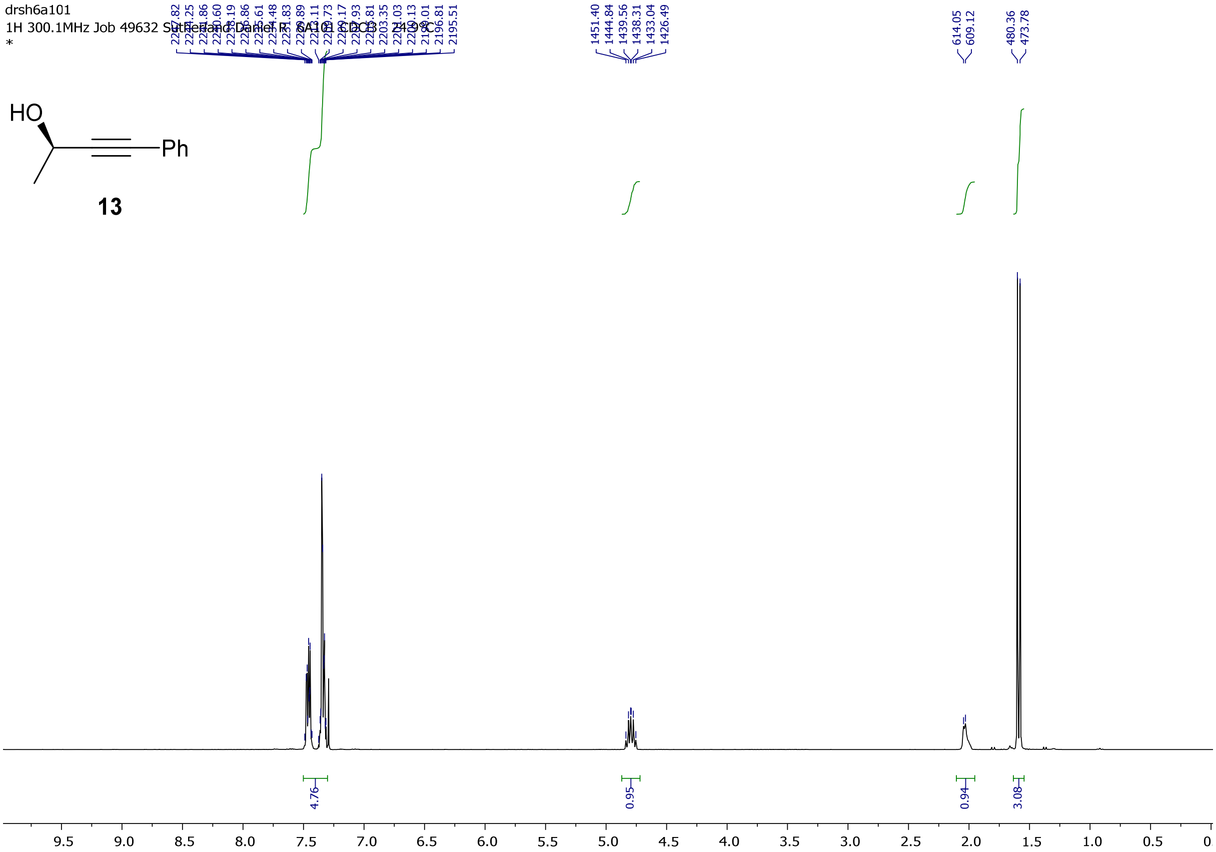
drsh6a101
*
HO
Ph
13
1451.40
1444.84
1439.56
1438.31
1433.04
1426.49
614.05
609.12
480.36
473.78
4.76
0.95
0.94
3.08
9.5 9.0 8.5 8.0 7.5 7.0 6.5 6.0 5.5 5.0 4.5 4.0 3.5 3.0 2.5 2.0 1.5 1.0 0.5 0

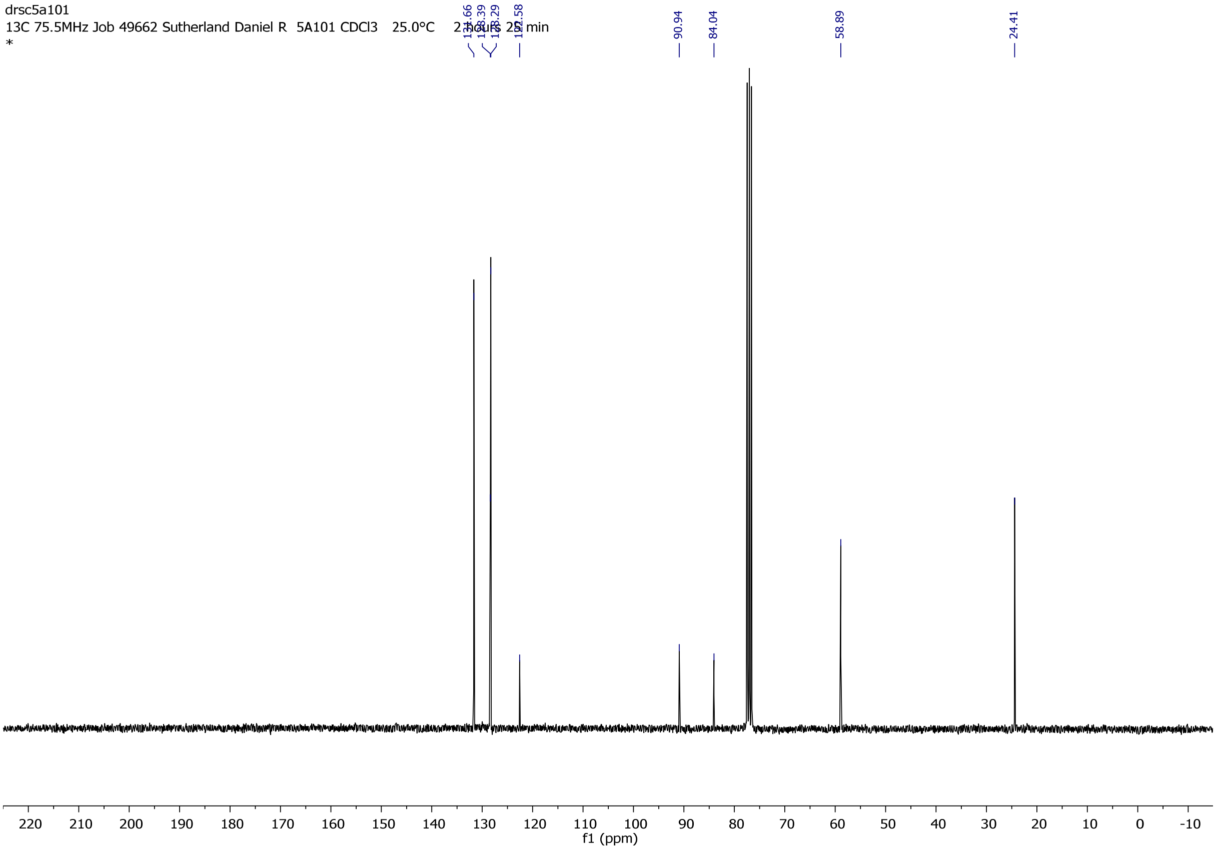
drsc5a101
13C 75.5MHz Job 49662 Sutherland Daniel R 5A101 CDCl3 25.0°C
*
90.94
84.04
58.89
24.41
220 210 200 190 180 170 160 150 140 130 120 110 100 90 80 70 60 50 40 30 20 10 0 -10
f1 (ppm)

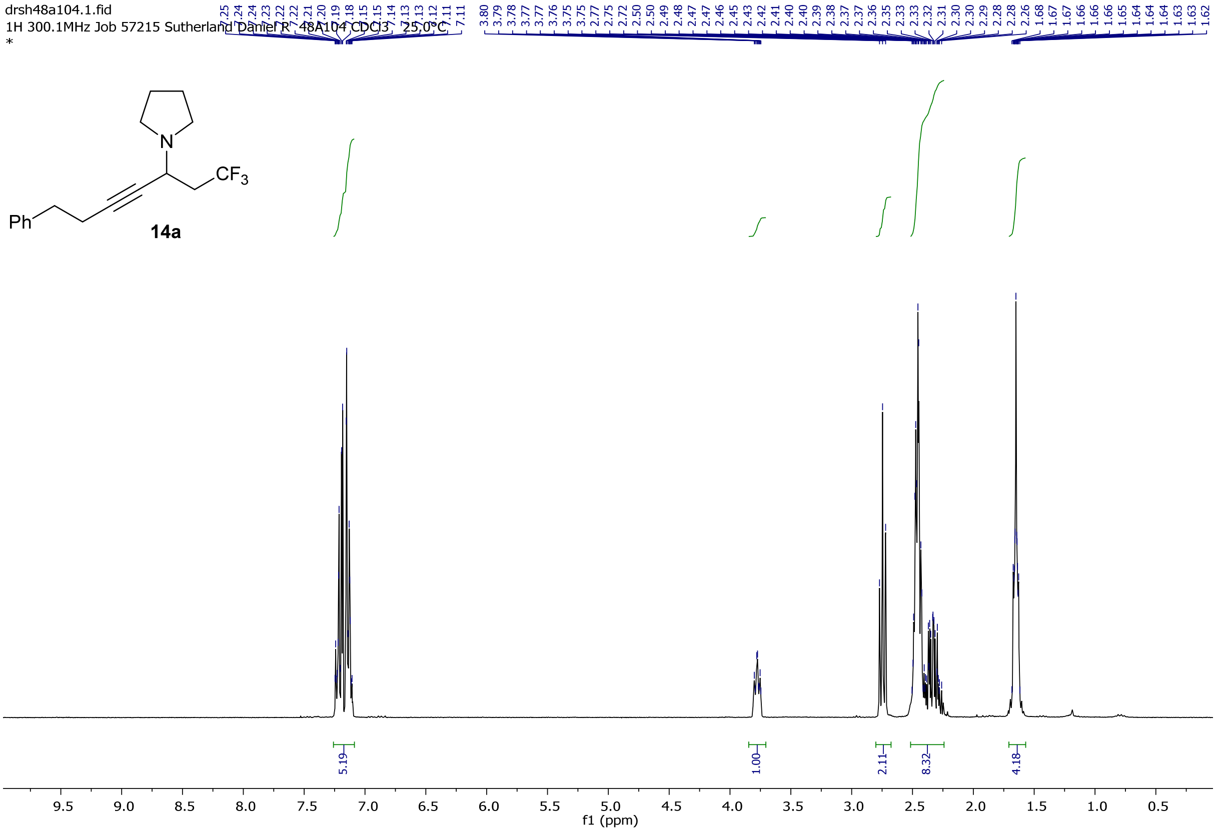
drsh48a104.1.fid
1H 300.1MHz Job 57215 Sutherland Daniel R 48A104 CDCl3 25.0°C
*
Ph
N
CF3
14a
5.19
1.00
2.11
8.32
4.18
f1 (ppm)

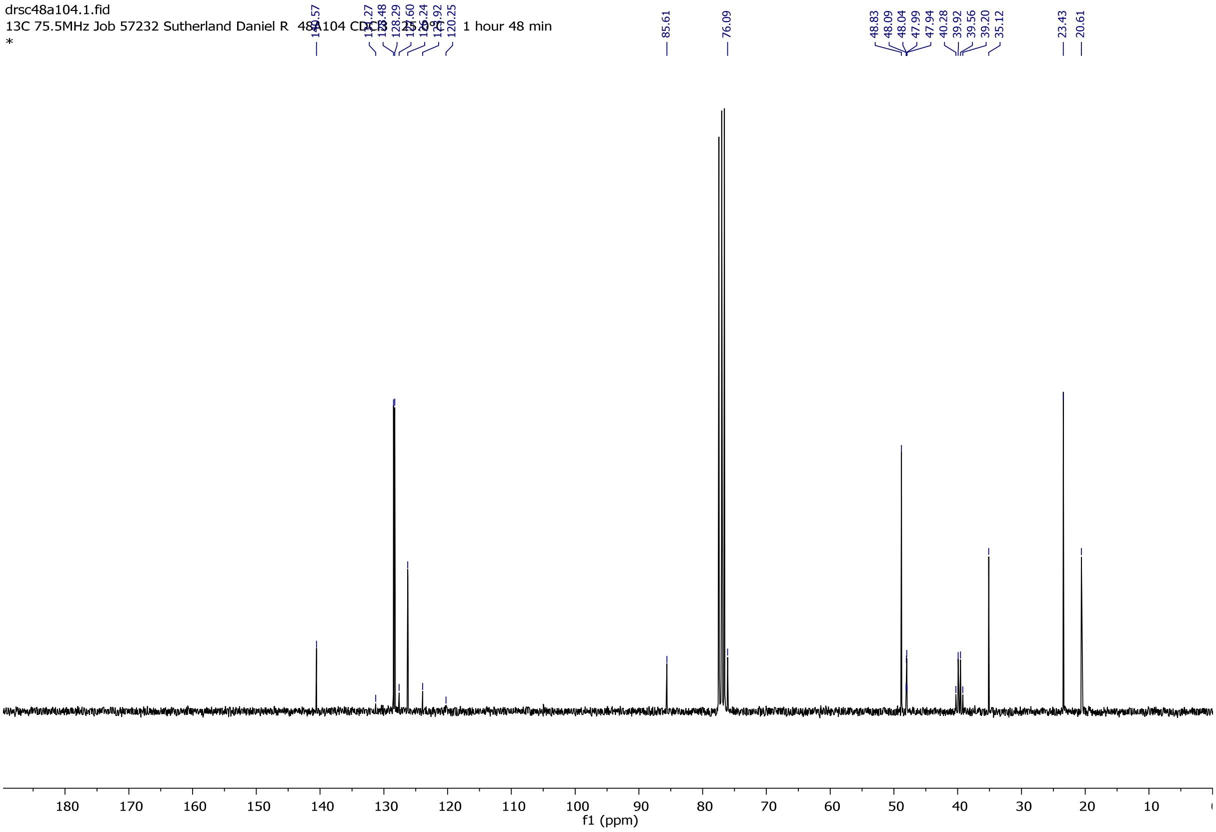
drsc48a104.1.fid
13C 75.5MHz Job 57232 Sutherland Daniel R 48A104 CDCl3 25.0°C 1 hour 48 min
*
85.61
76.09
48.83
48.09
48.04
47.99
47.94
40.28
39.92
39.56
39.20
35.12
23.43
20.61
f1 (ppm)

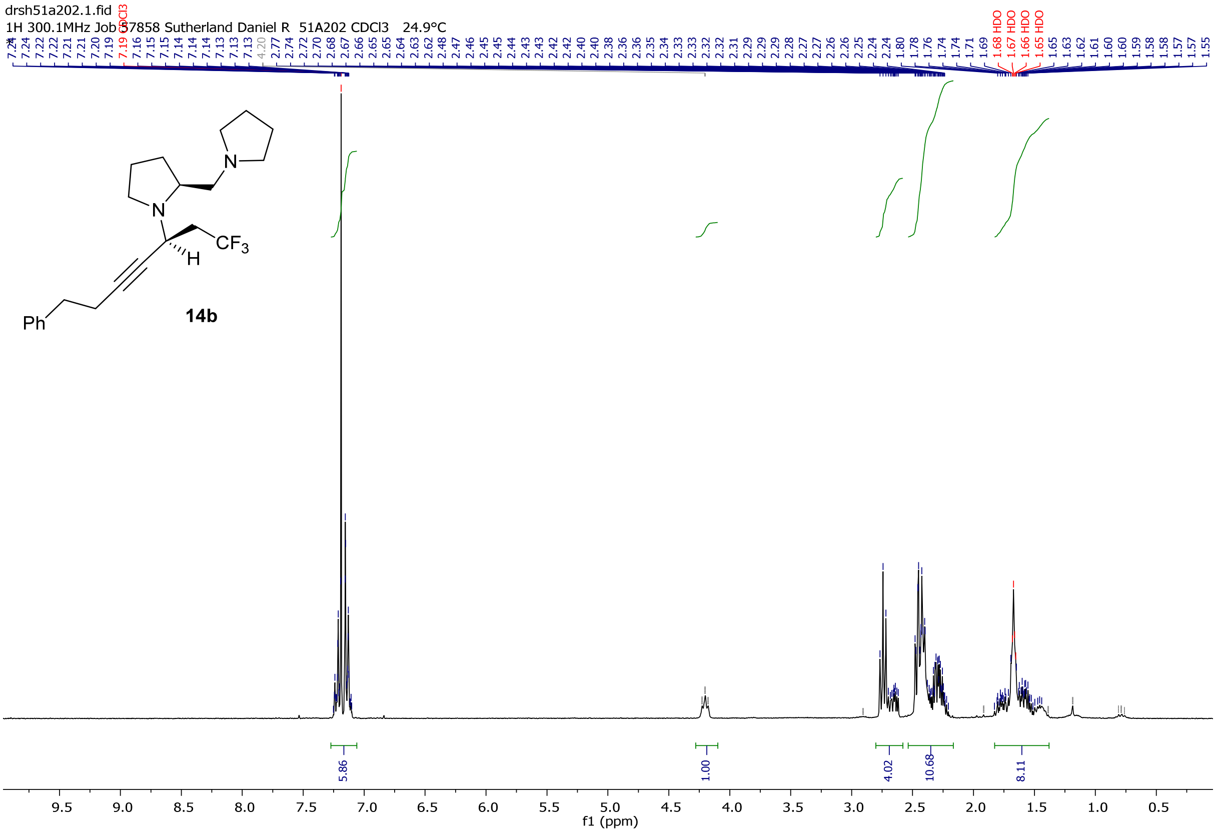
drsh51a202.1.fid
1H 300.1MHz Job 57858 Sutherland Daniel R 51A202 CDCl3 24.9°C
CF3
H
N
Ph
14b
5.86
1.00
4.02
10.68
8.11
f1 (ppm)

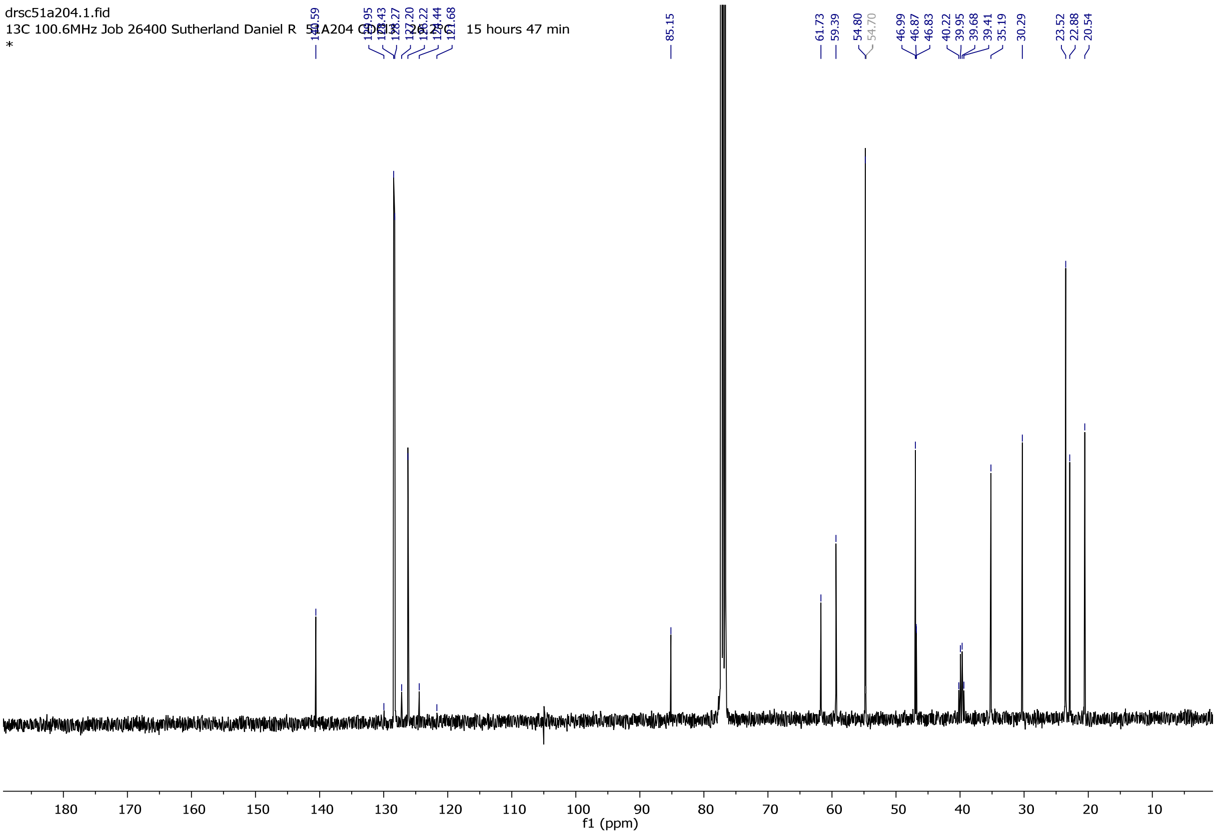
drsc51a204.1.fid
13C 100.6MHz Job 26400 Sutherland Daniel R 51A204 CDCl3 15 hours 47 min
f1 (ppm)

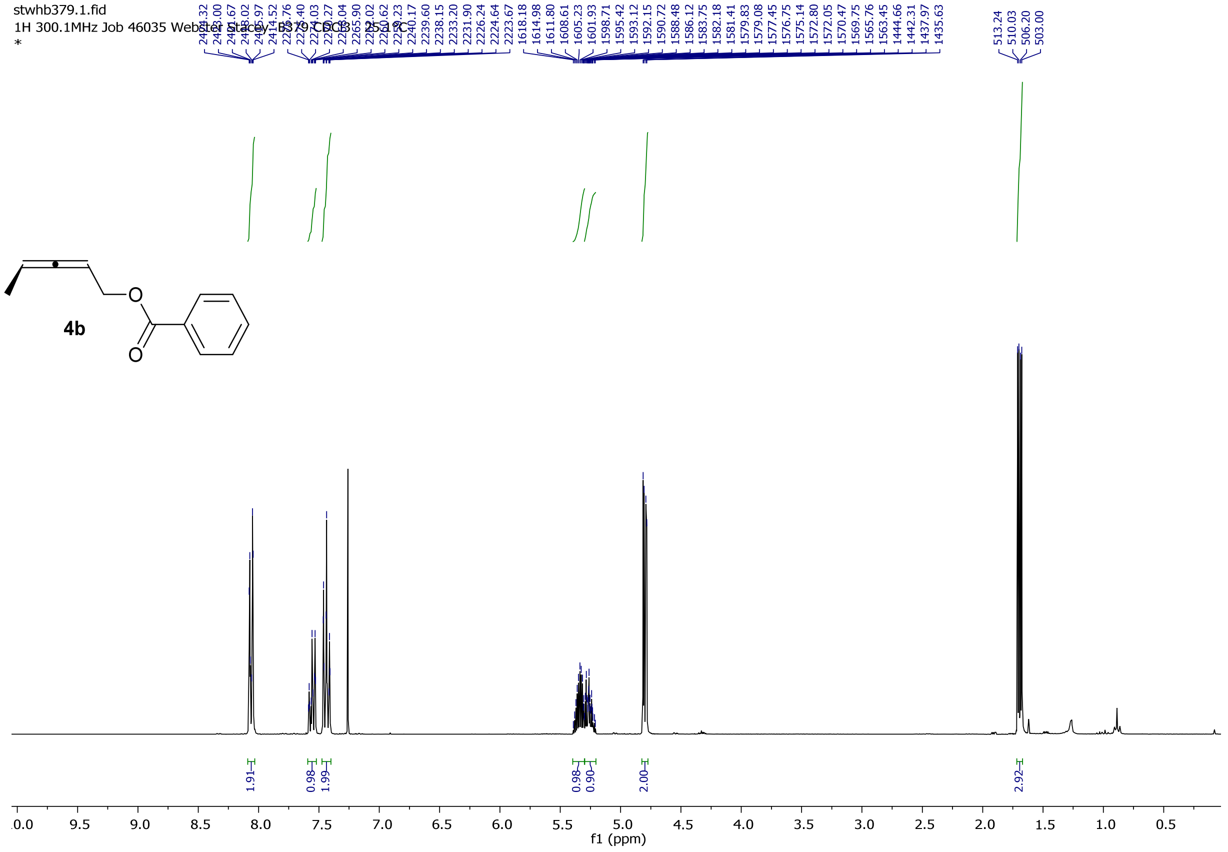
stwhb379.1.fid
1H 300.1MHz Job 46035 Webster Stacey B379 CDCl3 25.1°C
*
4b
1.91
0.98
1.99
0.98
0.90
2.00
2.92
513.24
510.03
506.20
503.00
f1 (ppm)

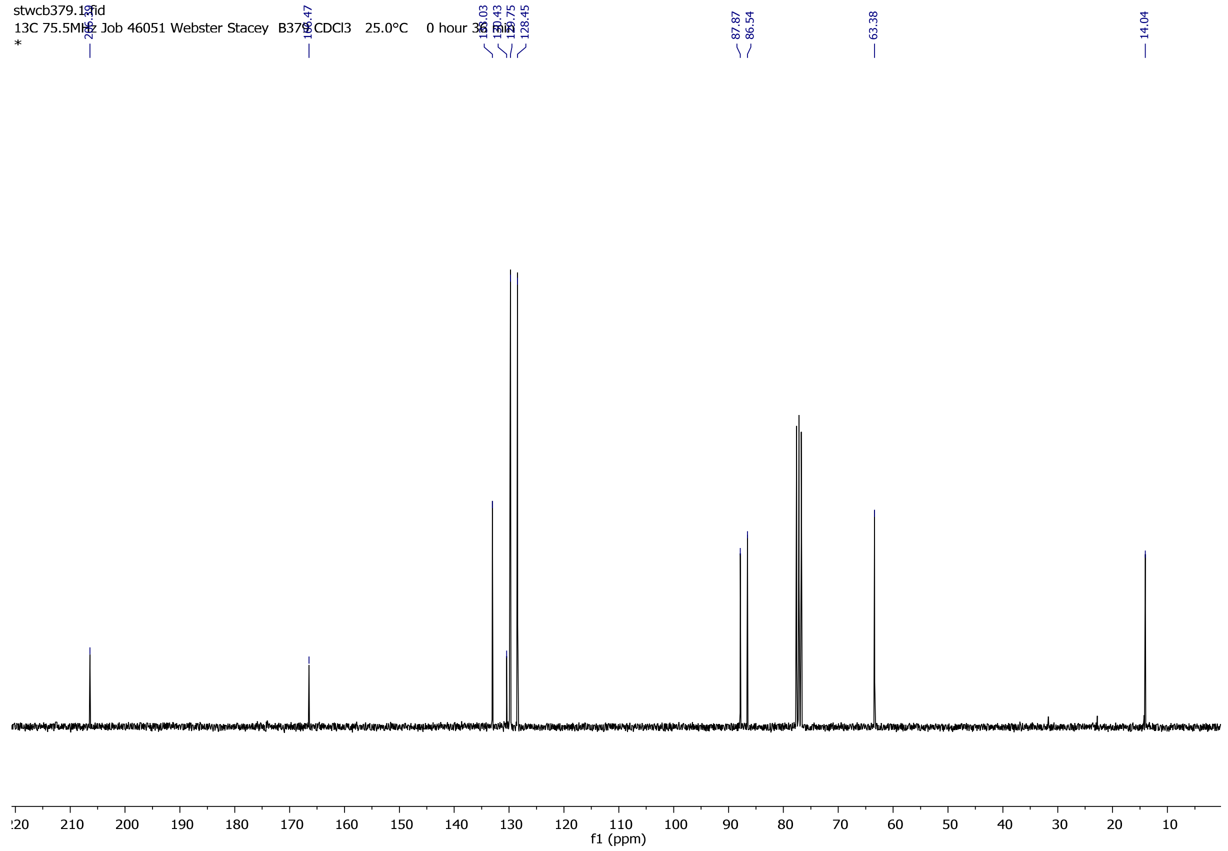
stwcb379.1.fid
13C 75.5MHz Job 46051 Webster Stacey B379 CDCl3 25.0°C
*
166.47
133.03
130.43
129.75
128.45
87.87
86.54
63.38
14.04
f1 (ppm)

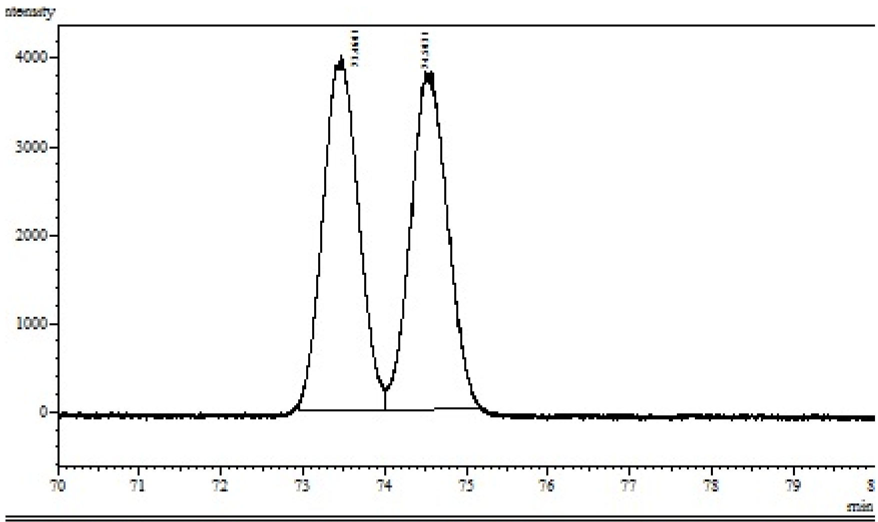

Peak#	Ret.Time	Area	Height	Conc.	Unit	Mark	ID#	Cmpd Name	Area%
1	73.460	120501	3982	50.138					50.1382
2	74.503	119837	3818	49.862		V			49.8618
Total		240338	7800						100.0000

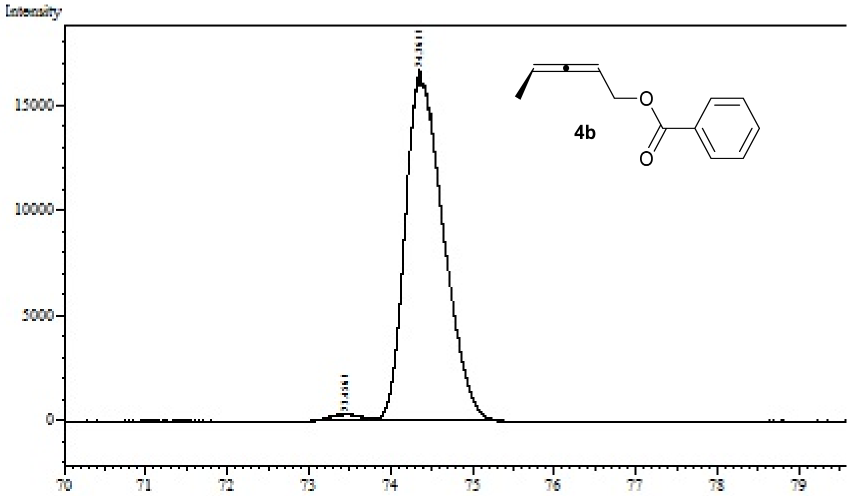

Peak#	Ret.Time	Area	Height	Conc.	Unit	Mark	ID#	Cmpd Name	Area%
1	73.456	6460	283	1.203					1.2026
2	74.361	530663	16614	98.797		V			98.7974
Total		537123	16897						100.0000

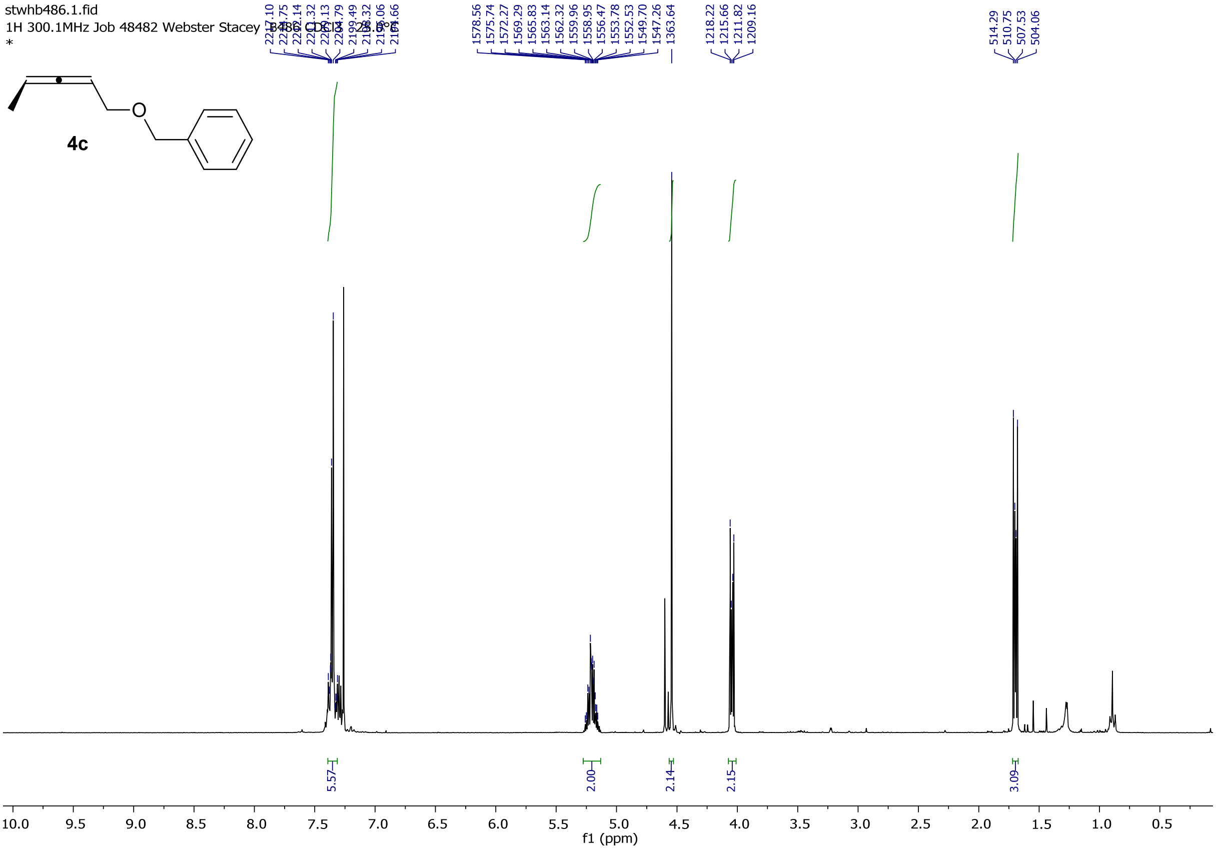
stwhb486.1.fid
1H 300.1MHz Job 48482 Webster Stacey
*
4c
1578.56
1575.74
1572.27
1569.29
1565.83
1563.14
1562.32
1559.96
1558.95
1556.47
1553.78
1552.53
1549.70
1547.26
1363.64
1218.22
1215.66
1211.82
1209.16
514.29
510.75
507.53
504.06
5.57
2.00
2.14
2.15
3.09
10.0 9.5 9.0 8.5 8.0 7.5 7.0 6.5 6.0 5.5 5.0 4.5 4.0 3.5 3.0 2.5 2.0 1.5 1.0 0.5
f1 (ppm)

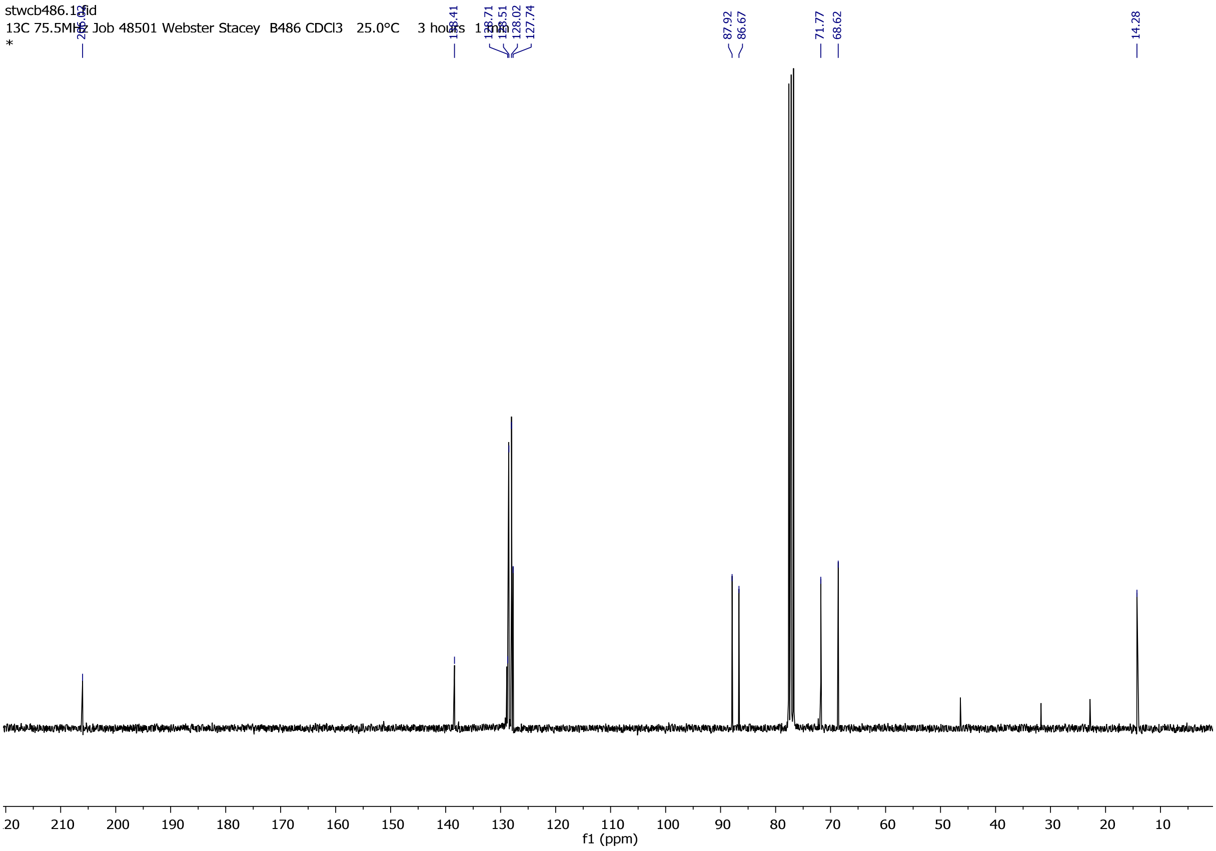
13C 75.5MHz Job 48501 Webster Stacey B486 CDCl3 25.0°C
*
138.41
128.71
128.51
128.02
127.74
87.92
86.67
71.77
68.62
14.28
210 200 190 180 170 160 150 140 130 120 110 100 90 80 70 60 50 40 30 20 10
f1 (ppm)

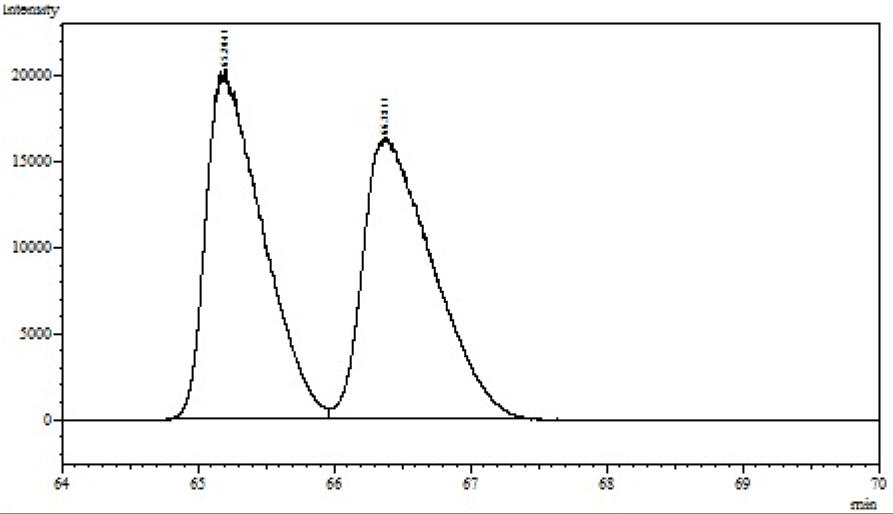

Peak#	Ret.Time	Area	Height	Conc.	Unit	Mark	ID#	Cmpd Name	Area%
1	65.204	580929	20289	49.938					49.9381
2	66.381	582368	16306	50.062		V			50.0619
Total		1163297	36595						100.0000

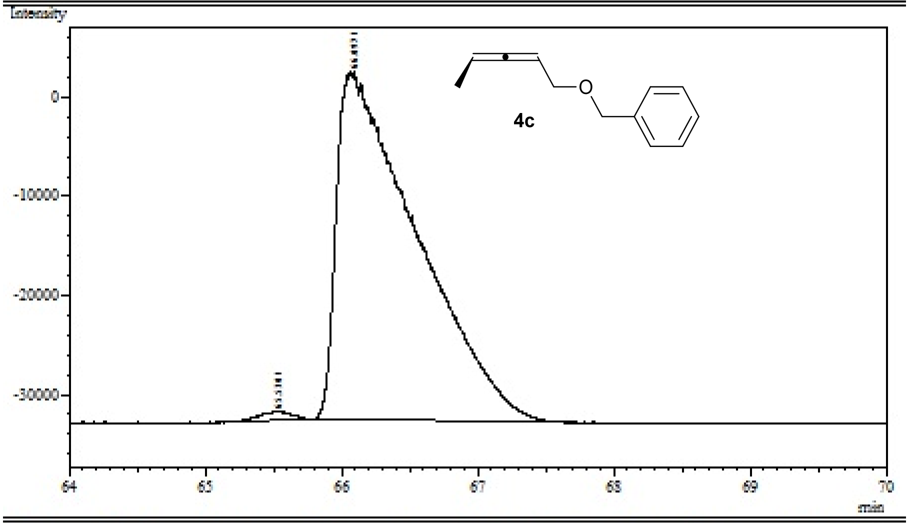

Peak#	Ret.Time	Area	Height	Conc.	Unit	Mark	ID#	Cmpd Name	Area%
1	65.530	15663	887	1.077					1.0774
2	66.097	1438144	35009	98.923					98.9226
Total		1453807	35896						100.0000

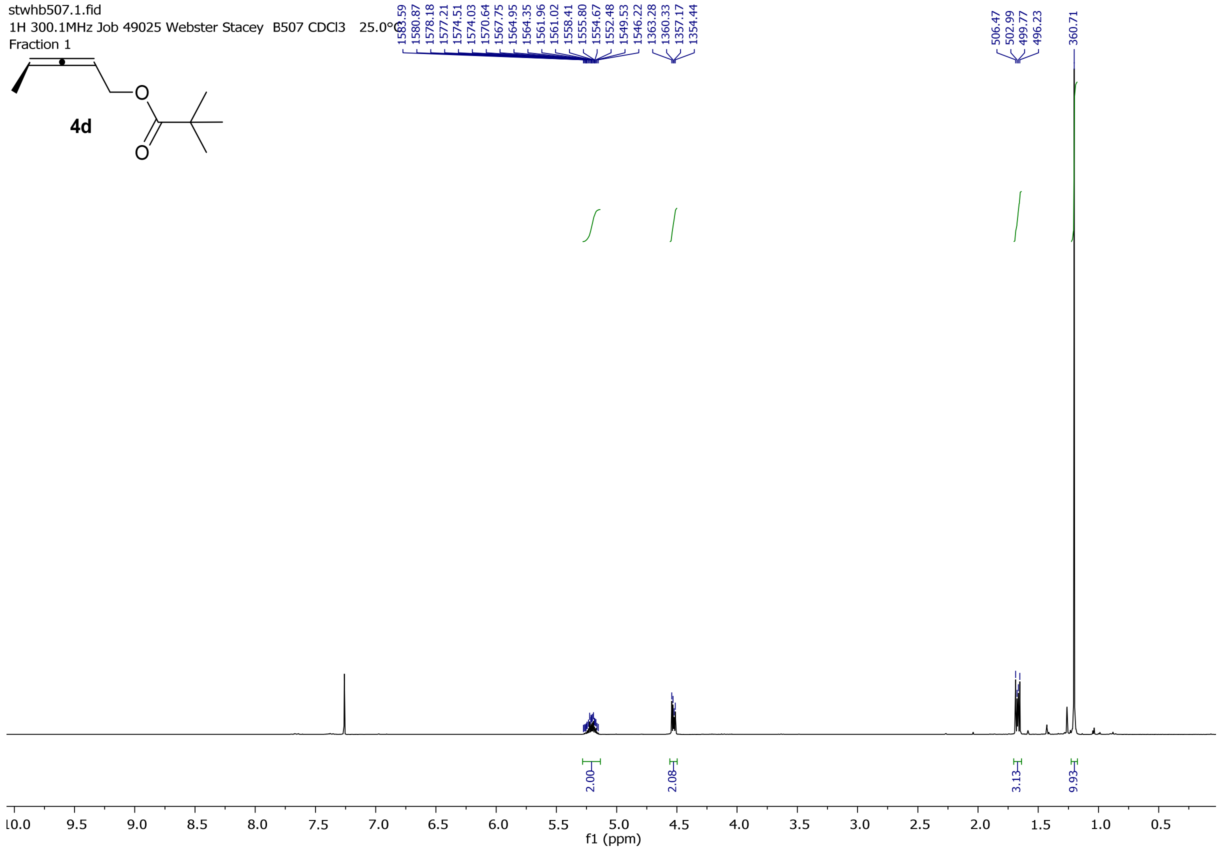
stwhb507.1.fid
1H 300.1MHz Job 49025 Webster Stacey B507 CDCl3 25.0°C
Fraction 1
4d
1580.87
1578.18
1577.21
1574.51
1574.03
1570.64
1567.75
1564.95
1564.35
1561.96
1561.02
1558.41
1555.80
1554.67
1552.48
1549.53
1546.22
1363.28
1360.33
1357.17
1354.44
506.47
502.99
499.77
496.23
360.71
2.00
2.08
3.13
9.93
f1 (ppm)

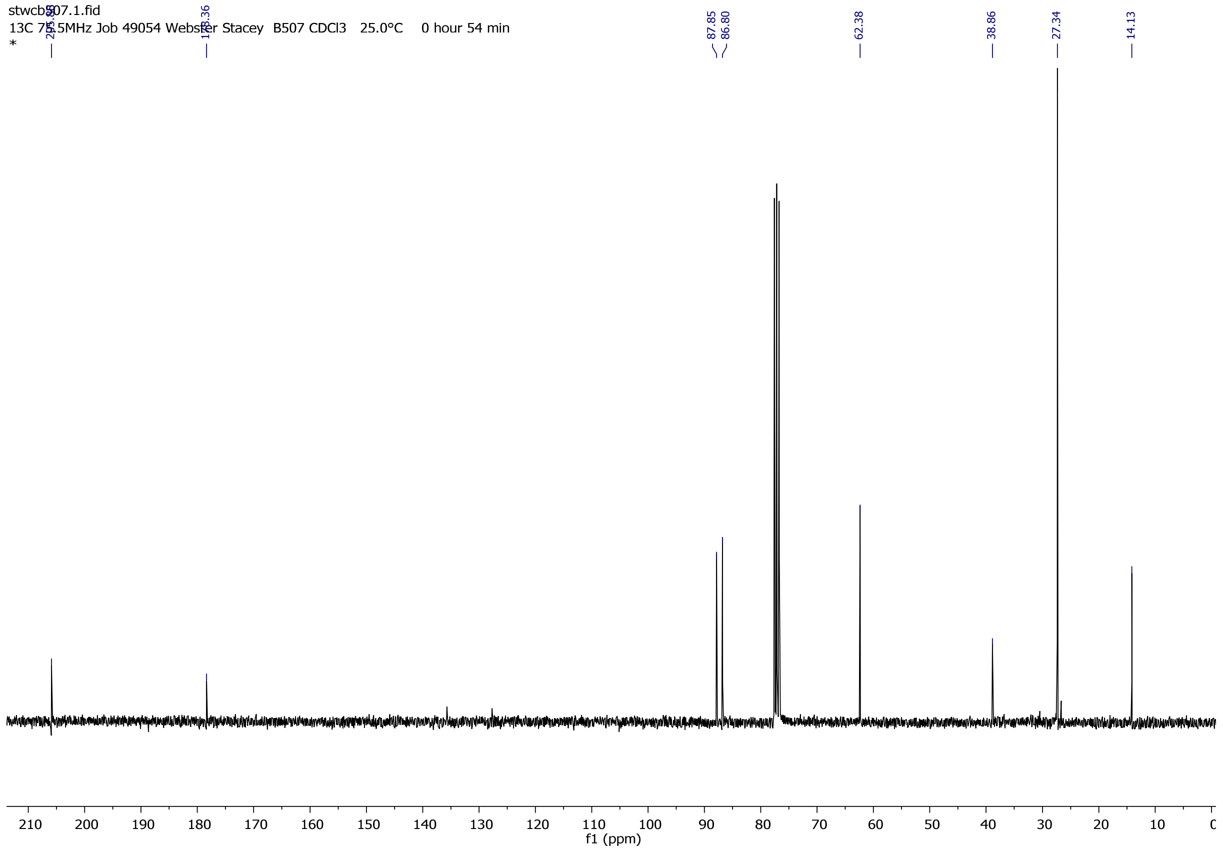
13C 75.5MHz Job 49054 Webster Stacey B507 CDCl3 25.0°C 0 hour 54 min
87.85
86.80
62.38
38.86
27.34
14.13
f1 (ppm)

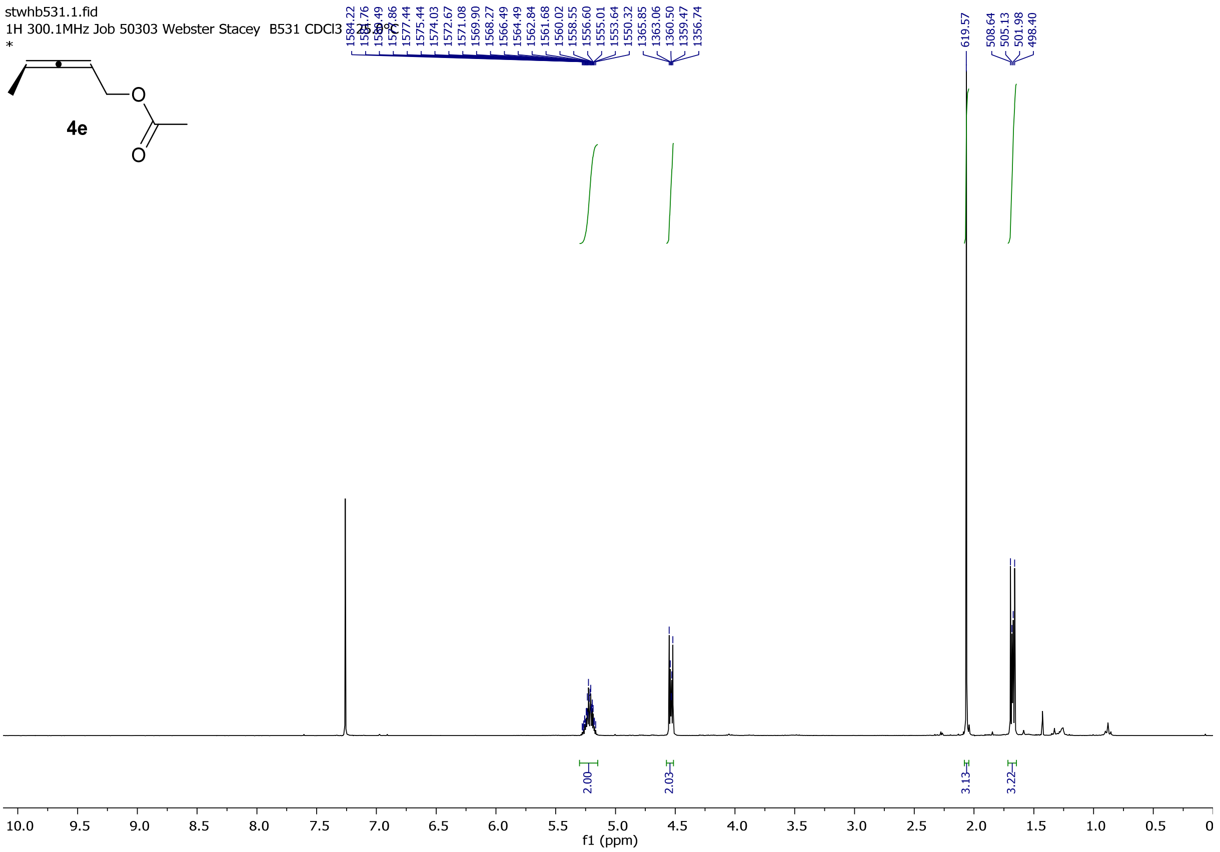
stwhb531.1.fid
1H 300.1MHz Job 50303 Webster Stacey B531 CDCl3 25.0°C
4e
2.00
2.03
3.13
3.22
f1 (ppm)

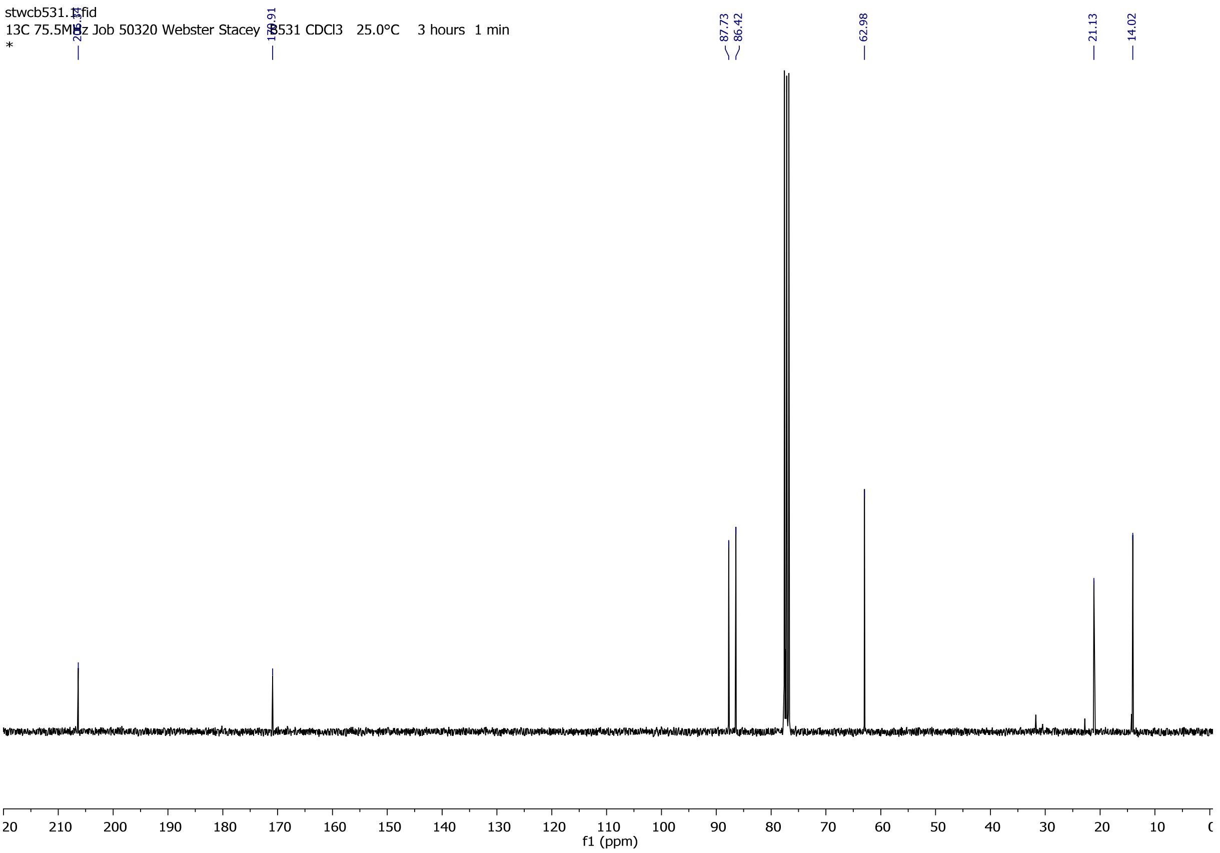
stwcb531.1.fid
13C 75.5MHz Job 50320 Webster Stacey B531 CDCl3 25.0°C 3 hours 1 min
170.91
87.73
86.42
62.98
21.13
14.02
f1 (ppm)

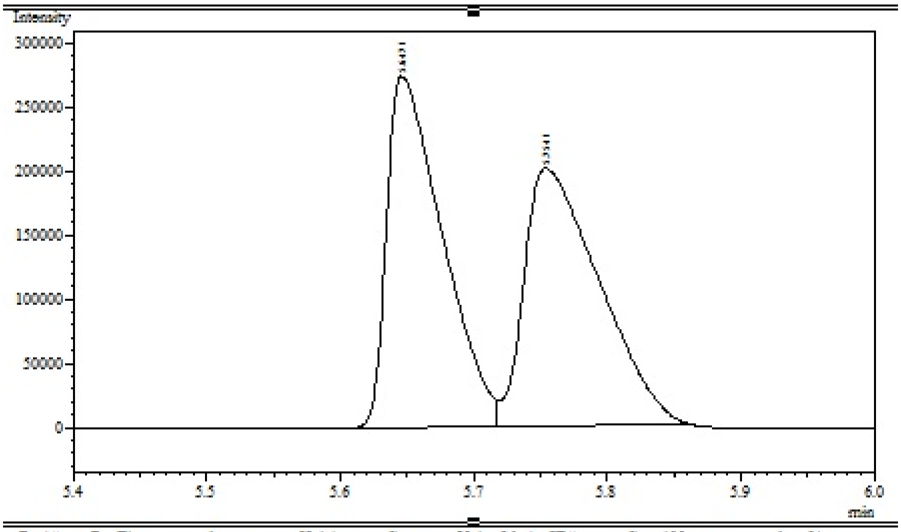

Peak#	Ret.Time	Area	Height	Conc.	Unit	Mark	ID#	Cmpd Name	Area%
1	5.647	794916	272656	50.010					50.0096
2	5.754	794611	200712	49.990		V			49.9904
Total		1589527	473368						100.0000

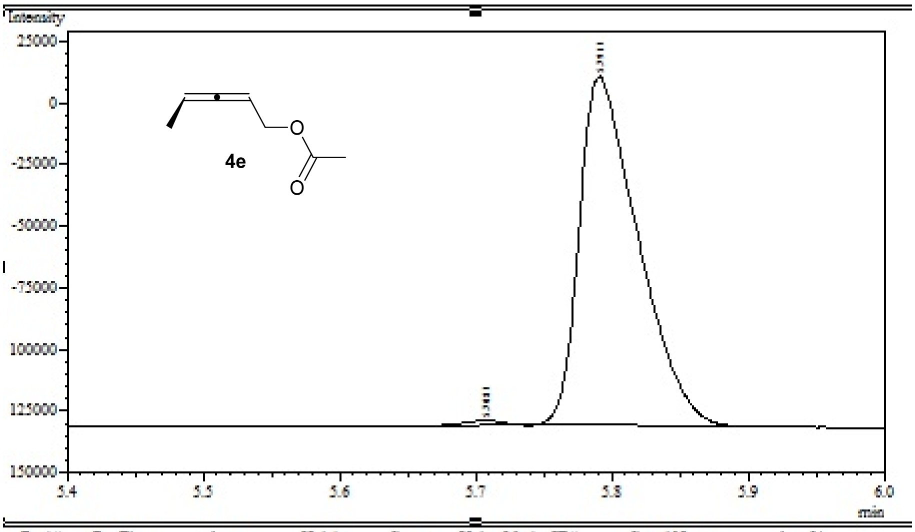

Peak#	Ret.Time	Area	Height	Conc.	Unit	Mark	ID#	Cmpd Name	Area%
1	5.708	4448	2049	1.034					1.0342
2	5.791	425677	140561	98.966					98.9658
Total		430125	142610						100.0000

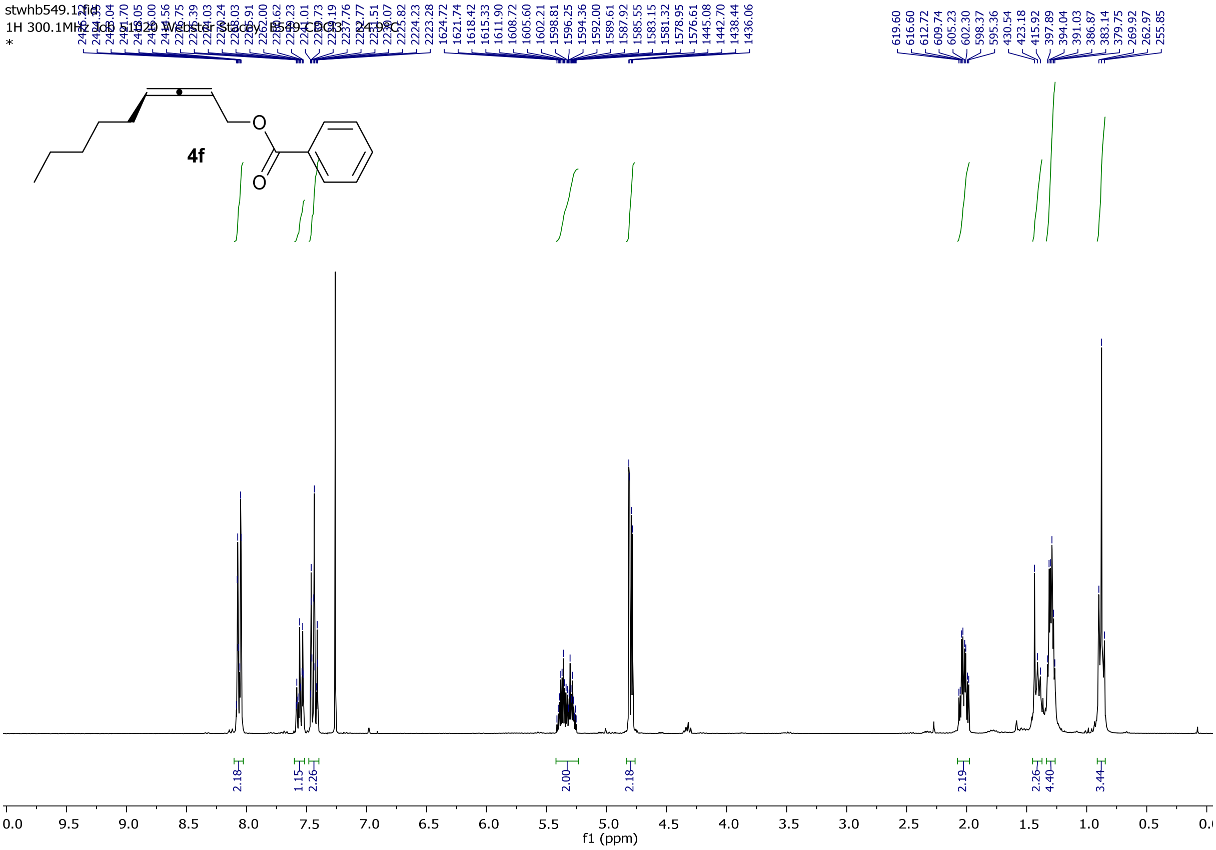
stwhb549.1.fid
4f
f1 (ppm)
2.18
1.15
2.26
2.00
2.18
2.19
2.26
4.40
3.44

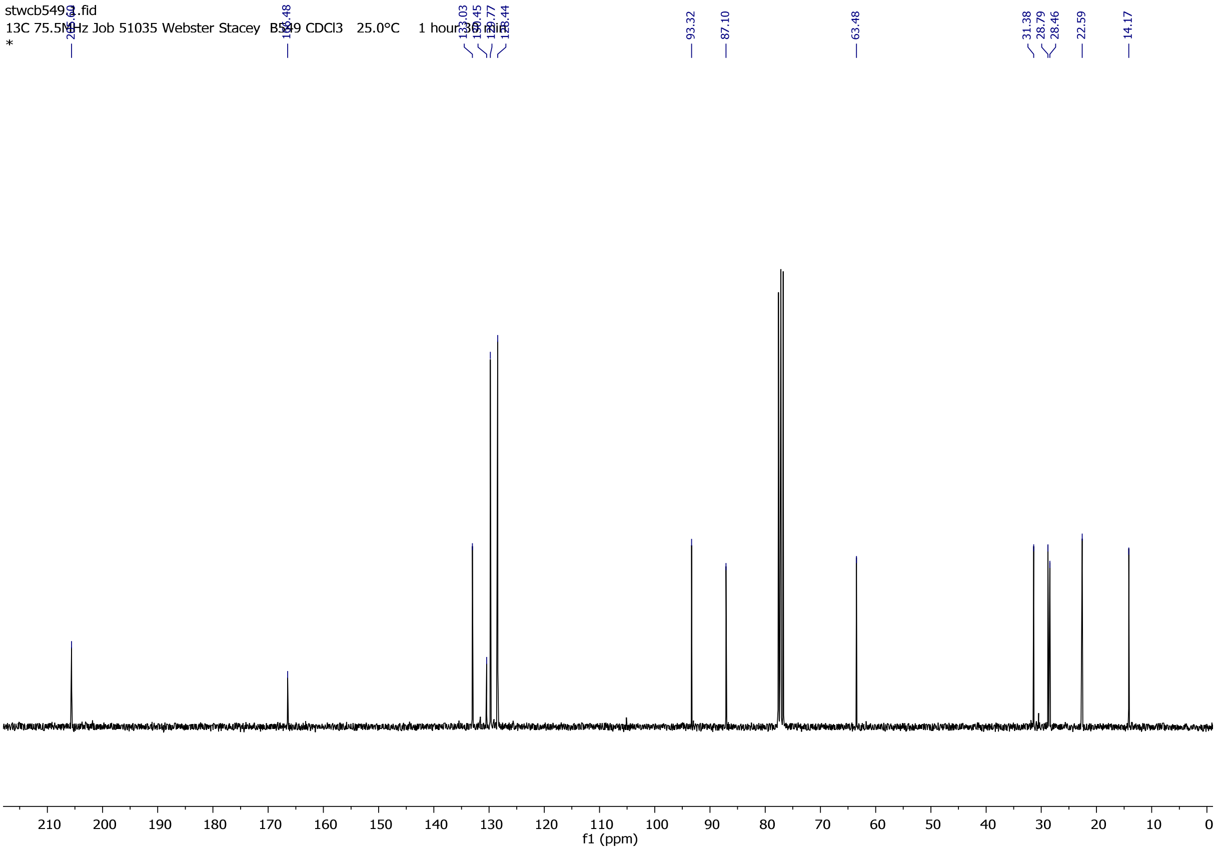
13C 75.5MHz Job 51035 Webster Stacey CDCl3 25.0°C
93.32
87.10
63.48
31.38
28.79
28.46
22.59
14.17
f1 (ppm)

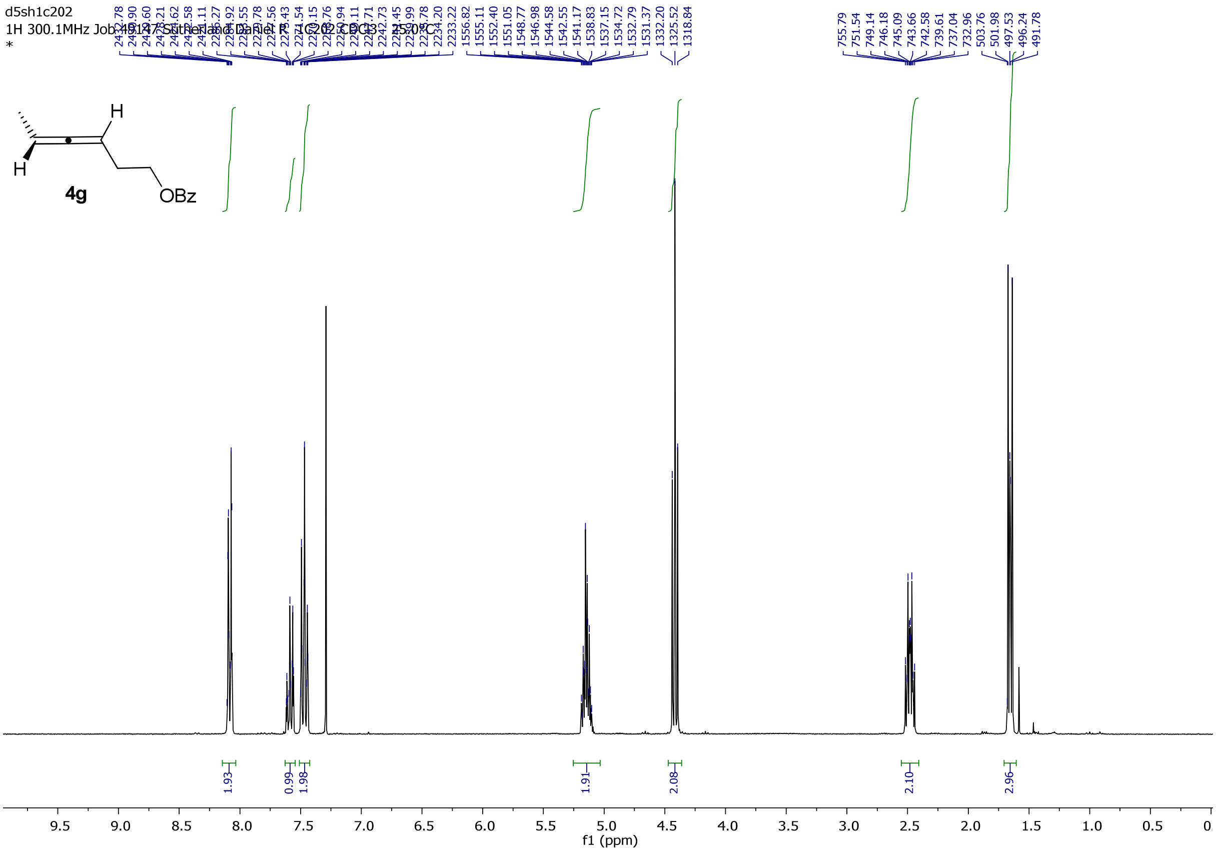
d5sh1c202
1H 300.1MHz
*
H
H
4g
OBz
755.79
751.54
749.14
746.18
745.09
743.66
742.58
739.61
737.04
732.96
503.76
501.98
497.53
496.24
491.78
1.93
0.99
1.98
1.91
2.08
2.10
2.96
f1 (ppm)

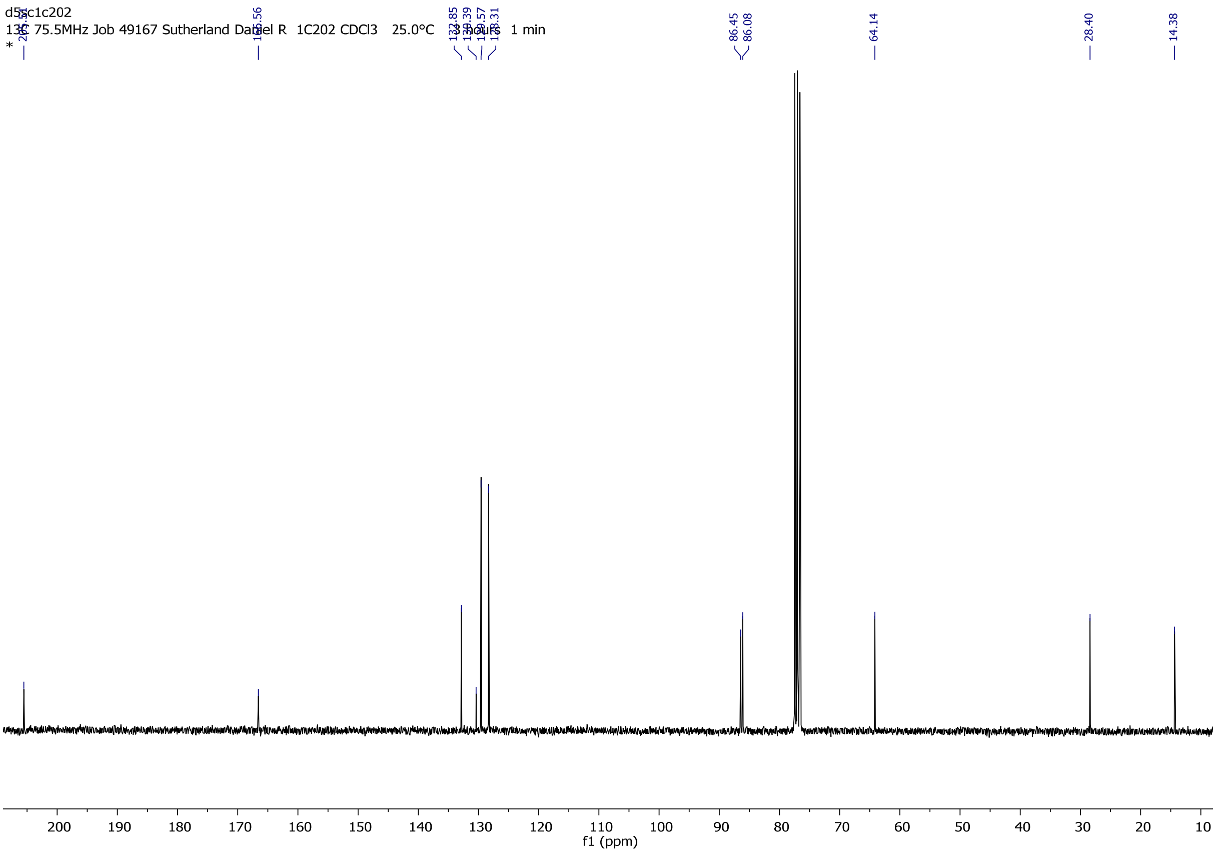
*
86.45
86.08
64.14
28.40
14.38
f1 (ppm)

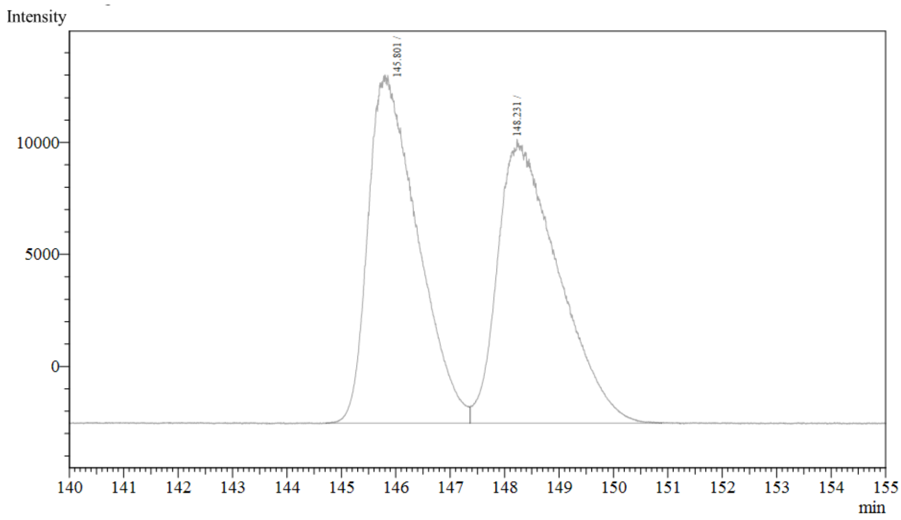

Peak#	Ret.Time	Area	Height	Conc.	Unit	Mark	ID#	Cmpd Name	Area%
1	145.801	979615	15528	50.024					50.0242
2	148.231	978665	12666	49.976		V			49.9758
Total		1958280	28194						100.0000

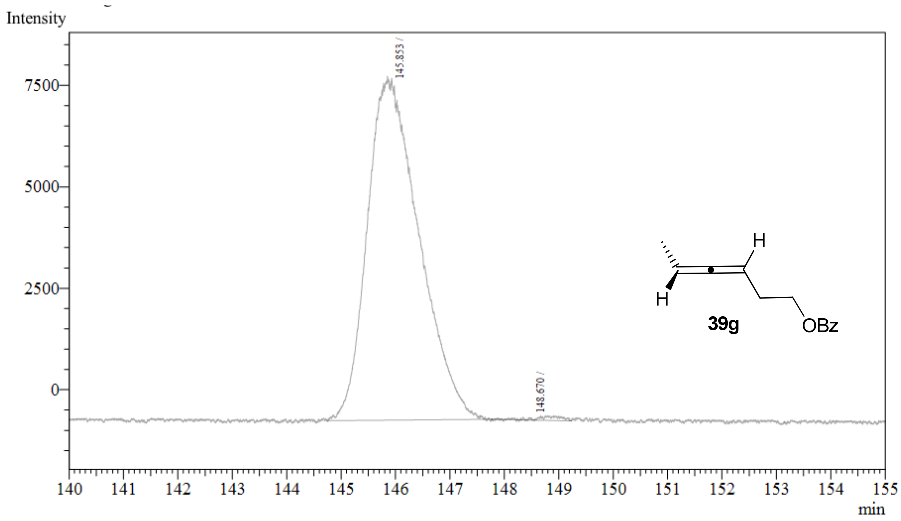

39g

Peak#	Ret.Time	Area	Height	Conc.	Unit	Mark	ID#	Cmpd Name	Area%
1	145.853	557077	8433	99.482					99.4824
2	148.670	2899	100	0.518					0.5176
Total		559976	8533						100.0000

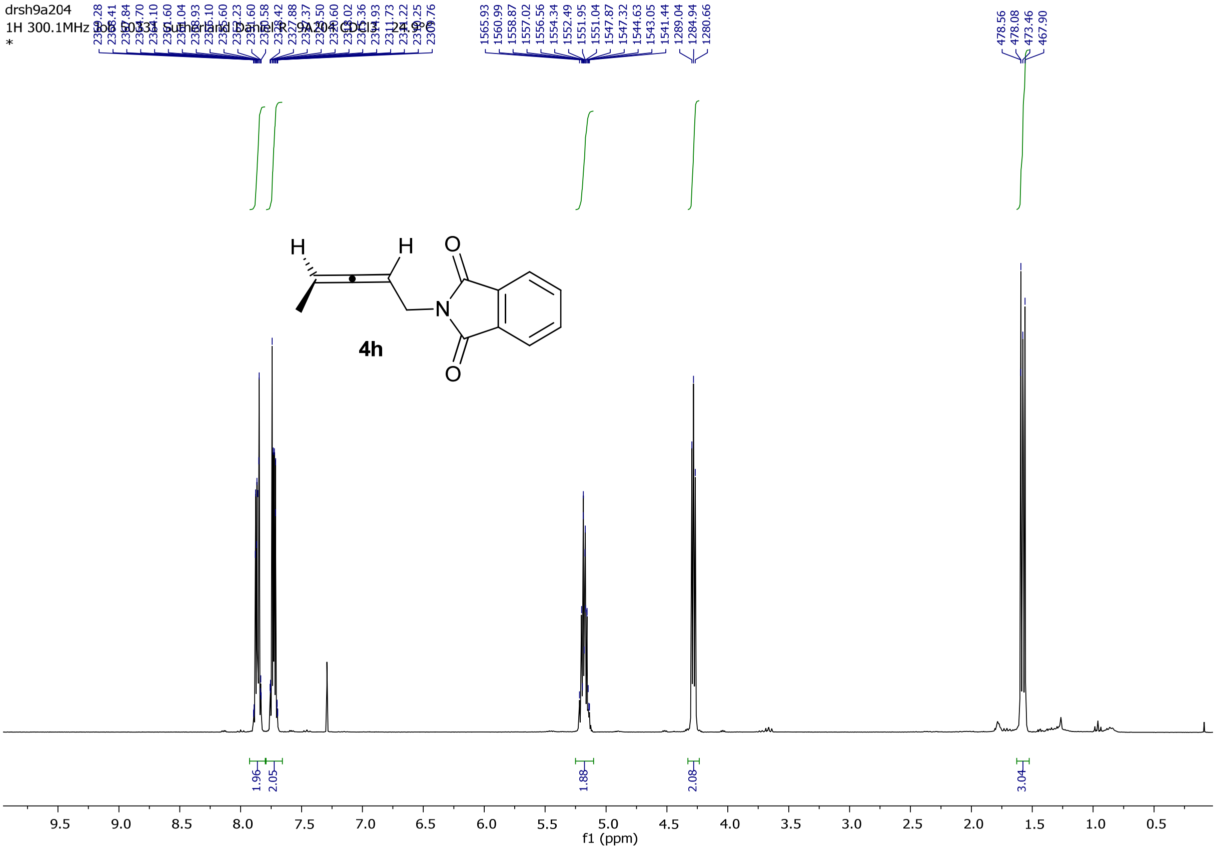
drsh9a204
1H 300.1MHz Job 50333 Sutherland Daniel R. 9A204 CDCl3 24.9°C
*
1565.93
1560.99
1558.87
1557.02
1556.56
1554.34
1552.49
1551.95
1551.04
1547.87
1547.32
1544.63
1543.05
1541.44
1289.04
1284.94
1280.66
478.56
478.08
473.46
467.90
H
H
O
N
O
4h
1.96
2.05
1.88
2.08
3.04
9.5 9.0 8.5 8.0 7.5 7.0 6.5 6.0 5.5 5.0 4.5 4.0 3.5 3.0 2.5 2.0 1.5 1.0 0.5
f1 (ppm)

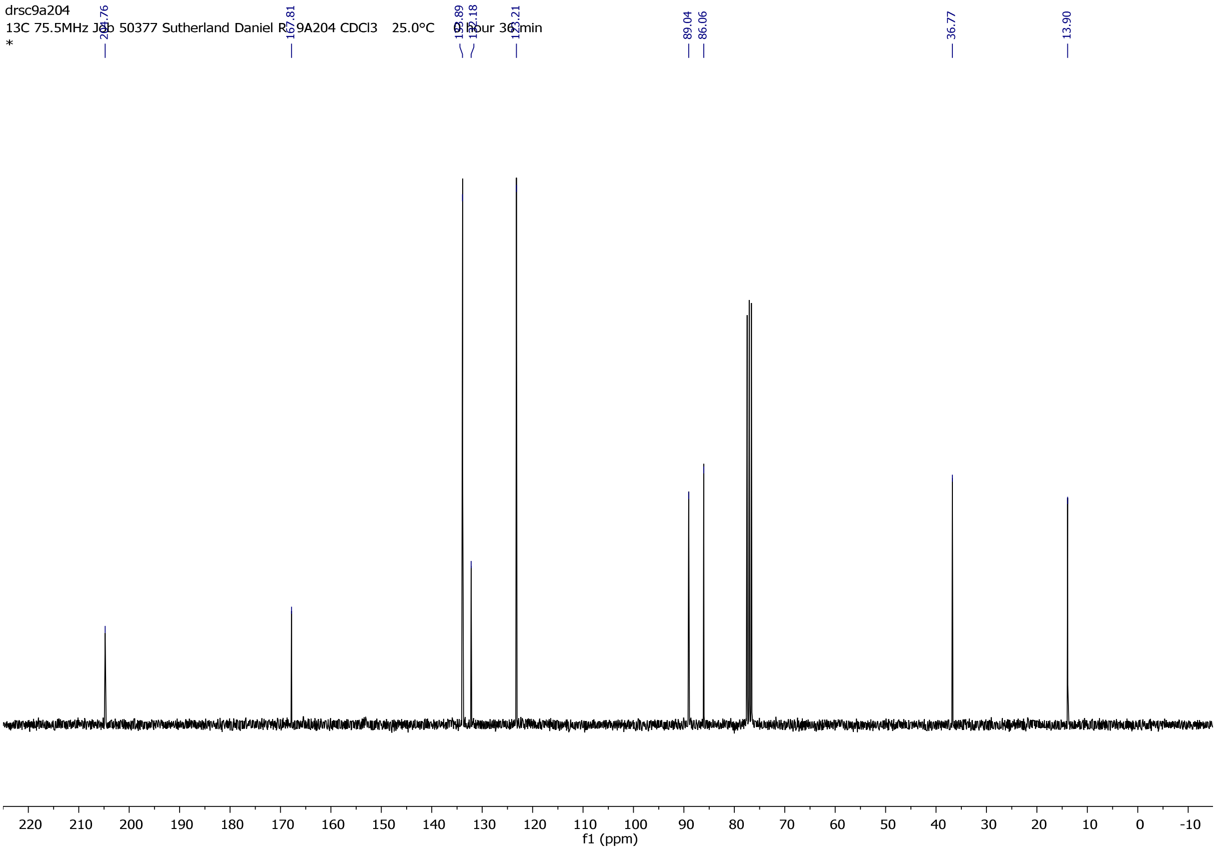
drsc9a204
13C 75.5MHz Job 50377 Sutherland Daniel R. 9A204 CDCl3 25.0°C 1 hour 36 min
*
204.76
167.81
133.89
132.18
123.21
89.04
86.06
36.77
13.90
220 210 200 190 180 170 160 150 140 130 120 110 100 90 80 70 60 50 40 30 20 10 0 -10
f1 (ppm)

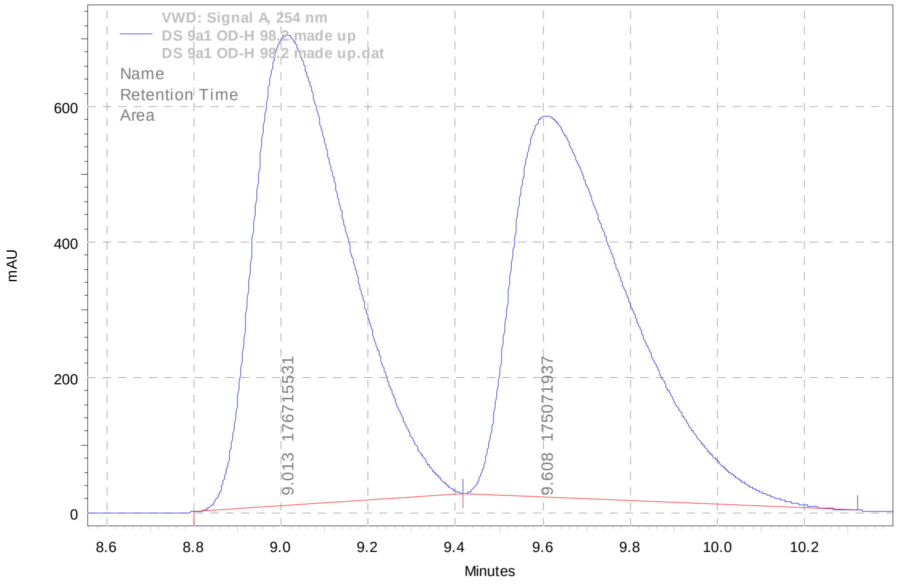

Retention Time	Area	Area %	Height	Height %
9.013	176715531	50.23	11669546	55.25
9.608	175071937	49.77	9450870	44.75
Totals	351787468	100.00	21120416	100.00

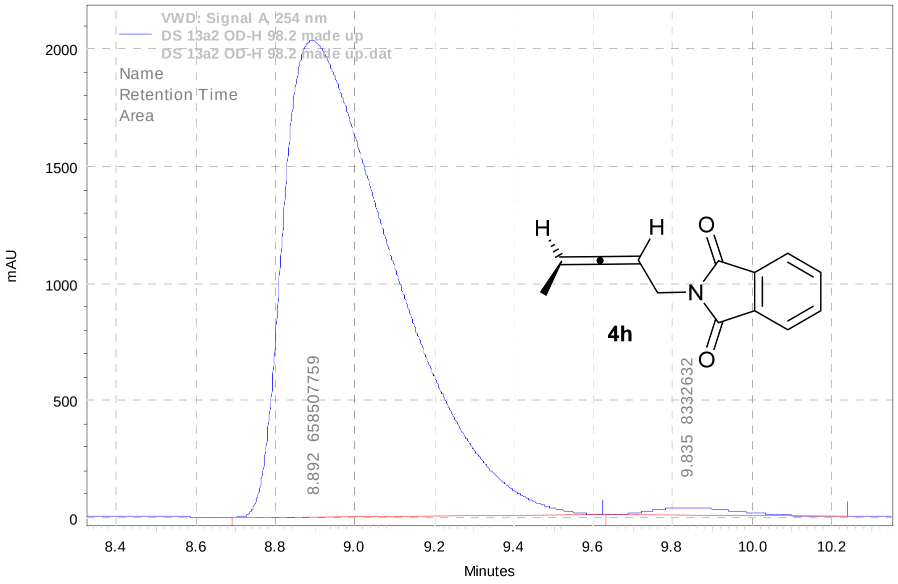

Retention Time	Area	Area %	Height	Height %
8.892	658507759	98.75	34119467	98.44
9.835	8332632	1.25	540803	1.56
Totals	666840391	100.00	34660270	100.00

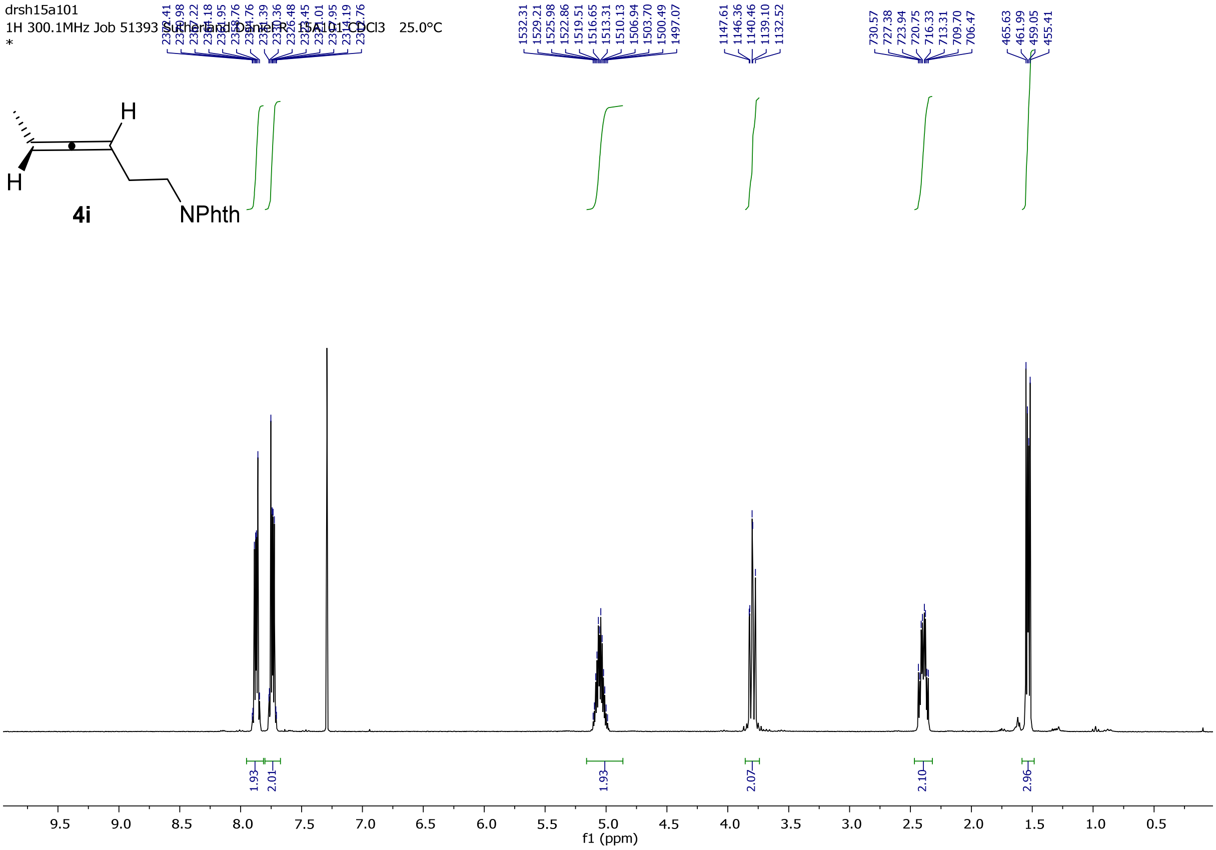
drsh15a101
1H 300.1MHz Job 51393 Sutherland Daniel R 15A101 CDCl3 25.0°C
*
H
H
4i
NPhth
1.93
2.01
1.93
2.07
2.10
2.96
f1 (ppm)

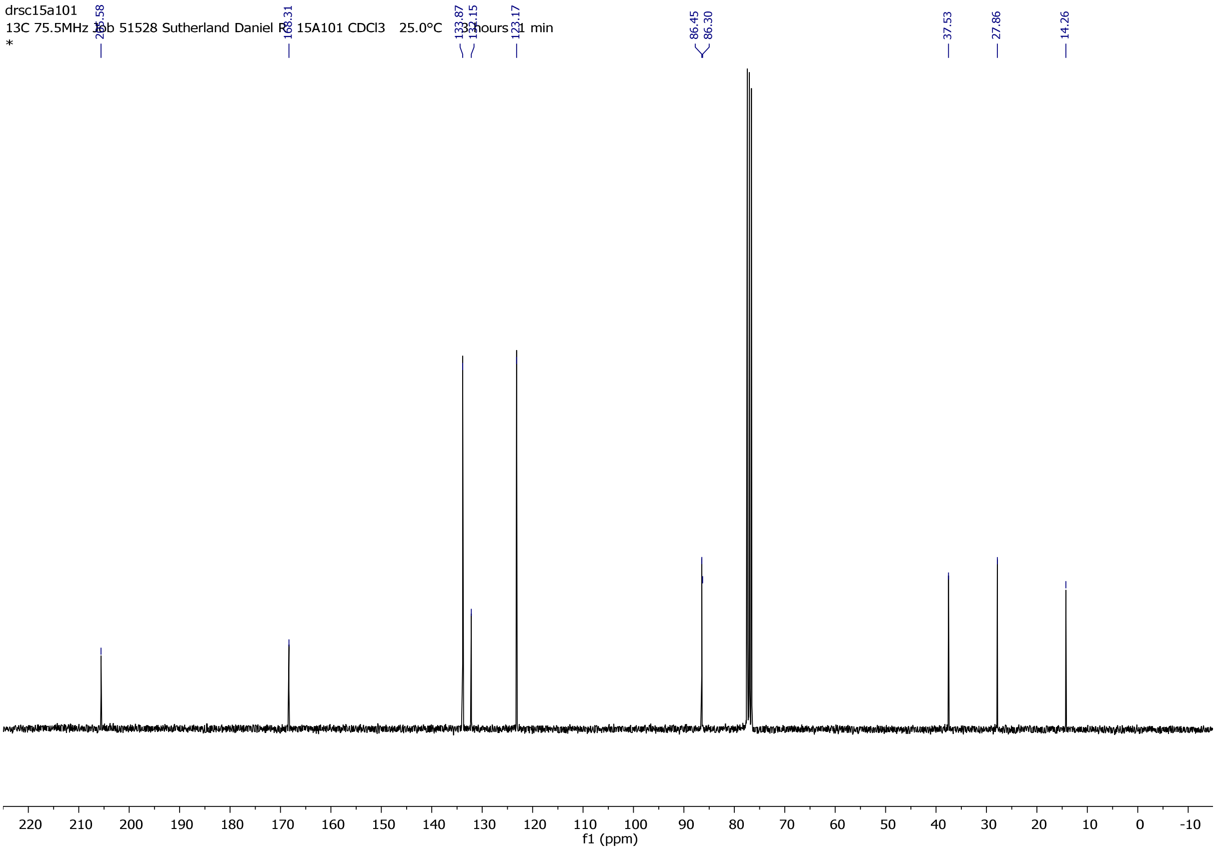
drsc15a101
13C 75.5MHz Job 51528 Sutherland Daniel R 15A101 CDCl3 25.0°C
*
205.58
168.31
133.87
132.15
123.17
86.45
86.30
37.53
27.86
14.26
f1 (ppm)

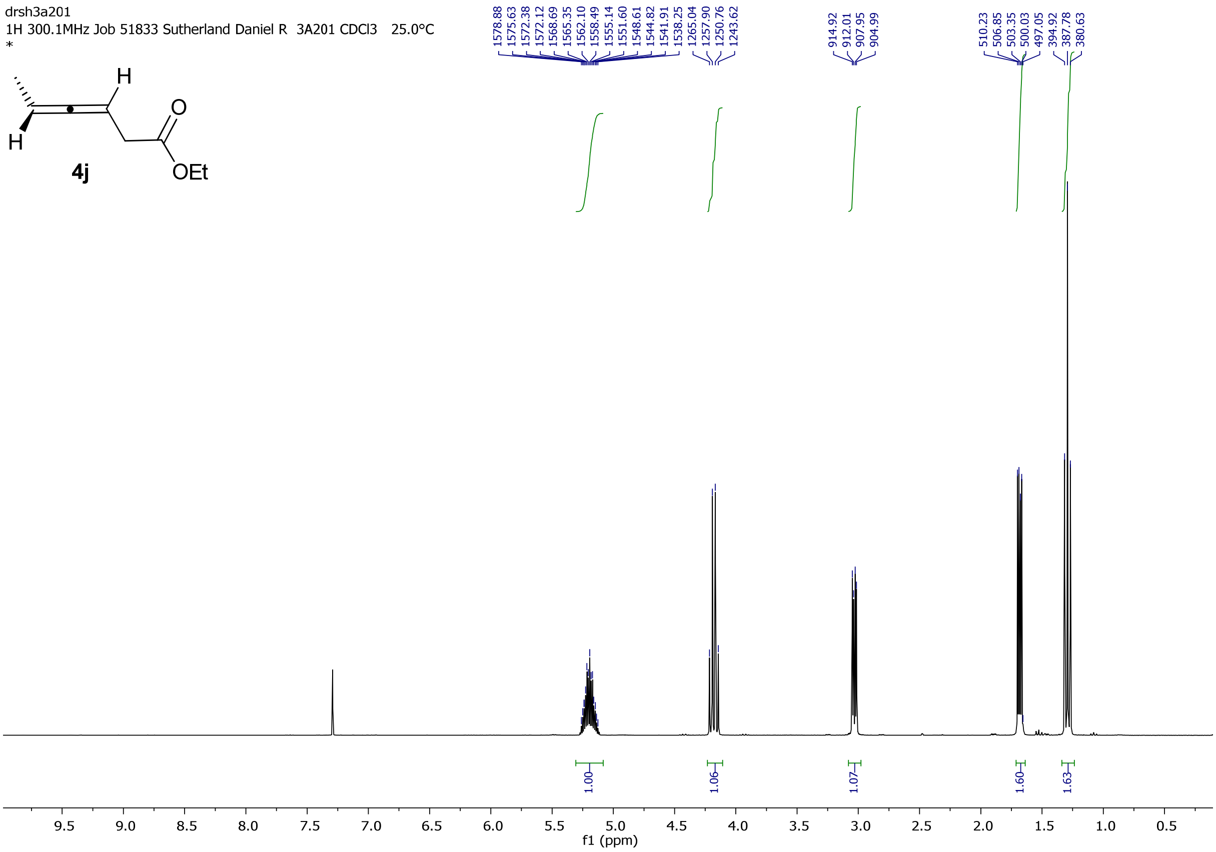
drsh3a201
1H 300.1MHz Job 51833 Sutherland Daniel R 3A201 CDCl3 25.0°C
*
1578.88
1575.63
1572.38
1572.12
1568.69
1565.35
1562.10
1558.49
1555.14
1551.60
1548.61
1544.82
1541.91
1538.25
1265.04
1257.90
1250.76
1243.62
914.92
912.01
907.95
904.99
510.23
506.85
503.35
500.03
497.05
394.92
387.78
380.63
H
O
H
4j
OEt
1.00
1.06
1.07
1.60
1.63
9.5
9.0
8.5
8.0
7.5
7.0
6.5
6.0
5.5
5.0
4.5
4.0
3.5
3.0
2.5
2.0
1.5
1.0
0.5
f1 (ppm)

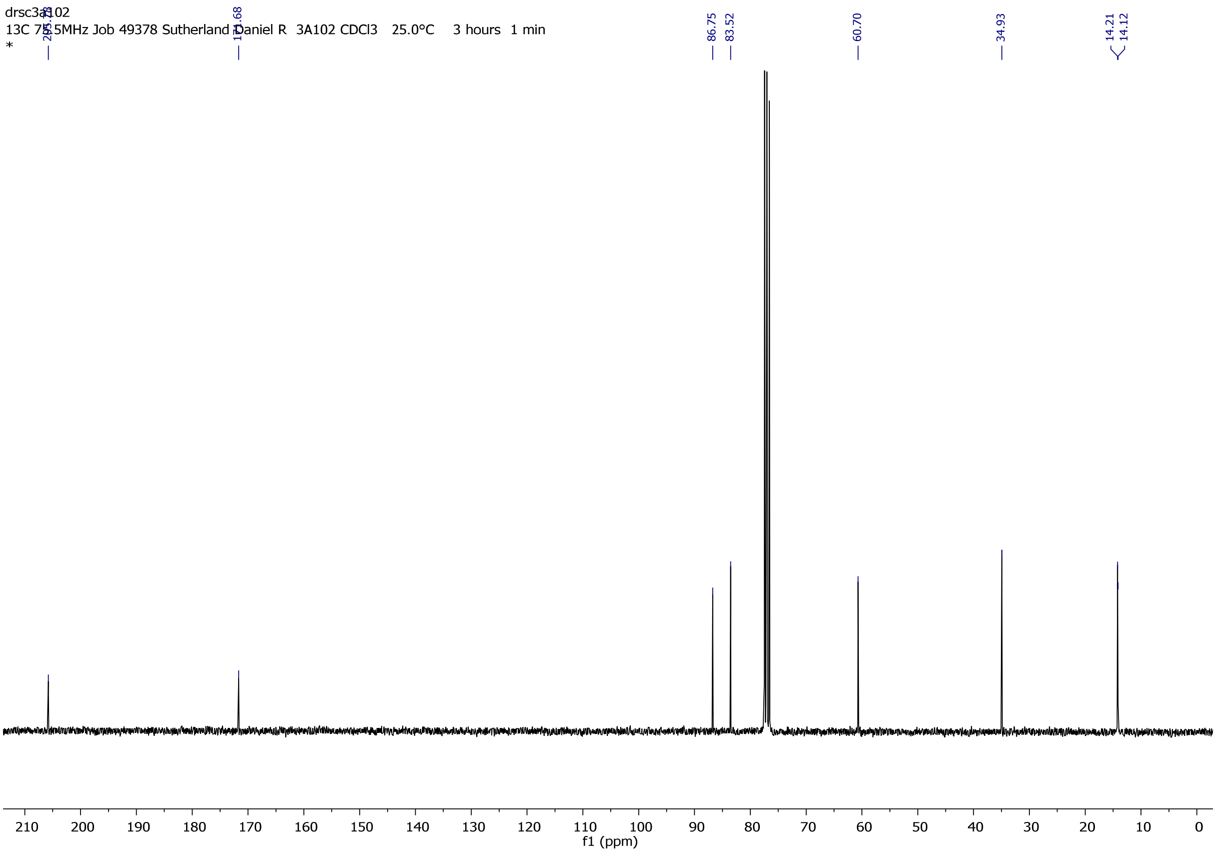
drsc3a102
13C 75.5MHz Job 49378 Sutherland Daniel R 3A102 CDCl3 25.0°C 3 hours 1 min
*
171.68
86.75
83.52
60.70
34.93
14.21
14.12
210
200
190
180
170
160
150
140
130
120
110
100
90
80
70
60
50
40
30
20
10
0
f1 (ppm)

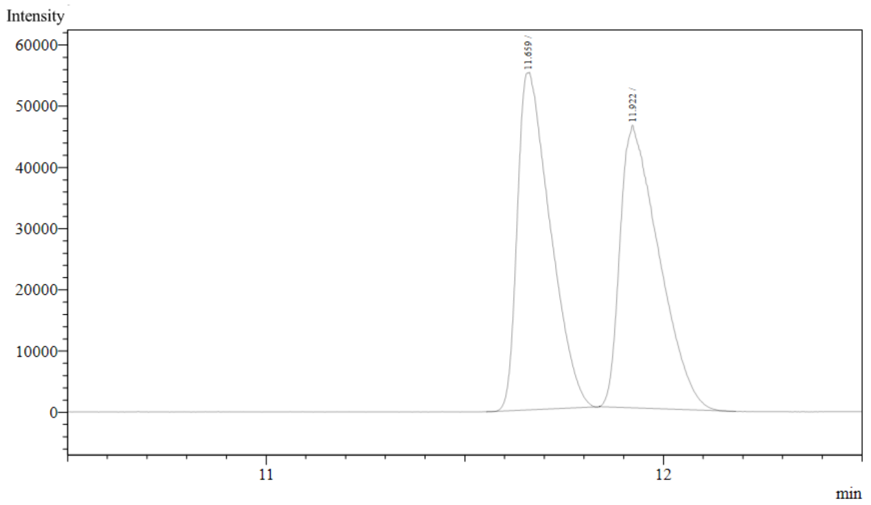

Peak#	Ret.Time	Area	Height	Conc.	Unit	Mark	ID#	Cmpd Name	Area%
1	11.659	316895	55101	50.160					50.1603
2	11.922	314870	46066	49.840					49.8397
**Total**		631765	101167						100.0000

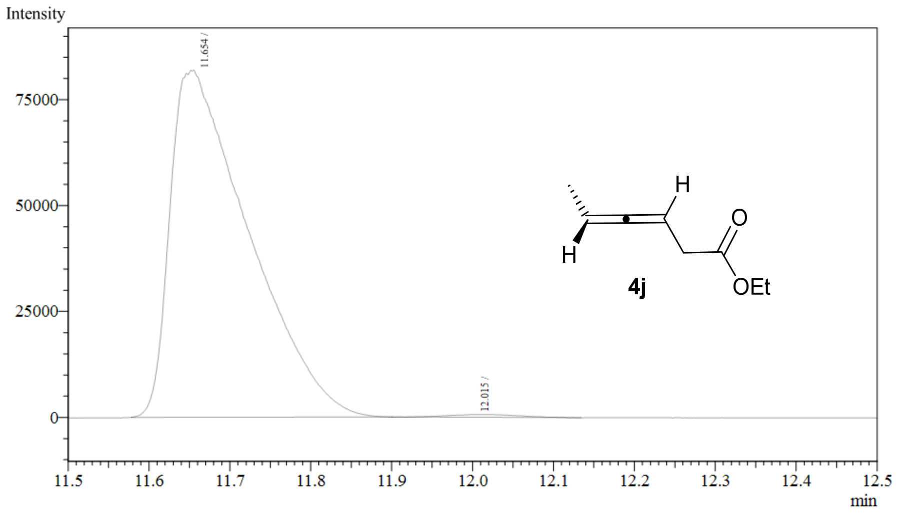

Peak#	Ret.Time	Area	Height	Conc.	Unit	Mark	ID#	Cmpd Name	Area%
1	11.654	555280	81625	99.298					99.2984
2	12.015	3923	653	0.702					0.7016
**Total**		559203	82278						100.0000

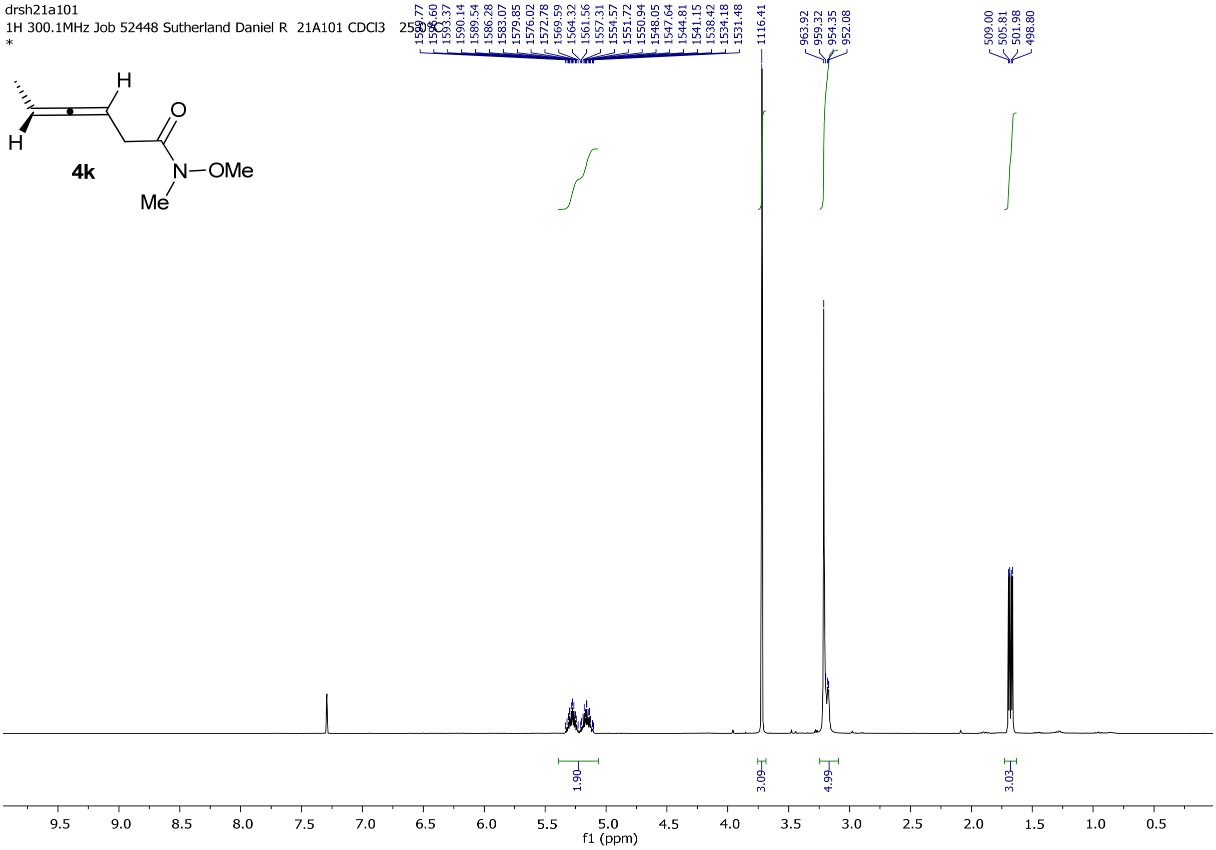
drsh21a101
1H 300.1MHz Job 52448 Sutherland Daniel R 21A101 CDCl3 25.0°C
*
4k
N—OMe
Me
1.90
3.09
4.99
3.03
f1 (ppm)

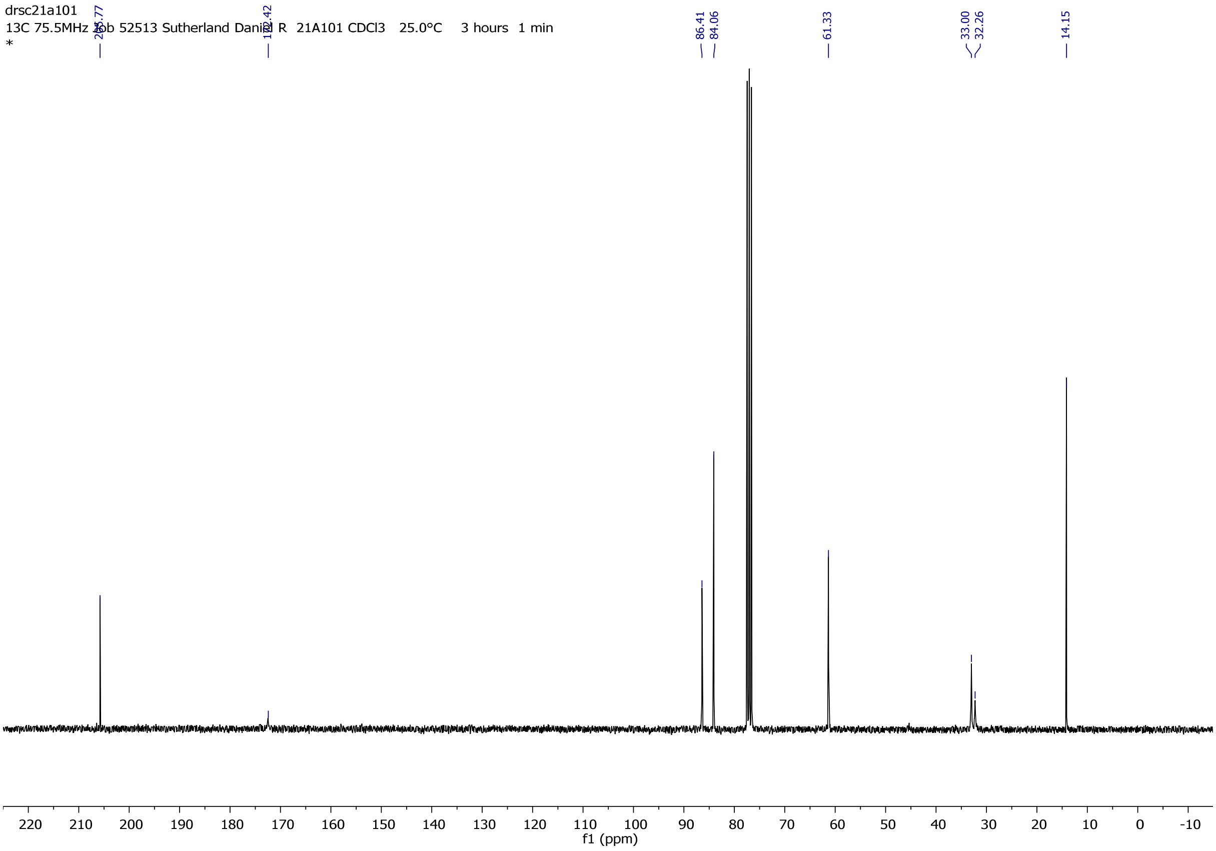
drsc21a101
13C 75.5MHz Job 52513 Sutherland Daniel R 21A101 CDCl3 25.0°C 3 hours 1 min
*
86.41
84.06
61.33
33.00
32.26
14.15
f1 (ppm)

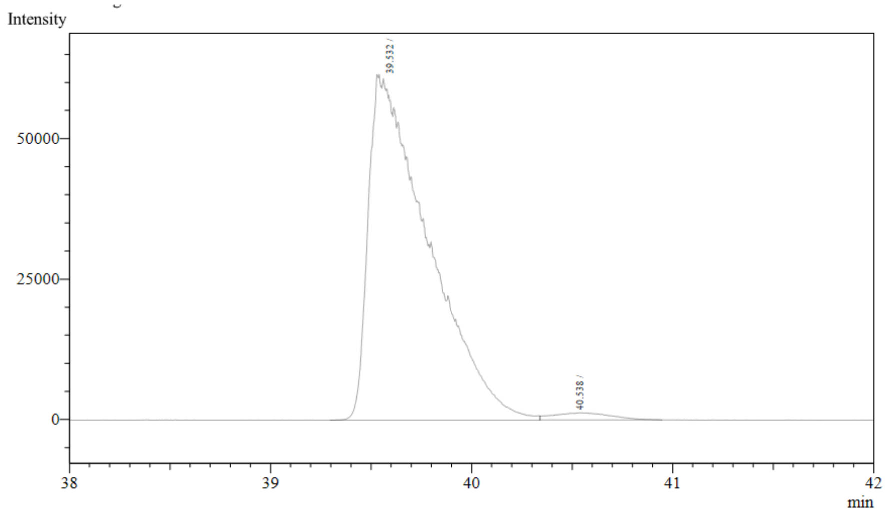

Peak#	Ret.Time	Area	Height	Conc.	Unit	Mark	ID#	Cmpd Name	Area%
1	39.532	1272673	61329	98.028					98.0282
2	40.538	25599	1276	1.972		V			1.9718
**Total**		1298272	62605						100.0000

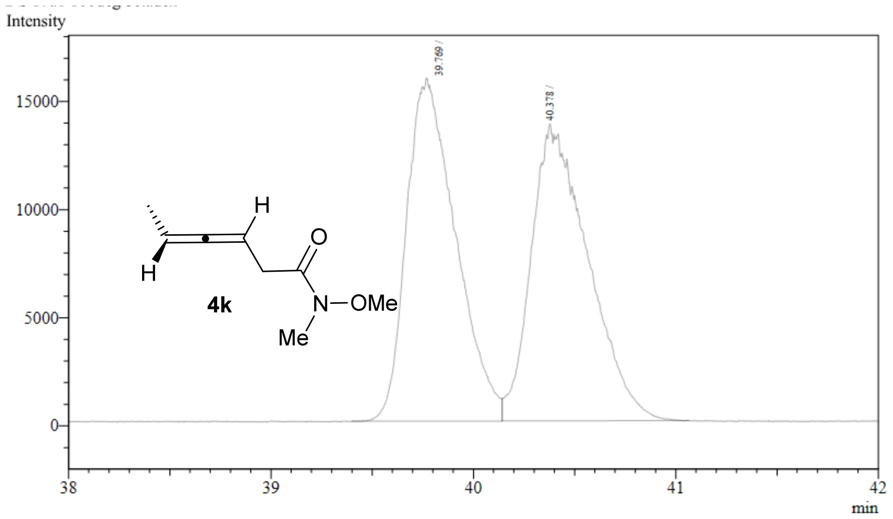

Peak#	Ret.Time	Area	Height	Conc.	Unit	Mark	ID#	Cmpd Name	Area%
1	39.769	267310	15833	49.941					49.9408
2	40.378	267944	13710	50.059		V			50.0592
**Total**		535254	29543						100.0000

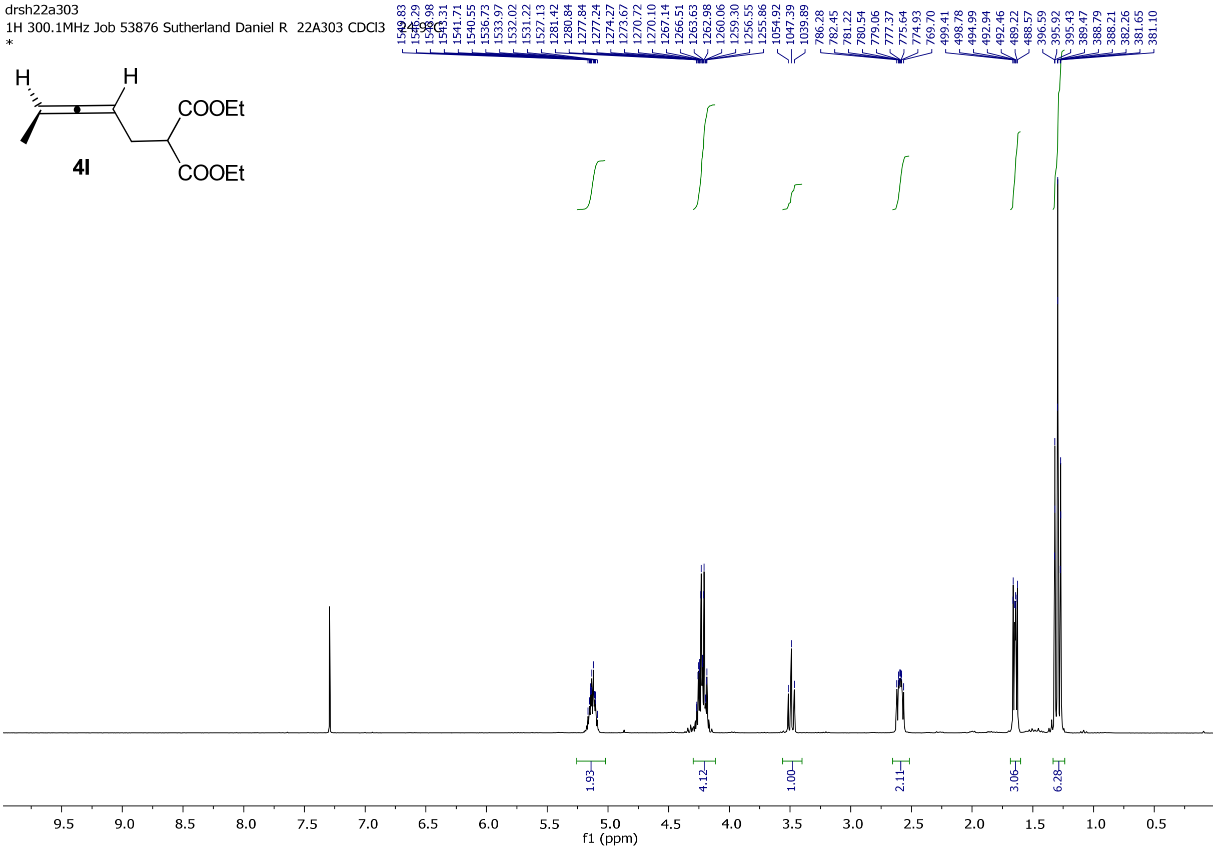
drsh22a303
1H 300.1MHz Job 53876 Sutherland Daniel R 22A303 CDCl3 24.9°C
COOEt
COOEt
4l
1.93
4.12
1.00
2.11
3.06
6.28
f1 (ppm)

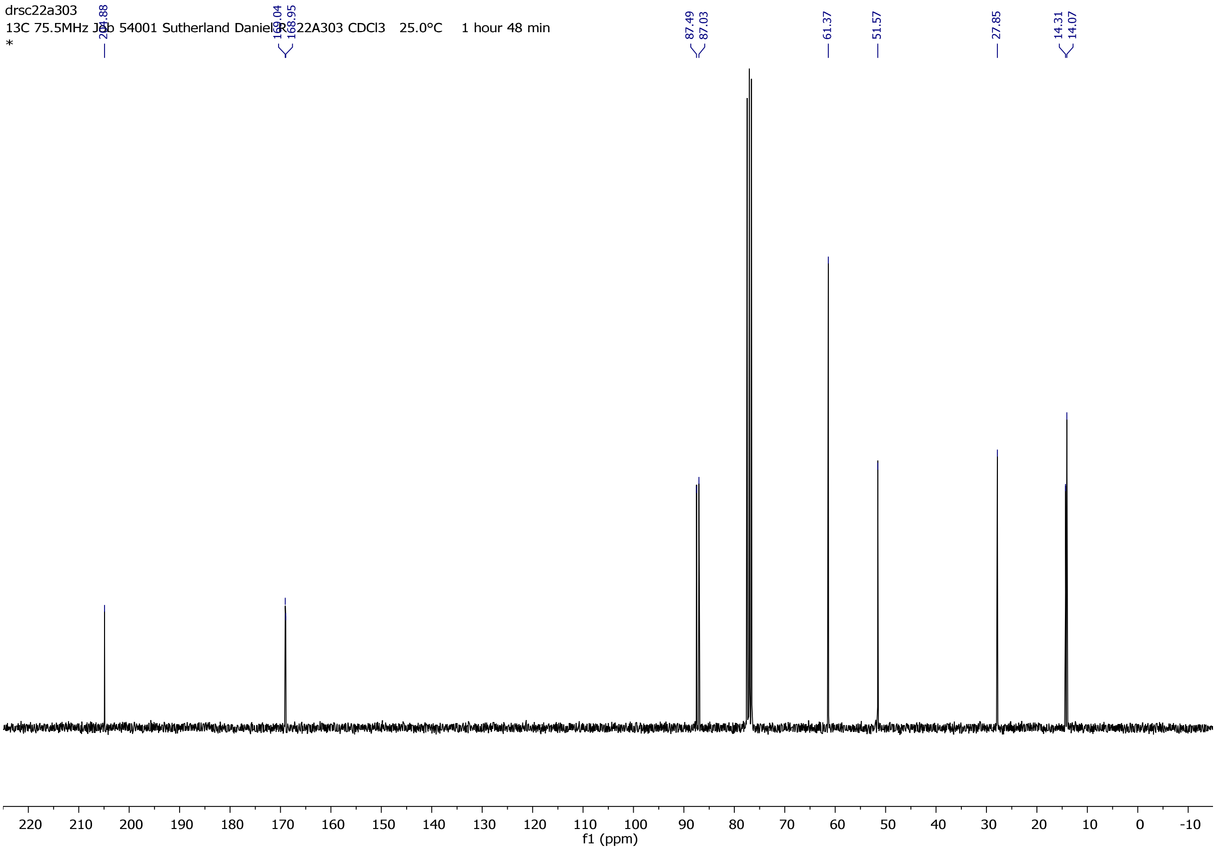
drsc22a303
13C 75.5MHz Job 54001 Sutherland Daniel R 22A303 CDCl3 25.0°C 1 hour 48 min
87.49
87.03
61.37
51.57
27.85
14.31
14.07
f1 (ppm)

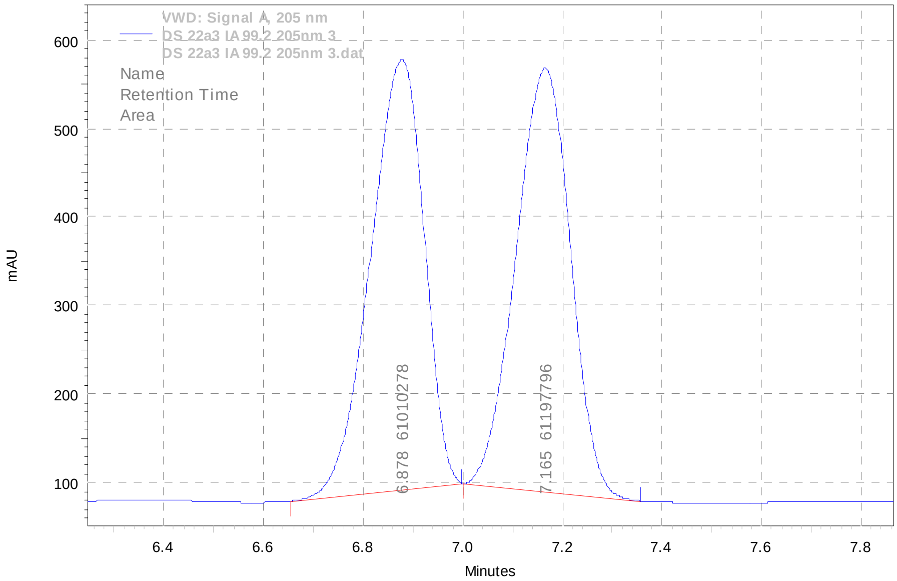

Retention Time	Area	Area %	Height	Height %
6.878	61010278	49.92	8137821	50.37
7.165	61197796	50.08	8017173	49.63
Totals	122208074	100.00	16154994	100.00

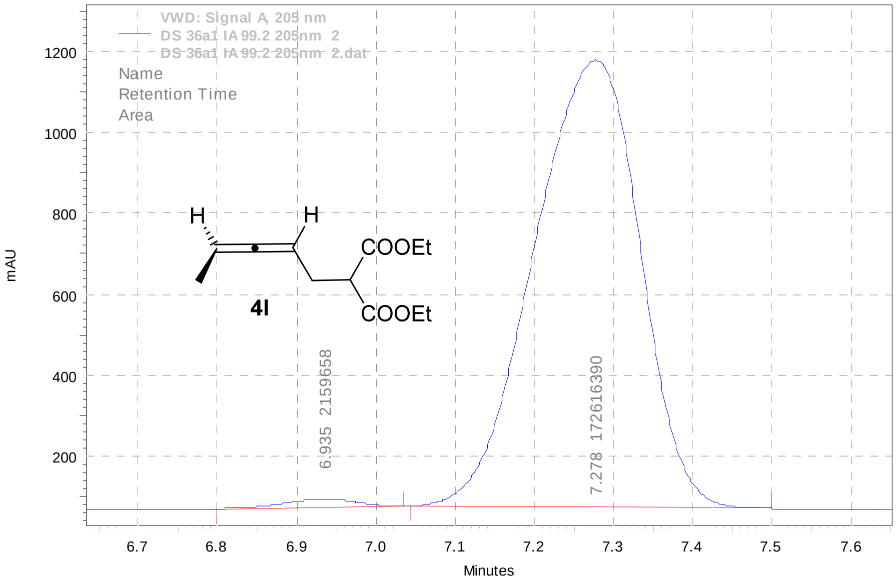

Retention Time	Area	Area %	Height	Height %
6.935	2159658	1.24	347042	1.84
7.278	172616390	98.76	18531559	98.16
Totals	174776048	100.00	18878601	100.00

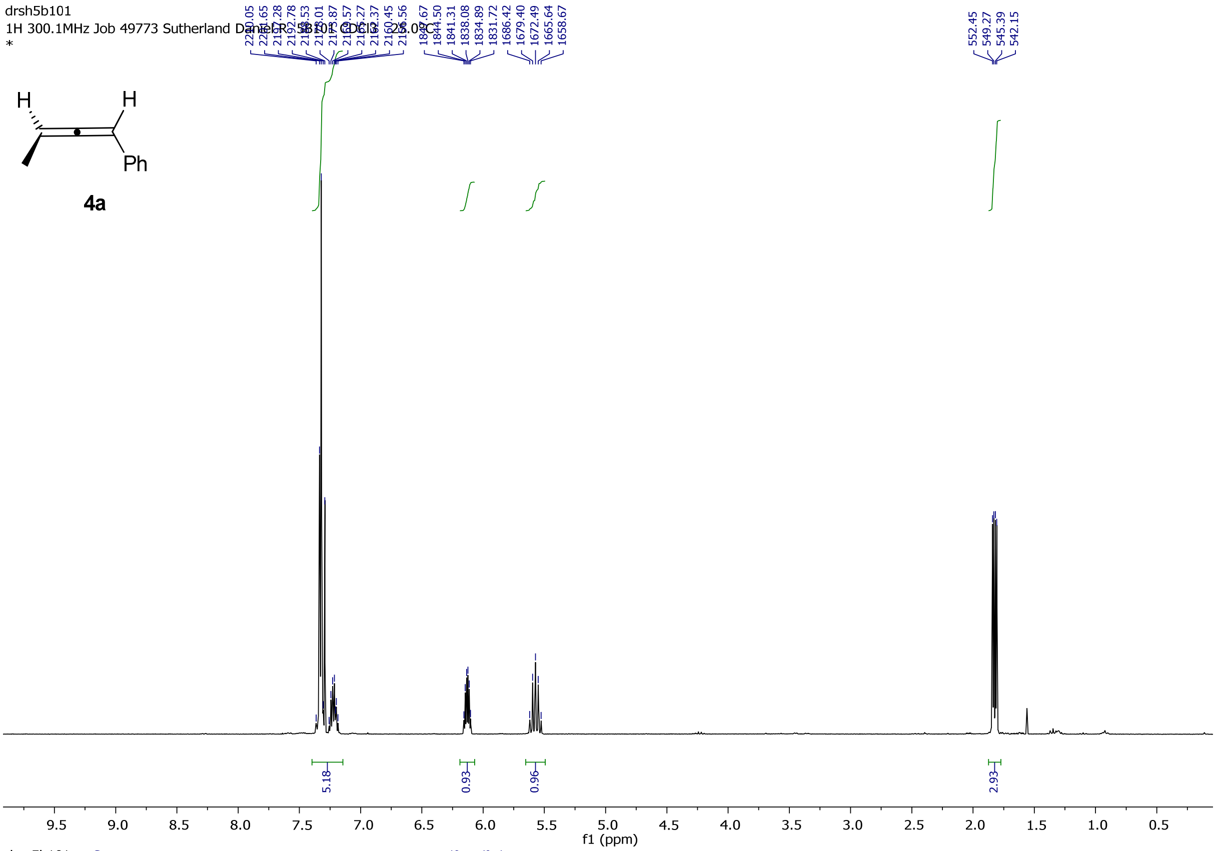

4a

drsh5b101
*
1841.31
1838.08
1834.89
1831.72
1686.42
1679.40
1672.49
1665.64
1658.67
552.45
549.27
545.39
542.15
5.18
0.93
0.96
2.93
9.5 9.0 8.5 8.0 7.5 7.0 6.5 6.0 5.5 5.0 4.5 4.0 3.5 3.0 2.5 2.0 1.5 1.0 0.5
f1 (ppm)

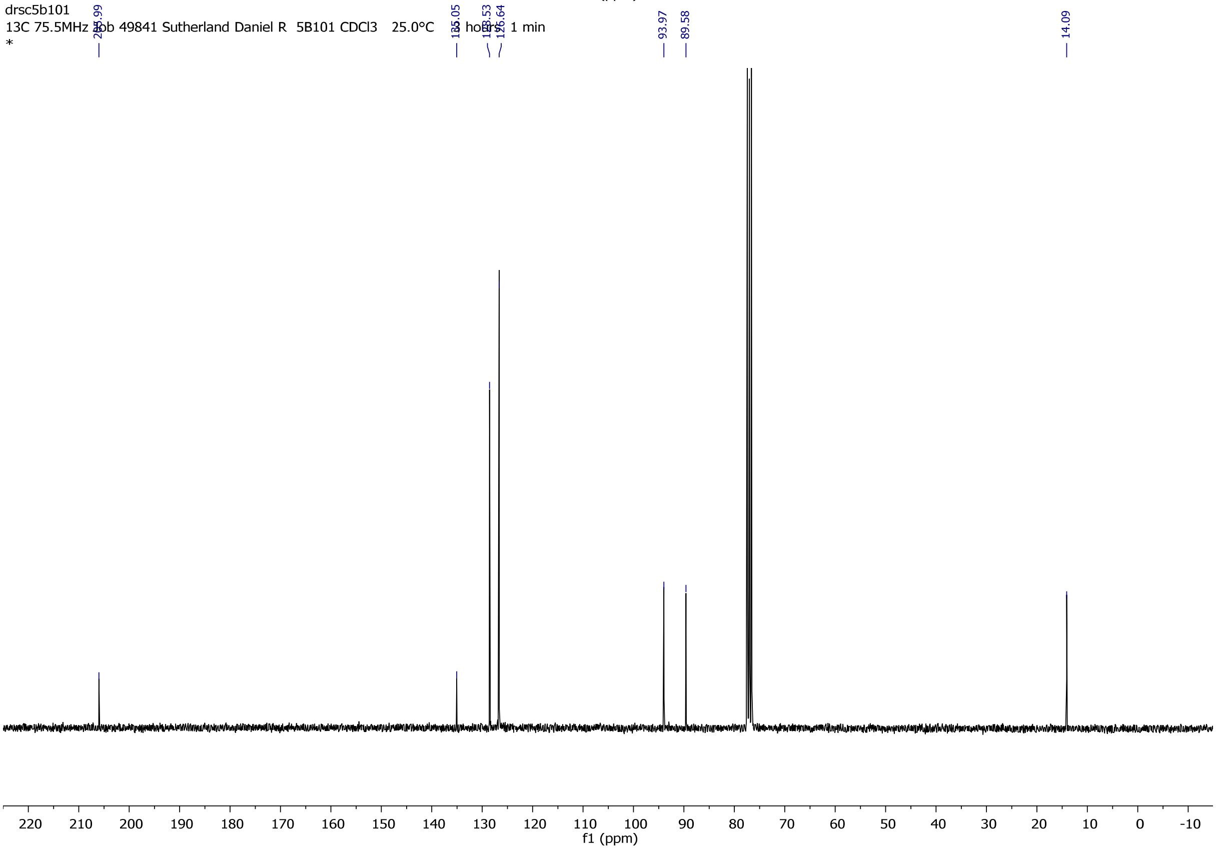
drsc5b101
*
135.05
93.97
89.58
14.09
220 210 200 190 180 170 160 150 140 130 120 110 100 90 80 70 60 50 40 30 20 10 0 -10
f1 (ppm)

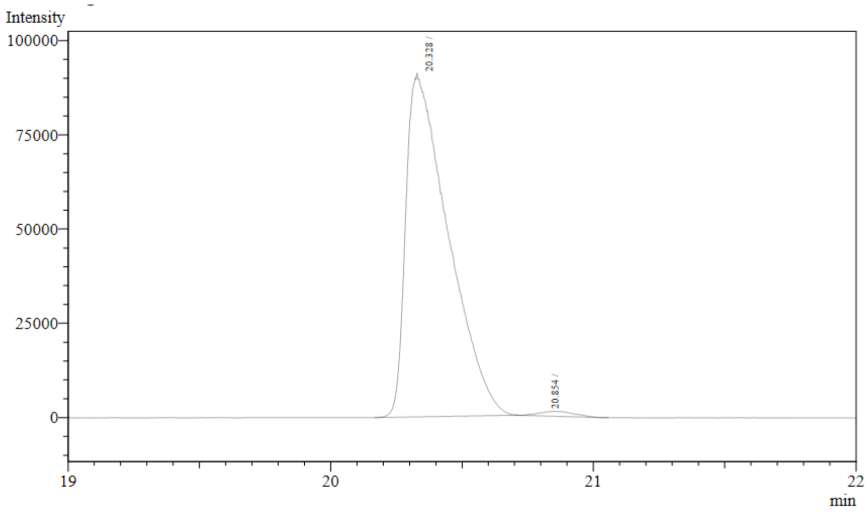

Peak#	Ret.Time	Area	Height	Conc.	Unit	Mark	ID#	Cmpd Name	Area%
1	20.328	1020271	90403	98.831					98.8311
2	20.854	12067	1276	1.169					1.1689
**Total**		1032338	91679						100.0000

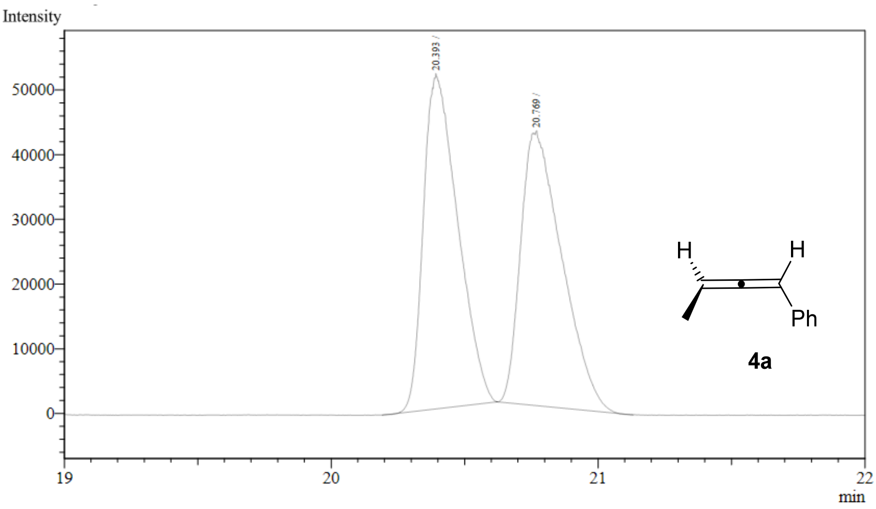

Peak#	Ret.Time	Area	Height	Conc.	Unit	Mark	ID#	Cmpd Name	Area%
1	20.393	462778	51539	50.351					50.3511
2	20.769	456325	42380	49.649					49.6489
**Total**		919103	93919						100.0000

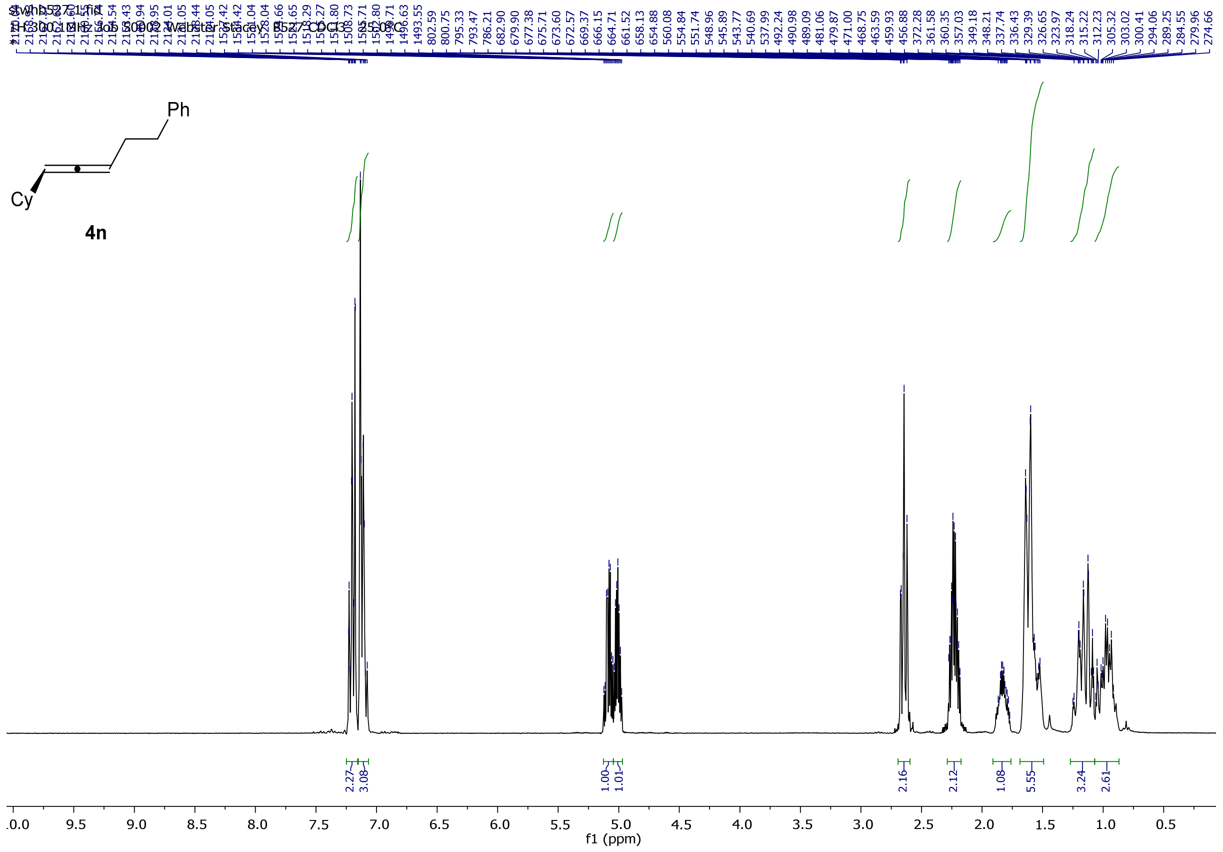
Ph
Cy
4n
f1 (ppm)

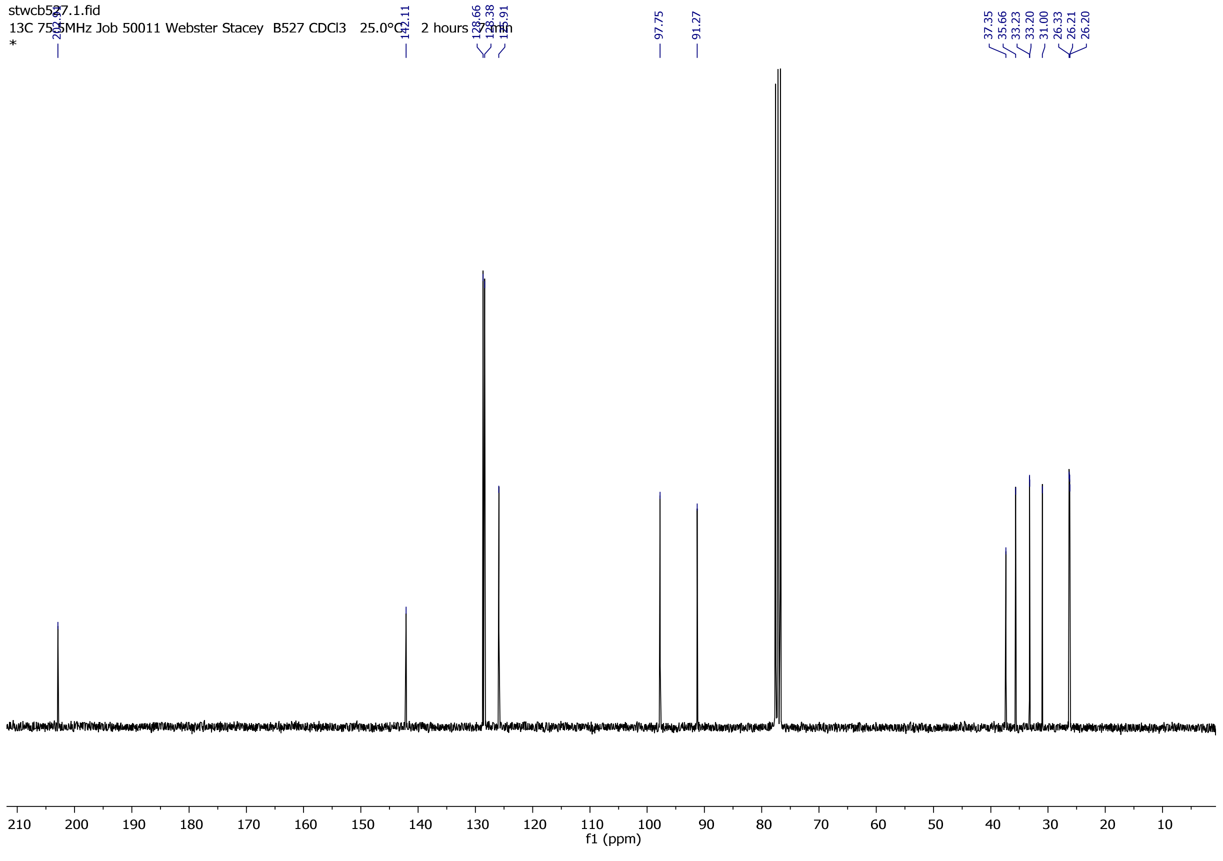
stwcb527.1.fid
13C 75.5MHz Job 50011 Webster Stacey B527 CDCl3 25.0°C 2 hours 7 min
142.11
128.66
128.38
125.91
97.75
91.27
37.35
35.66
33.23
33.20
31.00
26.33
26.21
26.20
f1 (ppm)

stwhb628.1.fid
1H 300.1MHz Job 54258 Webster Stacey B628 CDCl3 24.9°C
*

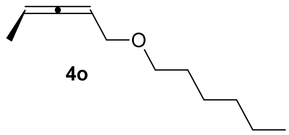

4o

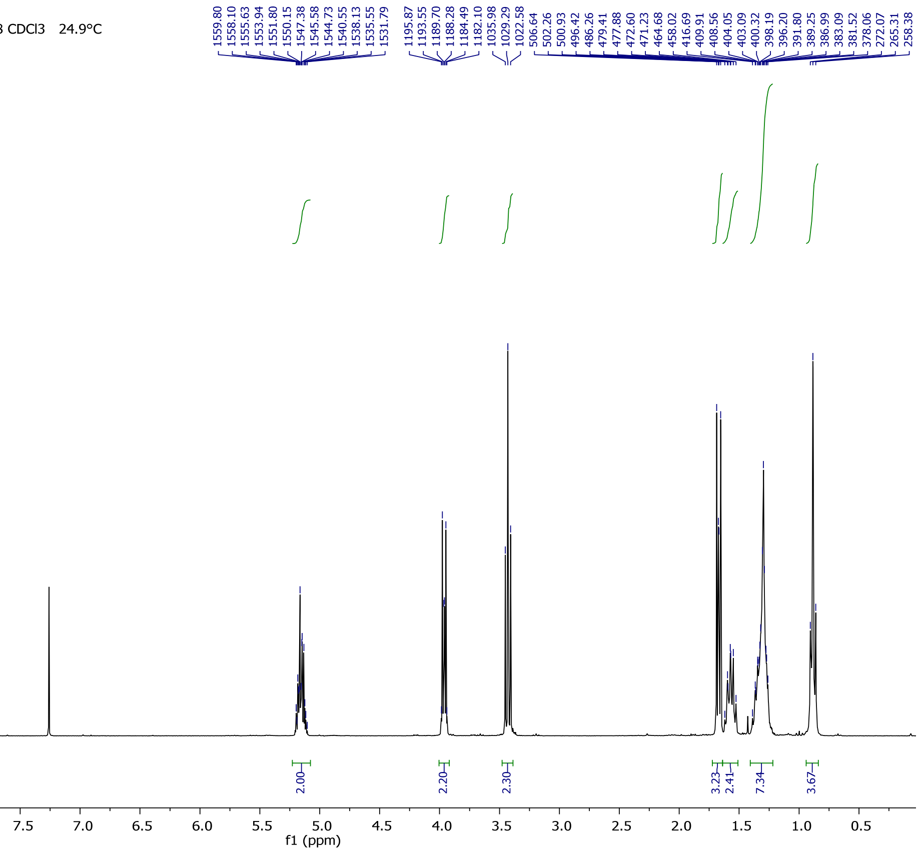

stwcb628.1.fid
13C 75.5MHz Job 54319 Webster Stacey B628 CDCl3 25.0°C 2 hours 7 min
*

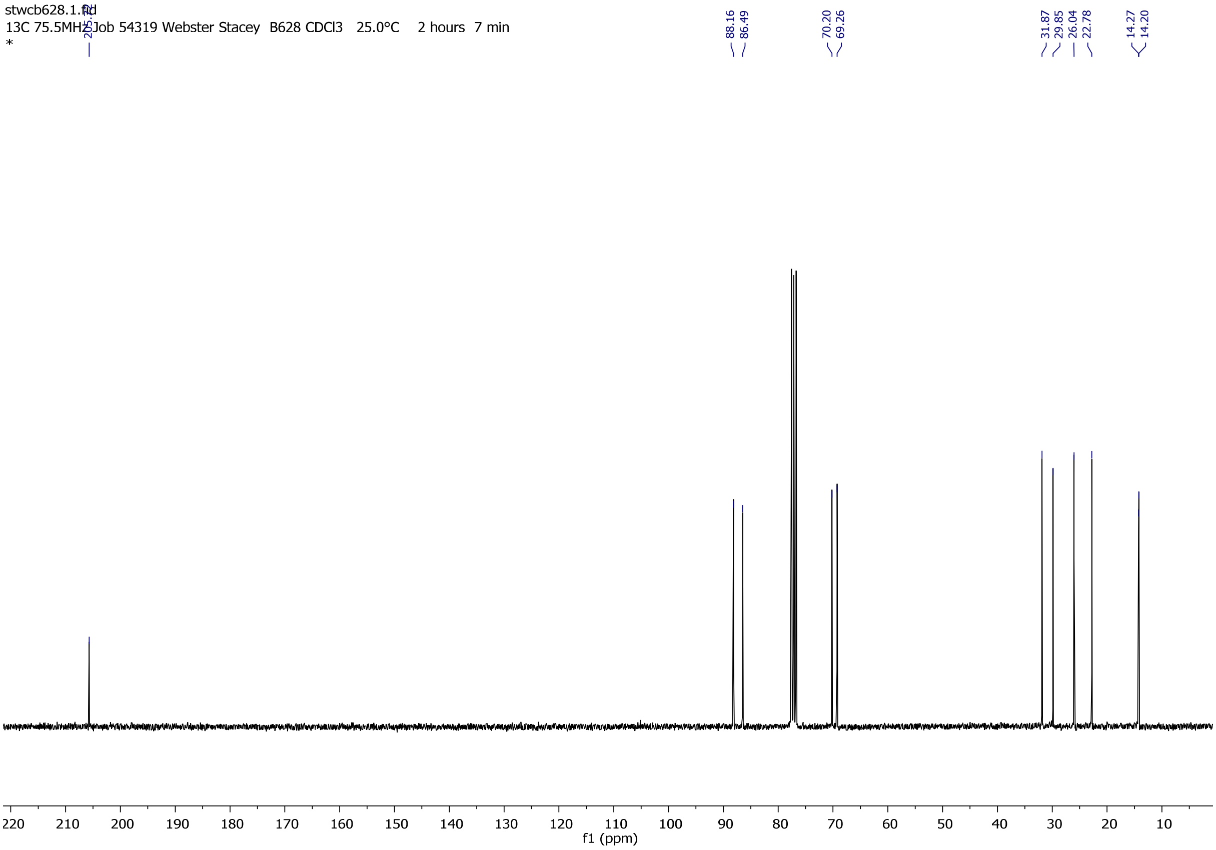

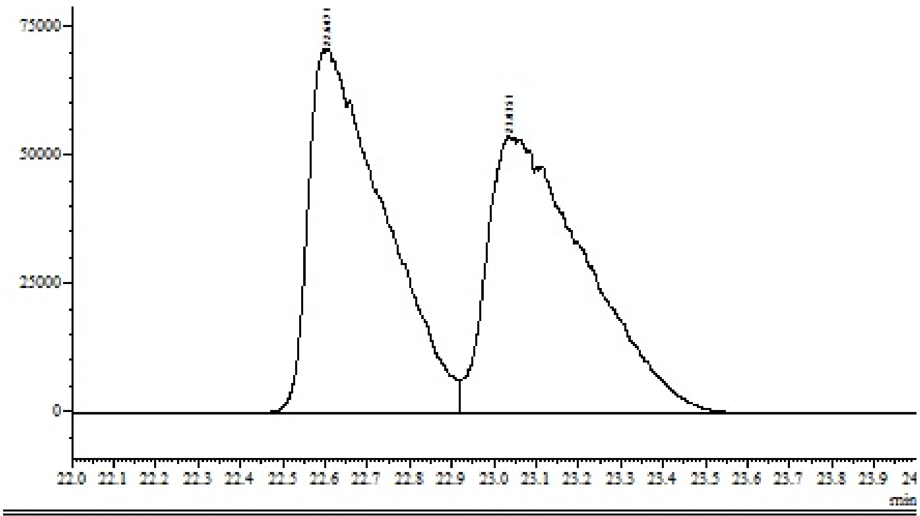

Peak#	Ret.Time	Area	Height	Conc.	Unit	Mark	ID#	Cmpd Name	Area%
1	22.607	881713	70771	50.138					50.1382
2	23.035	876852	53954	49.862		V			49.8618
Total		1758565	124725						100.0000

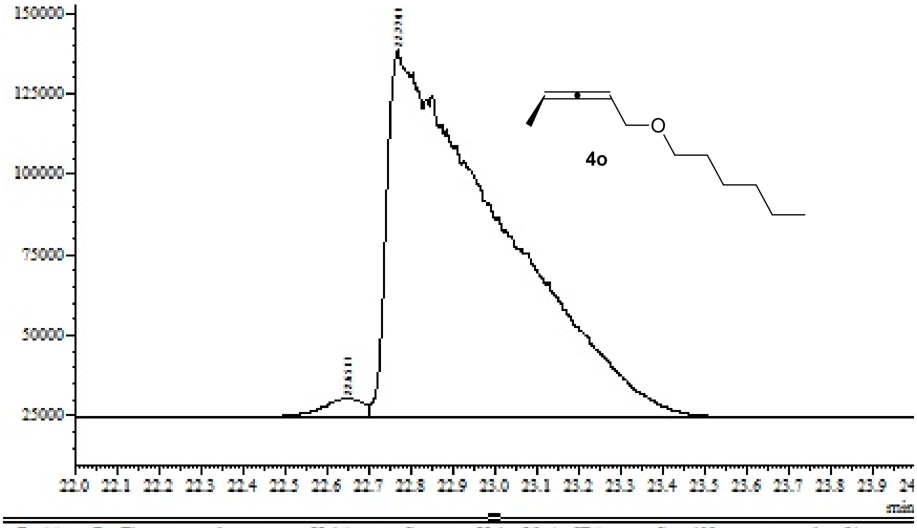

Peak#	Ret.Time	Area	Height	Conc.	Unit	Mark	ID#	Cmpd Name	Area%
1	22.651	39797	5926	1.834					1.8343
2	22.770	2129851	112641	98.166		V			98.1657
Total		2169648	118567						100.0000

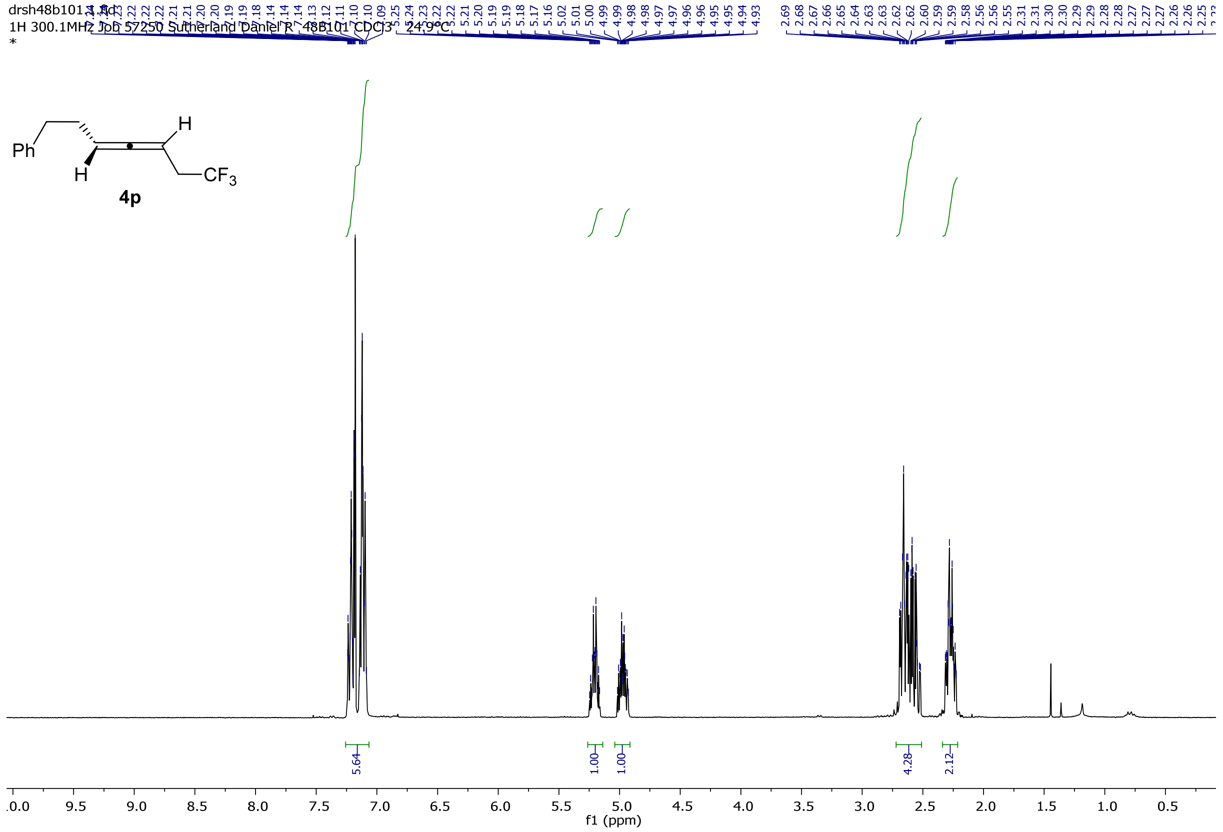
drsh48b101.1.fid
1H 300.1MHz Job 57250 Sutherland Daniel R 48B101 CDCl3 24.9°C
*
Ph
H
H
CF3
4p
5.64
1.00
1.00
4.28
2.12
f1 (ppm)

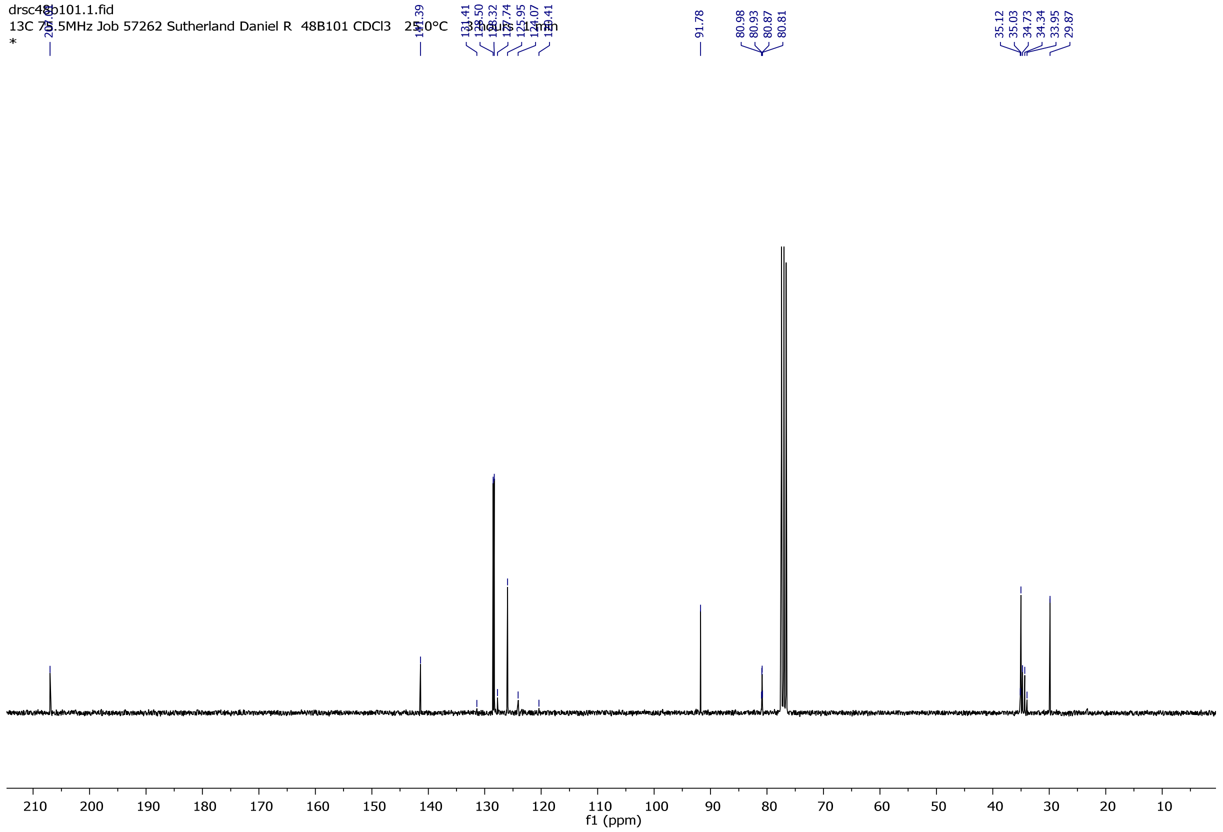
drsc48b101.1.fid
13C 75.5MHz Job 57262 Sutherland Daniel R 48B101 CDCl3 25.0°C
*
91.78
80.98
80.93
80.87
80.81
35.12
35.03
34.73
34.34
33.95
29.87
f1 (ppm)

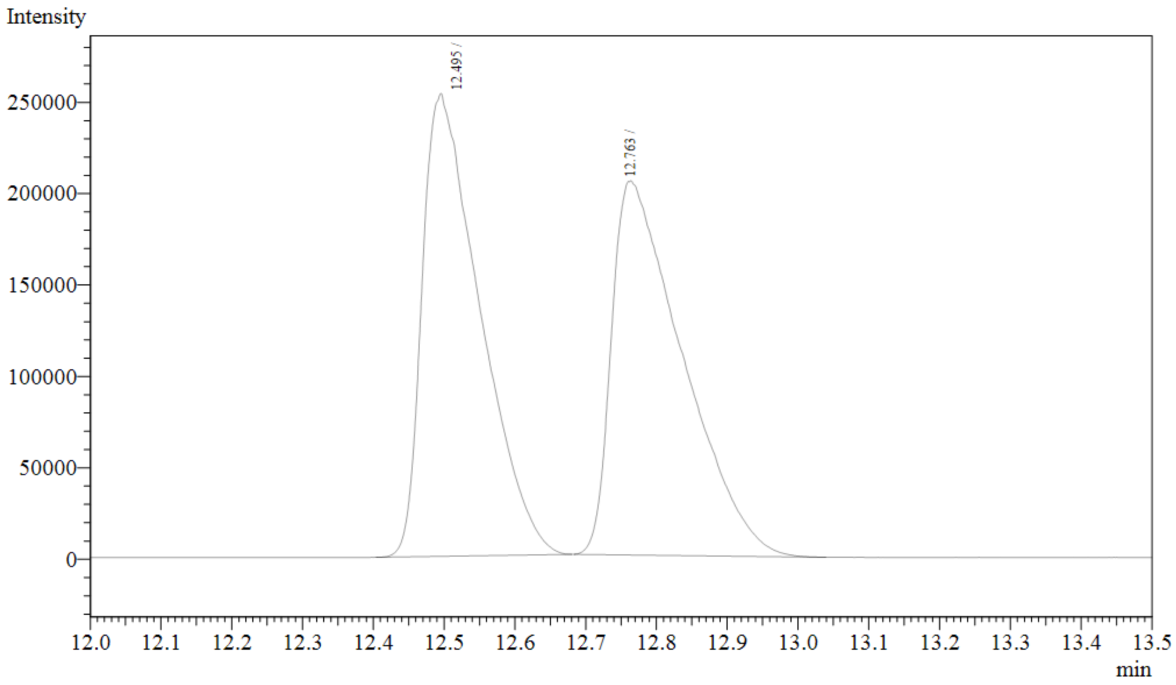

Peak#	Ret.Time	Area	Height	Conc.	Unit	Mark	ID#	Cmpd Name	Area%
1	12.495	1435841	252640	50.143					50.1432
2	12.763	1427640	204450	49.857					49.8568
**Total**		2863481	457090						100.0000

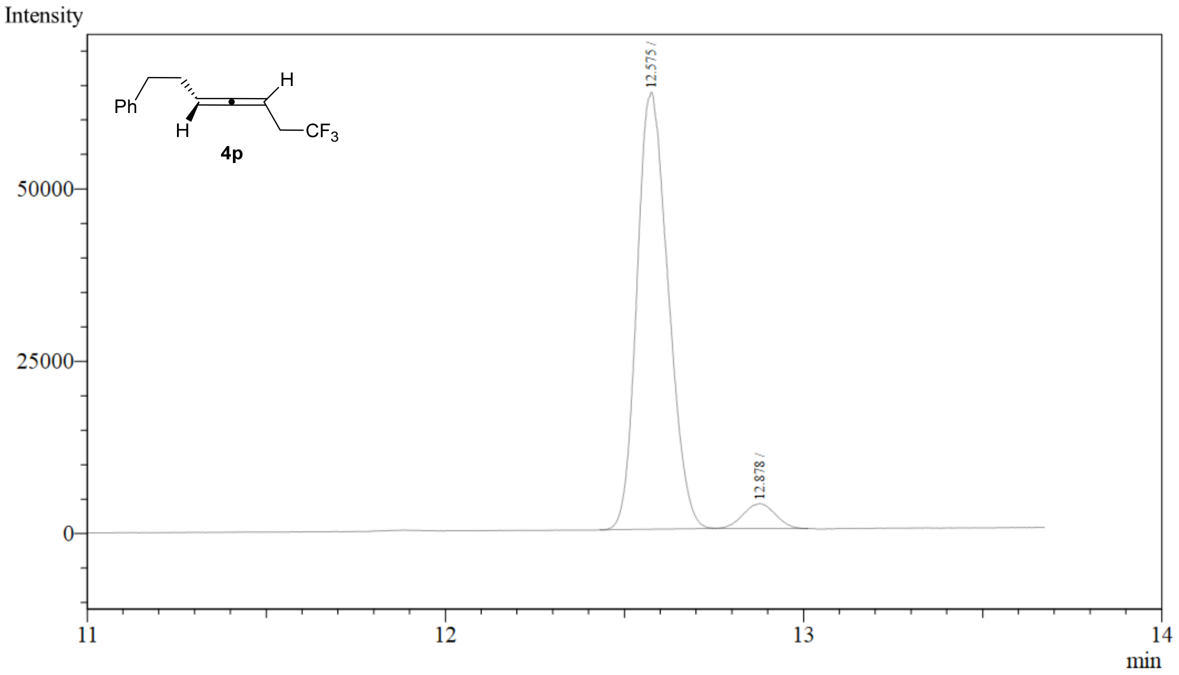

Peak#	Ret.Time	Area	Height	Conc.	Unit	Mark	ID#	Cmpd Name	Area%
1	12.575	384125	63369	94.525					94.5251
2	12.878	22248	3588	5.475					5.4749
**Total**		406373	66957						100.0000

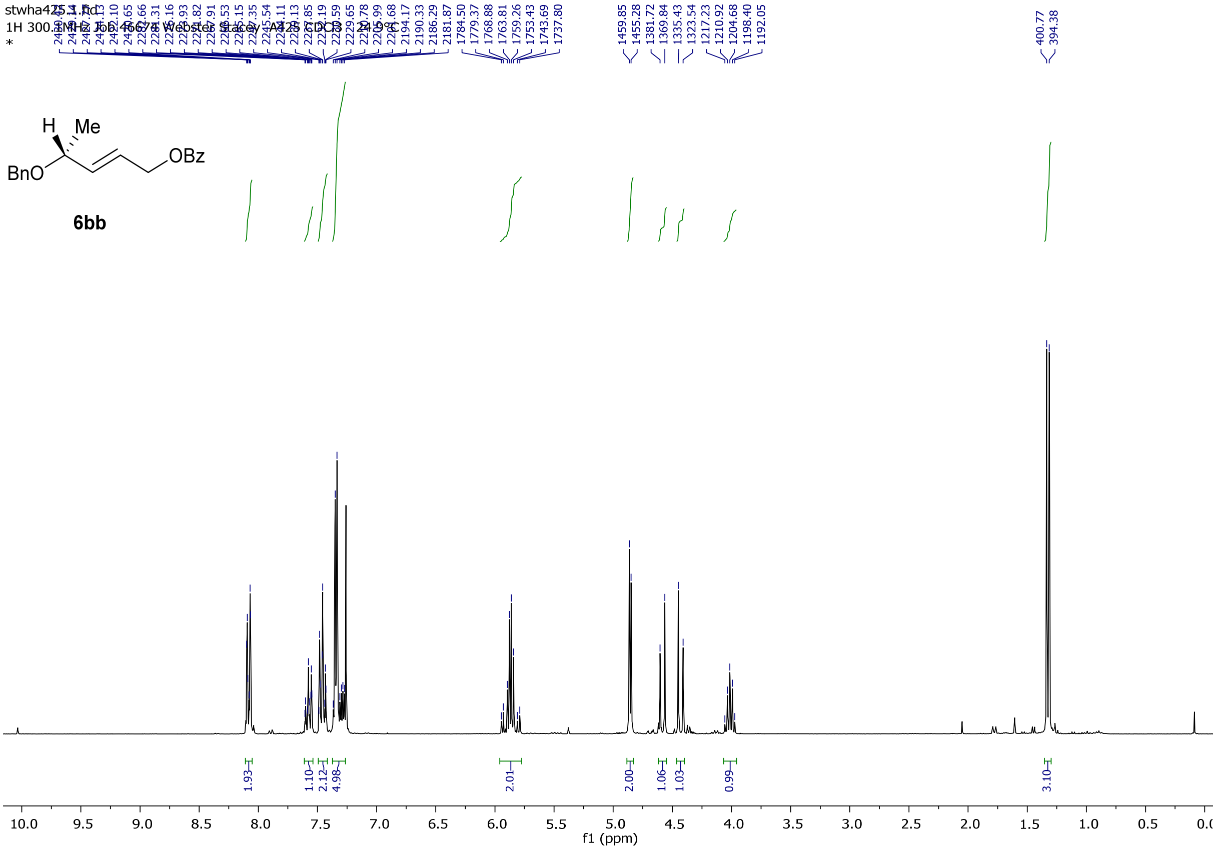
stwha425.1.fid
H Me
BnO
OBz
6bb
1.93
1.10
2.12
4.98
2.01
2.00
1.06
1.03
0.99
3.10
f1 (ppm)

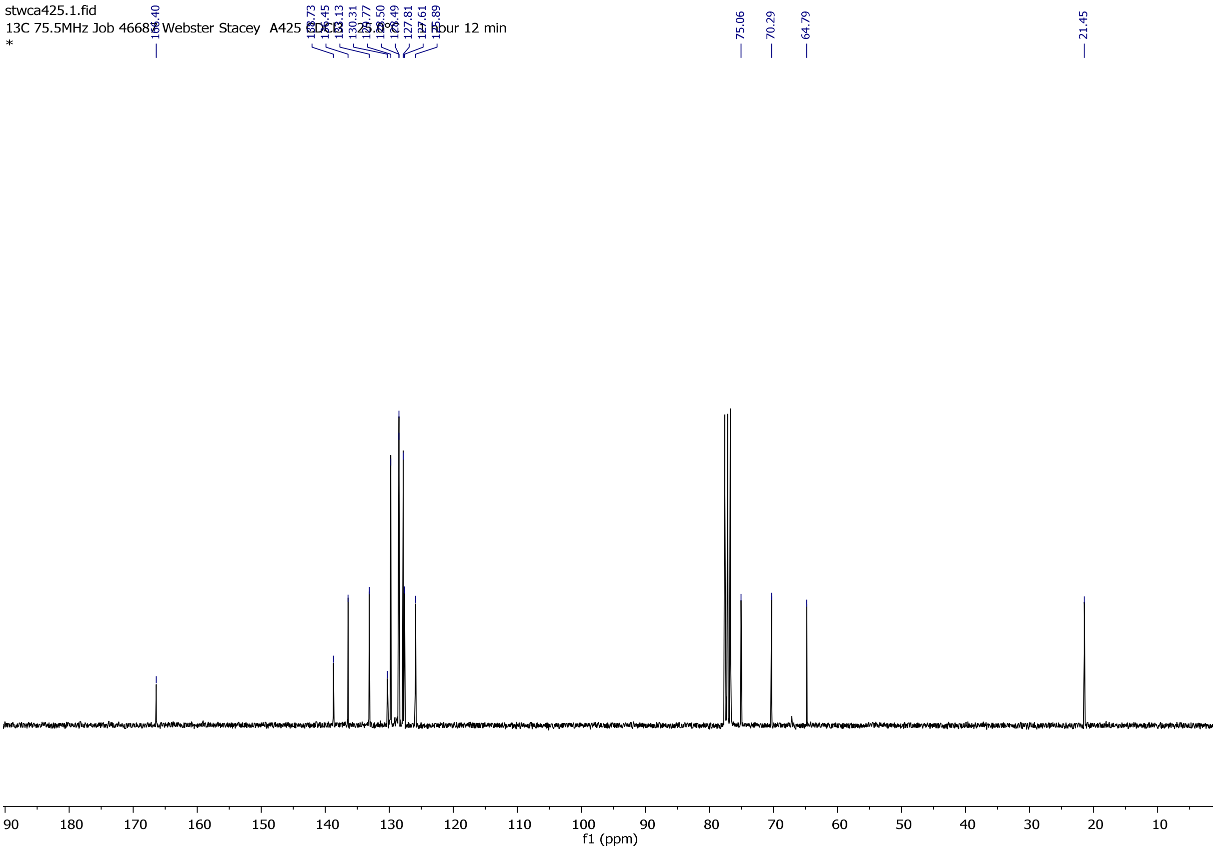
stwca425.1.fid
166.40
75.06
70.29
64.79
21.45
f1 (ppm)

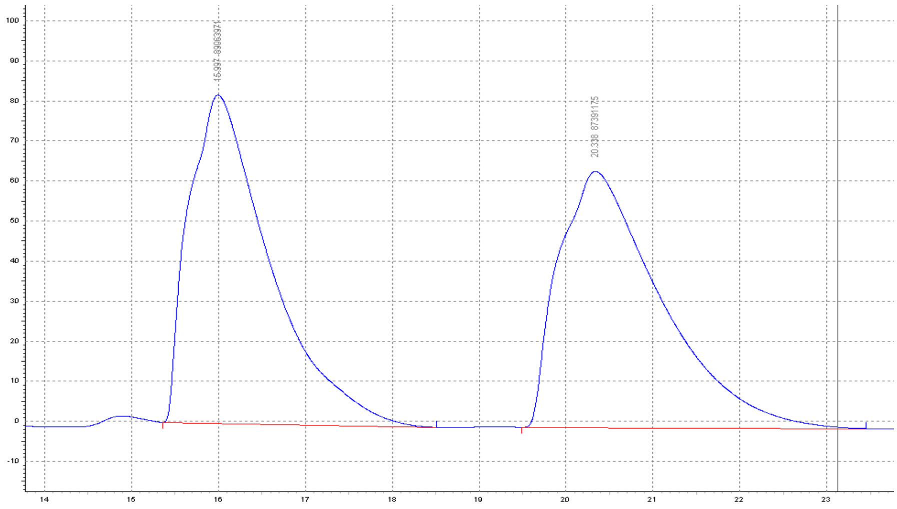

Retention Time	Area	Area %	Height	Height %
15.997	89063971	50.47	1376364	56.20
20.338	87391175	49.53	1072735	43.80
Totals	176455146	100.00	2449099	100.00

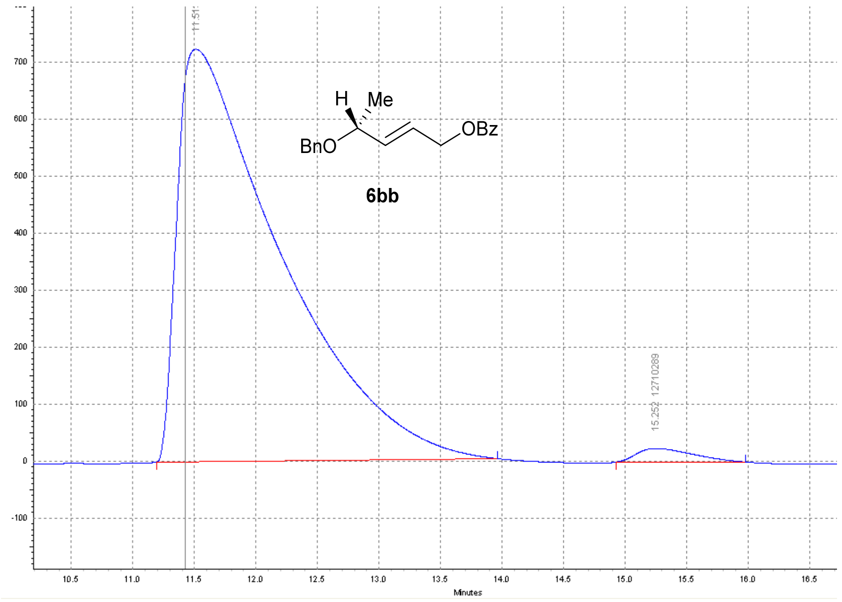

Retention Time	Area	Area %	Height	Height %
11.513	708497694	98.24	12145235	96.81
15.252	12710289	1.76	400583	3.19
Totals	721207983	100.00	12545818	100.00

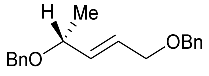
H
Me
BnO
OBn

6cb

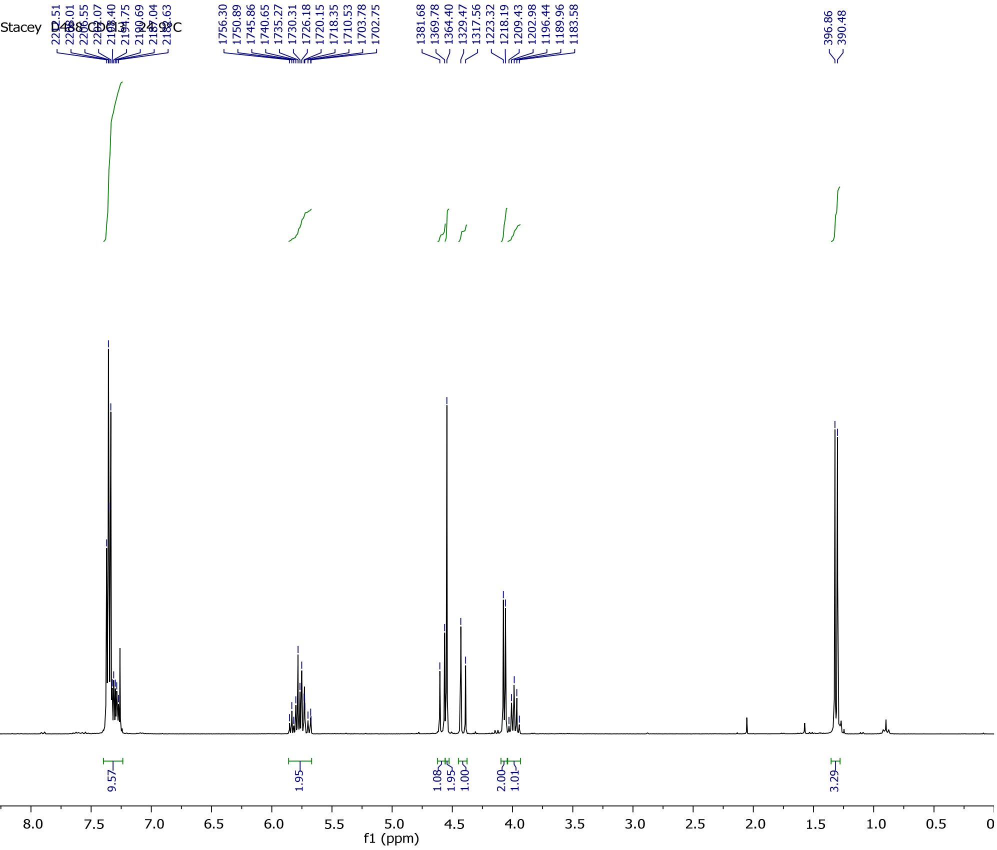
stwhd488.1.fid
1H 300.1MHz Job 48557 Webster Stacey
*
1756.30
1750.89
1745.86
1740.65
1735.27
1730.31
1726.18
1720.15
1718.35
1710.53
1703.78
1702.75
1381.68
1369.78
1364.40
1329.47
1317.56
1223.32
1218.19
1209.43
1202.98
1196.44
1189.96
1183.58
396.86
390.48
9.57
1.95
1.08
1.95
1.00
2.00
1.01
3.29
f1 (ppm)

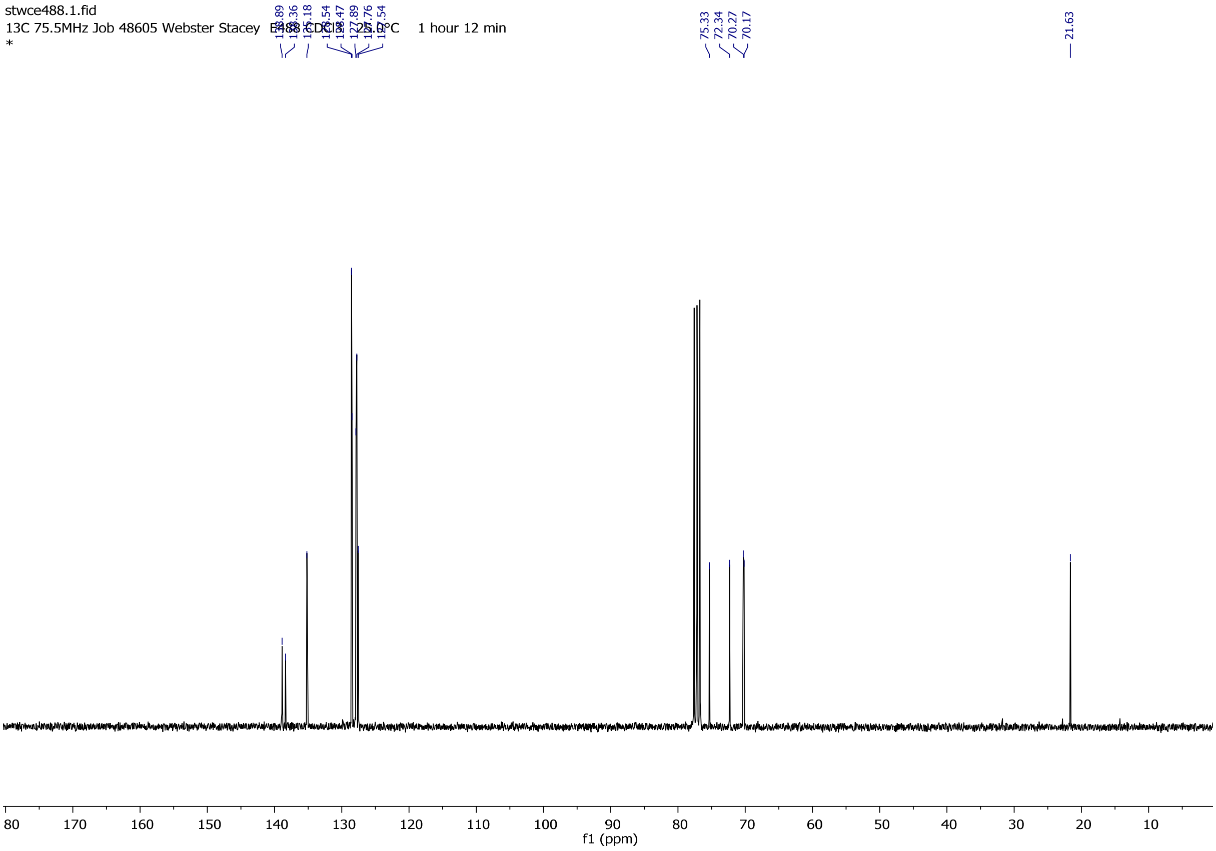
stwce488.1.fid
13C 75.5MHz Job 48605 Webster Stacey
1 hour 12 min
*
75.33
72.34
70.27
70.17
21.63
f1 (ppm)

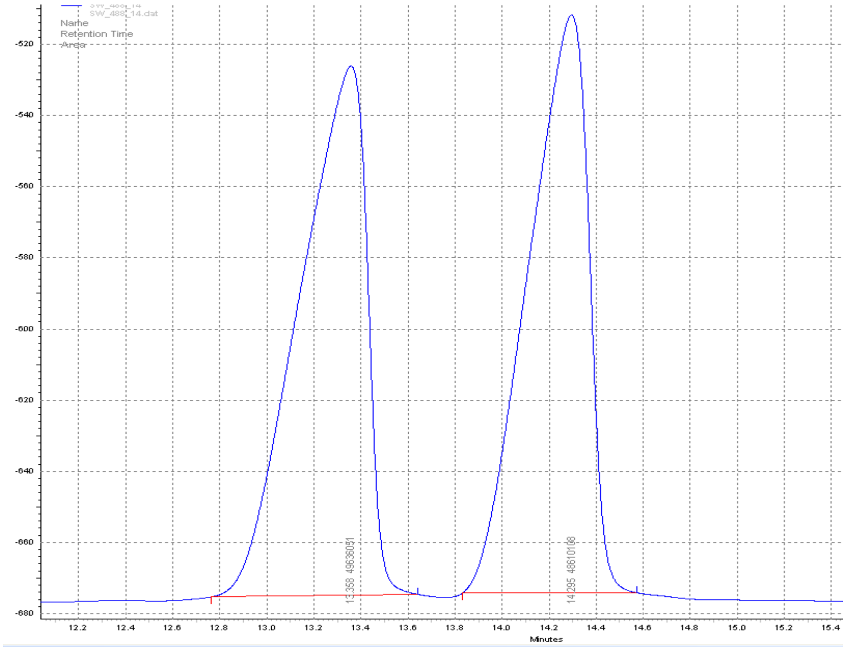

Retention Time	Area	Area %	Height	Height %
13.358	49636051	50.52	2495004	47.80
14.295	48610108	49.48	2724486	52.20
Totals	98246159	100.00	5219490	100.00

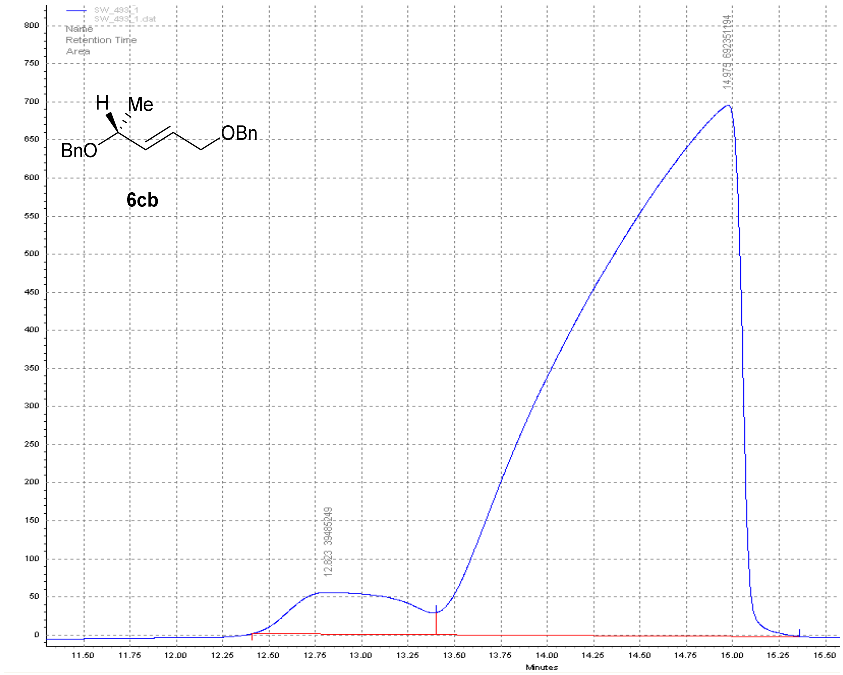

Retention Time	Area	Area %	Height	Height %
12.823	39485249	5.40	918749	7.28
14.975	692351194	94.60	11694004	92.72
Totals	731836443	100.00	12612753	100.00

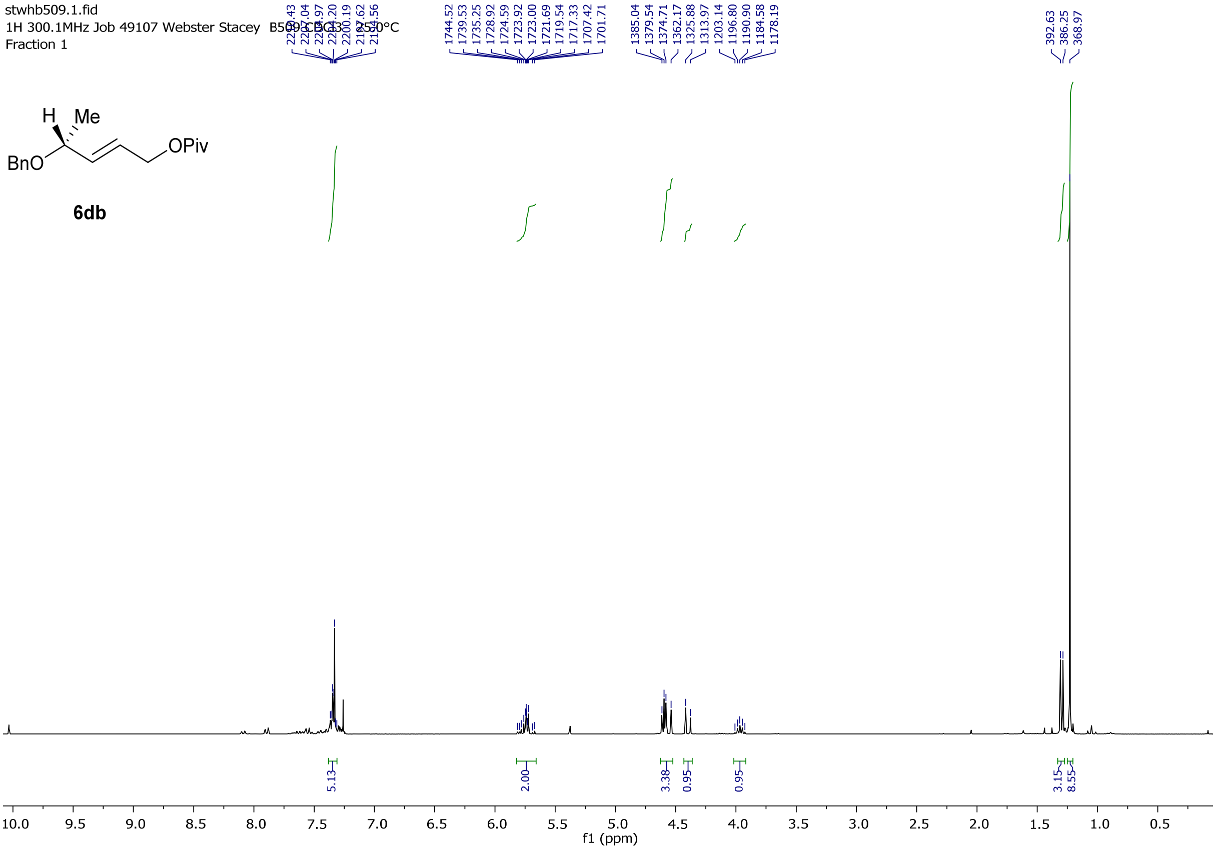
stwhb509.1.fid
1H 300.1MHz Job 49107 Webster Stacey
Fraction 1
H
Me
BnO
OPiv
f1 (ppm)

6db

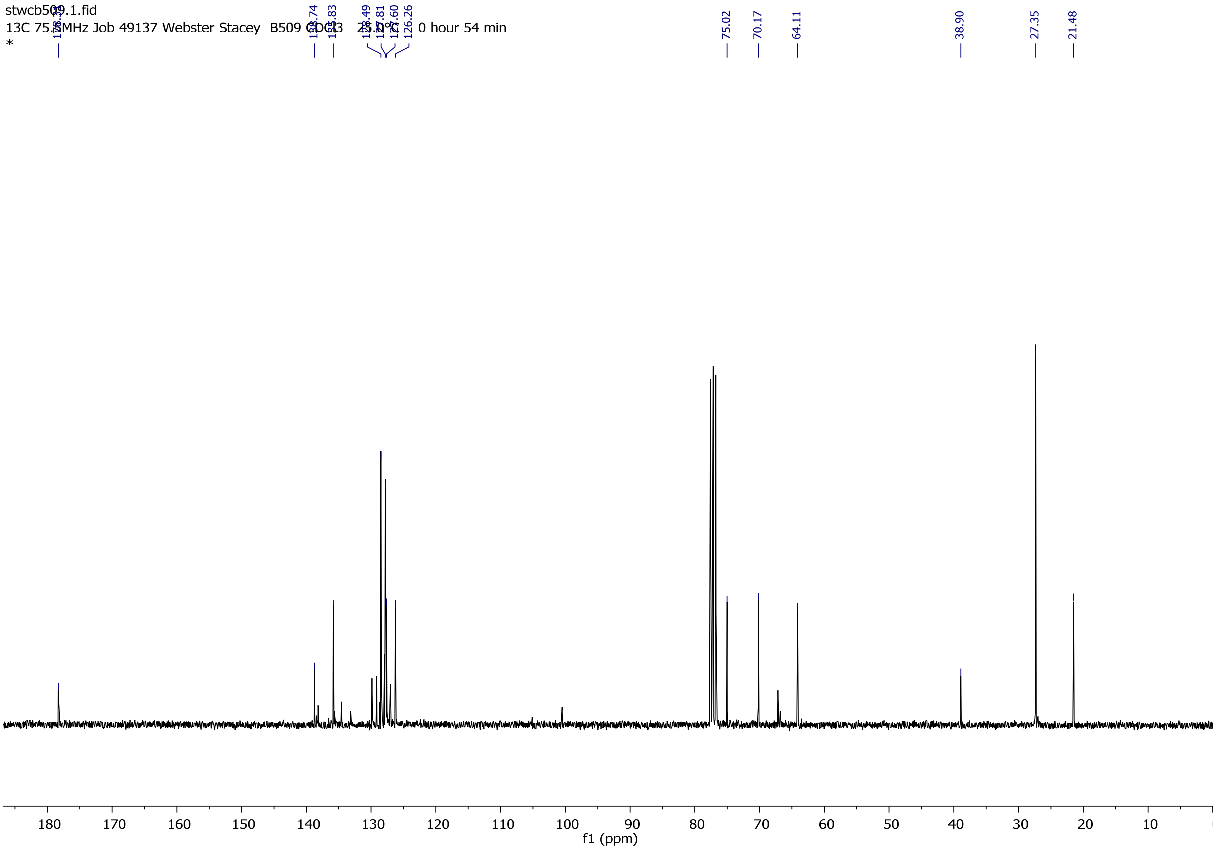
stwcb509.1.fid
13C 75.5MHz Job 49137 Webster Stacey B509
0 hour 54 min
f1 (ppm)

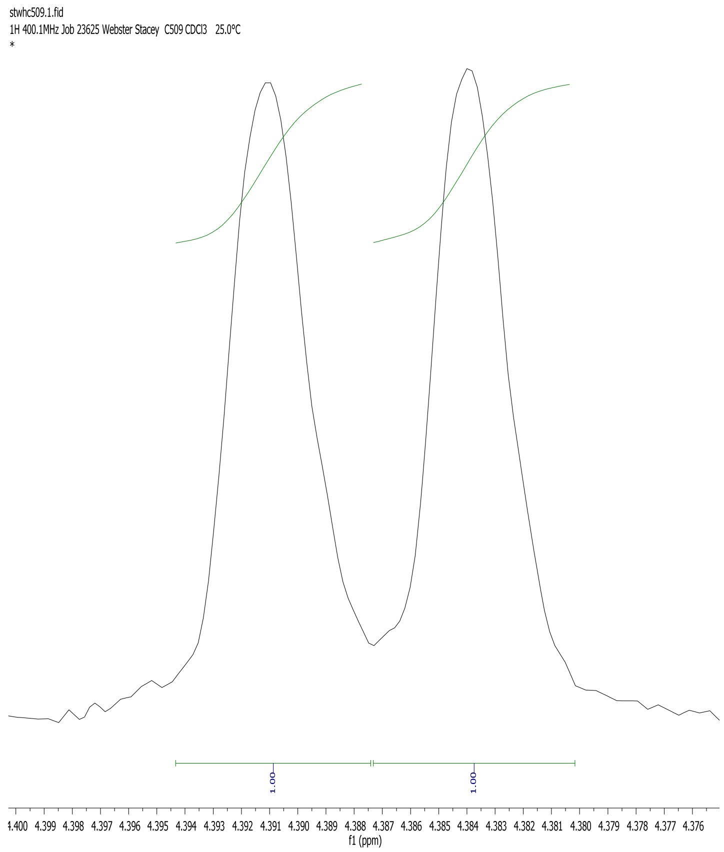
stwhc509.1.fid
1H 400.1MHz Job 23625 Webster Stacey C509 CDCl3 25.0°C
1.00
1.00
f1 (ppm)

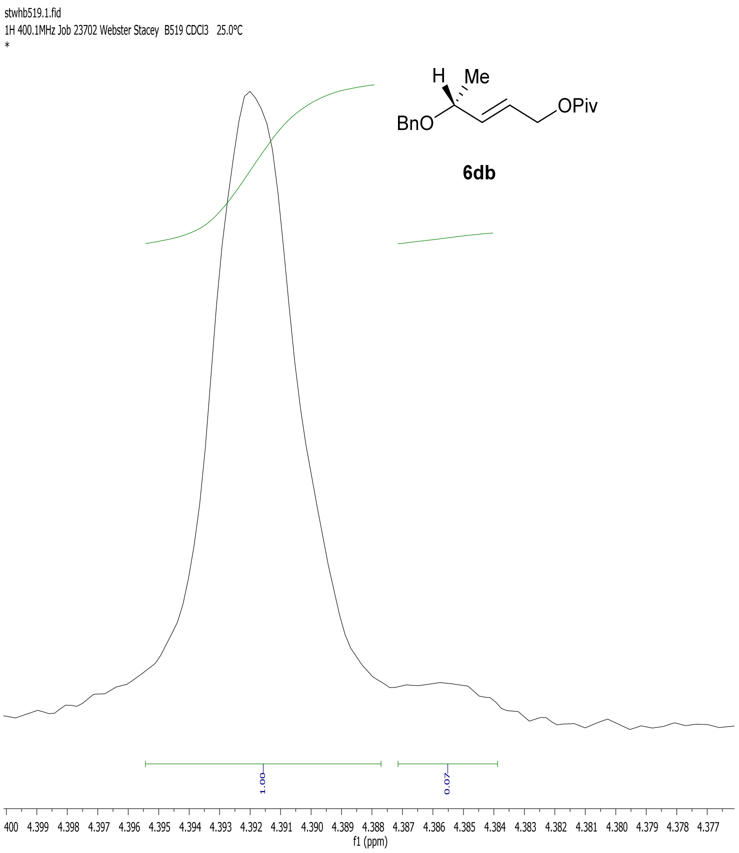
stwhb519.1.fid
1H 400.1MHz Job 23702 Webster Stacey B519 CDCl3 25.0°C
H
Me
BnO
OPiv
6db
1.00
0.07
f1 (ppm)

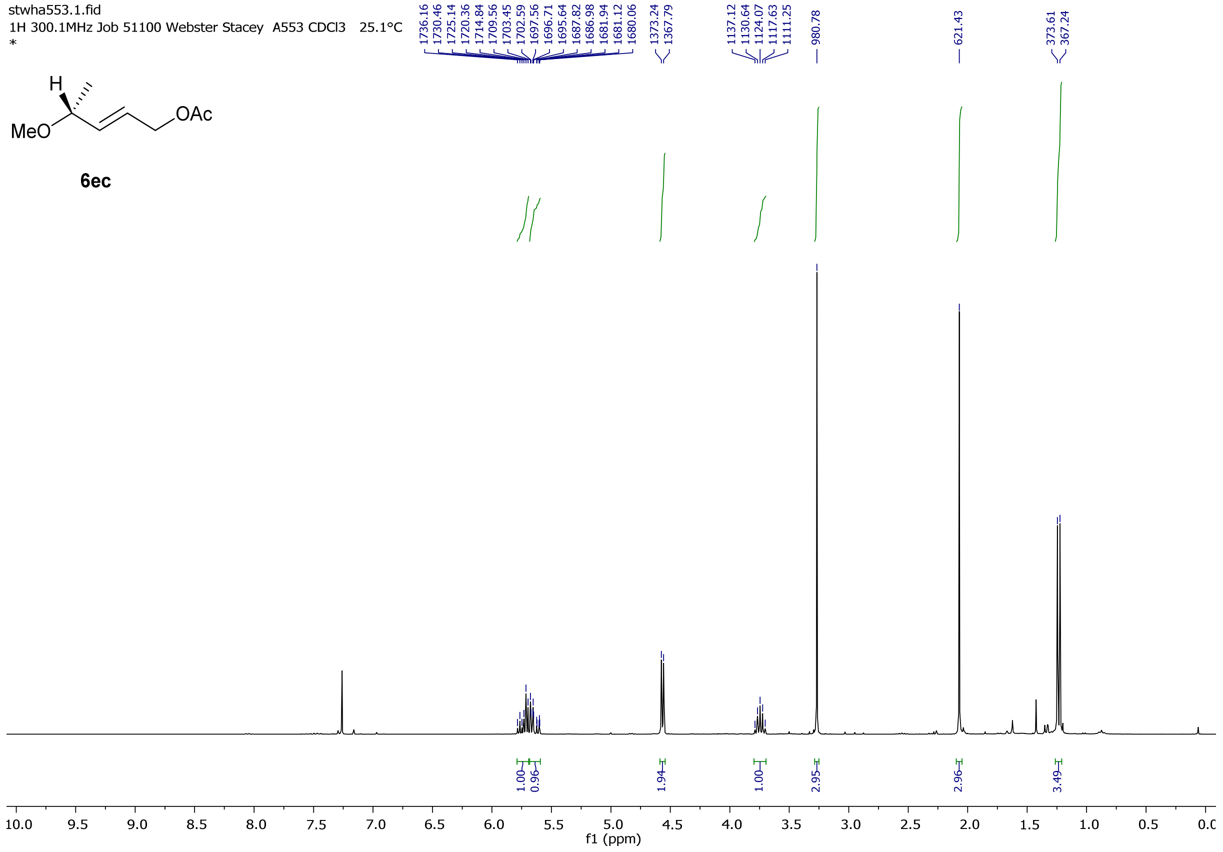
stwha553.1.fid
1H 300.1MHz Job 51100 Webster Stacey A553 CDCl3 25.1°C
H
MeO
OAc
6ec
1736.16
1730.46
1725.14
1720.36
1714.84
1709.56
1703.45
1702.59
1697.56
1696.71
1695.64
1687.82
1686.98
1681.94
1681.12
1680.06
1373.24
1367.79
1137.12
1130.64
1124.07
1117.63
1111.25
980.78
621.43
373.61
367.24
1.00
0.96
1.94
1.00
2.95
2.96
3.49
f1 (ppm)

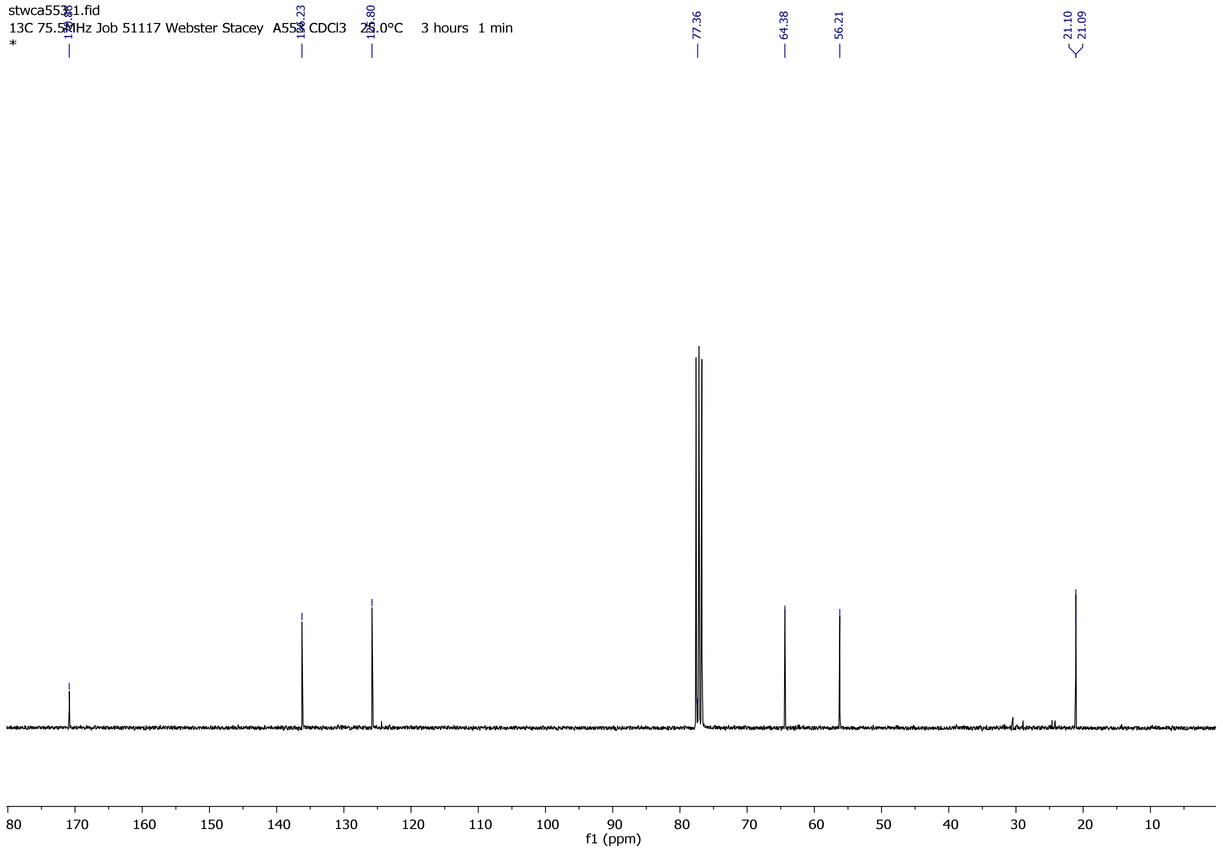
13C 75.5MHz Job 51117 Webster Stacey CDCl3 3 hours 1 min
136.23
77.36
64.38
56.21
21.10
21.09
f1 (ppm)

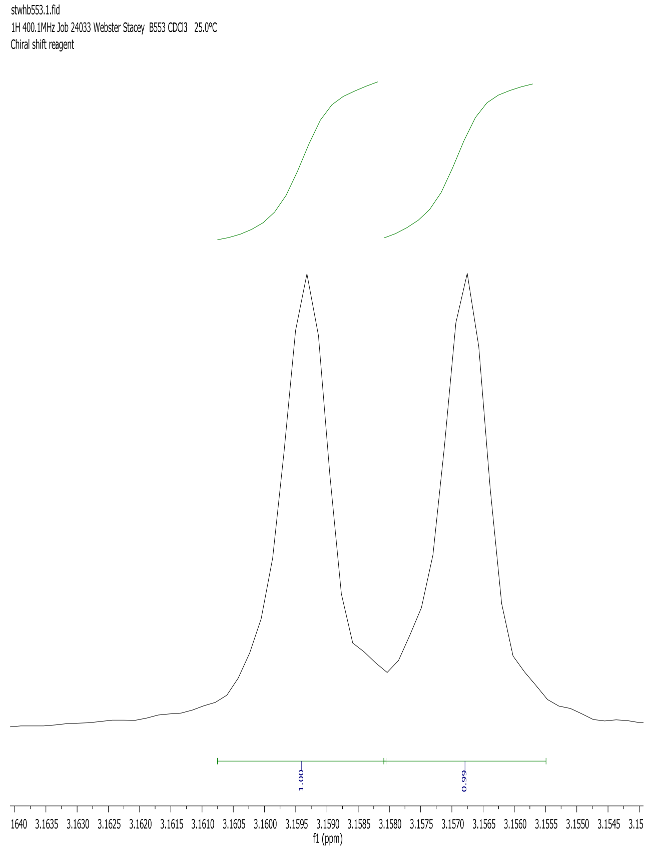
stwhb553.1.fid
1H 400.1MHz Job 24033 Webster Stacey B553 CDCl3 25.0°C
Chiral shift reagent
1.00
0.99
f1 (ppm)

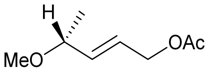

6ec

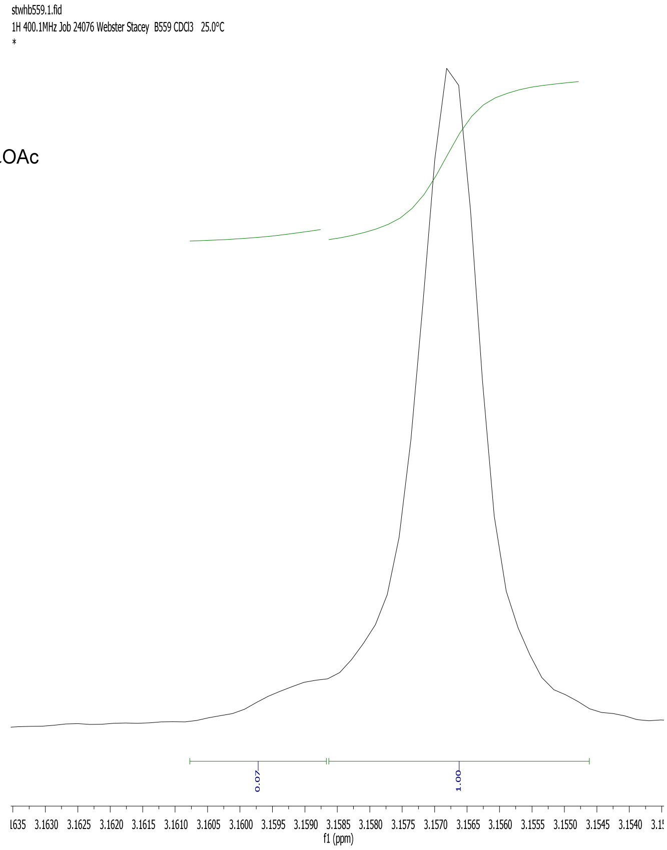
stwhb559.1.fid
1H 400.1MHz Job 24076 Webster Stacey B559 CDCl3 25.0°C
*
0.07
1.00
f1 (ppm)

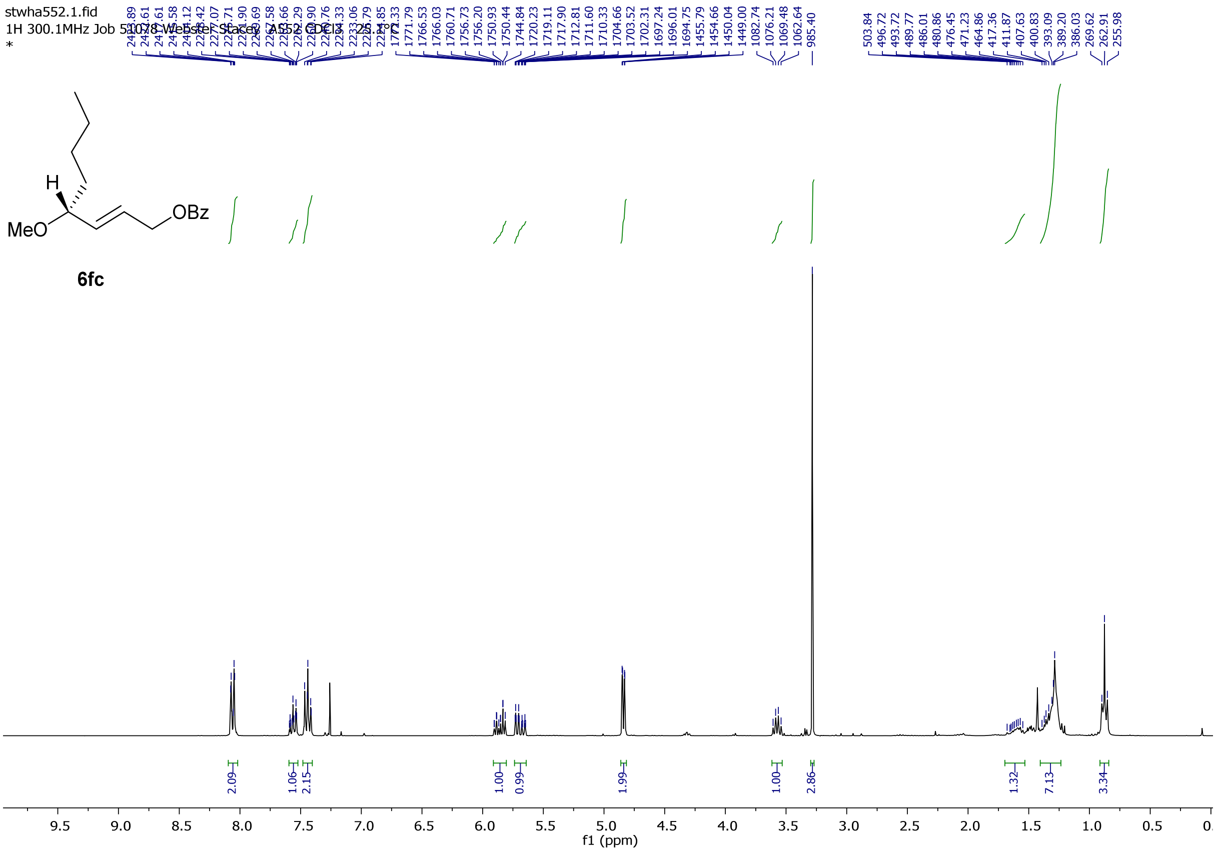
stwha552.1.fid
1H 300.1MHz Job 51078 Webster Stacey A552 CDCl3 25.1°C
MeO
H
OBz
6fc
f1 (ppm)

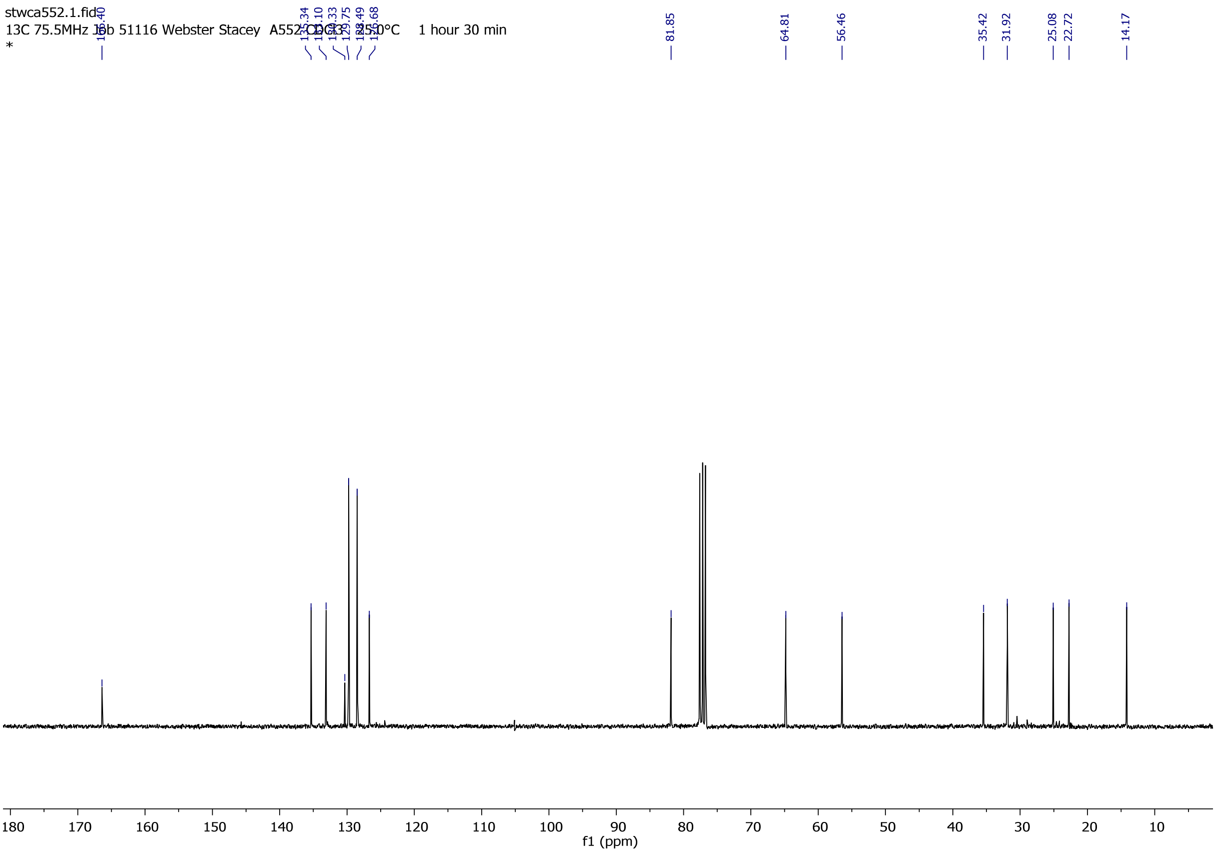
stwca552.1.fid
13C 75.5MHz Job 51116 Webster Stacey A552 CDCl3 25.0°C 1 hour 30 min
81.85
64.81
56.46
35.42
31.92
25.08
22.72
14.17
f1 (ppm)

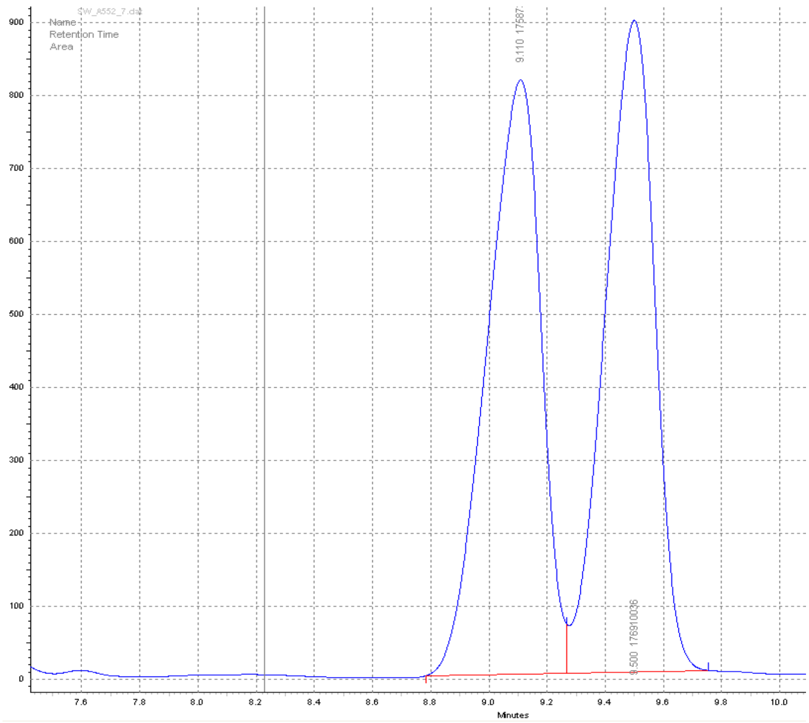

Retention Time	Area	Area %	Height	Height %
9.110	175873703	49.85	13663308	47.69
9.500	176910036	50.15	14987604	52.31
Totals	352783739	100.00	28650912	100.00

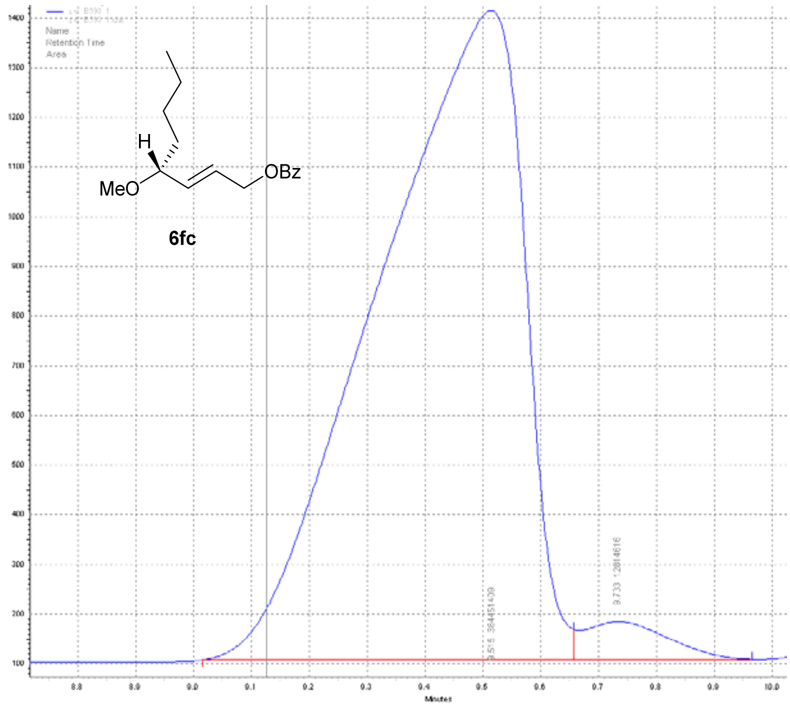

Retention Time	Area	Area %	Height	Height %
9.515	384451409	96.77	21911519	94.53
9.733	12814616	3.23	1268780	5.47
Totals	397266025	100.00	23180299	100.00

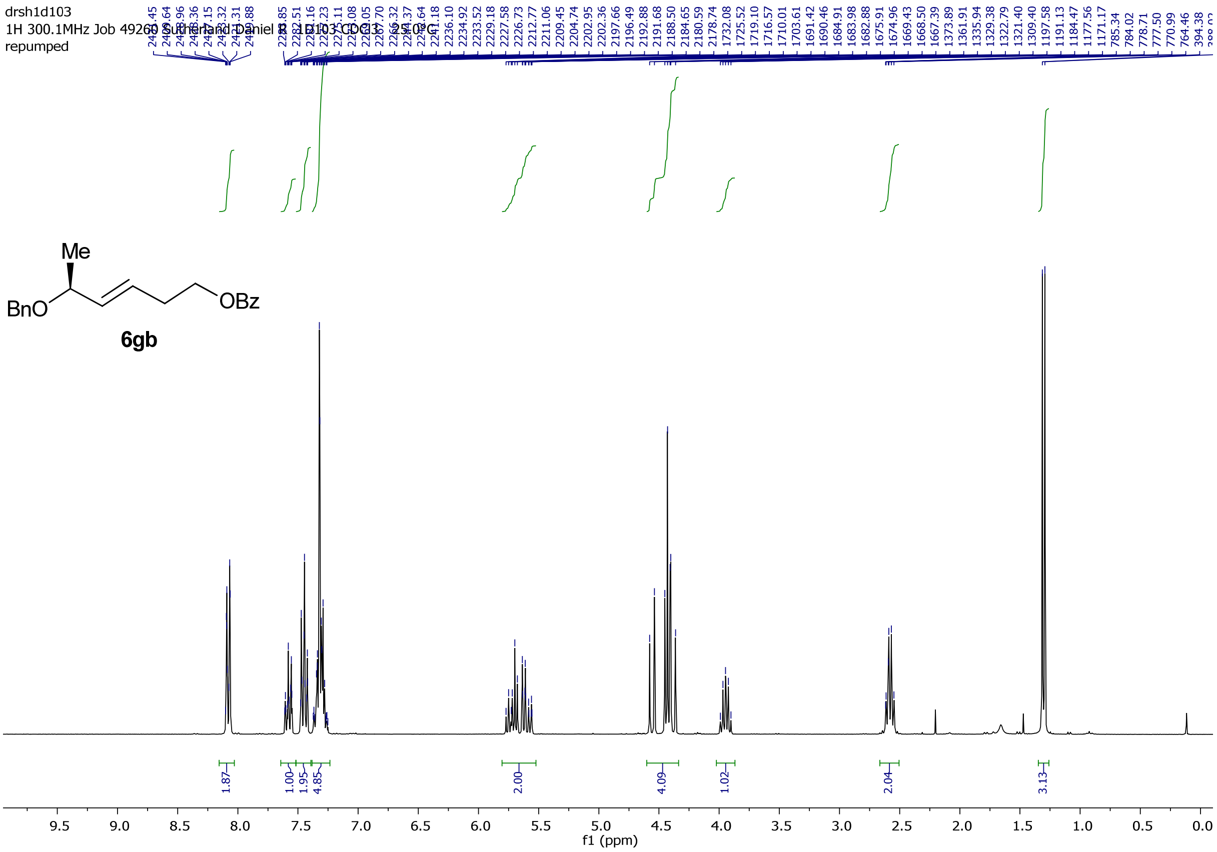
drsh1d103
1H 300.1MHz Job 49260 Sutherland Daniel R 1d103 CDCl3 25.0°C
repumped
Me
BnO
OBz
6gb
f1 (ppm)

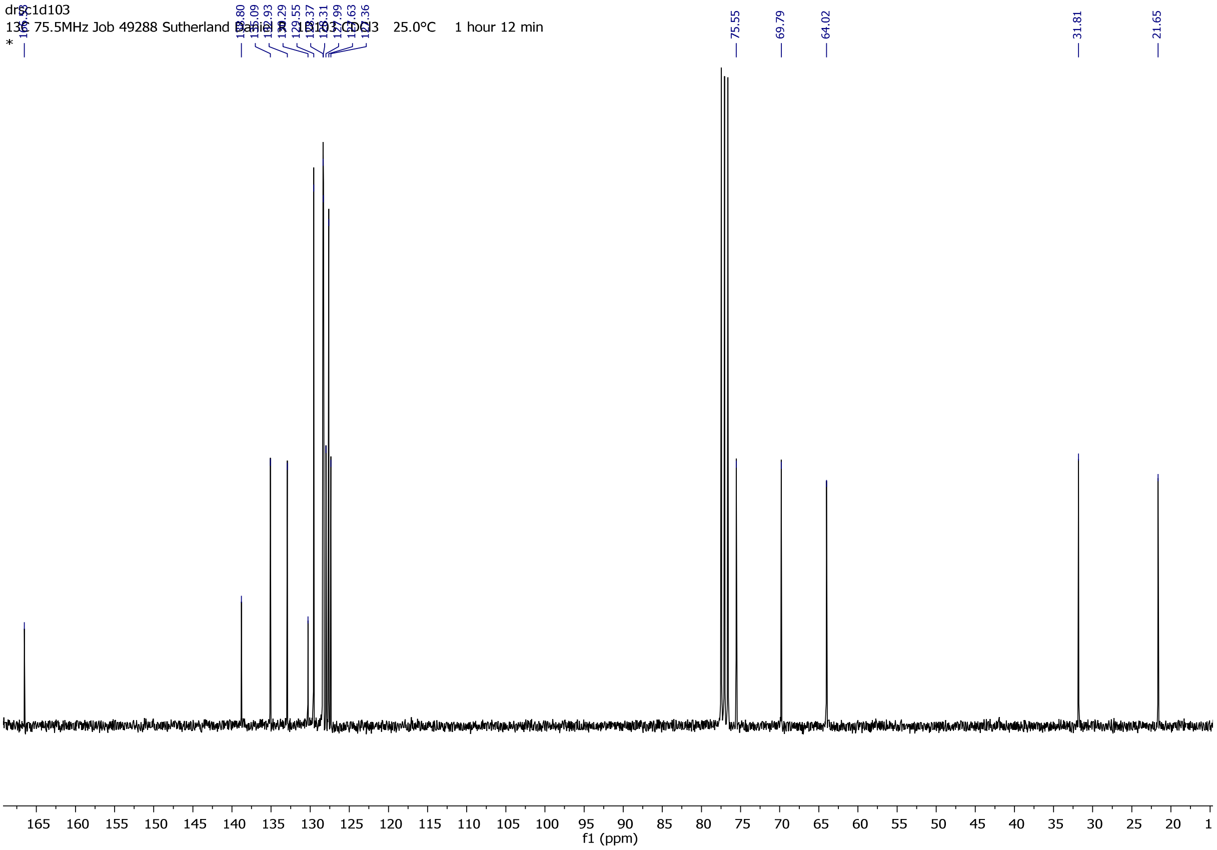
drsc1d103
13C 75.5MHz Job 49288 Sutherland Daniel R 1d103 CDCl3 25.0°C 1 hour 12 min
75.55
69.79
64.02
31.81
21.65
f1 (ppm)

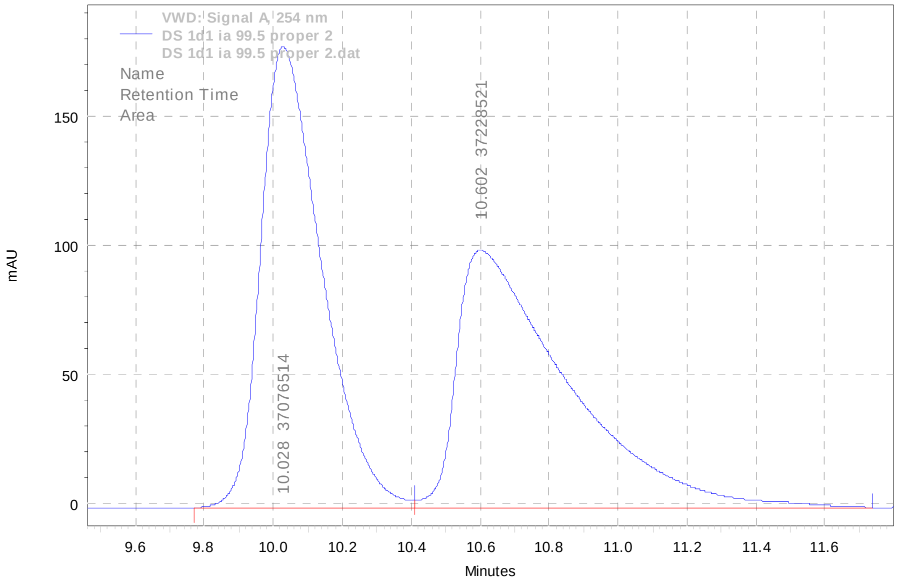

Retention Time	Area	Area %	Height	Height %
10.028	37076514	49.90	2994089	64.17
10.602	37228521	50.10	1671607	35.83
Totals	74305035	100.00	4665696	100.00

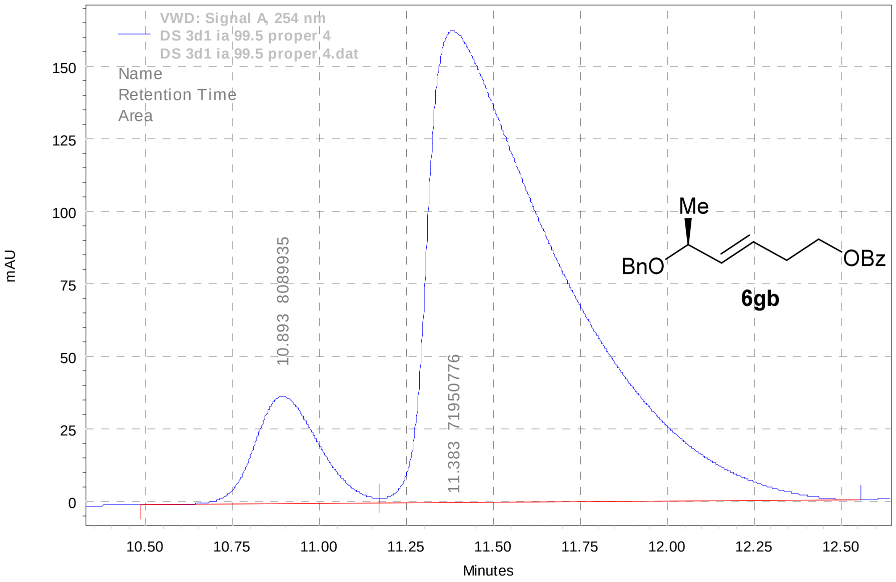

Retention Time	Area	Area %	Height	Height %
10.893	8089935	10.11	623374	18.60
11.383	71950776	89.89	2727743	81.40
Totals	80040711	100.00	3351117	100.00

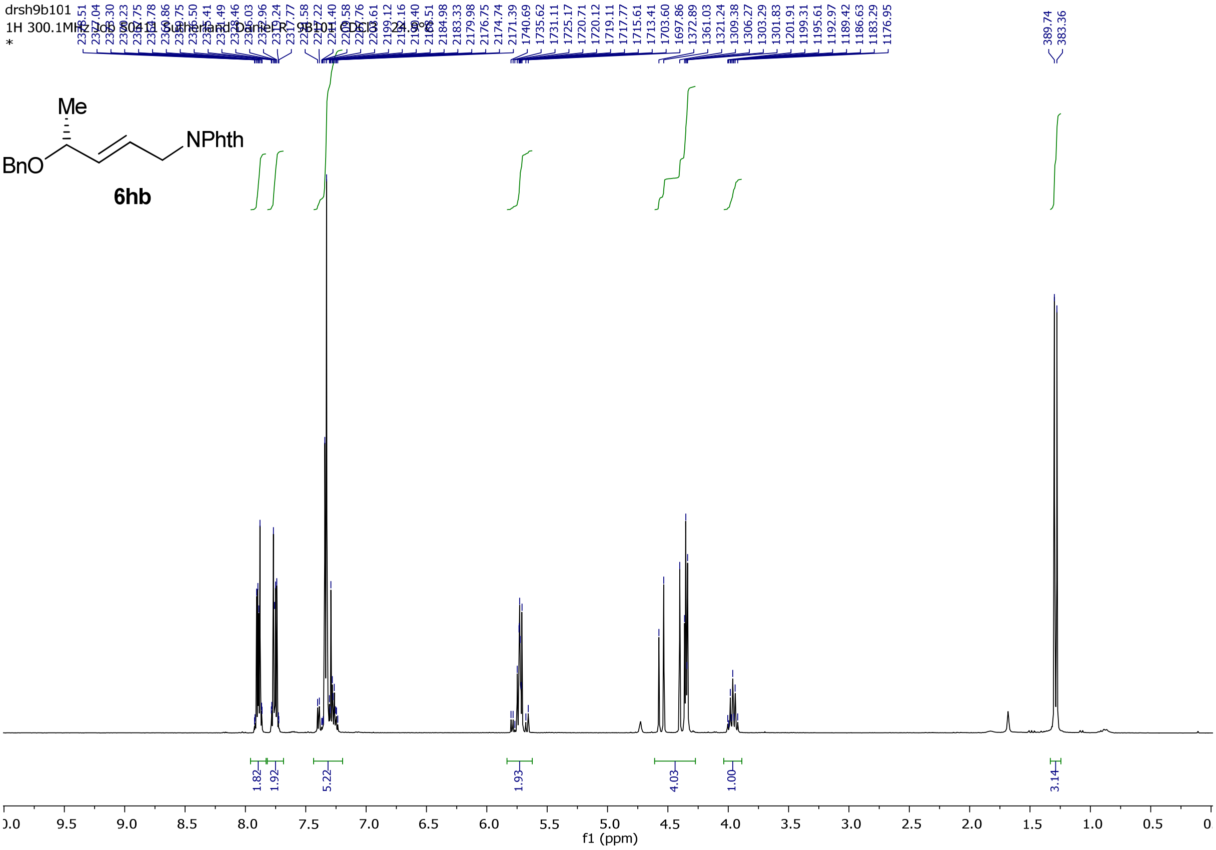
drsh9b101
Me
BnO
NPhth
6hb
389.74
383.36
1.82
1.92
5.22
1.93
4.03
1.00
3.14
f1 (ppm)

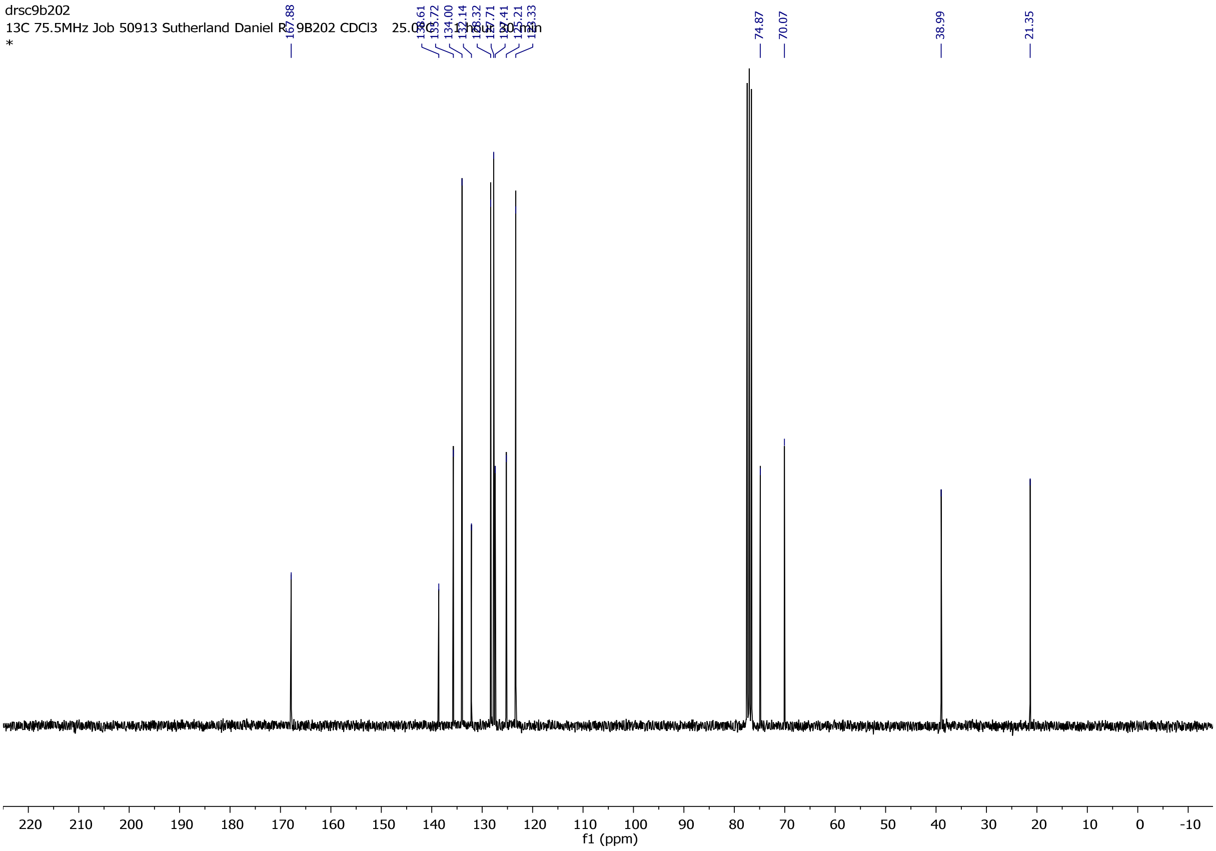
drsc9b202
167.88
74.87
70.07
38.99
21.35
f1 (ppm)

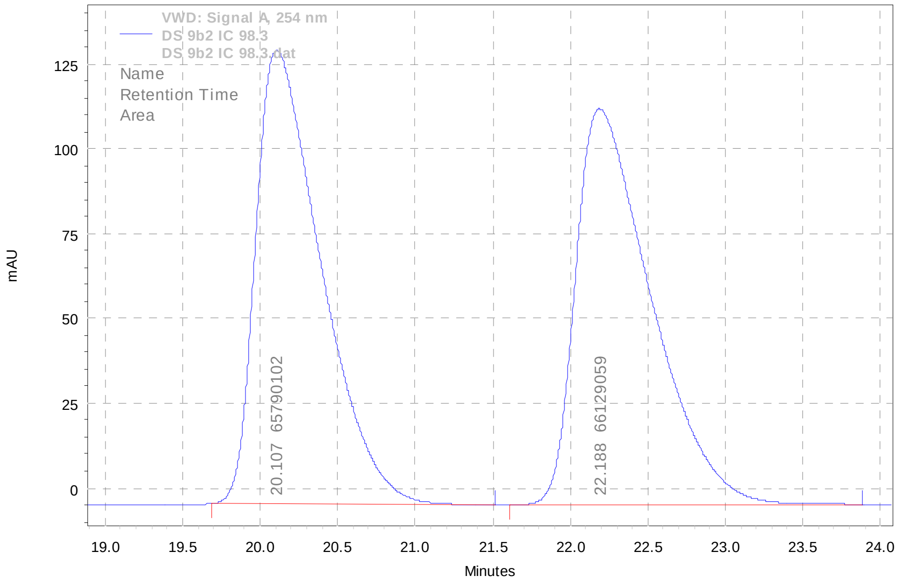

Retention Time	Area	Area %	Height	Height %
20.107	65790102	49.87	2242258	53.38
22.188	66129059	50.13	1958521	46.62
Totals	131919161	100.00	4200779	100.00

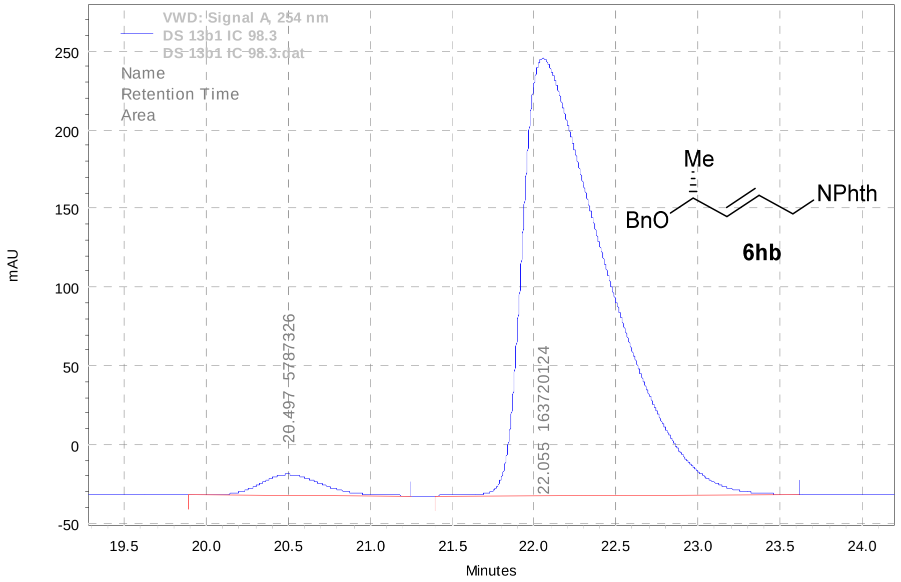

Retention Time	Area	Area %	Height	Height %
20.497	5787326	3.41	222680	4.57
22.055	163720124	96.59	4653404	95.43
Totals	169507450	100.00	4876084	100.00

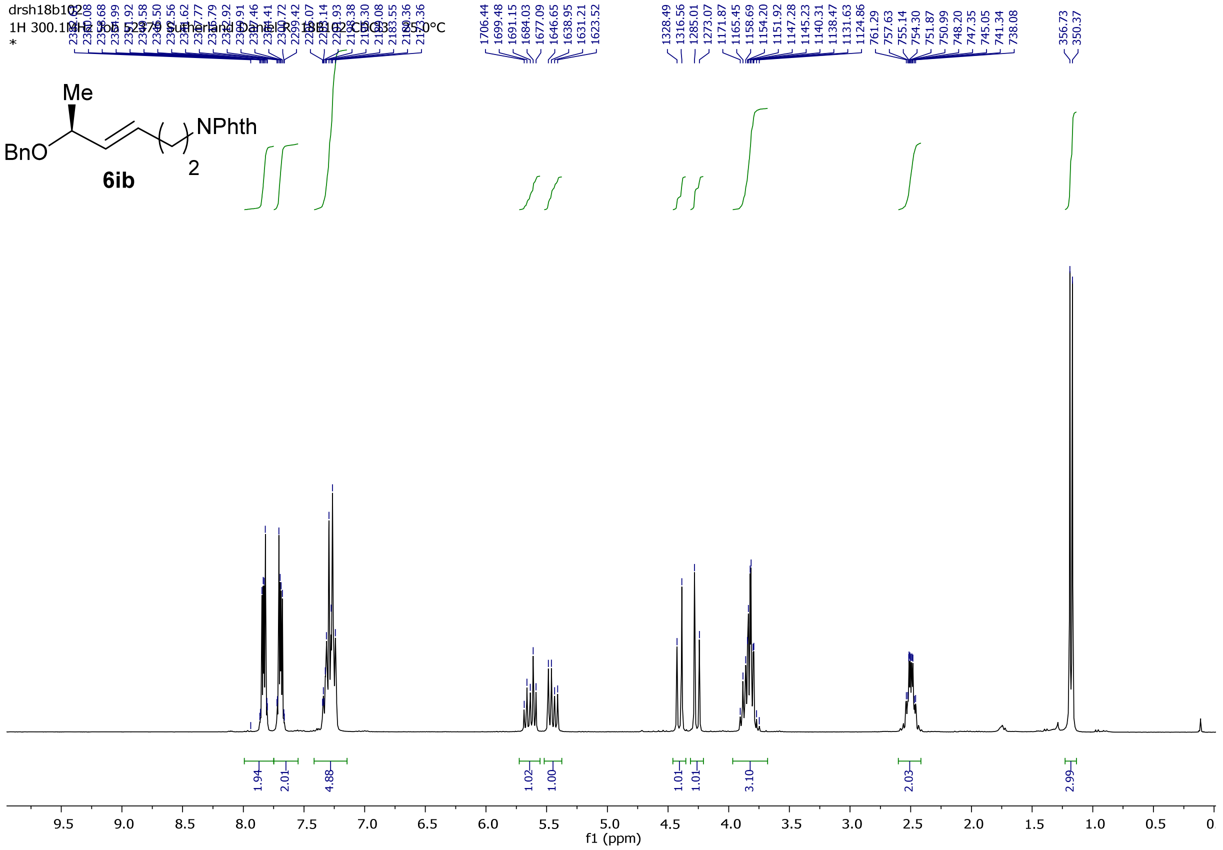
drsh18b102
*
Me
BnO
NPhth
2
6ib
1.94
2.01
4.88
1.02
1.00
1.01
1.01
3.10
2.03
2.99
f1 (ppm)

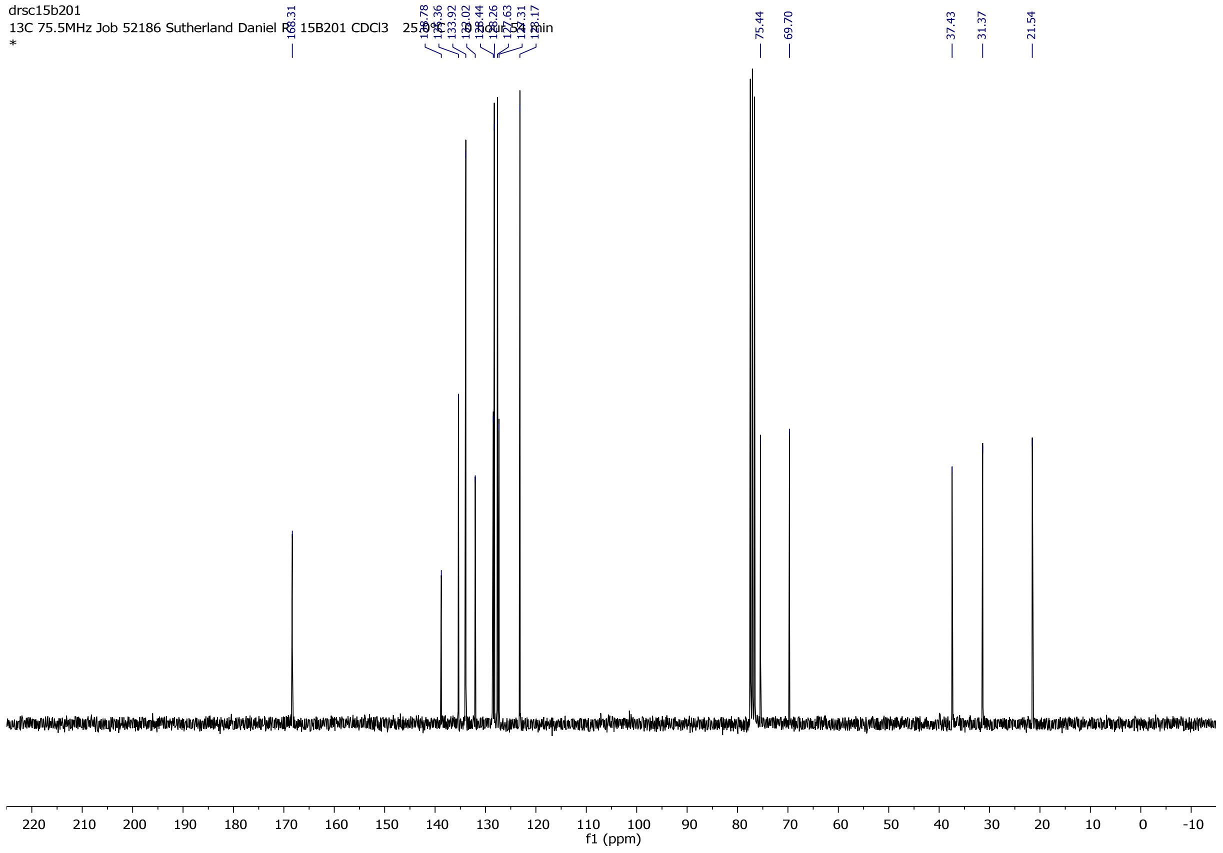
drsc15b201
13C 75.5MHz Job 52186 Sutherland Daniel R. 15B201 CDCl3
*
75.44
69.70
37.43
31.37
21.54
f1 (ppm)

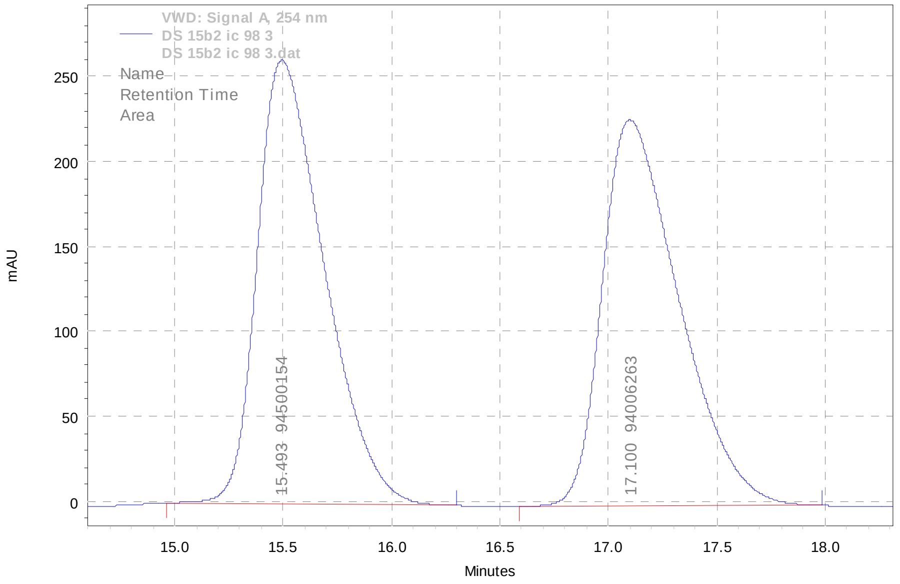

Retention Time	Area	Area %	Height	Height %
15.493	94500154	50.13	4386611	53.50
17.100	94006263	49.87	3813159	46.50
Totals	188506417	100.00	8199770	100.00

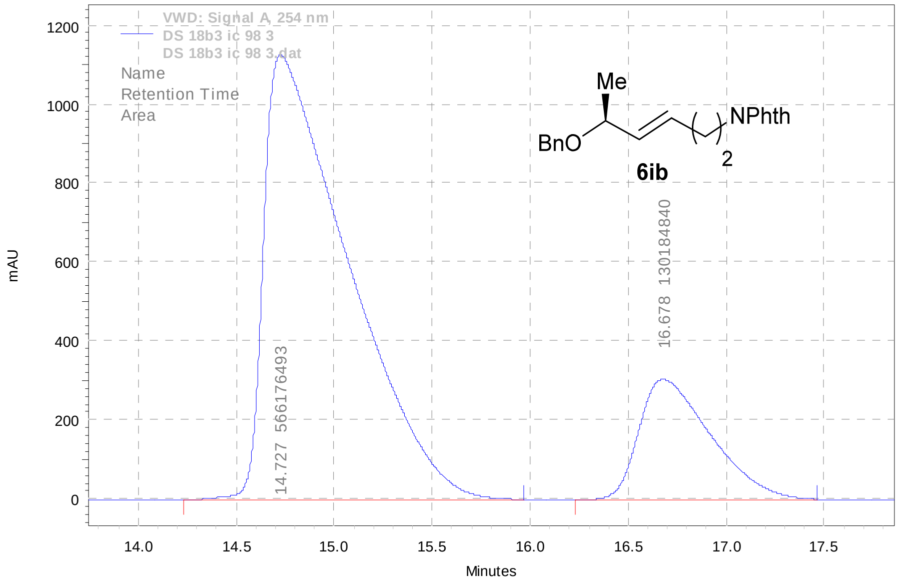

Retention Time	Area	Area %	Height	Height %
14.727	566176493	81.30	18905196	78.71
16.678	130184840	18.70	5114197	21.29
Totals	696361333	100.00	24019393	100.00

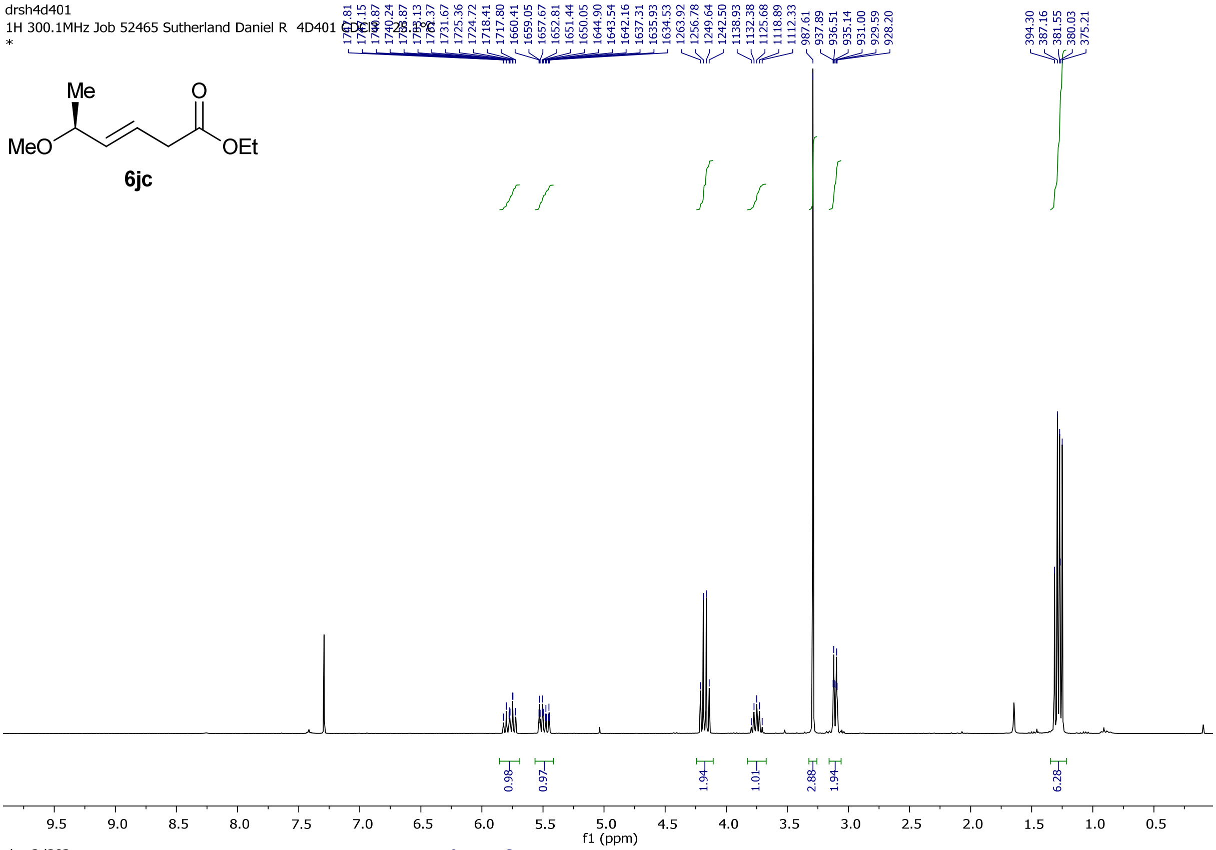
drsh4d401
1H 300.1MHz Job 52465 Sutherland Daniel R 4D401 CDCl3 25.0°C
*
Me
O
MeO
OEt
6jc
0.98
0.97
1.94
1.01
2.88
1.94
6.28
f1 (ppm)

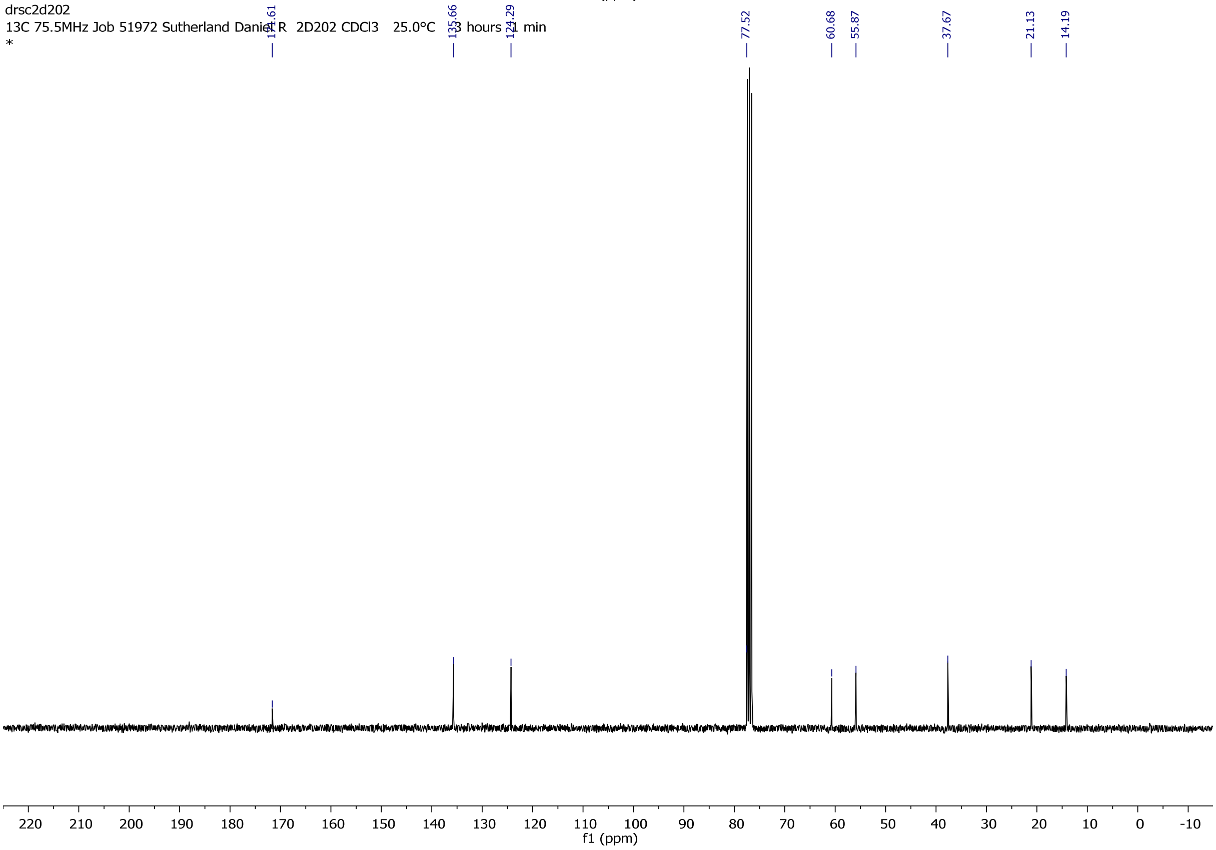
drsc2d202
13C 75.5MHz Job 51972 Sutherland Daniel R 2D202 CDCl3 25.0°C 3 hours 41 min
*
171.61
135.66
124.29
77.52
60.68
55.87
37.67
21.13
14.19
f1 (ppm)

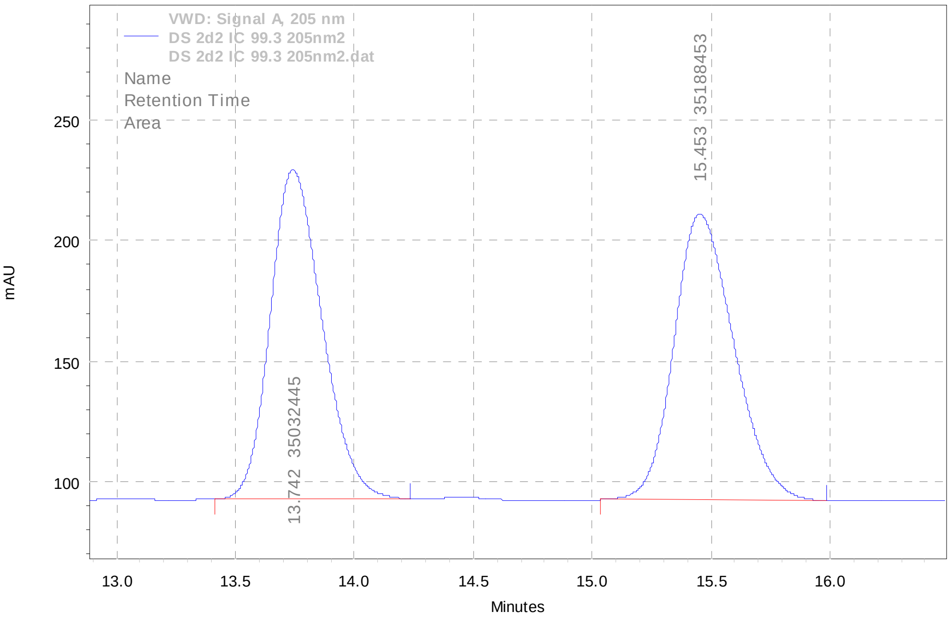

Retention Time	Area	Area %	Height	Height %
13.742	35032445	49.89	2302704	53.52
15.453	35188453	50.11	2000026	46.48
Totals	70220898	100.00	4302730	100.00

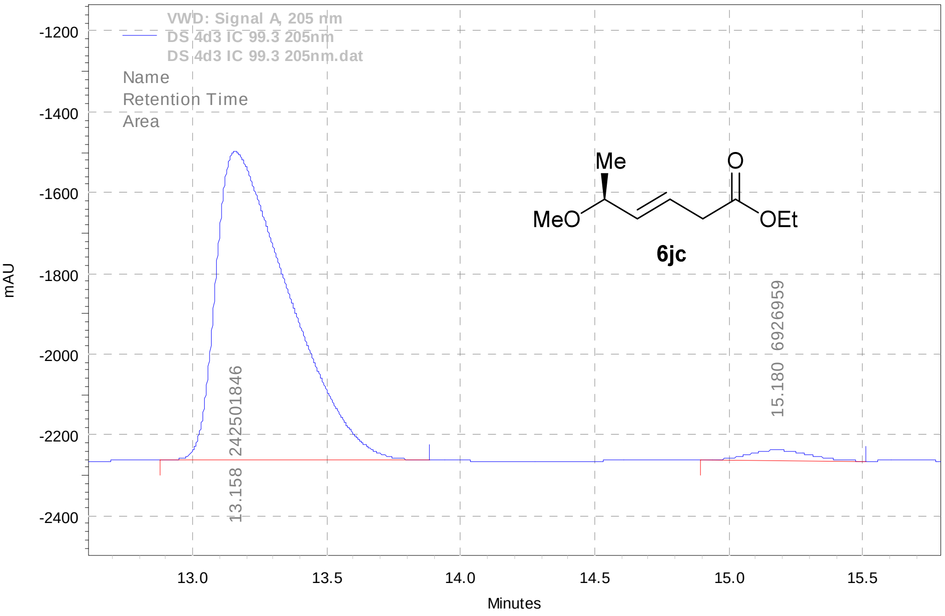

Retention Time	Area	Area %	Height	Height %
13.158	242501846	97.22	12804368	96.71
15.180	6926959	2.78	435113	3.29
Totals	249428805	100.00	13239481	100.00

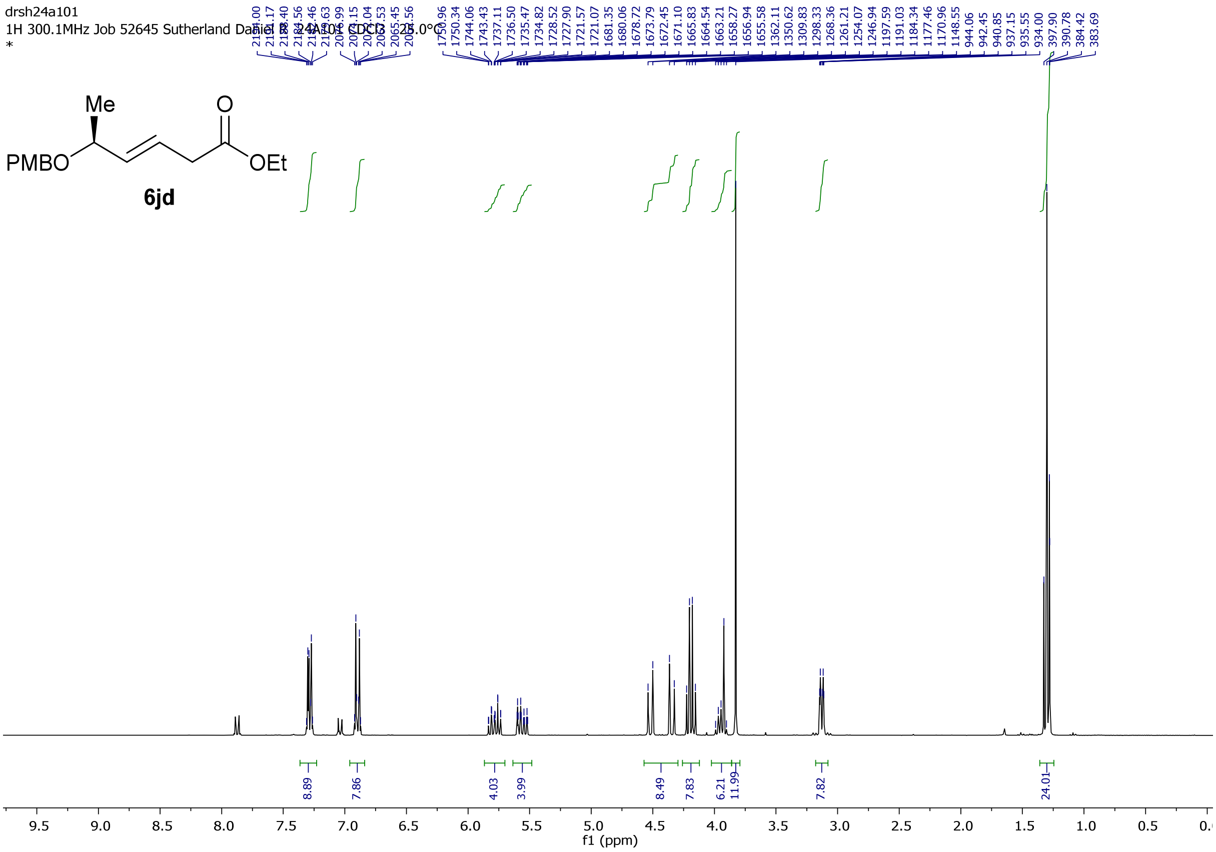
drsh24a101
1H 300.1MHz Job 52645 Sutherland Daniel R 24A101 CDCl3 25.0°C
Me
O
PMBO
OEt
6jd
f1 (ppm)

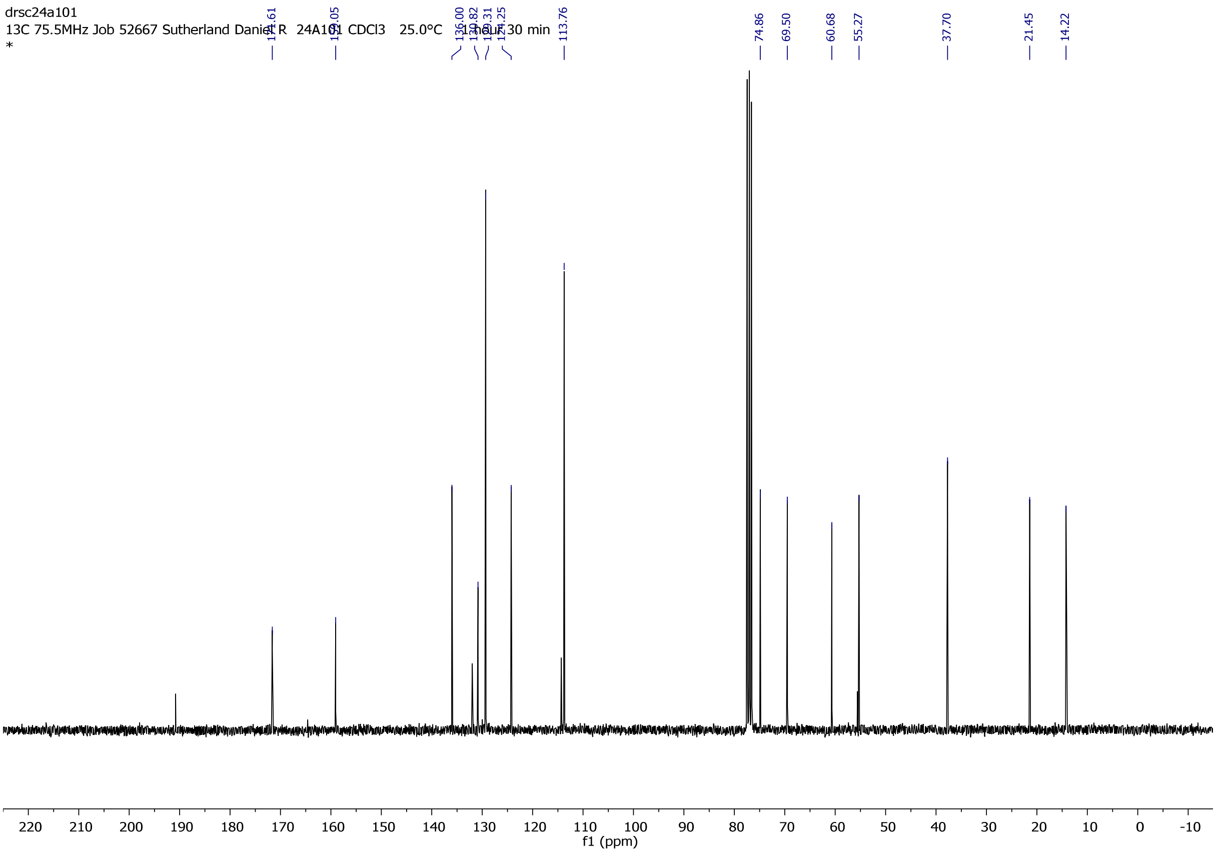
drsc24a101
13C 75.5MHz Job 52667 Sutherland Daniel R 24A101 CDCl3 25.0°C 1 hour 30 min
136.00
130.82
129.31
124.25
113.76
74.86
69.50
60.68
55.27
37.70
21.45
14.22
f1 (ppm)

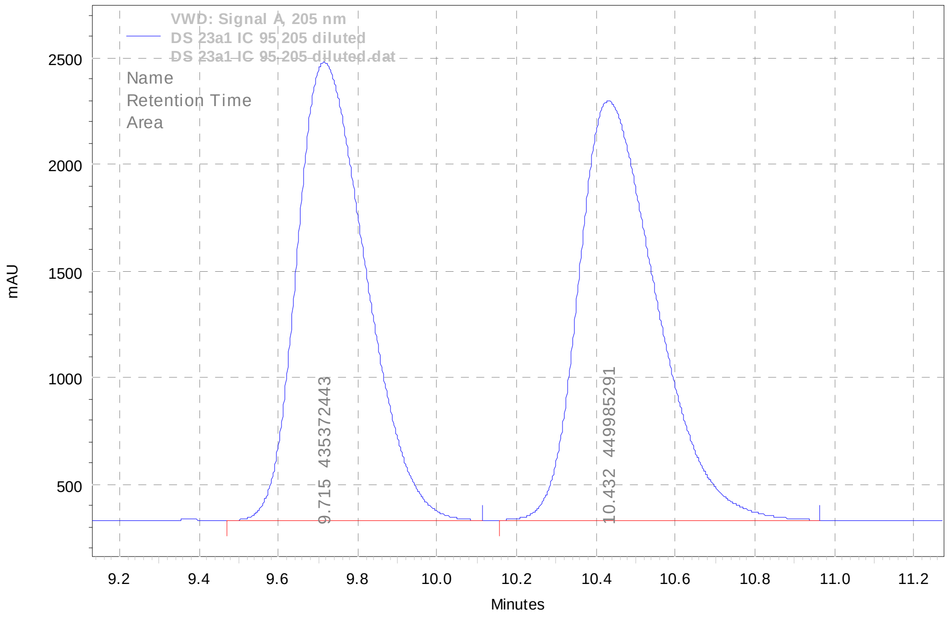

Retention Time	Area	Area %	Height	Height %
9.715	435372443	49.17	36069452	52.20
10.432	449985291	50.83	33024079	47.80
Totals	885357734	100.00	69093531	100.00

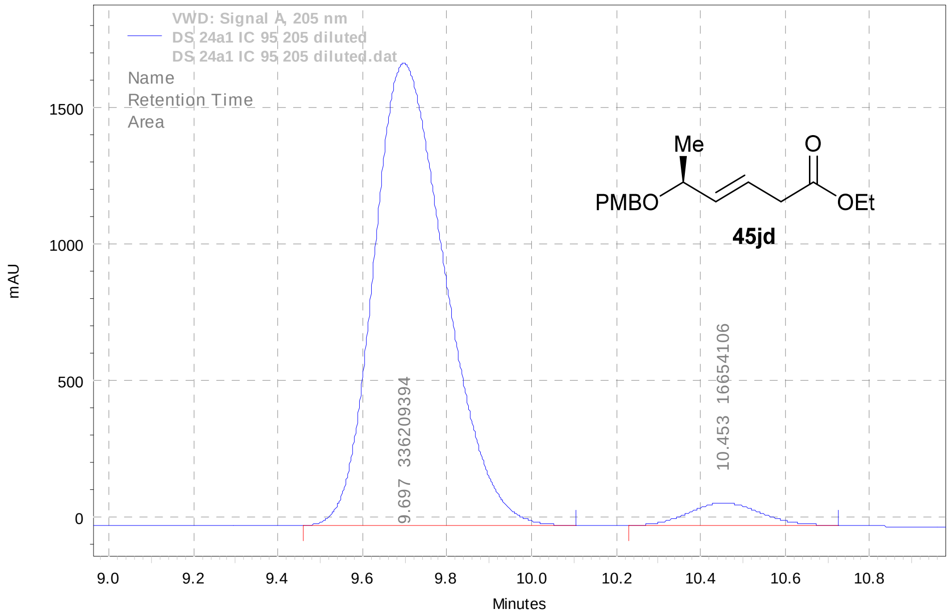

Retention Time	Area	Area %	Height	Height %
9.697	336209394	95.28	28476888	95.32
10.453	16654106	4.72	1399124	4.68
Totals	352863500	100.00	29876012	100.00

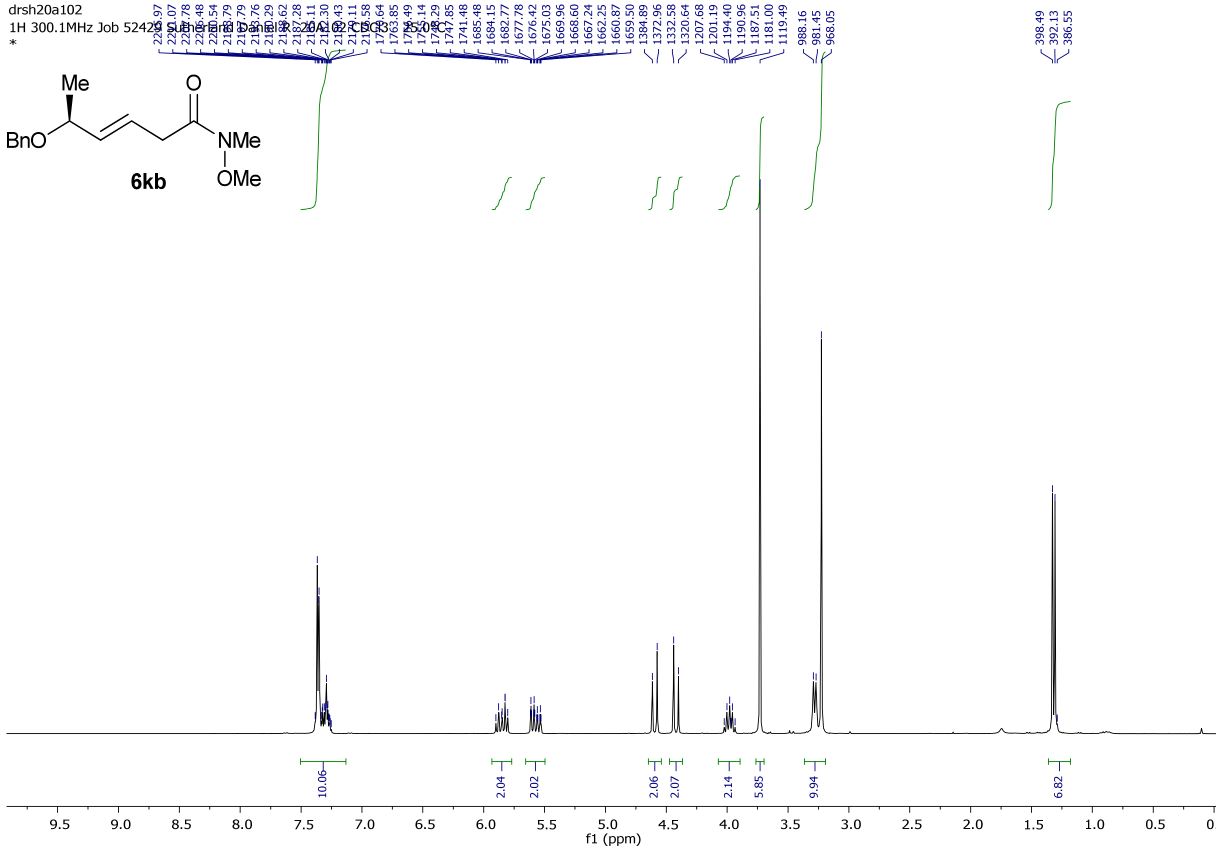
drsh20a102
1H 300.1MHz Job 52429 Sutherland Daniel R 20A102 CDCl3 25.0°C
Me
O
BnO
NMe
OMe
6kb
1384.89
1372.96
1332.58
1320.64
1207.68
1201.19
1194.40
1190.96
1187.51
1181.00
1119.49
988.16
981.45
968.05
398.49
392.13
386.55
10.06
2.04
2.02
2.06
2.07
2.14
5.85
9.94
6.82
f1 (ppm)

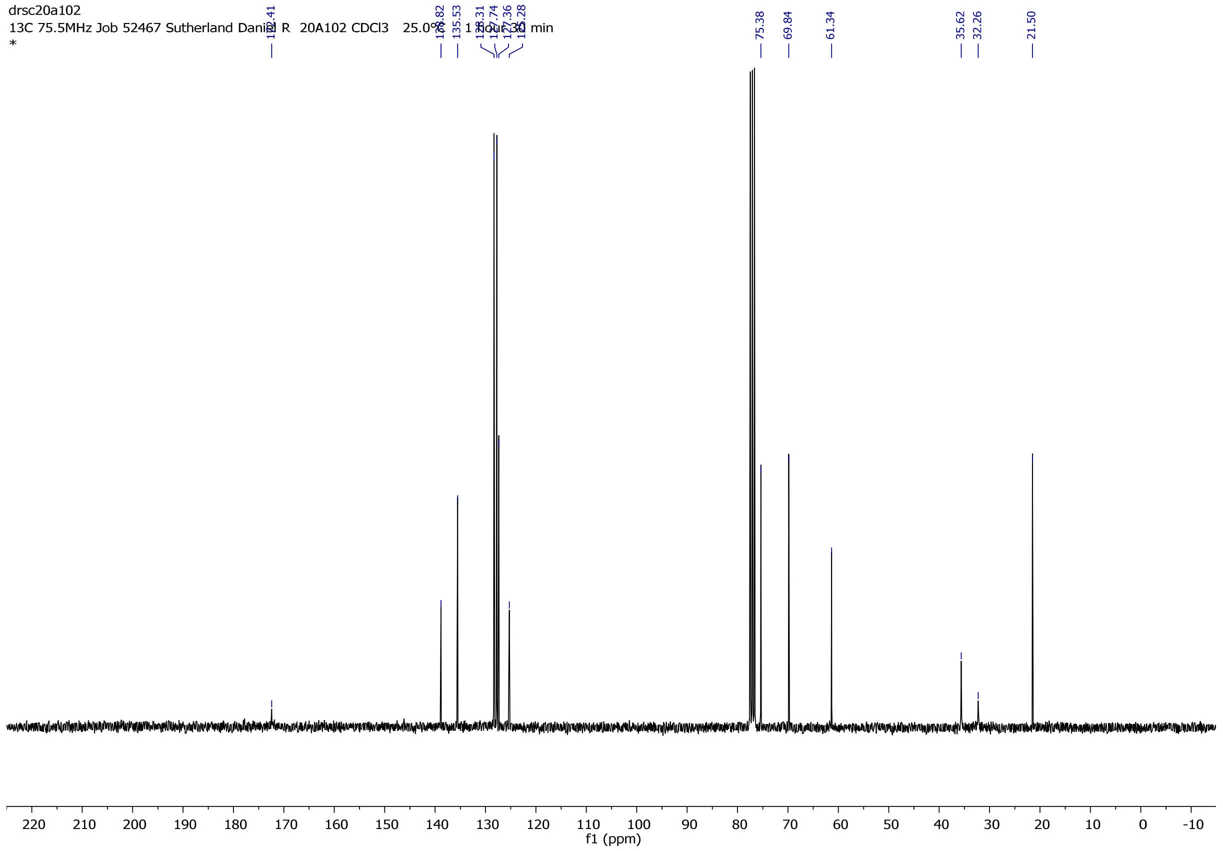
drsc20a102
13C 75.5MHz Job 52467 Sutherland Daniel R 20A102 CDCl3 25.0°C
75.38
69.84
61.34
35.62
32.26
21.50
f1 (ppm)

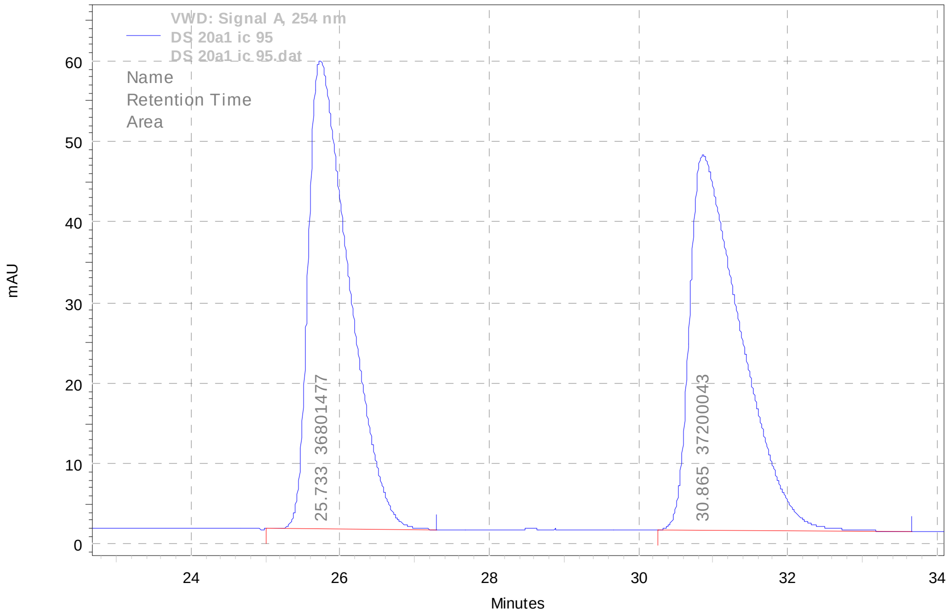

Retention Time	Area	Area %	Height	Height %
25.733	36801477	49.73	975764	55.53
30.865	37200043	50.27	781544	44.47
Totals	74001520	100.00	1757308	100.00

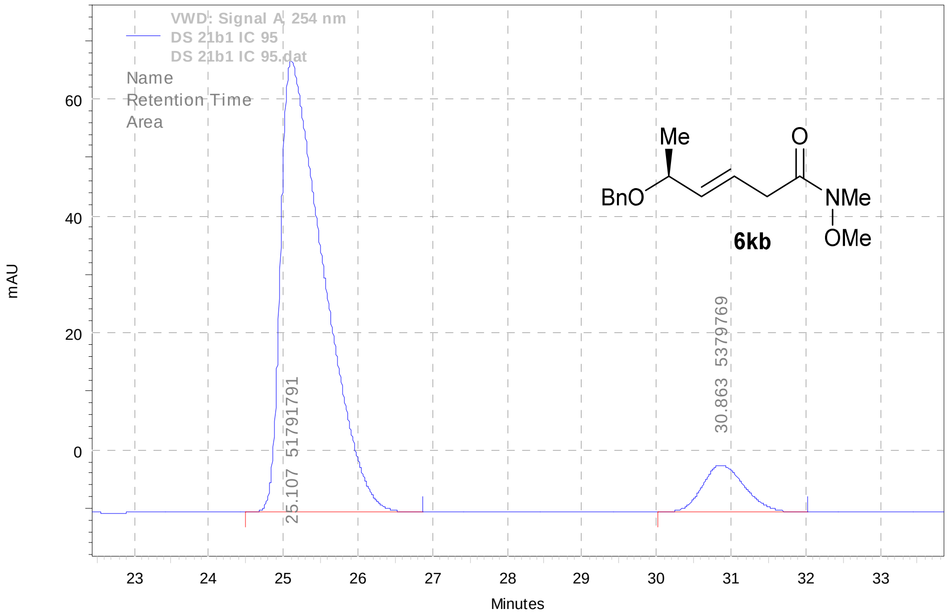

Retention Time	Area	Area %	Height	Height %
25.107	51791791	90.59	1293498	90.51
30.863	5379769	9.41	135688	9.49
Totals	57171560	100.00	1429186	100.00

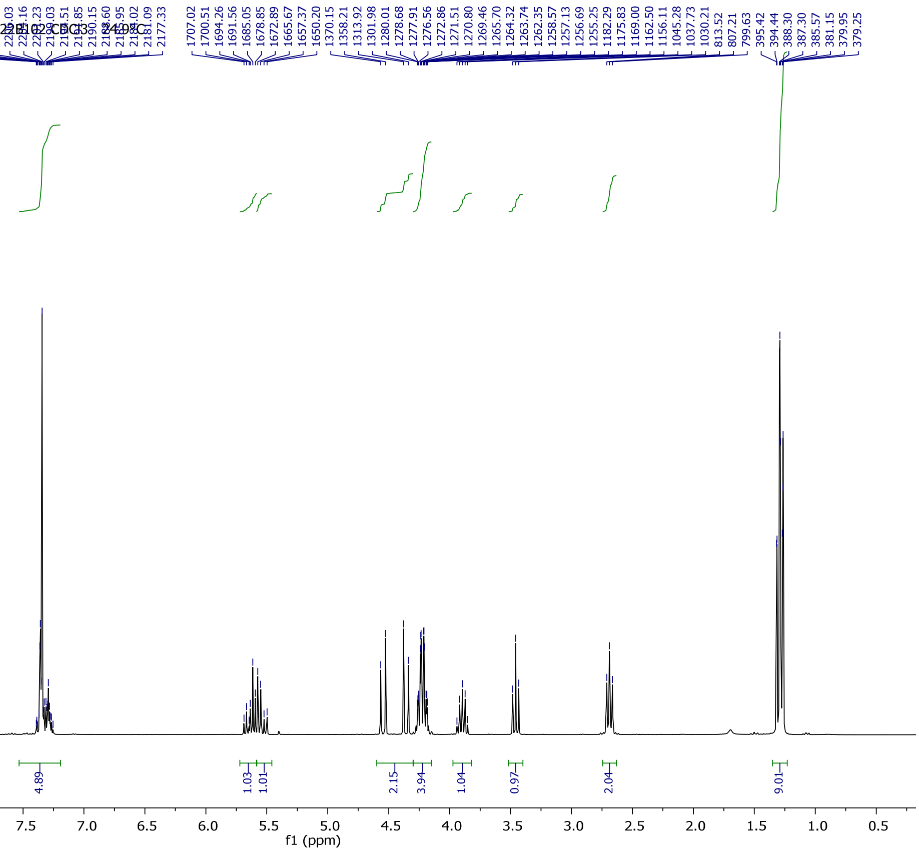
drsh22b102
1H 300.1MHz Job 54014 Sutherland
*
4.89
1.03
1.01
2.15
3.94
1.04
0.97
2.04
9.01
f1 (ppm)

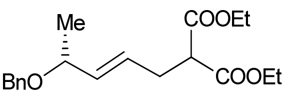
Me
COOEt
BnO
COOEt

6lb

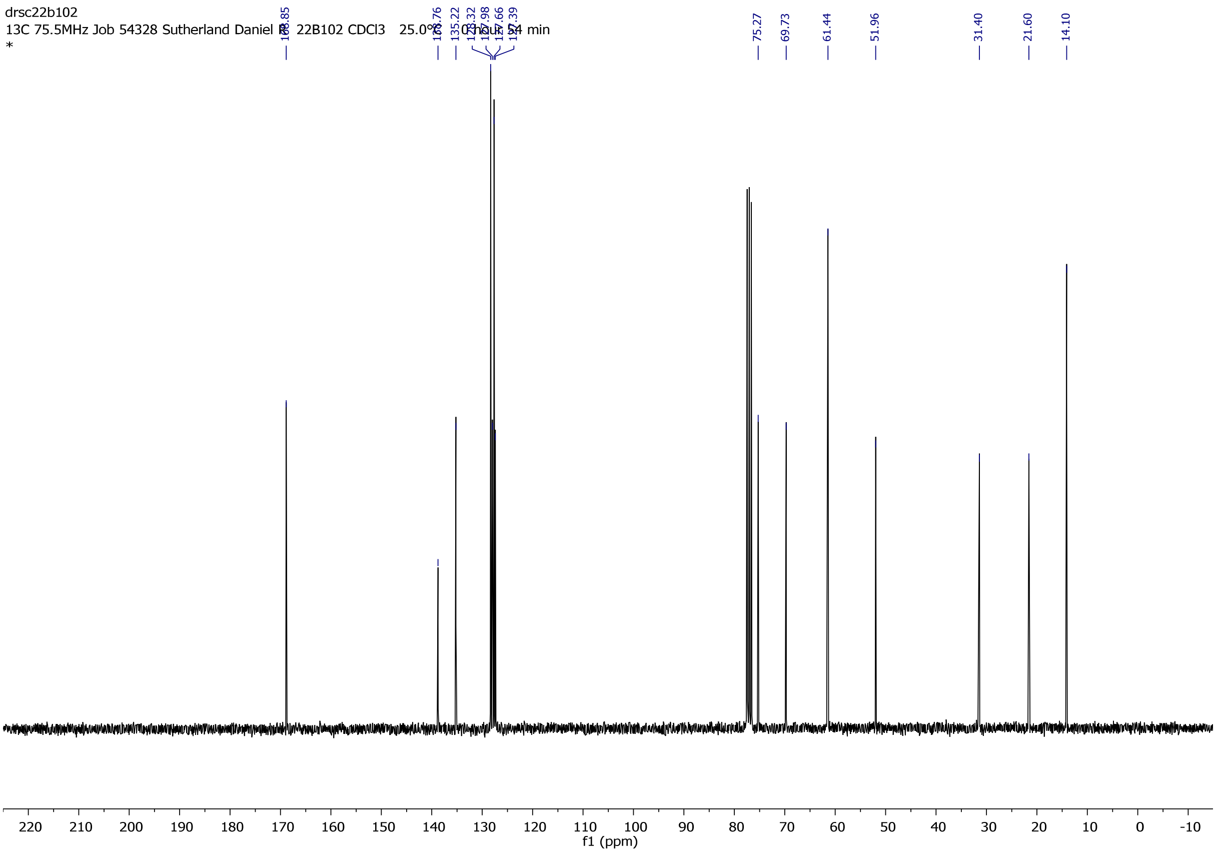
drsc22b102
13C 75.5MHz Job 54328 Sutherland Daniel
*
75.27
69.73
61.44
51.96
31.40
21.60
14.10
f1 (ppm)

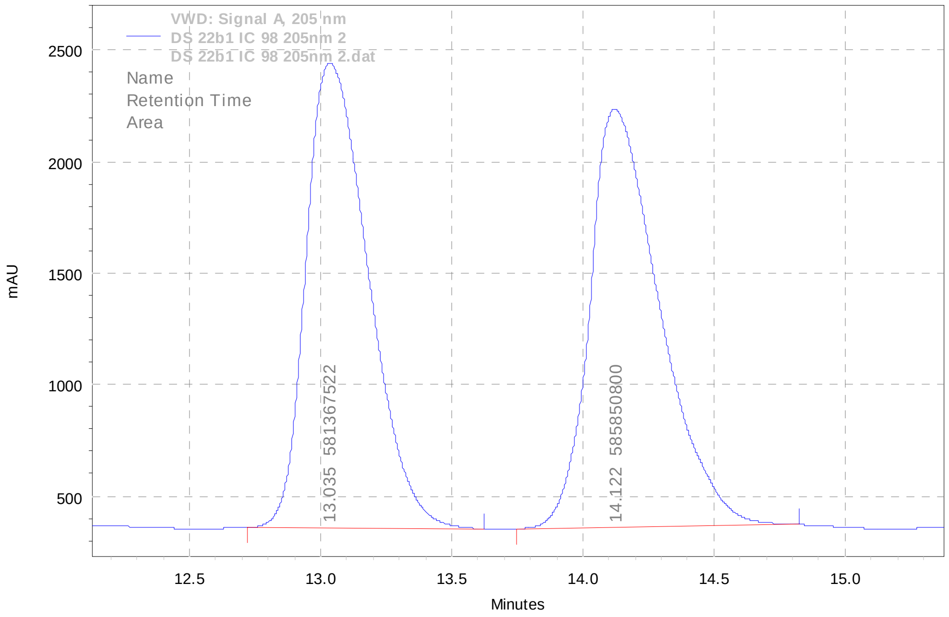

Retention Time	Area	Area %	Height	Height %
13.035	581367522	49.81	35020288	52.64
14.122	585850800	50.19	31509777	47.36
Totals	1167218322	100.00	66530065	100.00

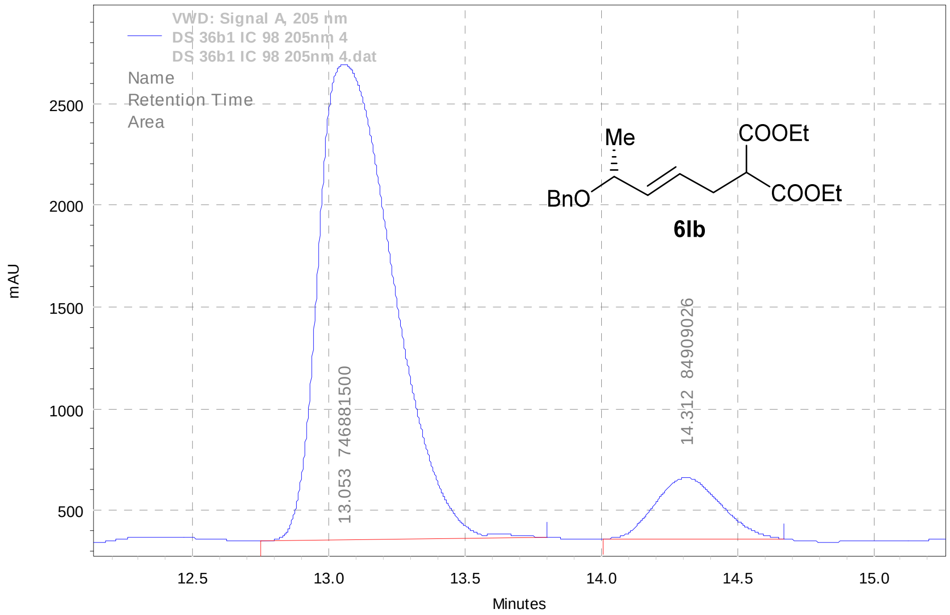

Retention Time	Area	Area %	Height	Height %
13.053	746881500	89.79	39220866	88.62
14.312	84909026	10.21	5038880	11.38
Totals	831790526	100.00	44259746	100.00

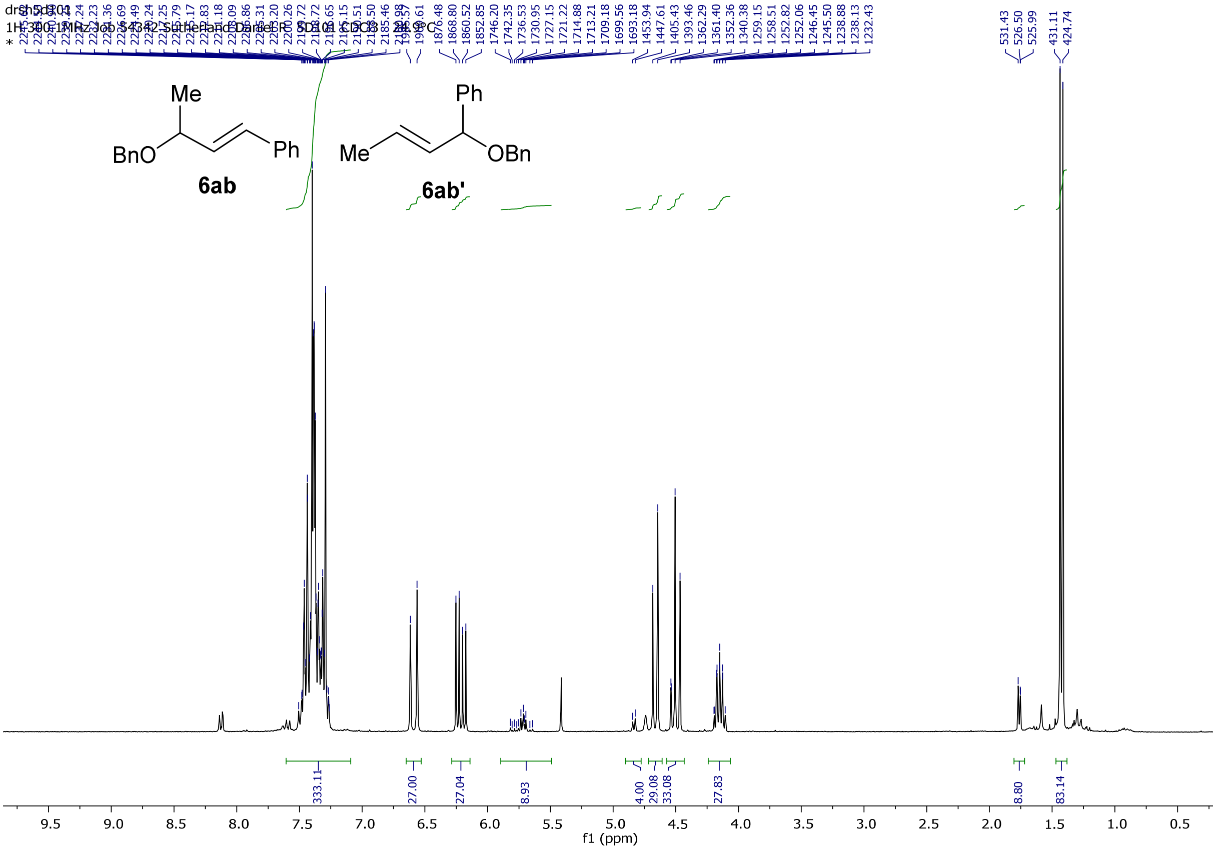
6ab
6ab'
f1 (ppm)

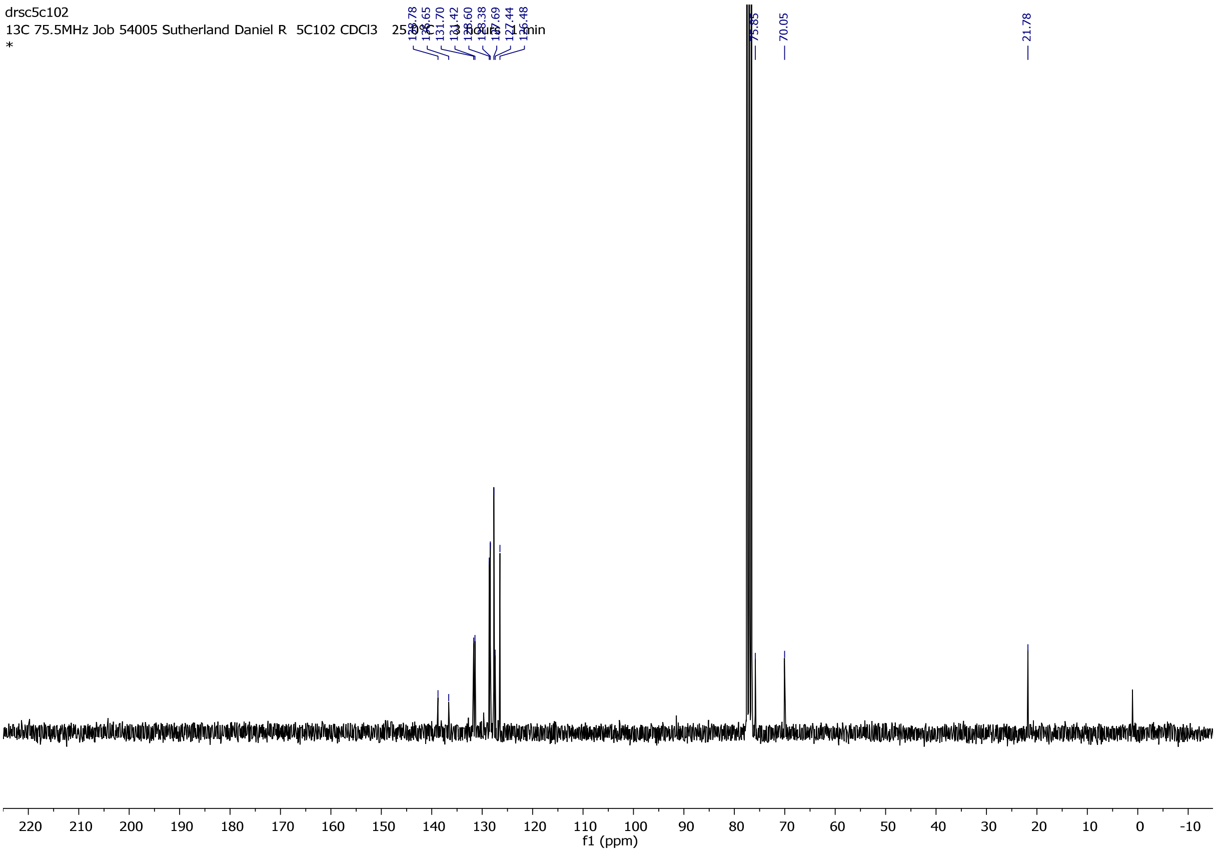
drsc5c102
75.85
70.05
21.78
f1 (ppm)

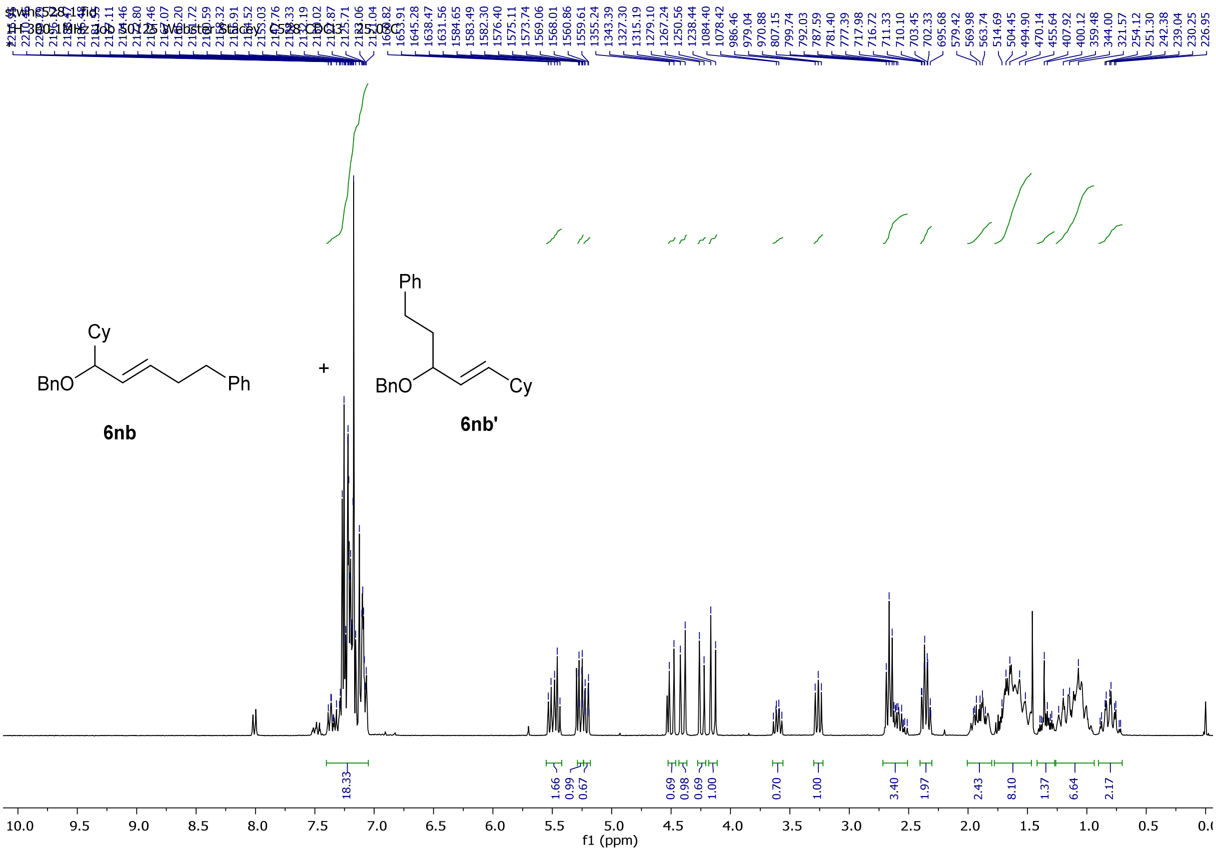
Cy
BnO
Ph
6nb
+
Ph
BnO
Cy
6nb'
18.33
1.66
0.99
0.67
0.69
0.98
0.69
1.00
0.70
1.00
3.40
1.97
2.43
8.10
1.37
6.64
2.17
f1 (ppm)

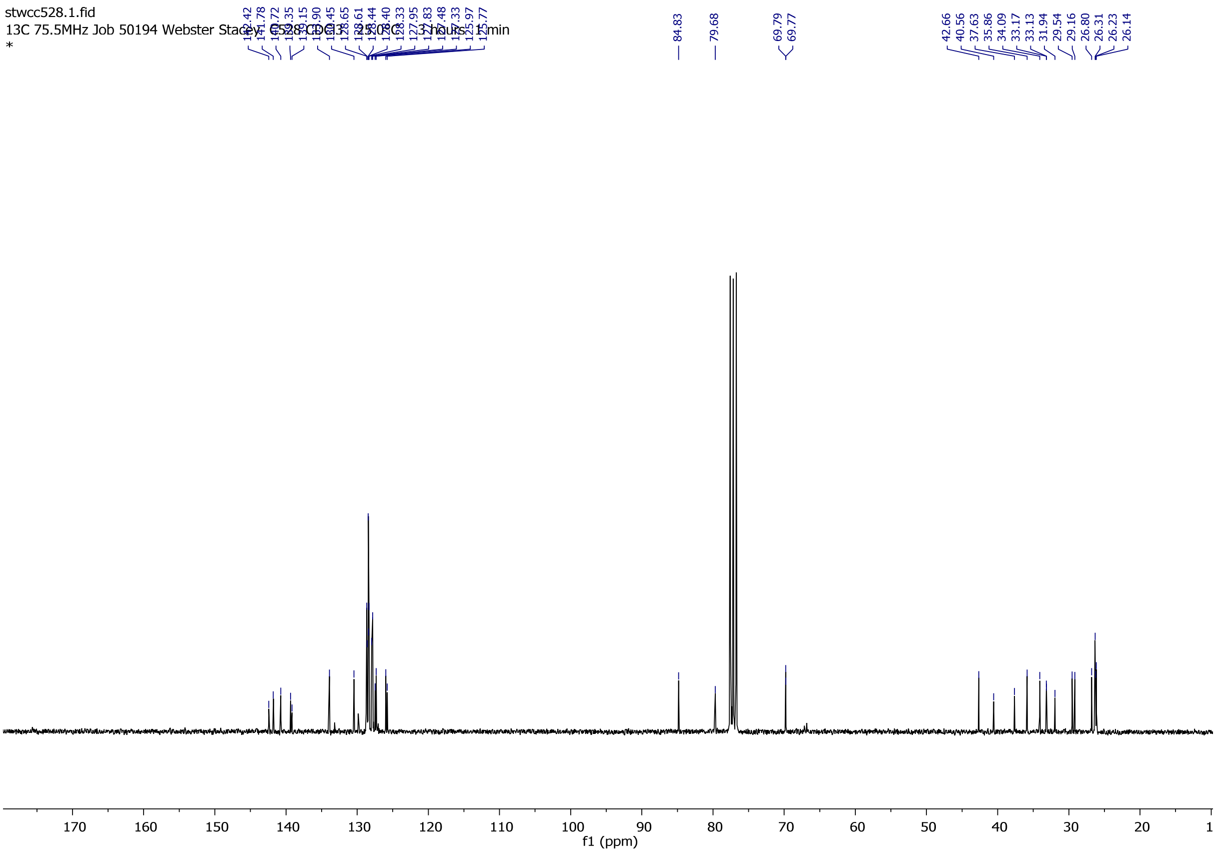
stwcc528.1.fid
13C 75.5MHz Job 50194 Webster Stacey C528 CDCl3 25.0°C 3 hours 1 min
*
84.83
79.68
69.79
69.77
42.66
40.56
37.63
35.86
34.09
33.17
33.13
31.94
29.54
29.16
26.80
26.31
26.23
26.14
f1 (ppm)

stwha627.1.fid
1H 300.1MHz Job 54183 Webster Stacey A627 CDCl3 25.0°C
*

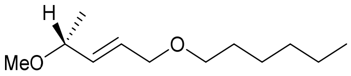

6oc

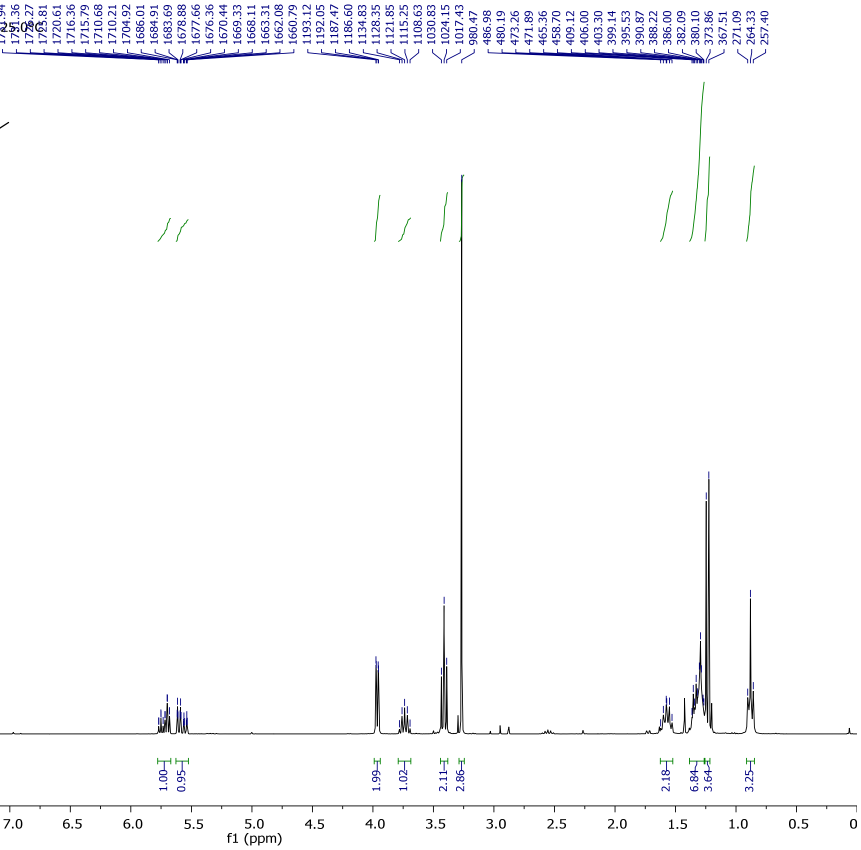

stwca627.1.fid
13C 75.5MHz Job 54252 Webster Stacey A627 CDCl3 25.0°C 2 hours 43 min
*

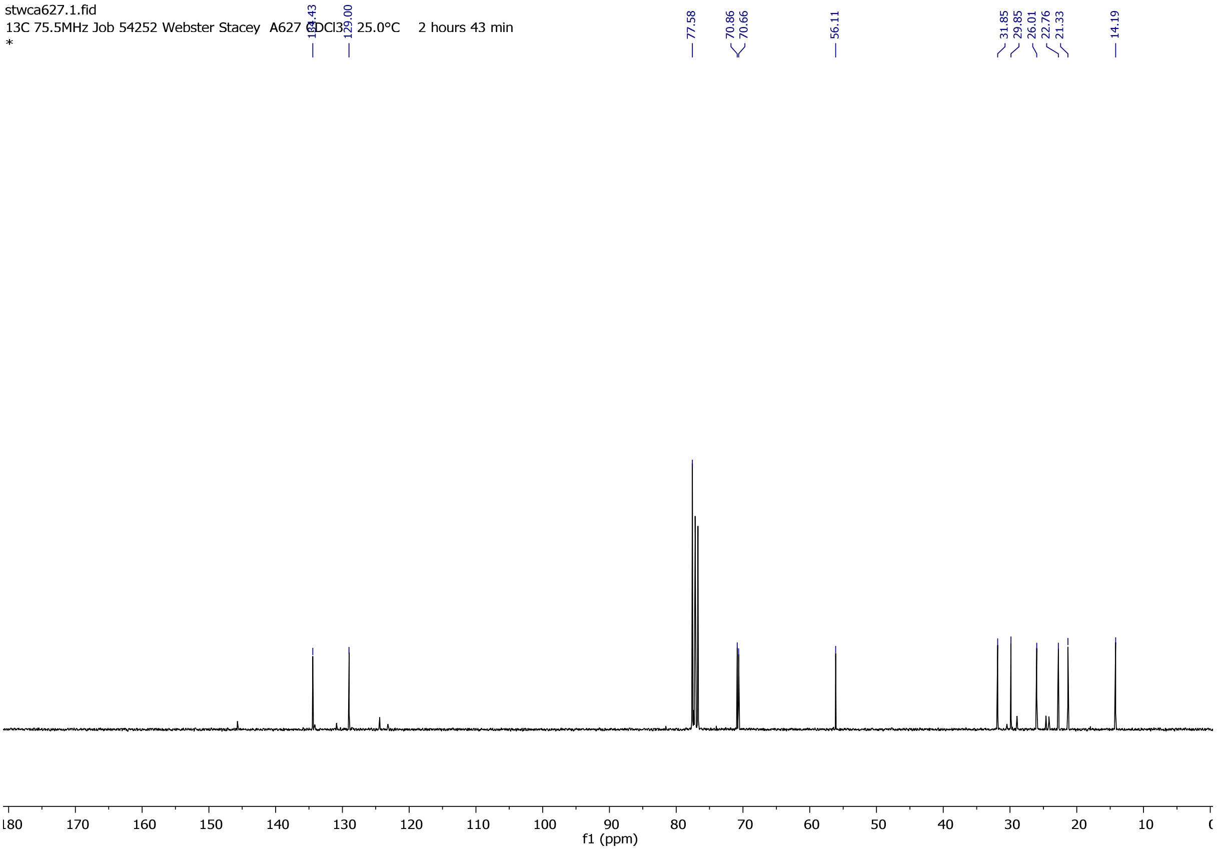

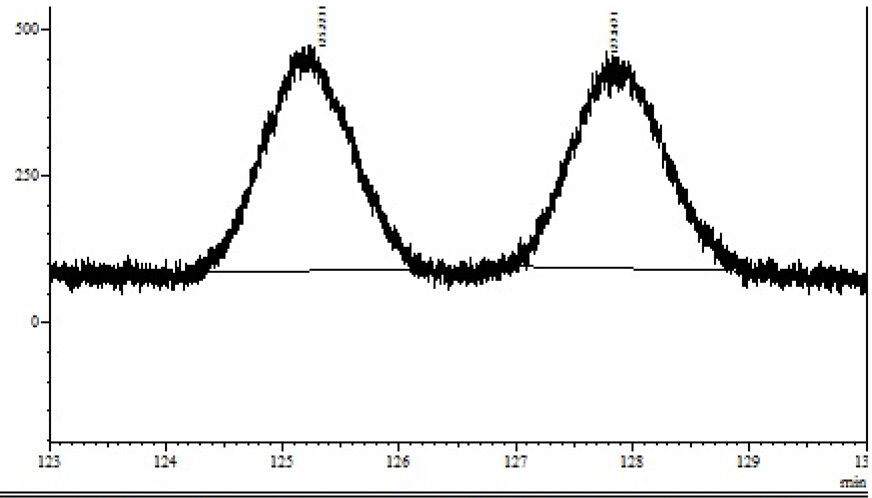

Peak#	Ret.Time	Area	Height	Conc.	Unit	Mark	ID#	Cmpd Name	Area%
1	125.223	19476	380	50.173					50.1725
2	127.847	19342	354	49.827					49.8275
**Total**		38818	734						100.0000

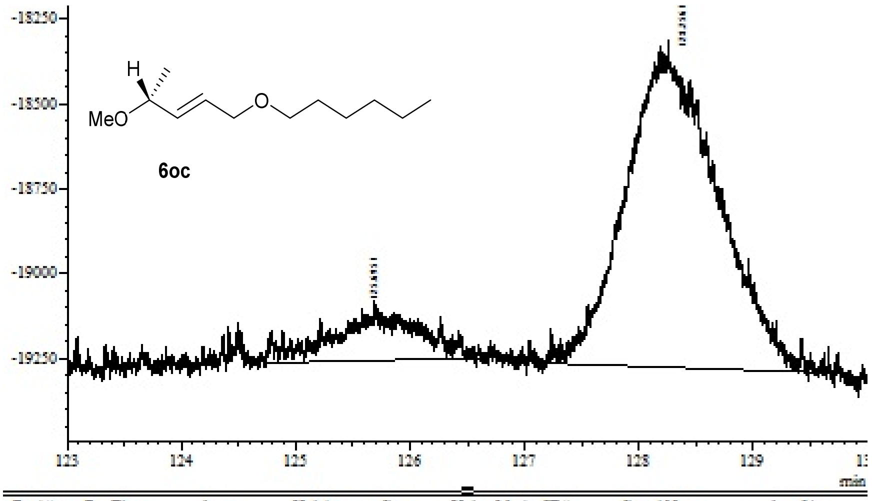

Peak#	Ret.Time	Area	Height	Conc.	Unit	Mark	ID#	Cmpd Name	Area%
1	125.695	7916	161	13.078					13.0777
2	128.256	52614	956	86.922					86.9223
**Total**		60530	1117						100.0000

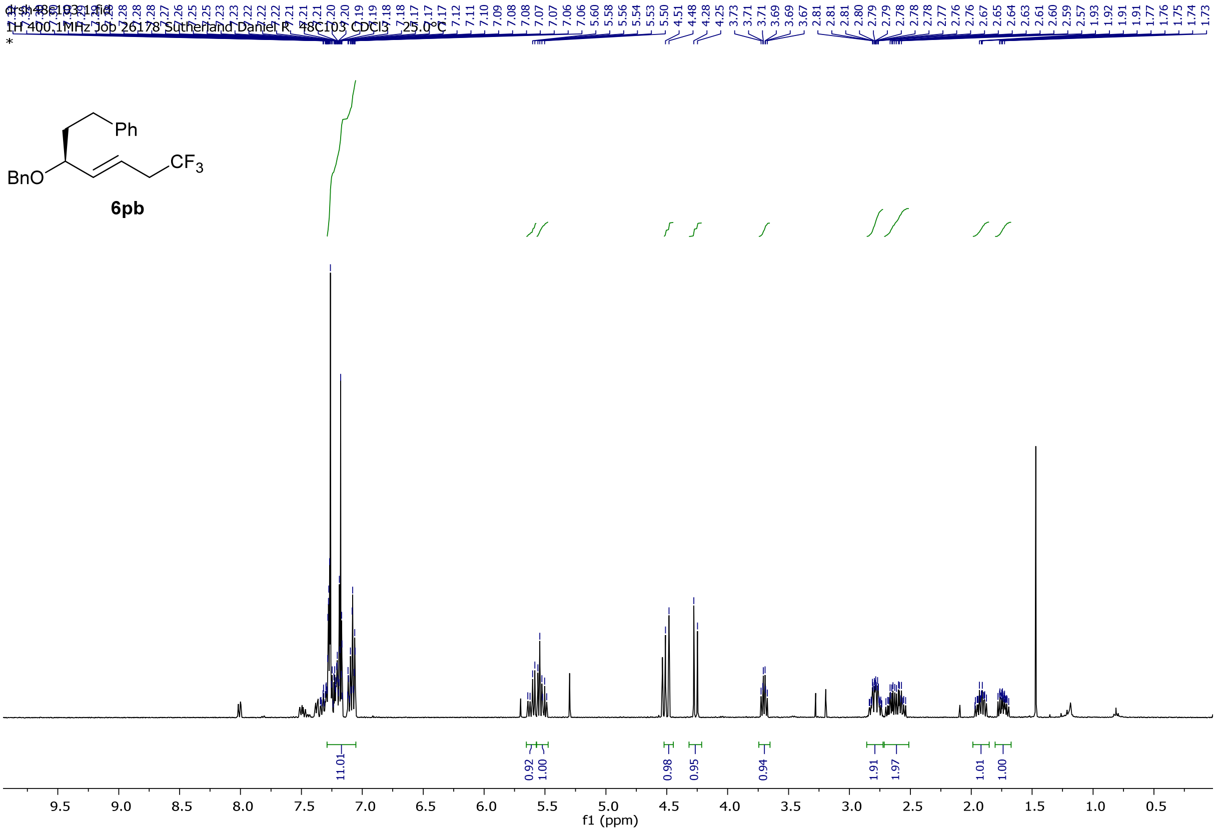
1H 400.1MHz Job 26178 Sutherland Daniel R 48C103 CDCl3 25.0°C
Ph
BnO
CF3
6pb
f1 (ppm)

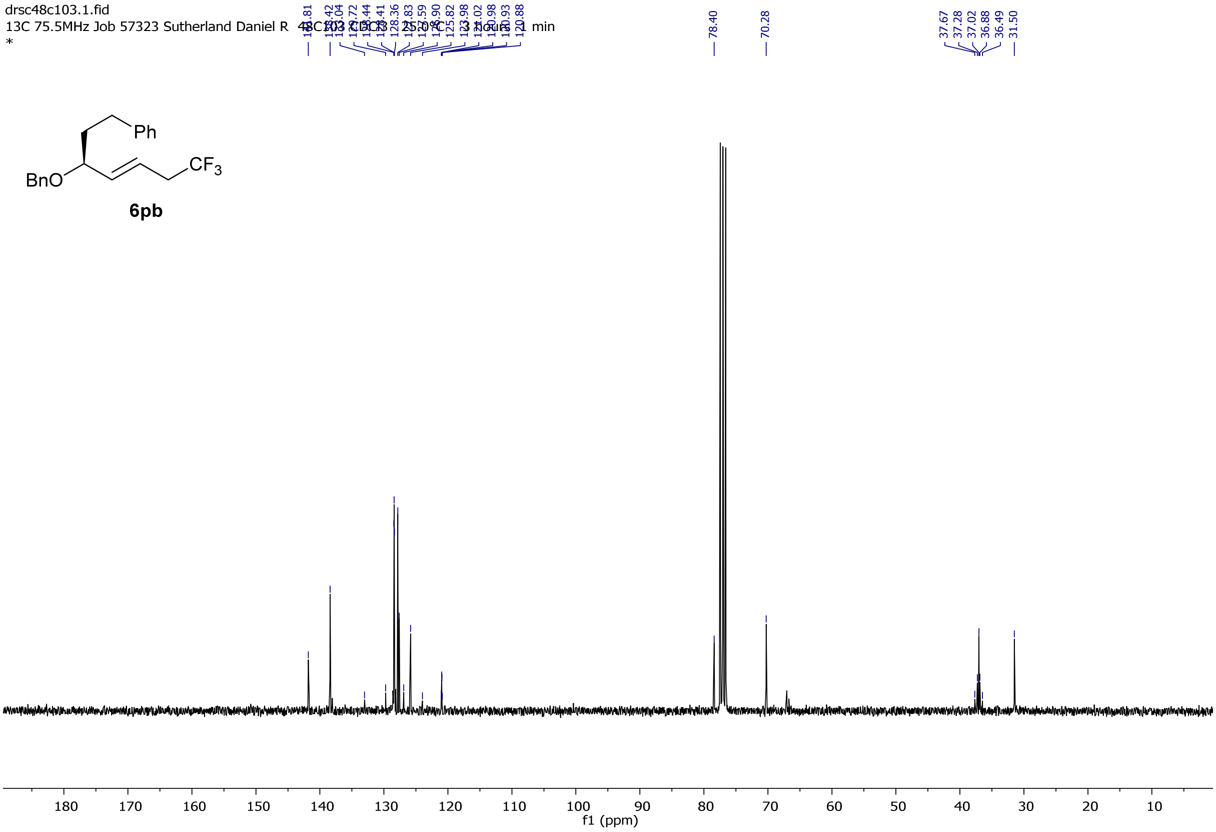
drsc48c103.1.fid
13C 75.5MHz Job 57323 Sutherland Daniel R
78.40
70.28
37.67
37.28
37.02
36.88
36.49
31.50
Ph
BnO
CF3
6pb
f1 (ppm)

COSY 2D NMR

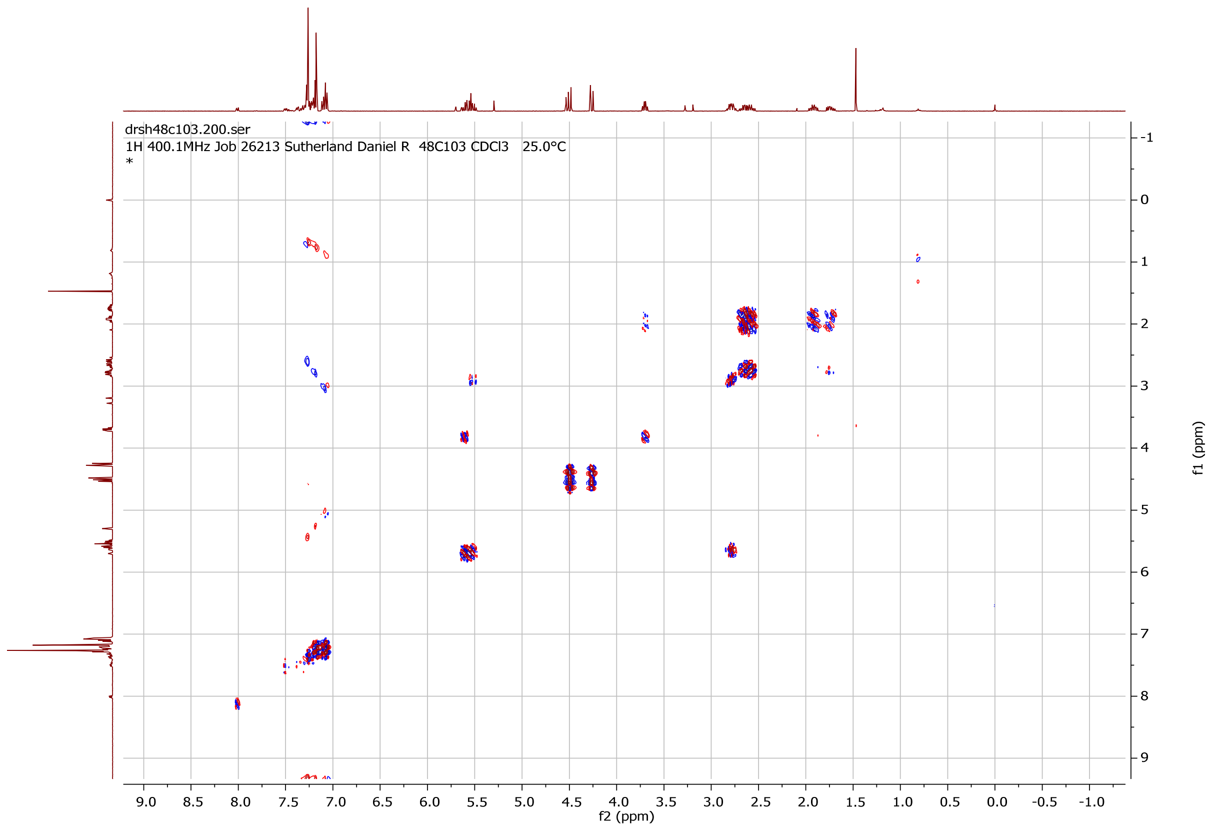

COSY NMR was used to confirm the structure of 6bp.

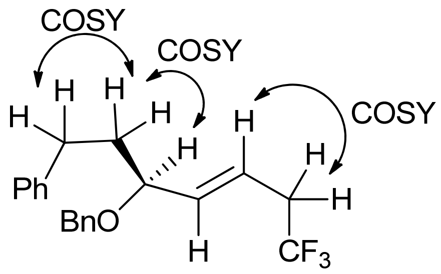

6pb

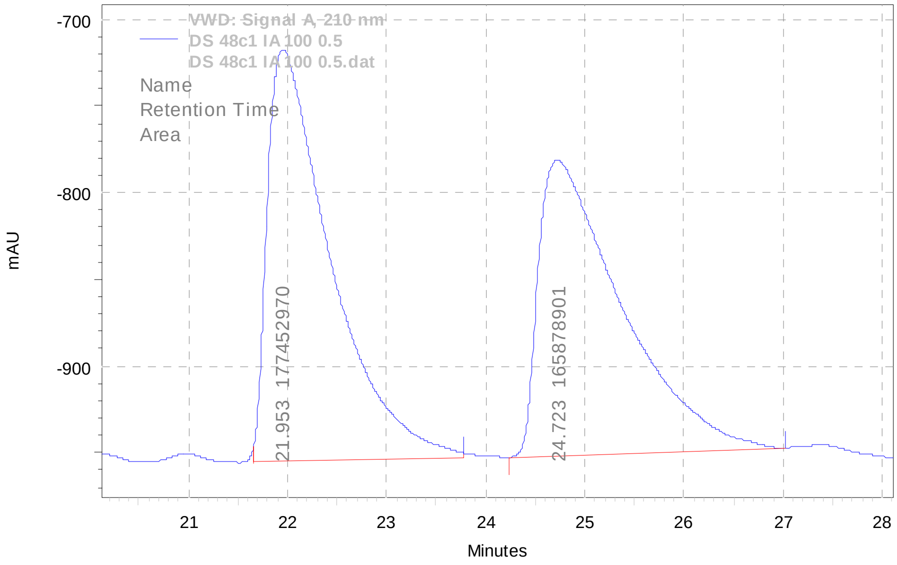

Retention Time	Area	Area %	Height	Height %
21.953	177452970	51.69	3990112	58.29
24.723	165878901	48.31	2855345	41.71
Totals	343331871	100.00	6845457	100.00

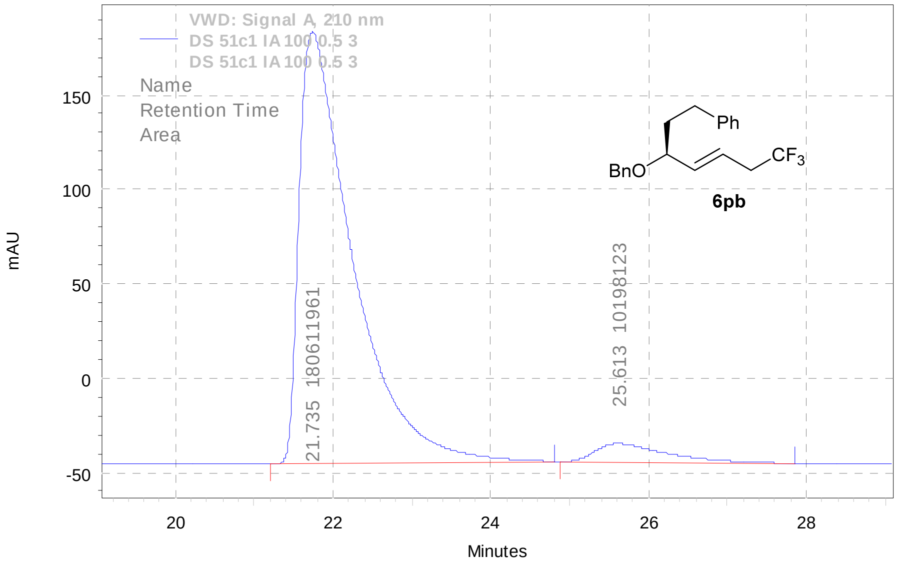

Retention Time	Area	Area %	Height	Height %
21.735	180611961	94.66	3835663	95.93
25.613	10198123	5.34	162799	4.07
Totals	190810084	100.00	3998462	100.00

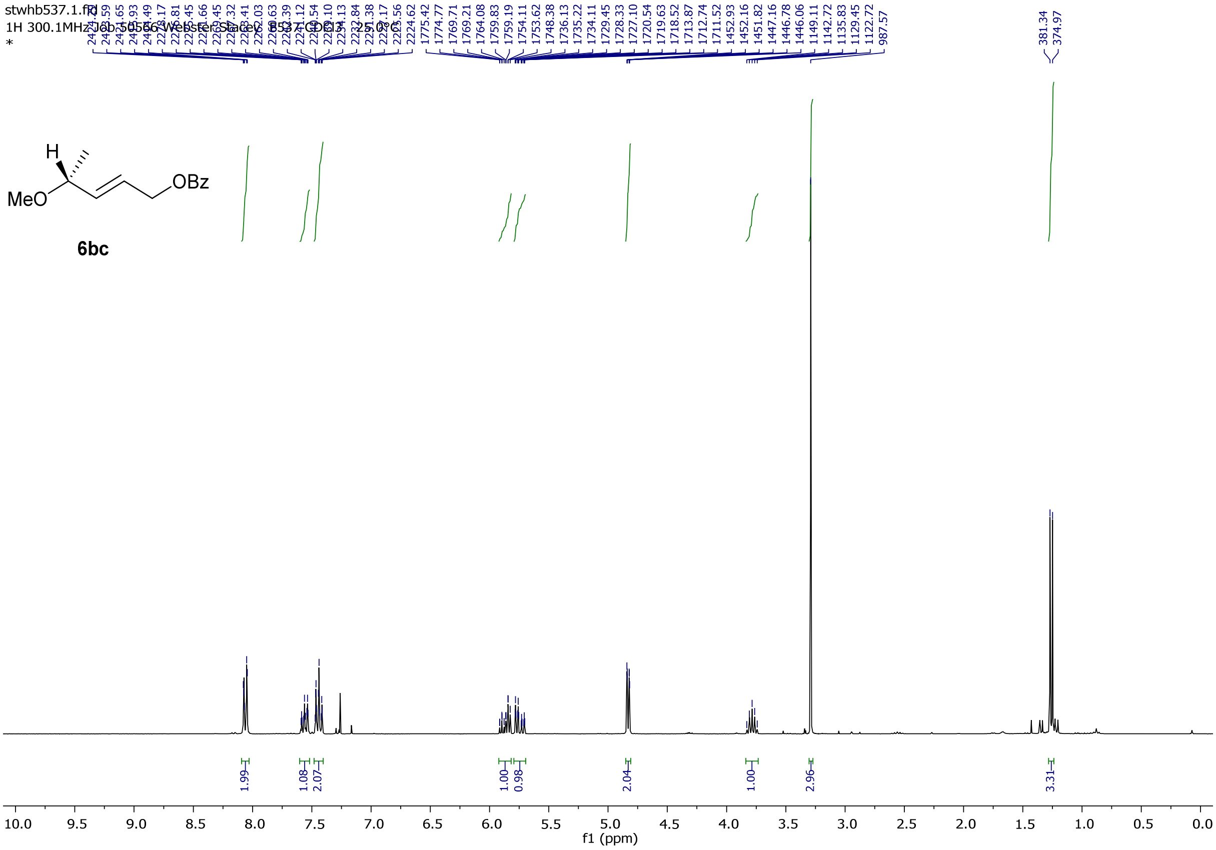
stwhb537.1.fid
1H 300.1MHz
*
381.34
374.97
H
MeO
OBz
6bc
1.99
1.08
2.07
1.00
0.98
2.04
1.00
2.96
3.31
10.0 9.5 9.0 8.5 8.0 7.5 7.0 6.5 6.0 5.5 5.0 4.5 4.0 3.5 3.0 2.5 2.0 1.5 1.0 0.5 0.0
f1 (ppm)

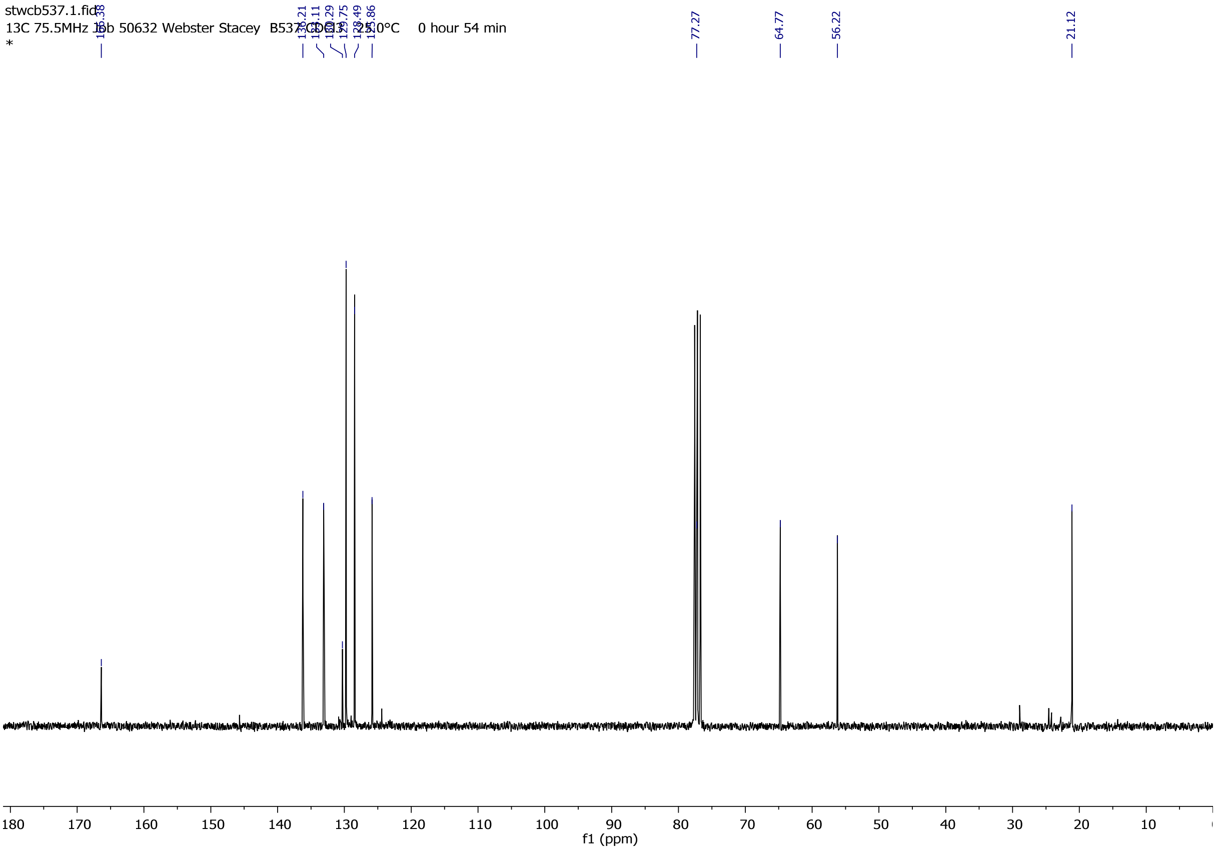
stwcb537.1.fid
13C 75.5MHz Job 50632 Webster Stacey B537 CDCl3 25.0°C 0 hour 54 min
*
77.27
64.77
56.22
21.12
180 170 160 150 140 130 120 110 100 90 80 70 60 50 40 30 20 10
f1 (ppm)

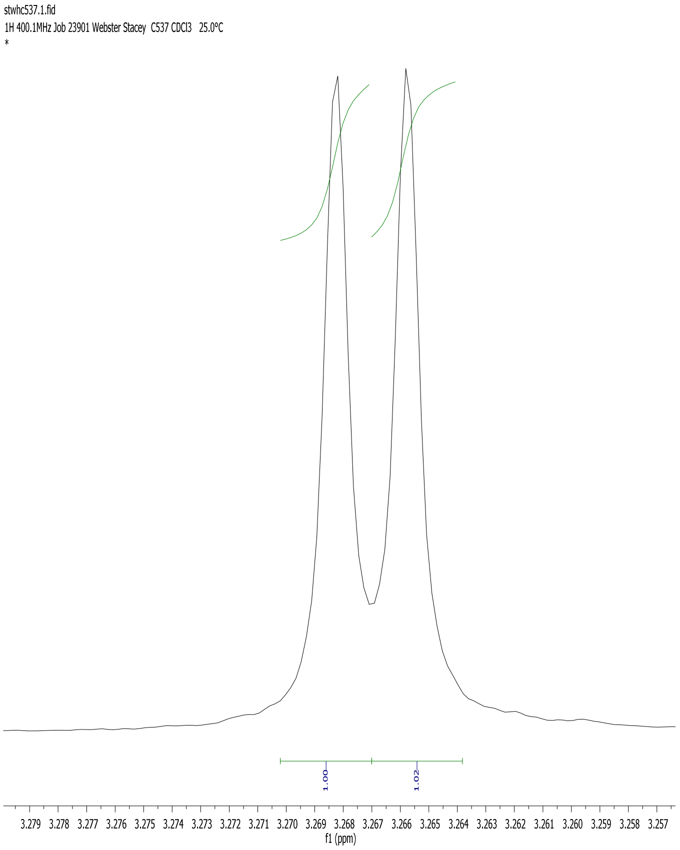
stwhc537.1.fid
1H 400.1MHz Job 23901 Webster Stacey C537 CDCl3 25.0°C
*
1.00
1.02
3.279 3.278 3.277 3.276 3.275 3.274 3.273 3.272 3.271 3.270 3.269 3.268 3.267 3.266 3.265 3.264 3.263 3.262 3.261 3.260 3.259 3.258 3.257
f1 (ppm)

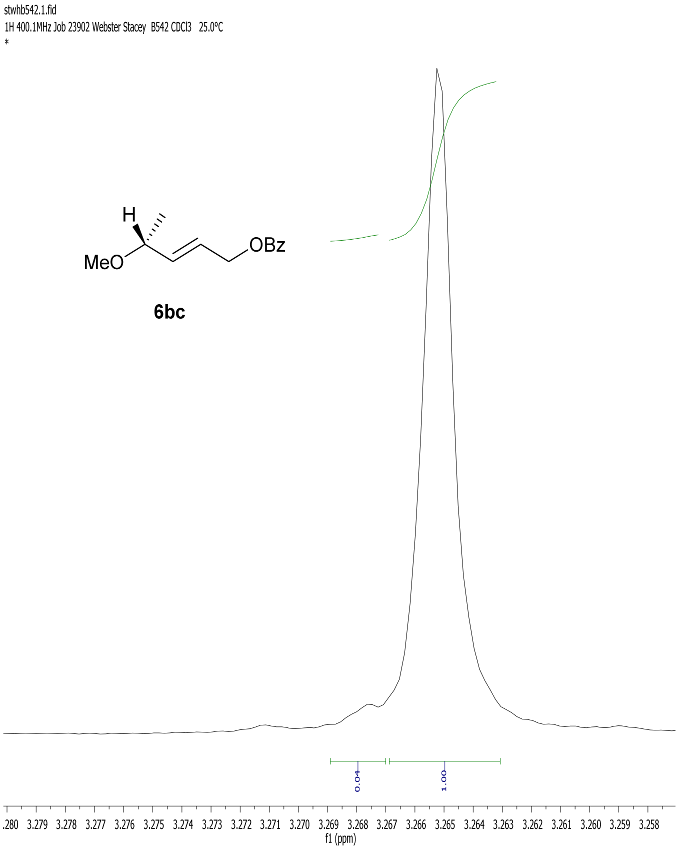
stwhb542.1.fid
1H 400.1MHz Job 23902 Webster Stacey B542 CDCl3 25.0°C
*
H
MeO
OBz
6bc
0.04
1.00
.280 3.279 3.278 3.277 3.276 3.275 3.274 3.273 3.272 3.271 3.270 3.269 3.268 3.267 3.266 3.265 3.264 3.263 3.262 3.261 3.260 3.259 3.258
f1 (ppm)

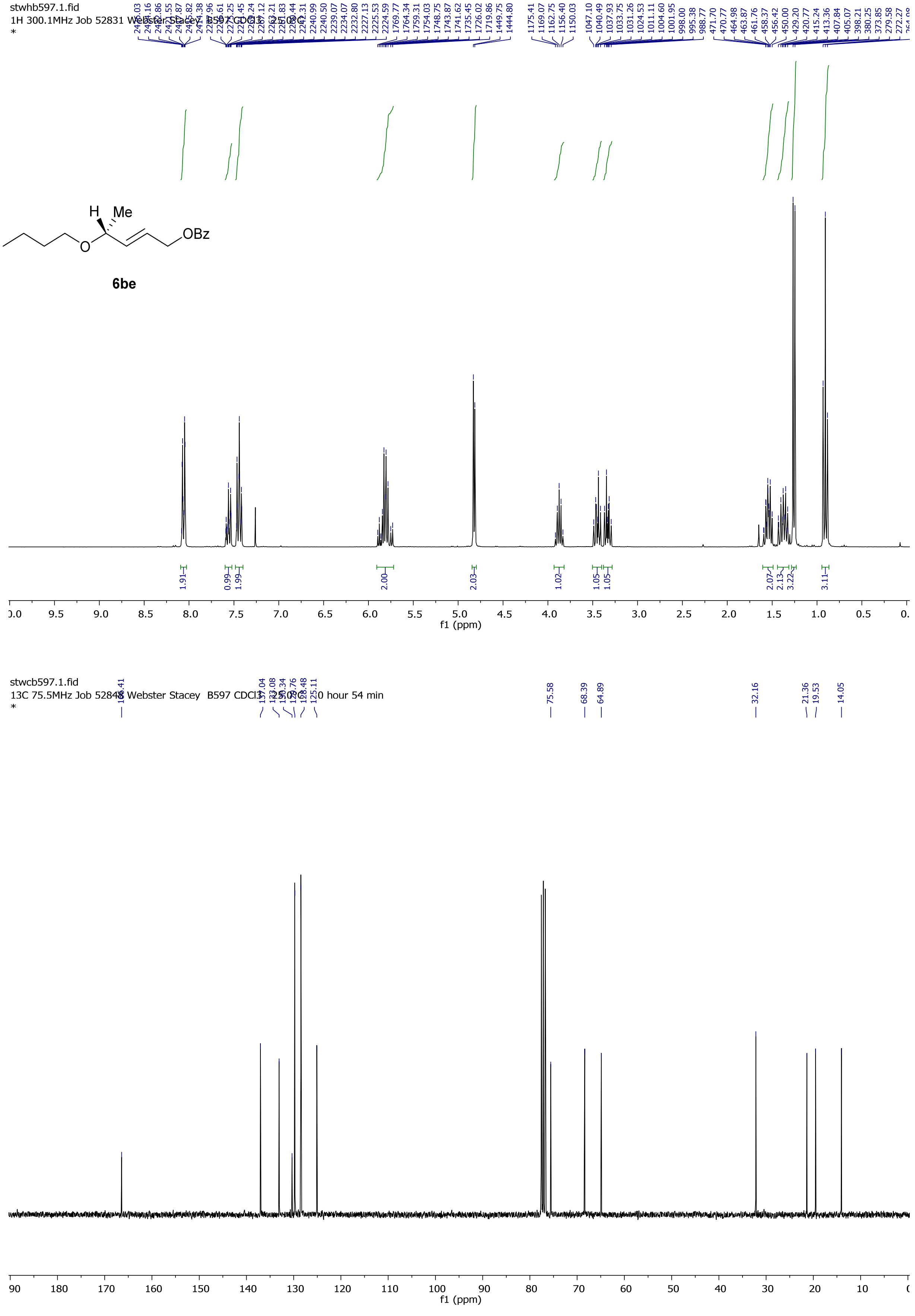
stwhb597.1.fid
1H 300.1MHz Job 52831 Webster Stacey B597 CDCl3
OBz
6be
f1 (ppm)
stwcb597.1.fid
13C 75.5MHz Job 52848 Webster Stacey B597 CDCl3
166.41
137.04
133.08
130.34
129.76
128.48
125.11
75.58
68.39
64.89
32.16
21.36
19.53
14.05
f1 (ppm)

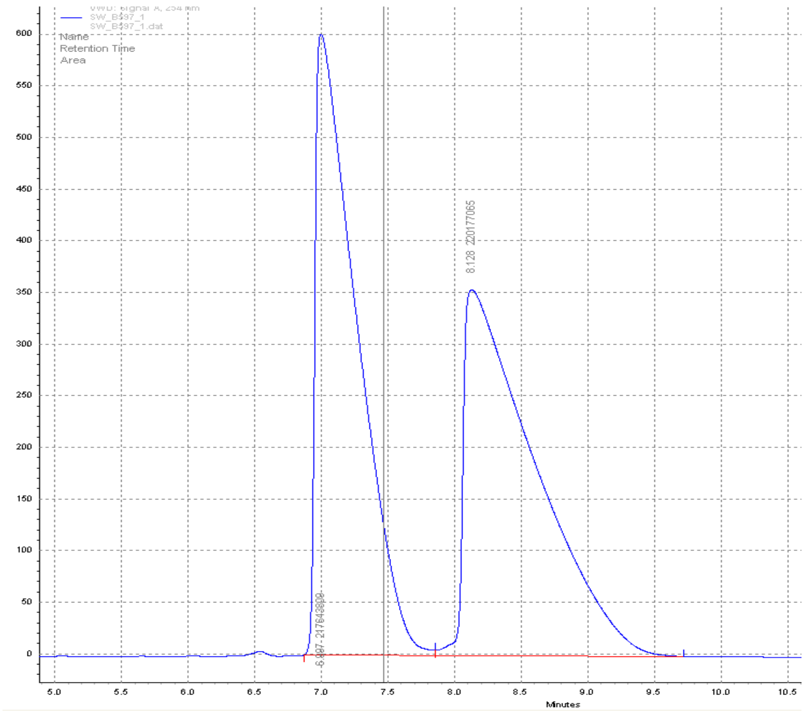

Retention Time	Area	Area %	Height	Height %
6.997	217643809	49.71	10084386	62.92
8.128	220177065	50.29	5943620	37.08
Totals	437820874	100.00	16028006	100.00

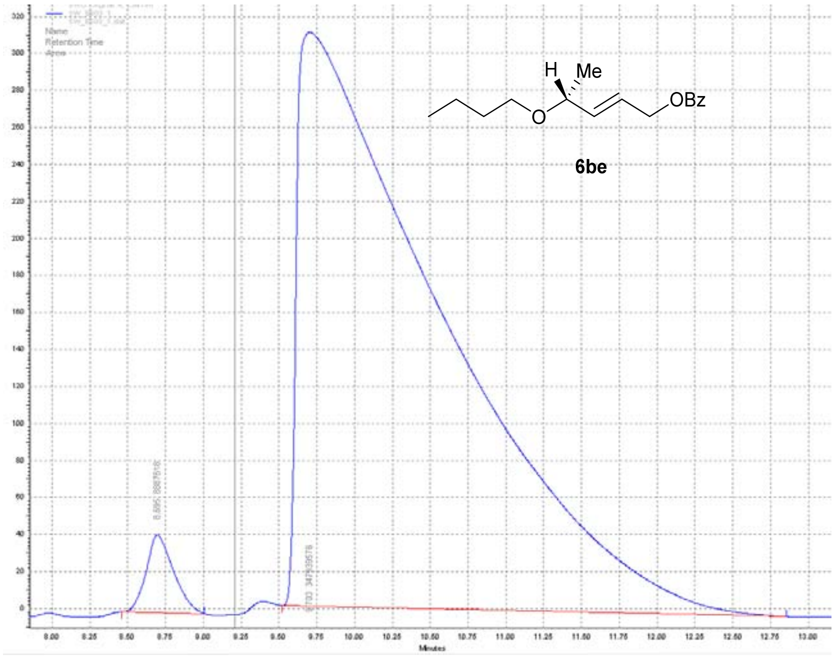

Retention Time	Area	Area %	Height	Height %
8.695	8887618	2.49	704496	11.92
9.703	347939576	97.51	5203709	88.08
Totals	356827194	100.00	5908205	100.00

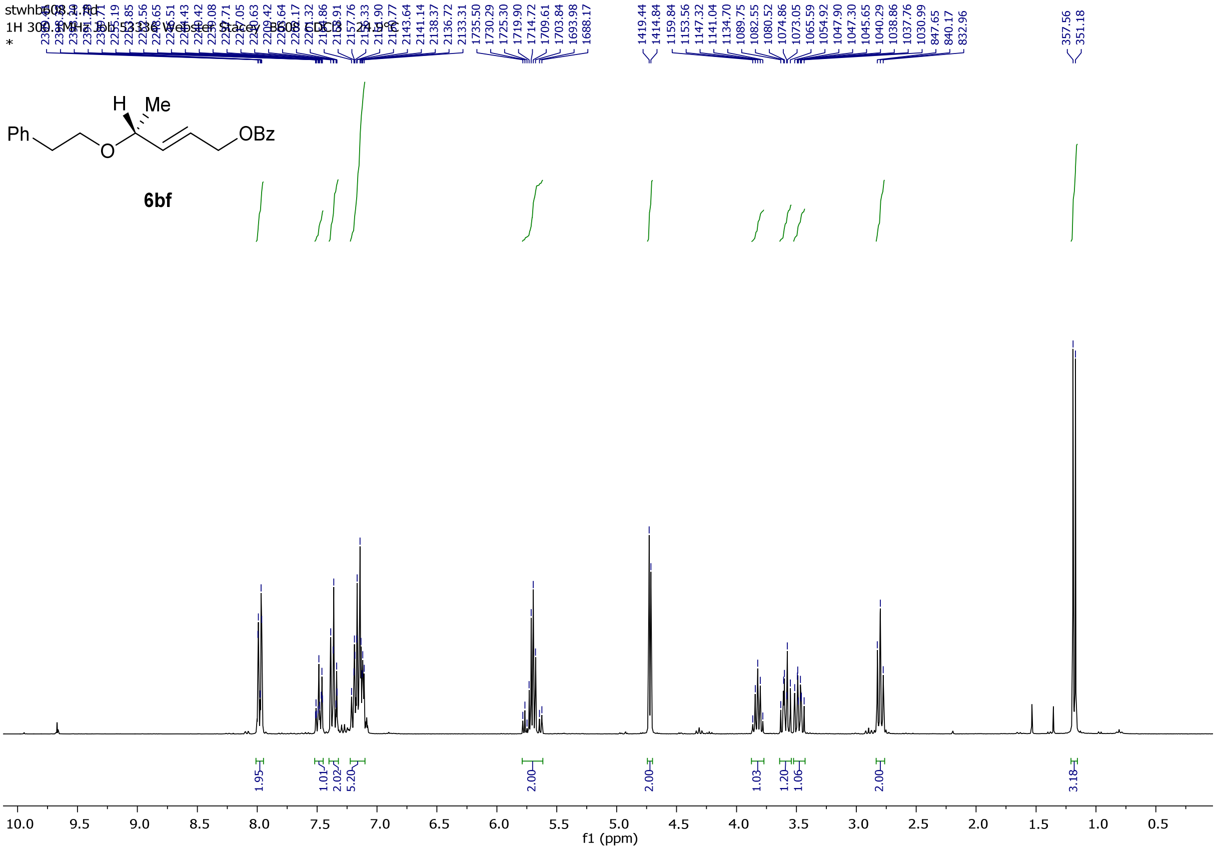
1H 300.1MHz
*
1735.50
1730.29
1725.30
1719.90
1714.72
1709.61
1703.84
1693.98
1688.17
1419.44
1414.84
1159.84
1153.56
1147.32
1141.04
1134.70
1089.75
1082.55
1080.52
1074.86
1073.05
1065.59
1054.92
1047.90
1047.30
1045.65
1040.29
1038.86
1037.76
1030.99
847.65
840.17
832.96
357.56
351.18
H
Me
Ph
O
OBz
6bf
1.95
1.01
2.02
5.20
2.00
2.00
1.03
1.20
1.06
2.00
3.18
10.0
9.5
9.0
8.5
8.0
7.5
7.0
6.5
6.0
5.5
5.0
4.5
4.0
3.5
3.0
2.5
2.0
1.5
1.0
0.5
f1 (ppm)

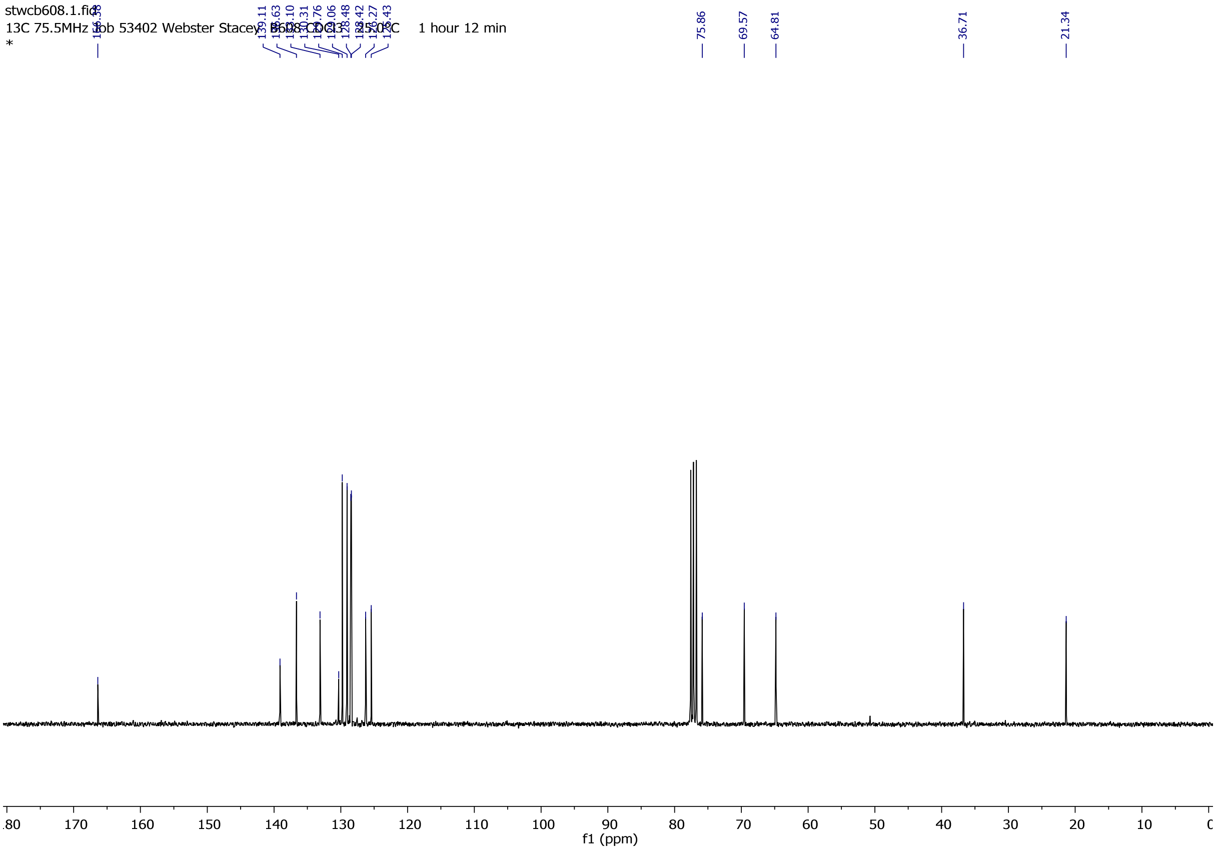
stwcb608.1.fid
13C 75.5MHz Job 53402 Webster Stacey B608 CDCl3 25.0°C 1 hour 12 min
*
75.86
69.57
64.81
36.71
21.34
170
160
150
140
130
120
110
100
90
80
70
60
50
40
30
20
10
f1 (ppm)

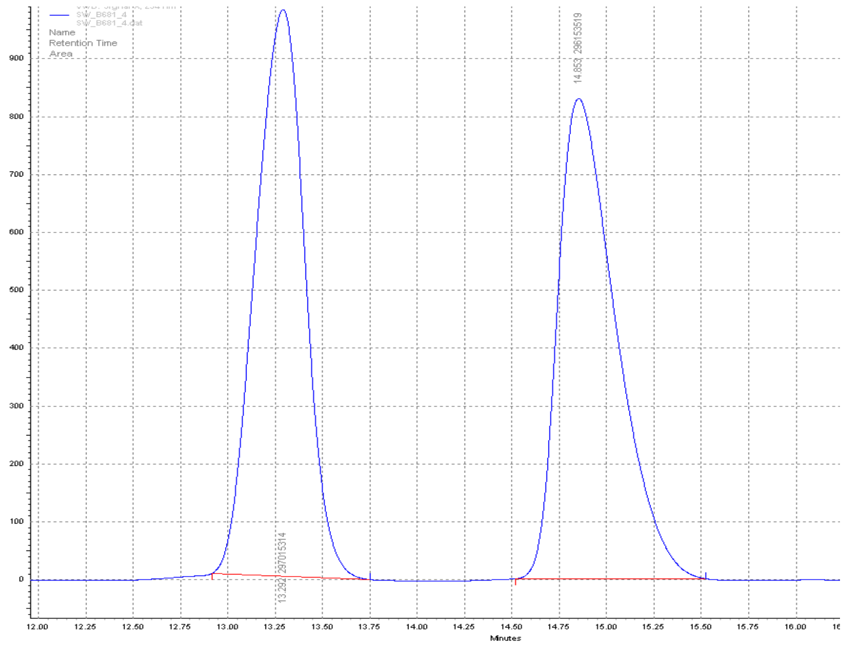

Retention Time	Area	Area %	Height	Height %
13.292	297015314	50.07	16418111	54.11
14.853	296153519	49.93	13921553	45.89

Totals				
	593168833	100.00	30339664	100.00

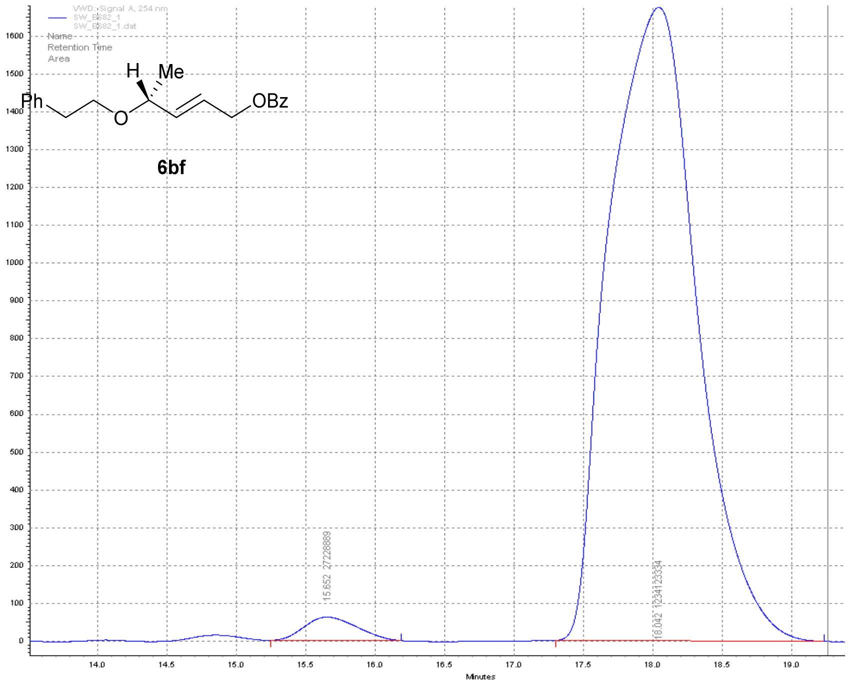

Retention Time	Area	Area %	Height	Height %
15.652	27228889	2.16	1034785	3.55
18.042	1234123334	97.84	28109165	96.45

Totals				
	1261352223	100.00	29143950	100.00

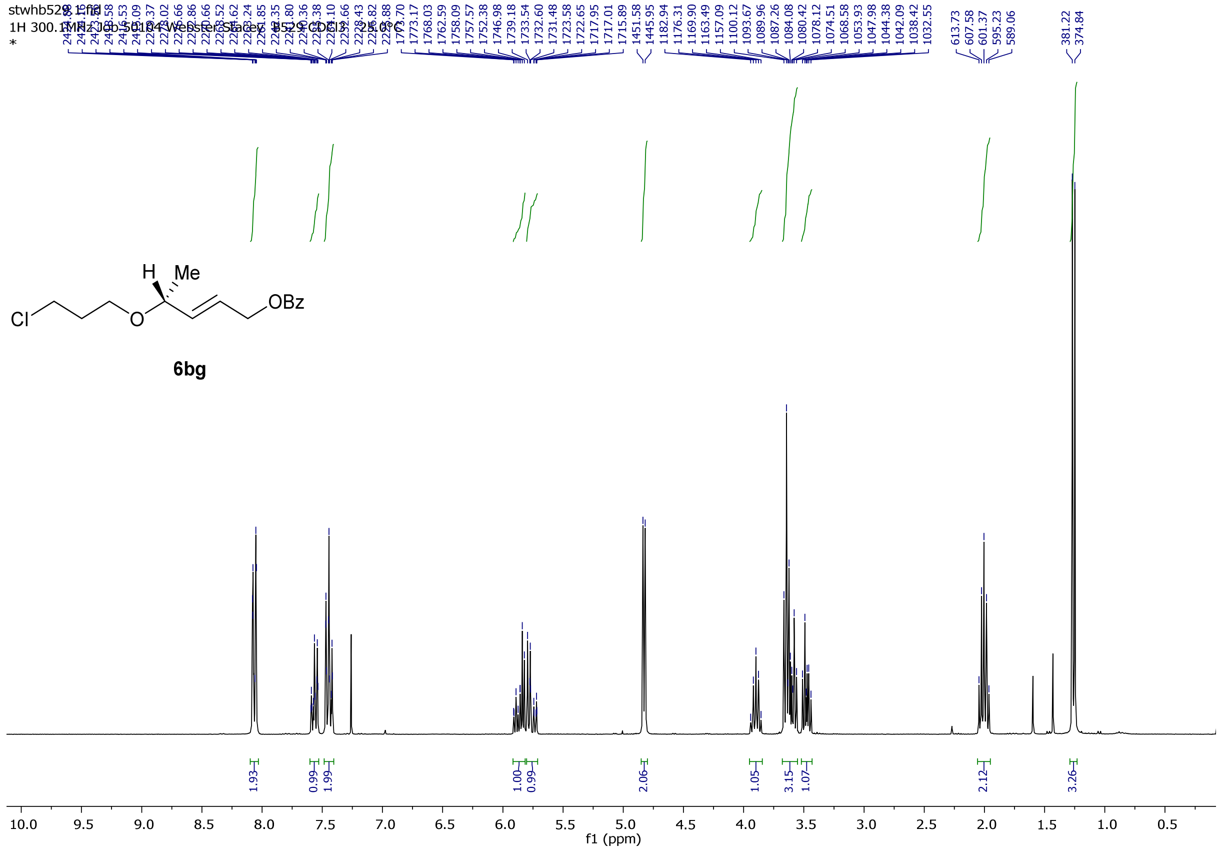
stwhb529.1.fid
1H 300.1MHz
*
1.93
0.99
1.99
1.00
0.99
2.06
1.05
3.15
1.07
2.12
3.26
f1 (ppm)
H
Me
Cl
O
OBz
6bg

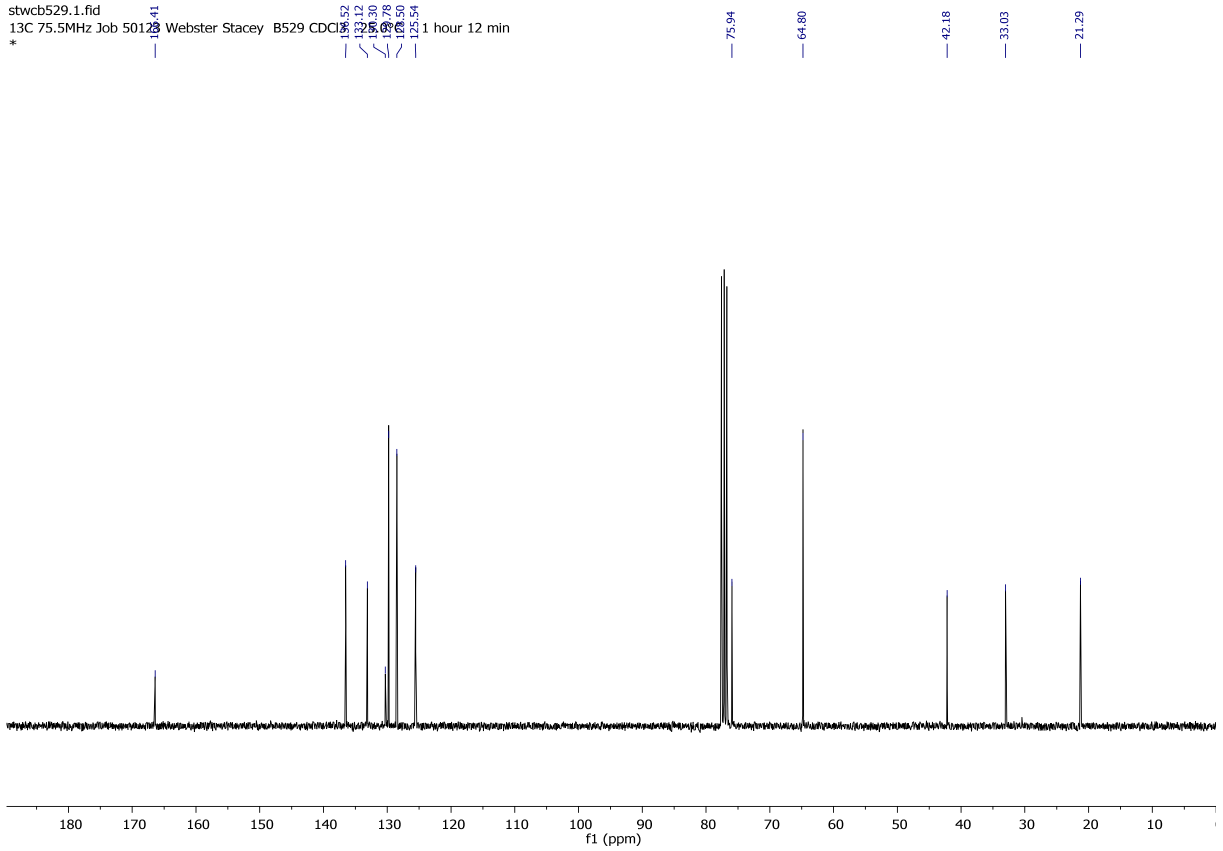
stwcb529.1.fid
13C 75.5MHz Job 50123 Webster Stacey B529 CDCl3
1 hour 12 min
*
133.12
125.54
75.94
64.80
42.18
33.03
21.29
f1 (ppm)

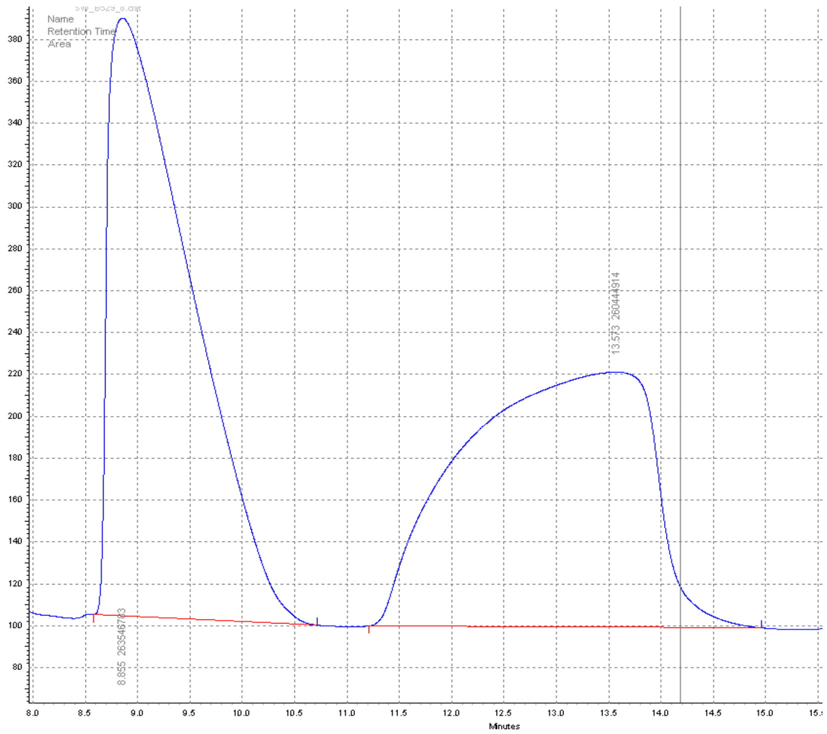

Retention Time	Area	Area %	Height	Height %
8.855	263546783	50.30	4790751	70.10
13.573	260444914	49.70	2043272	29.90
Totals	523991697	100.00	6834023	100.00

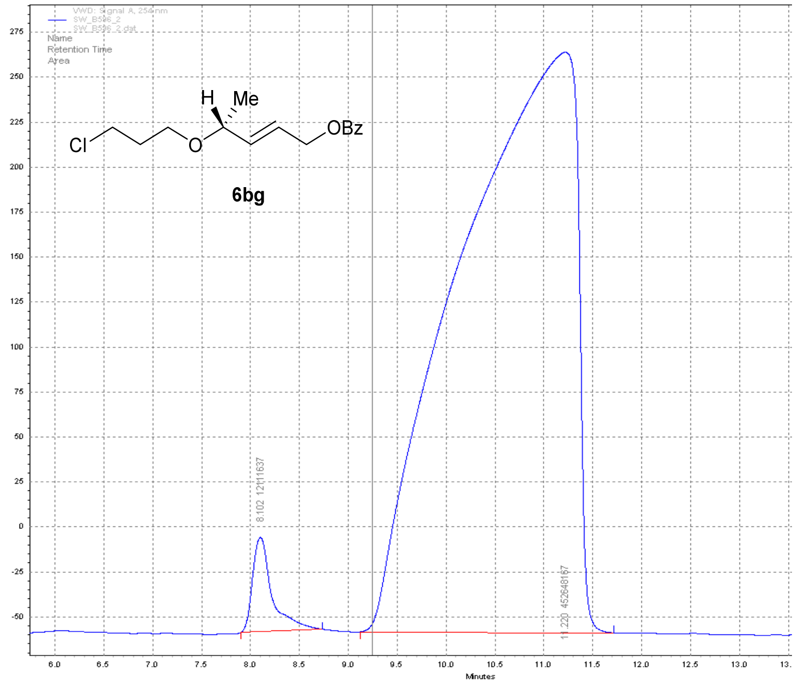

Retention Time	Area	Area %	Height	Height %
8.102	12111637	2.61	877962	13.95
11.220	452648167	97.39	5414167	86.05
Totals	464759804	100.00	6292129	100.00

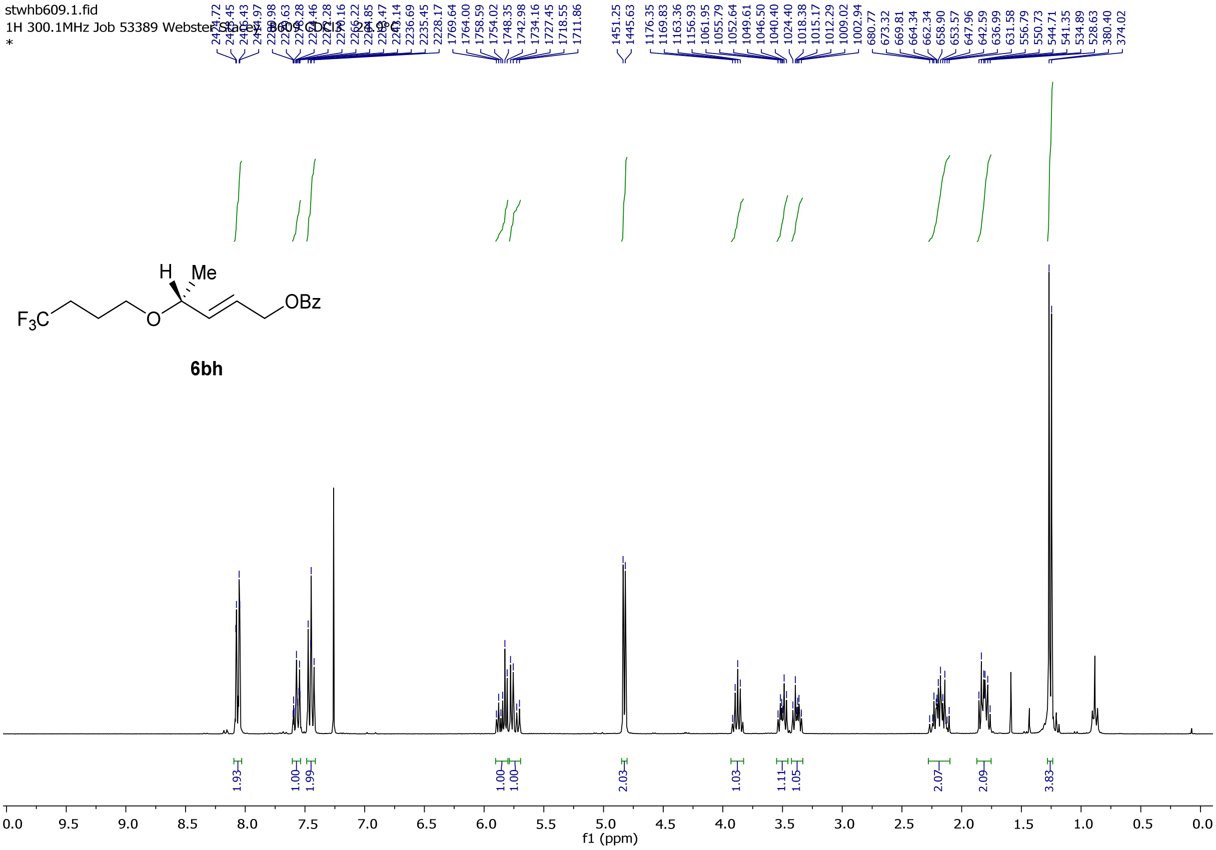
stwhb609.1.fid
1H 300.1MHz Job 53389 Webster Stacey B609 CDCl3 25.0°C
H
Me
F_3C
O
OBz
f1 (ppm)

6bh

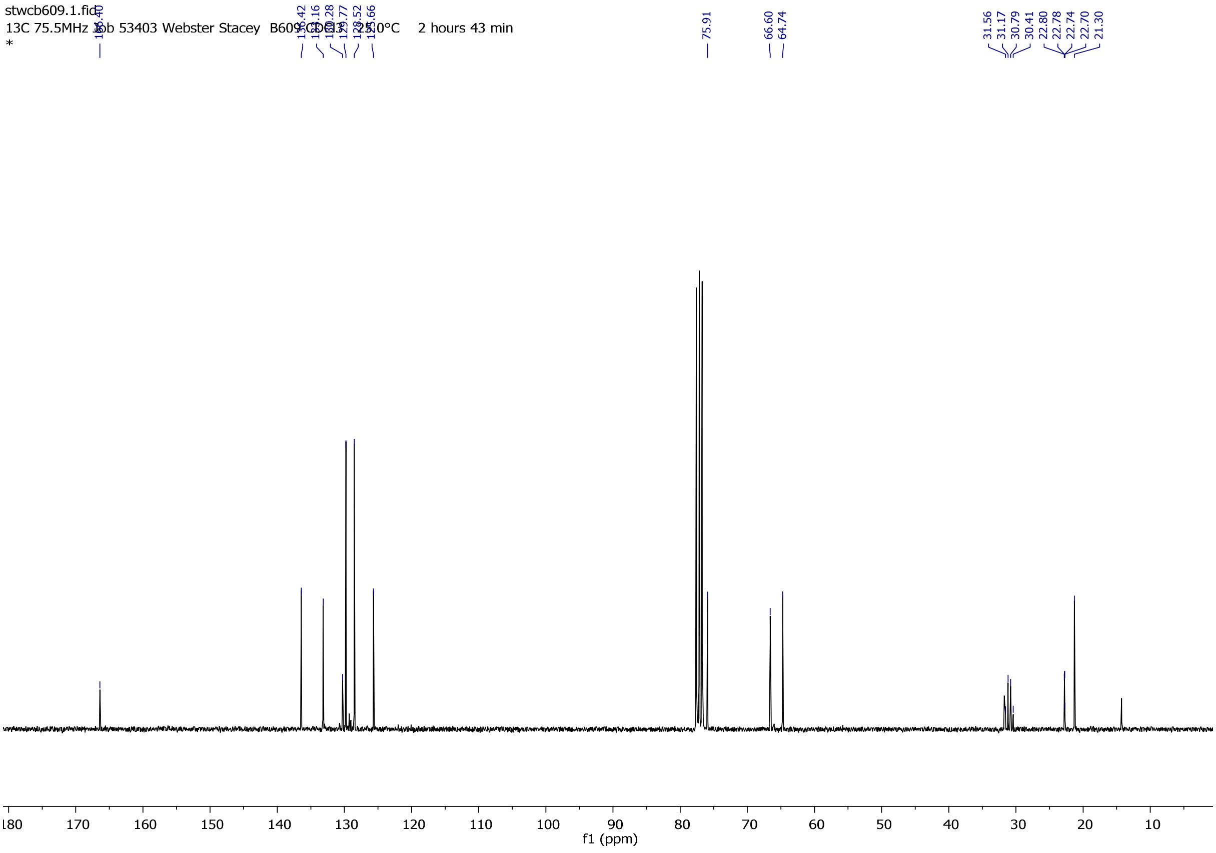
stwcb609.1.fid
13C 75.5MHz Job 53403 Webster Stacey B609 CDCl3 25.0°C 2 hours 43 min
f1 (ppm)

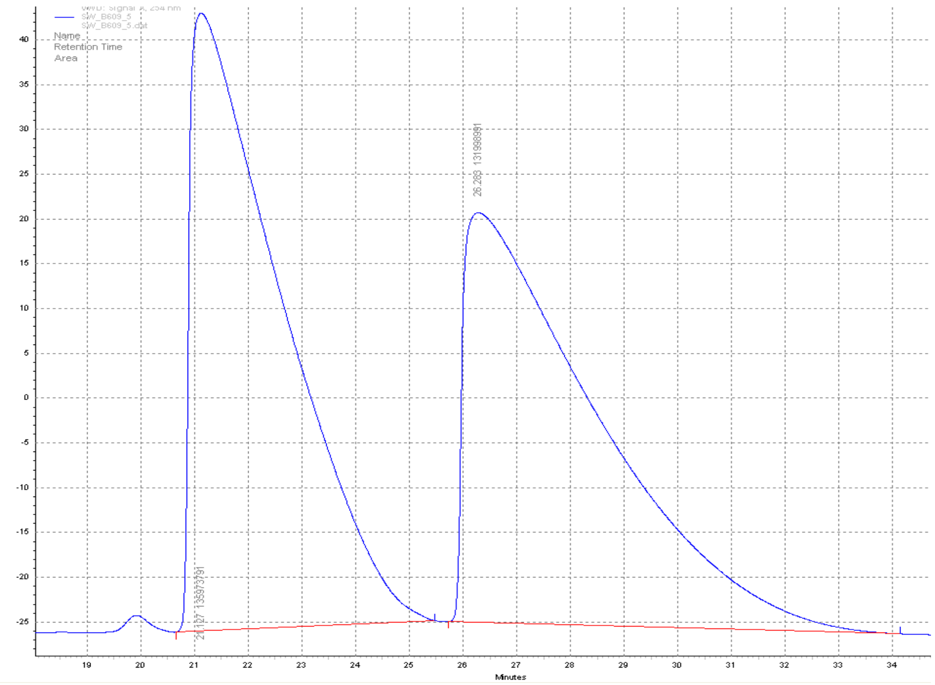

Retention Time	Area	Area %	Height	Height %
21.127	135973791	50.74	1157324	60.14
26.283	131998991	49.26	767113	39.86
Totals	267972782	100.00	1924437	100.00

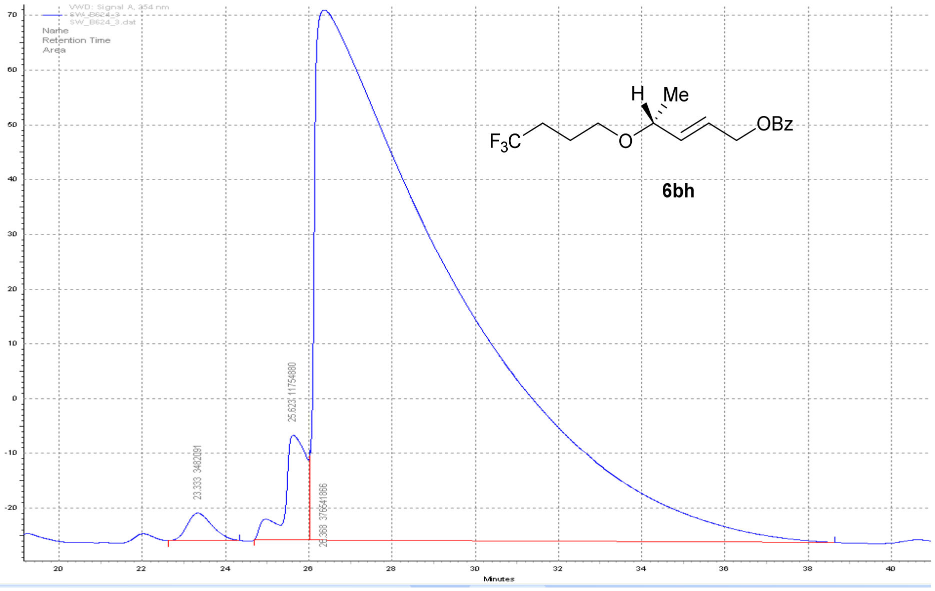

Retention Time	Area	Area %	Height	Height %
23.333	3482091	0.89	84048	4.14
25.623	11754880	3.00	320375	15.80
26.368	376541866	96.11	1623797	80.06
Totals	391778837	100.00	2028220	100.00

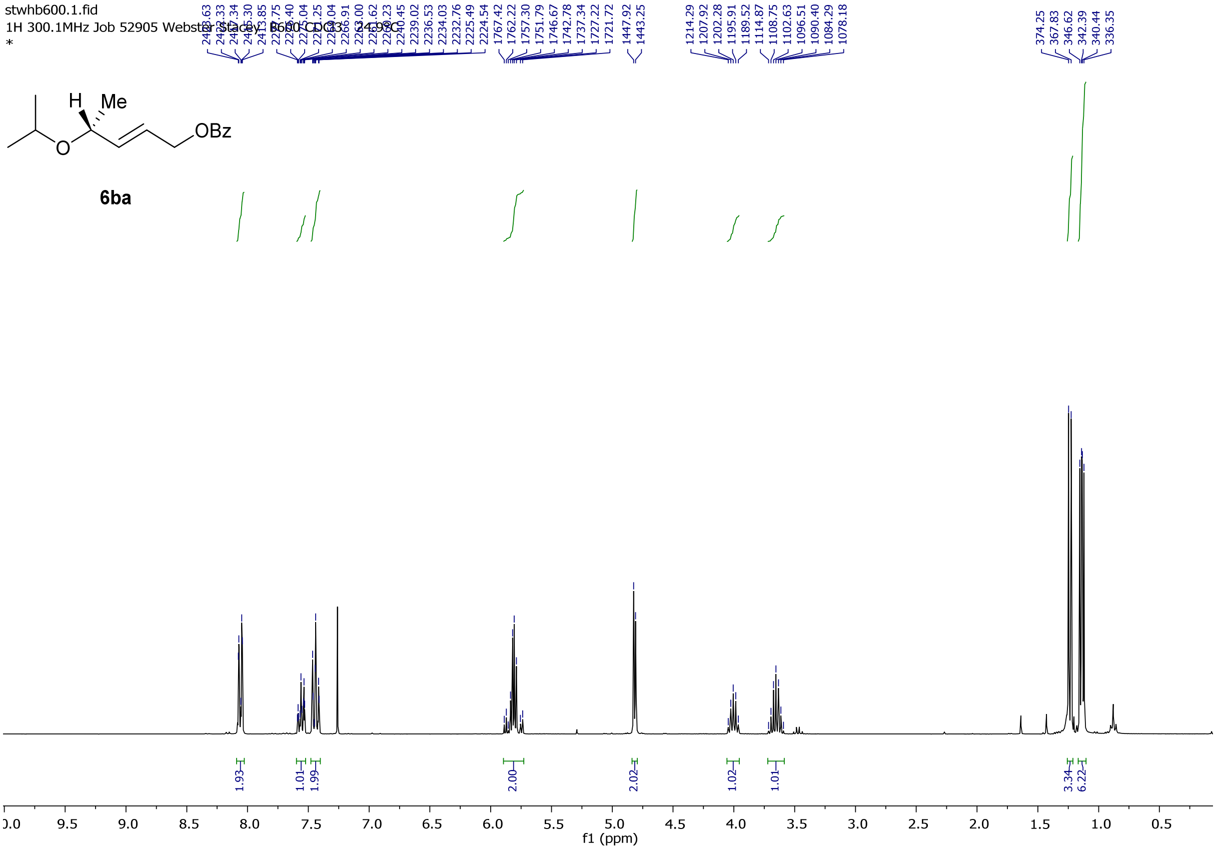
stwhb600.1.fid
1H 300.1MHz Job 52905 Webster Stacey B600 CDCl3 24.9°C
H Me
O
OBz
6ba
1.93
1.01
1.99
2.00
2.02
1.02
1.01
3.34
6.22
f1 (ppm)

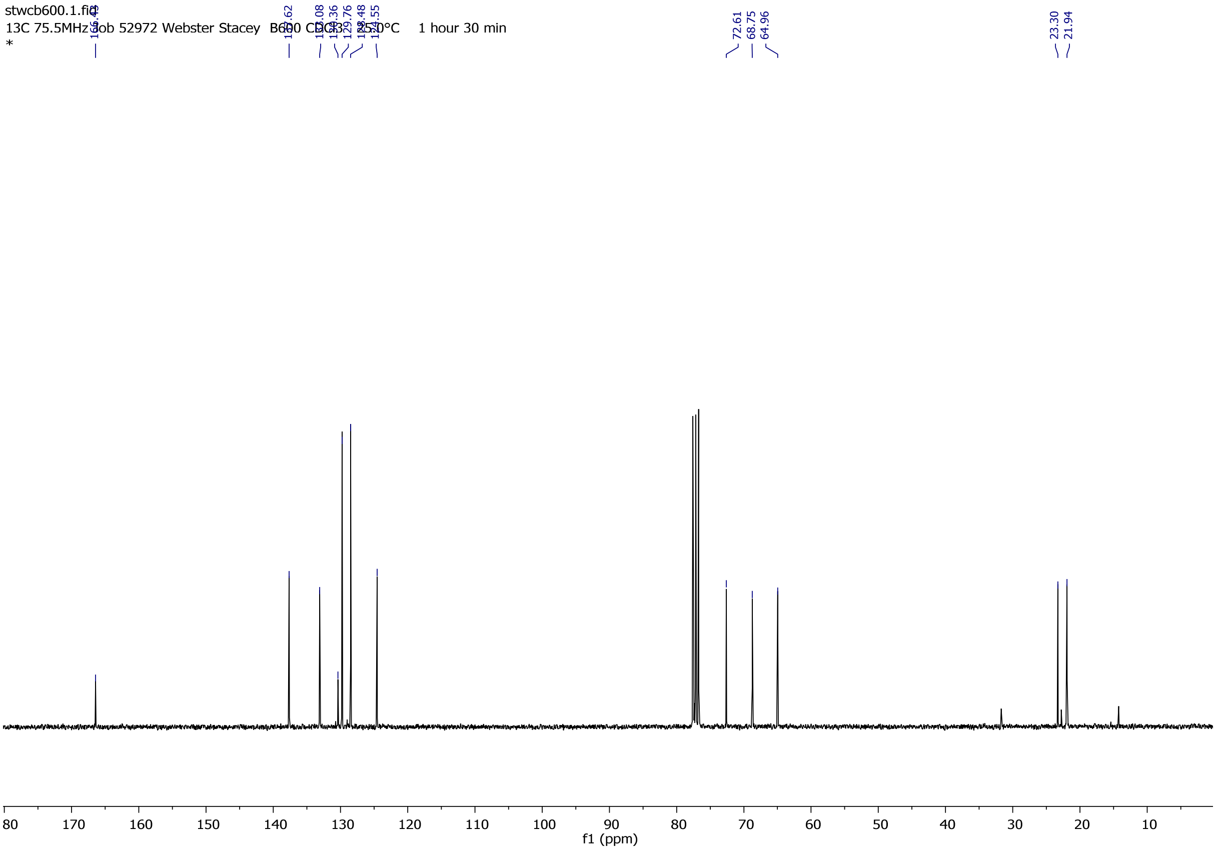
stwcb600.1.fid
13C 75.5MHz Job 52972 Webster Stacey B600 CDCl3 25.0°C 1 hour 30 min
72.61
68.75
64.96
23.30
21.94
f1 (ppm)

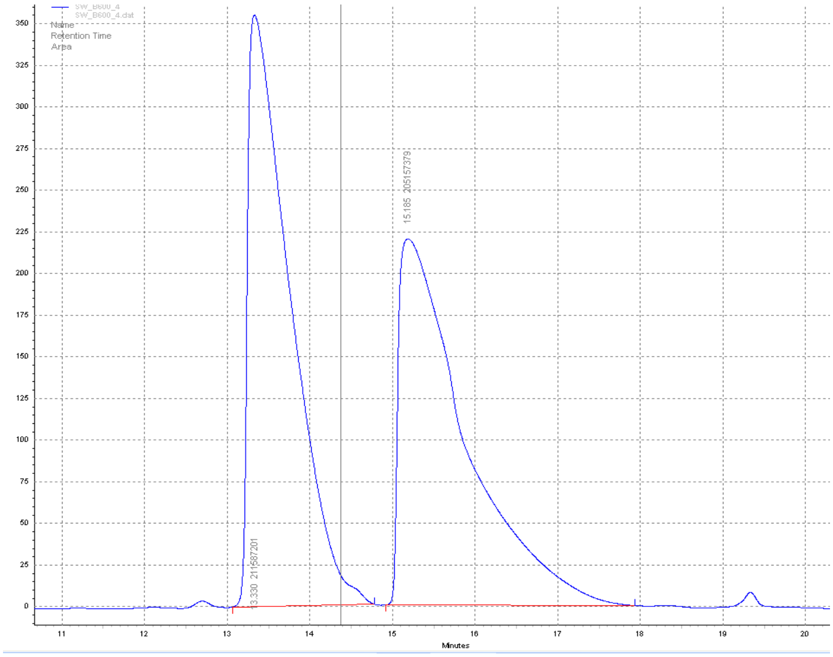

Retention Time	Area	Area %	Height	Height %
13.330	211587201	50.77	5957962	61.77
15.185	205157379	49.23	3687433	38.23
Totals	416744580	100.00	9645395	100.00

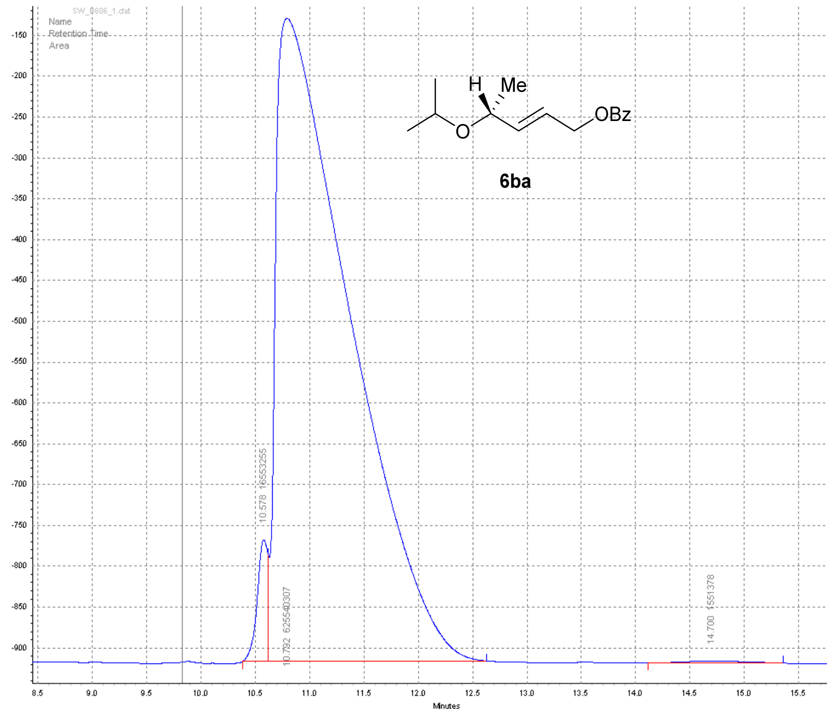

Retention Time	Area	Area %	Height	Height %
10.578	16553255	2.57	2497516	15.86
10.792	625540307	97.19	13208018	83.89
14.700	1551378	0.24	39403	0.25
Totals	643644940	100.00	15744937	100.00

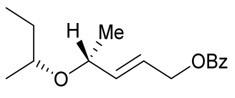

H
Me
O
OBz

6bj

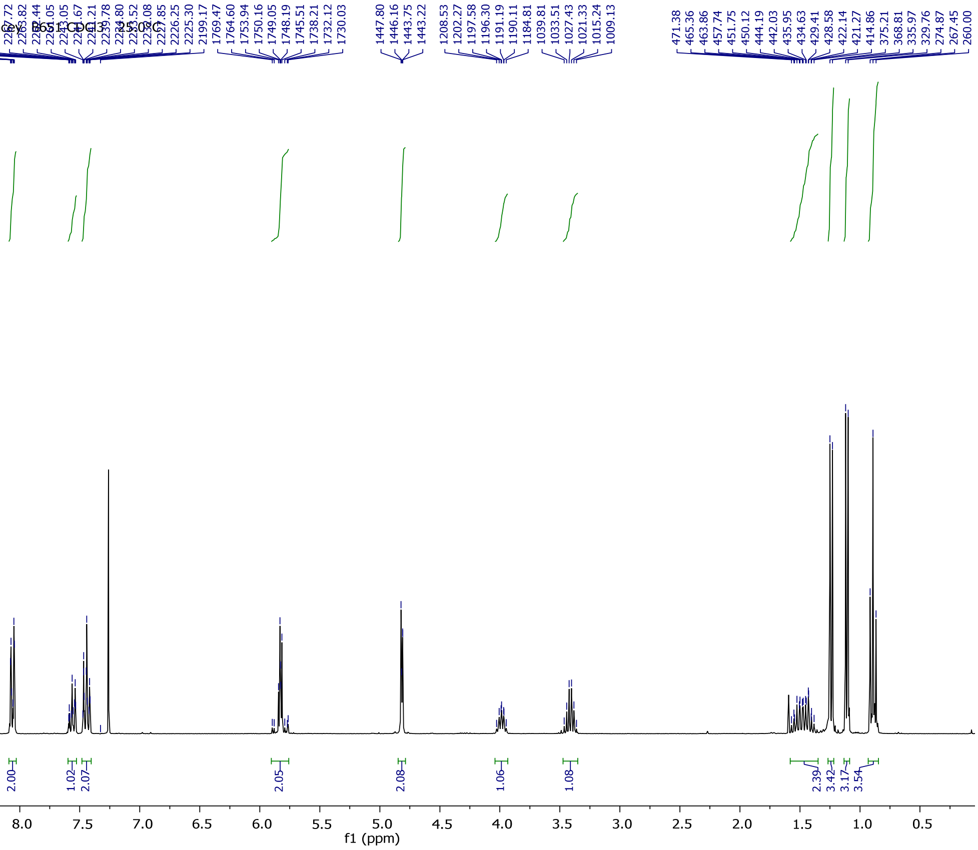

stwhb651.1.fid
1H 300.1MHz
f1 (ppm)
2.00
1.02
2.07
2.05
2.08
1.06
1.08
2.39
3.42
3.17
3.54

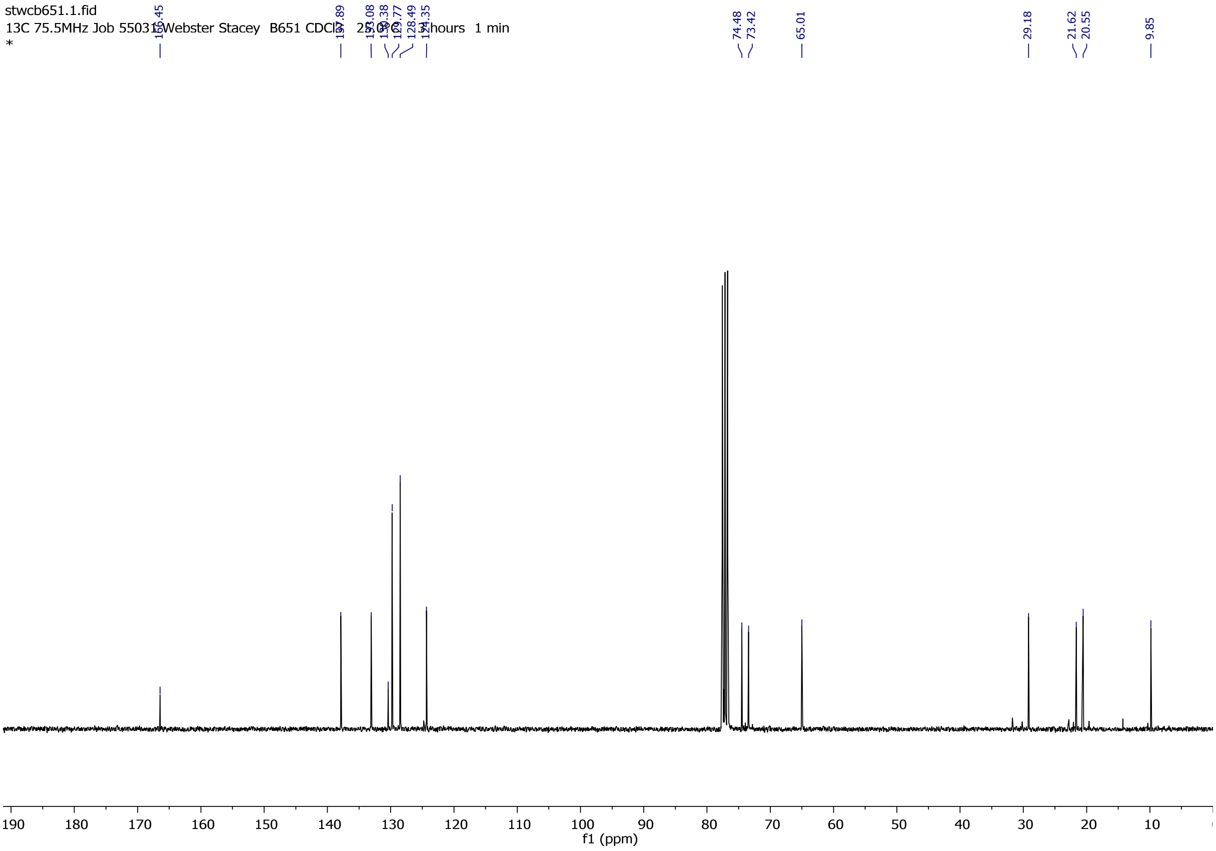

stwcb651.1.fid
13C 75.5MHz Job 55031 Webster Stacey B651 CDCl3
hours 1 min
137.89
133.08
130.38
129.77
128.49
74.48
73.42
65.01
29.18
21.62
20.55
9.85
f1 (ppm)

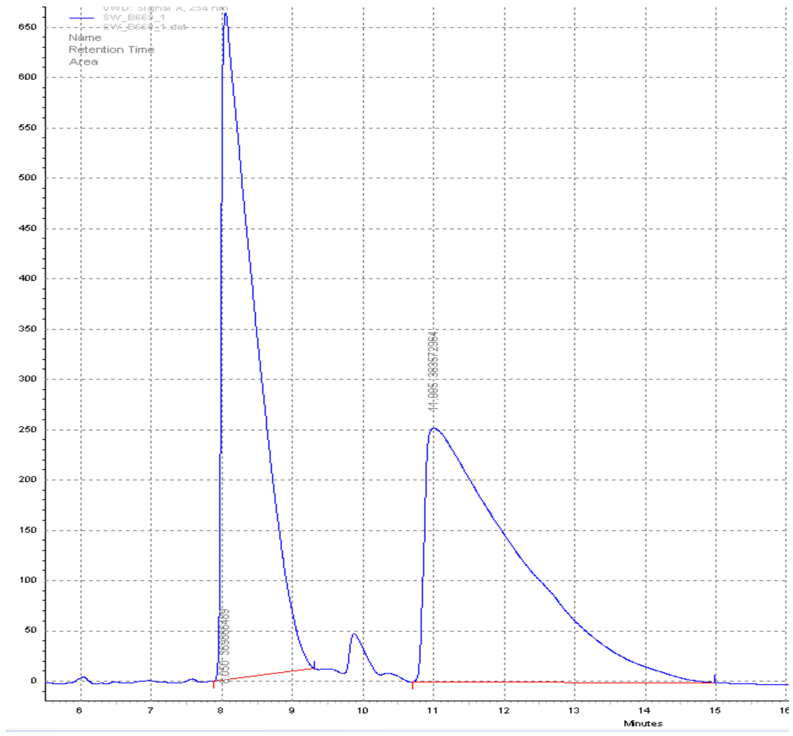

Retention Time	Area	Area %	Height	Height %
8.050	369866489	49.09	11120556	72.40
11.005	383572964	50.91	4239275	27.60

Totals	753439453	100.00	15359831	100.00

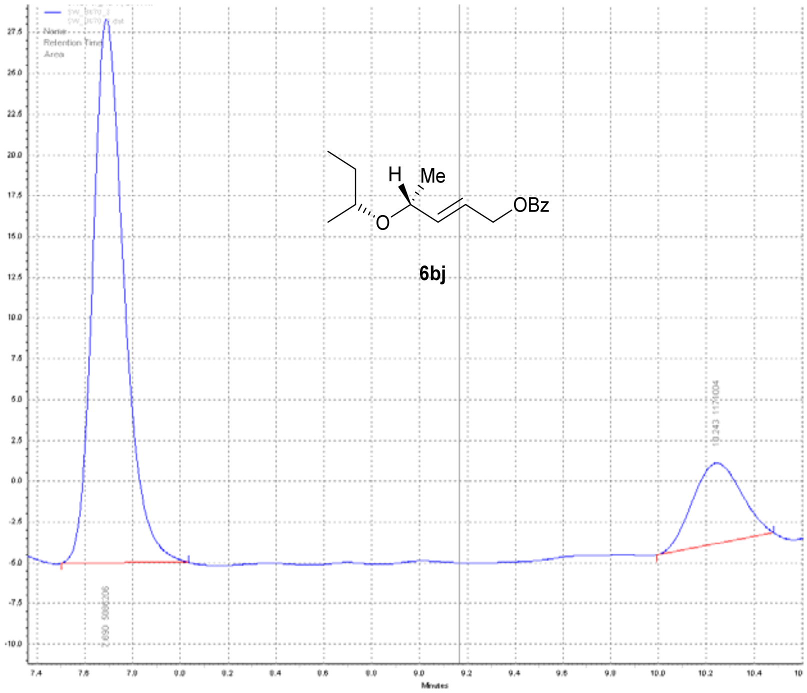

Retention Time	Area	Area %	Height	Height %
7.690	5086206	81.29	559286	87.08
10.243	1171004	18.71	82963	12.92

Totals	6257210	100.00	642249	100.00

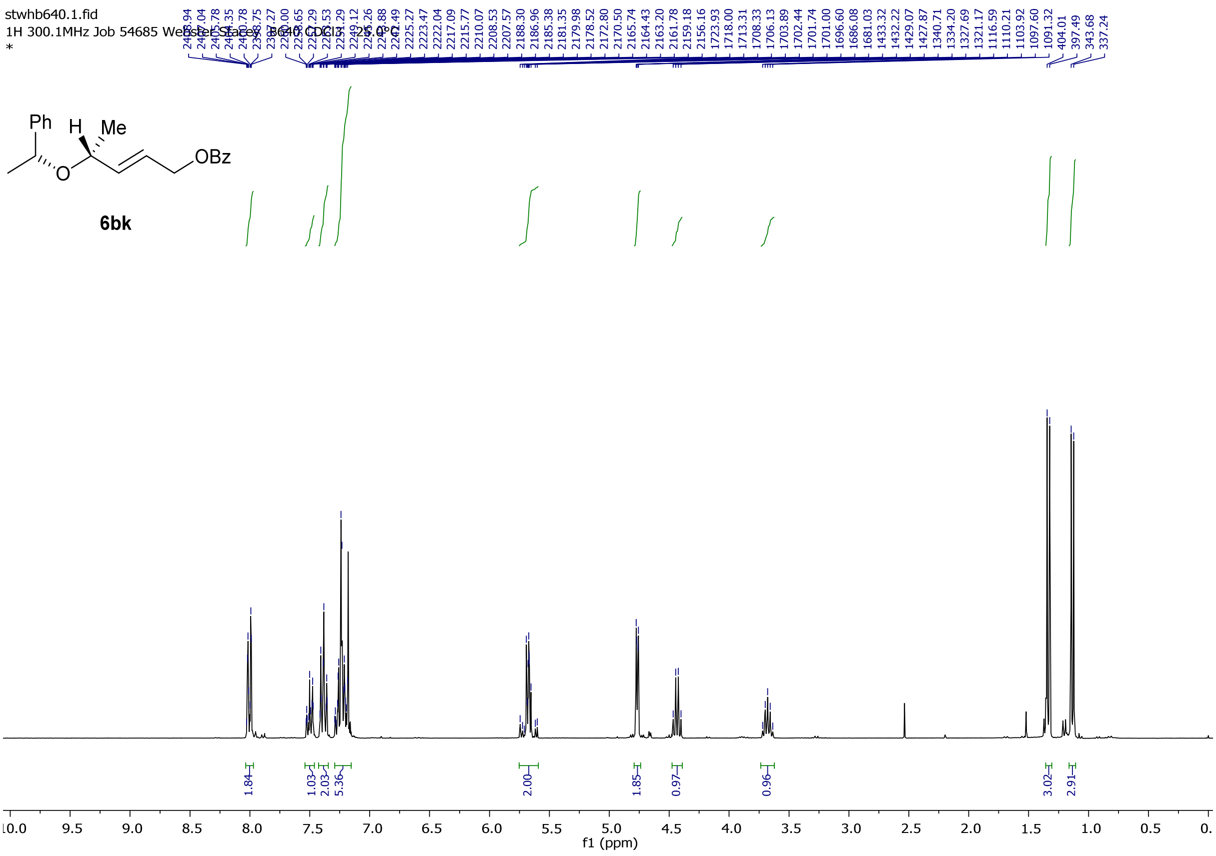
stwhb640.1.fid
Ph
H
Me
OBz
O
6bk
f1 (ppm)

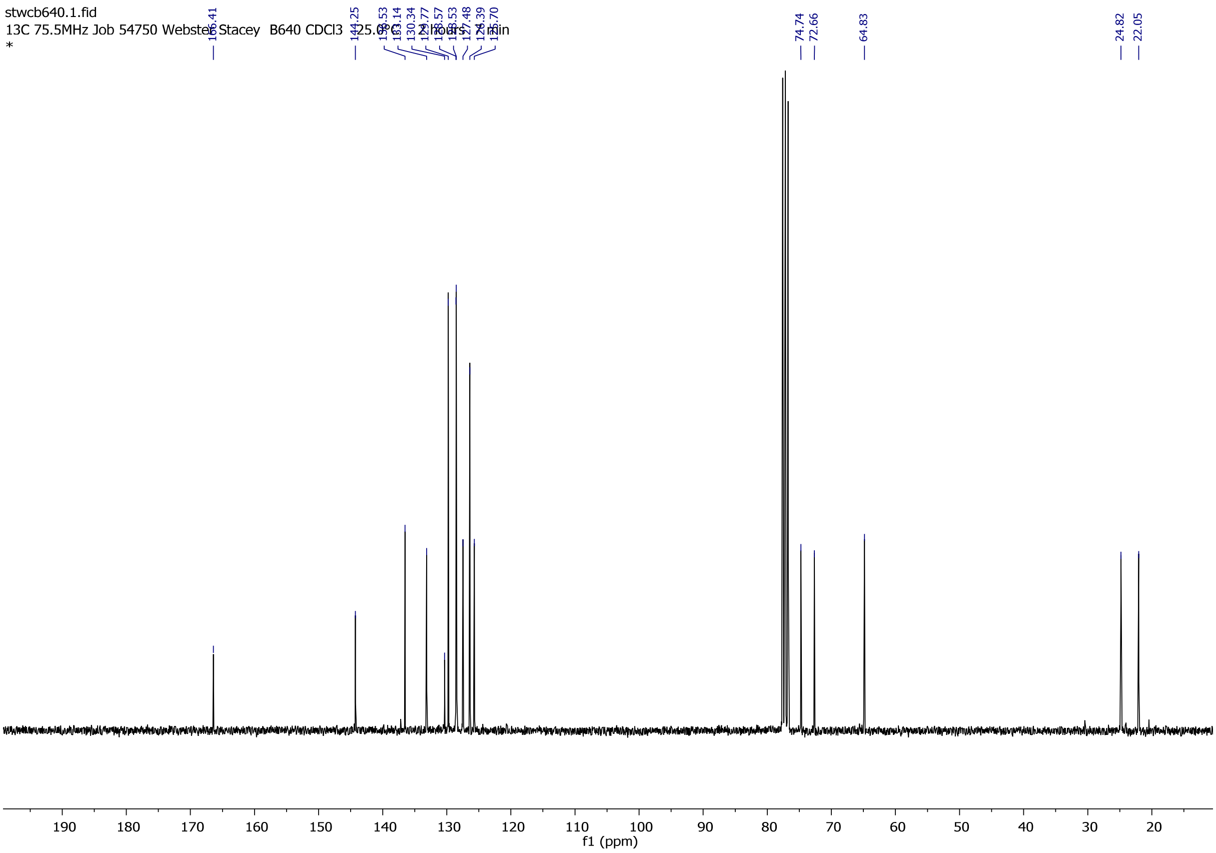
stwcb640.1.fid
13C 75.5MHz Job 54750 Webster Stacey B640 CDCl3
144.25
74.74
72.66
64.83
24.82
22.05
f1 (ppm)

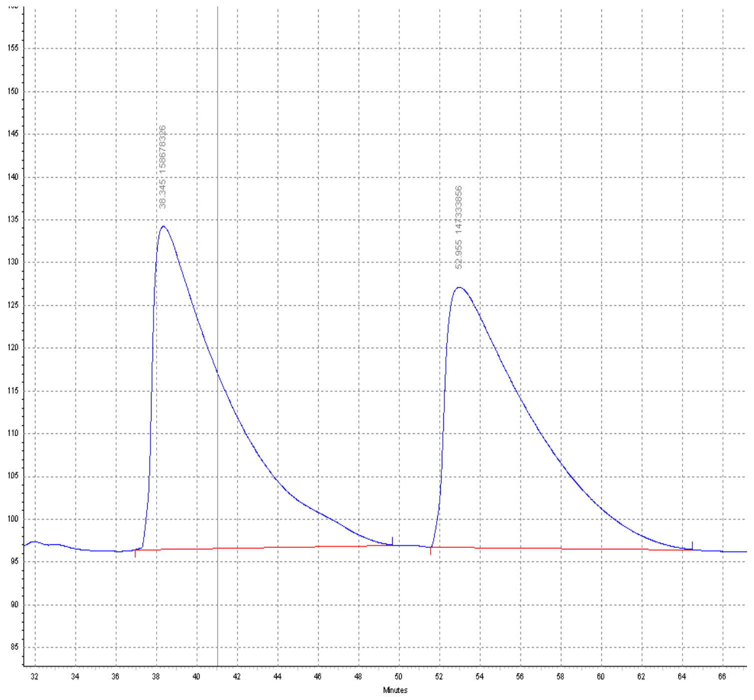

Retention Time	Area	Area %	Height	Height %
38.345	158678326	51.85	633417	55.36
52.955	147333856	48.15	510691	44.64

Totals				
	306012182	100.00	1144108	100.00

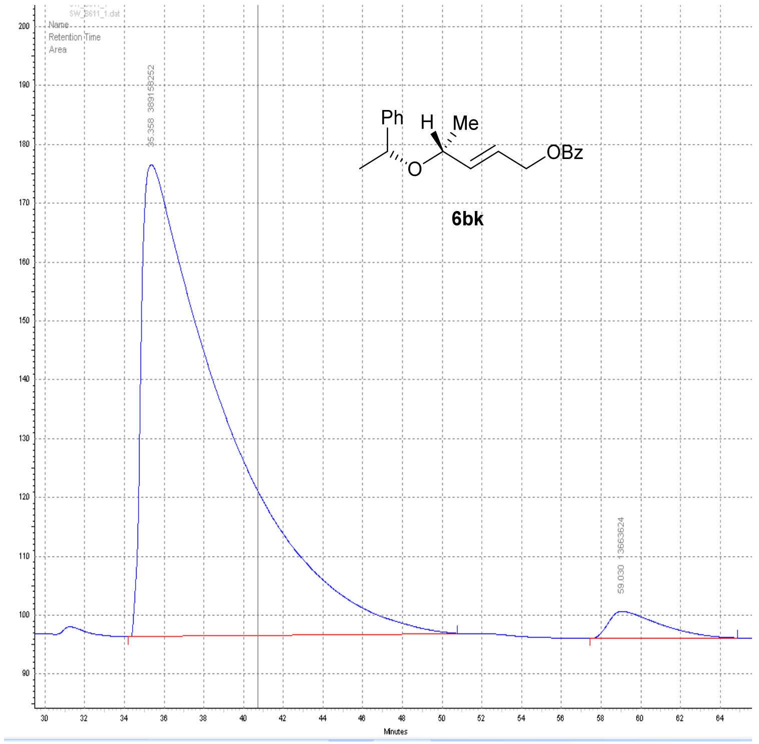

Retention Time	Area	Area %	Height	Height %
35.358	389158252	96.61	1344611	94.61
59.030	13663624	3.39	76537	5.39

Totals				
	402821876	100.00	1421148	100.00

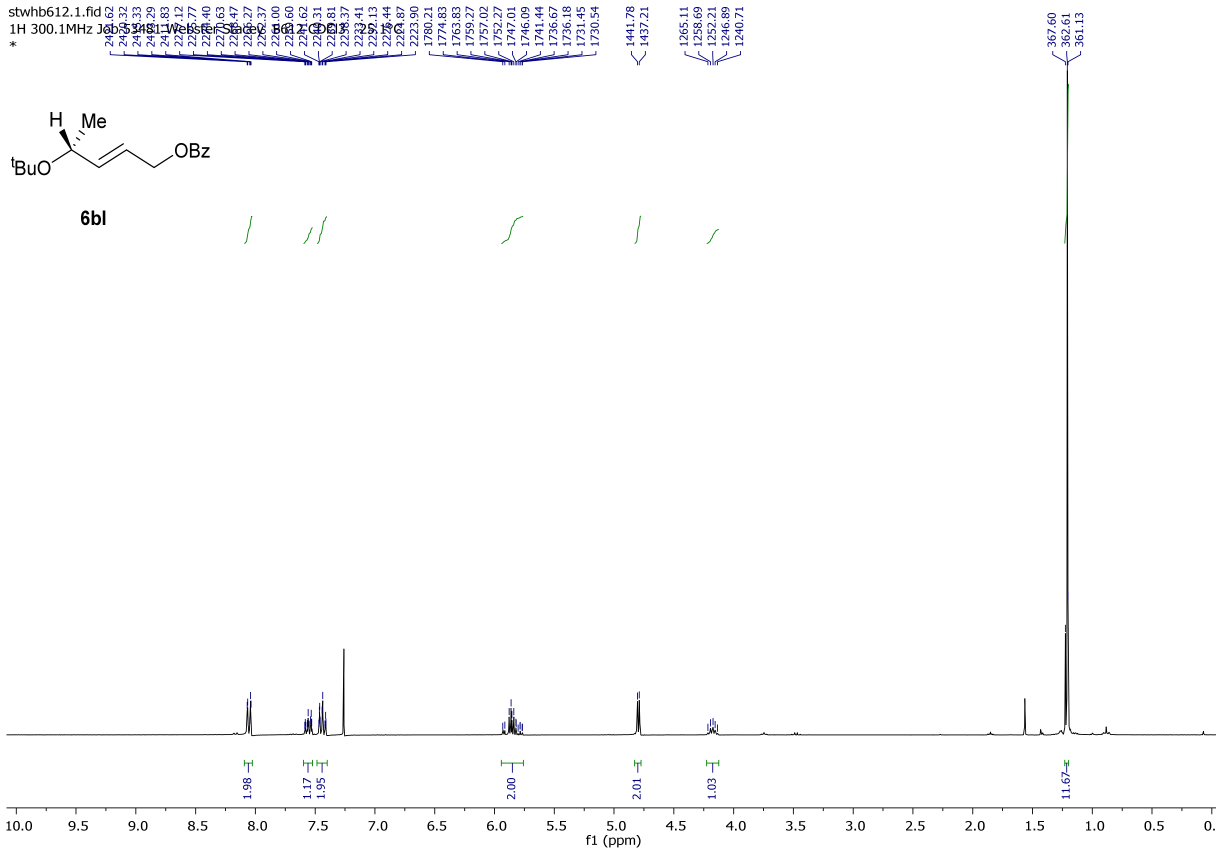
stwhb612.1.fid
1H 300.1MHz Job 53481 Webster Stacey B612 CDCl3
6bl
f1 (ppm)

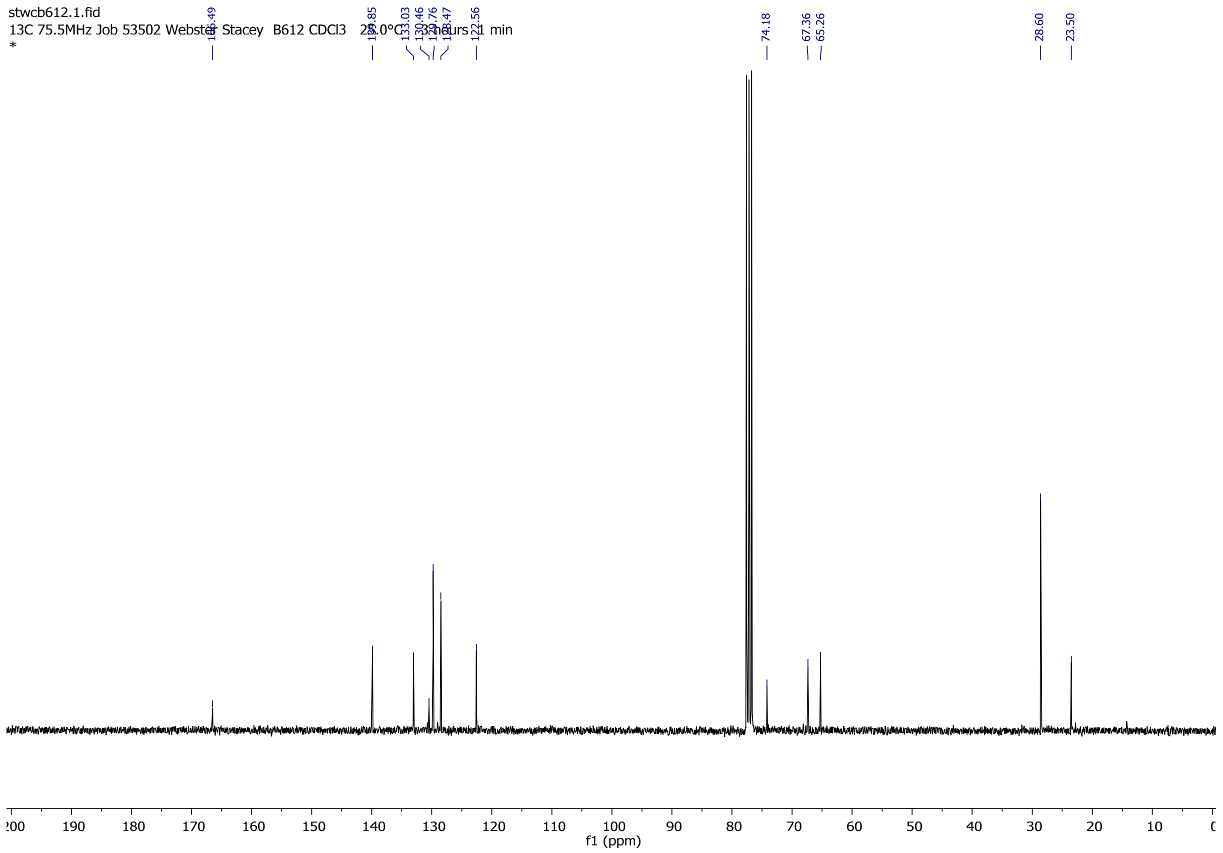
stwcb612.1.fid
13C 75.5MHz Job 53502 Webster Stacey B612 CDCl3
74.18
67.36
65.26
28.60
23.50
f1 (ppm)

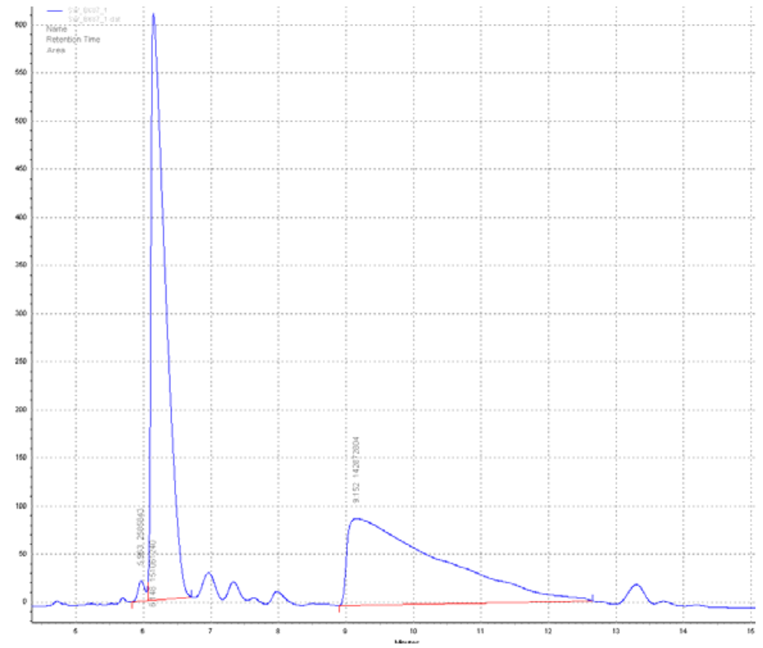

Retention Time	Area	Area %	Height	Height %
5.963	2585843	0.87	353919	2.93
6.148	151061240	50.94	10226632	84.56
9.152	142872804	48.18	1513466	12.51
Totals	296519887	100.00	12094017	100.00

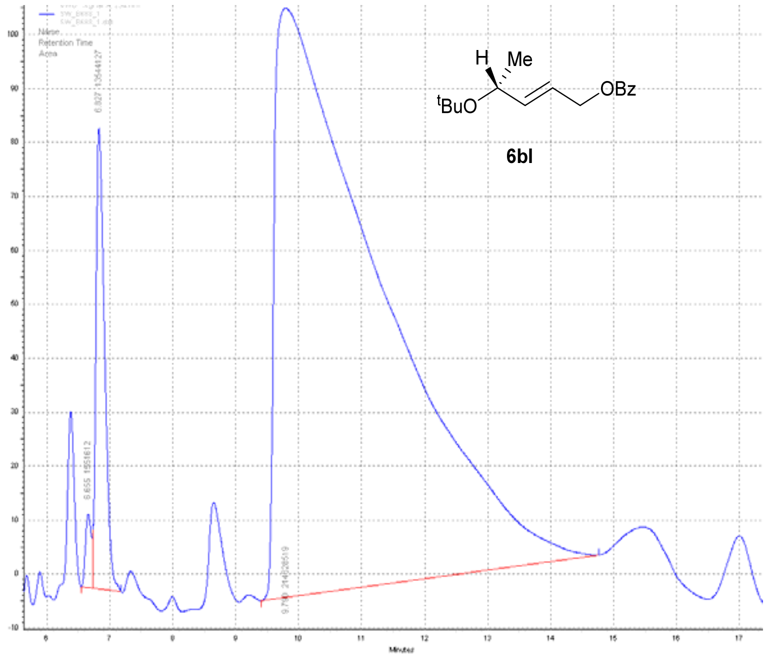

Retention Time	Area	Area %	Height	Height %
6.655	1551612	0.68	228507	6.54
6.827	13544127	5.90	1433521	41.01
9.790	214628519	93.43	1833250	52.45
Totals	229724258	100.00	3495278	100.00

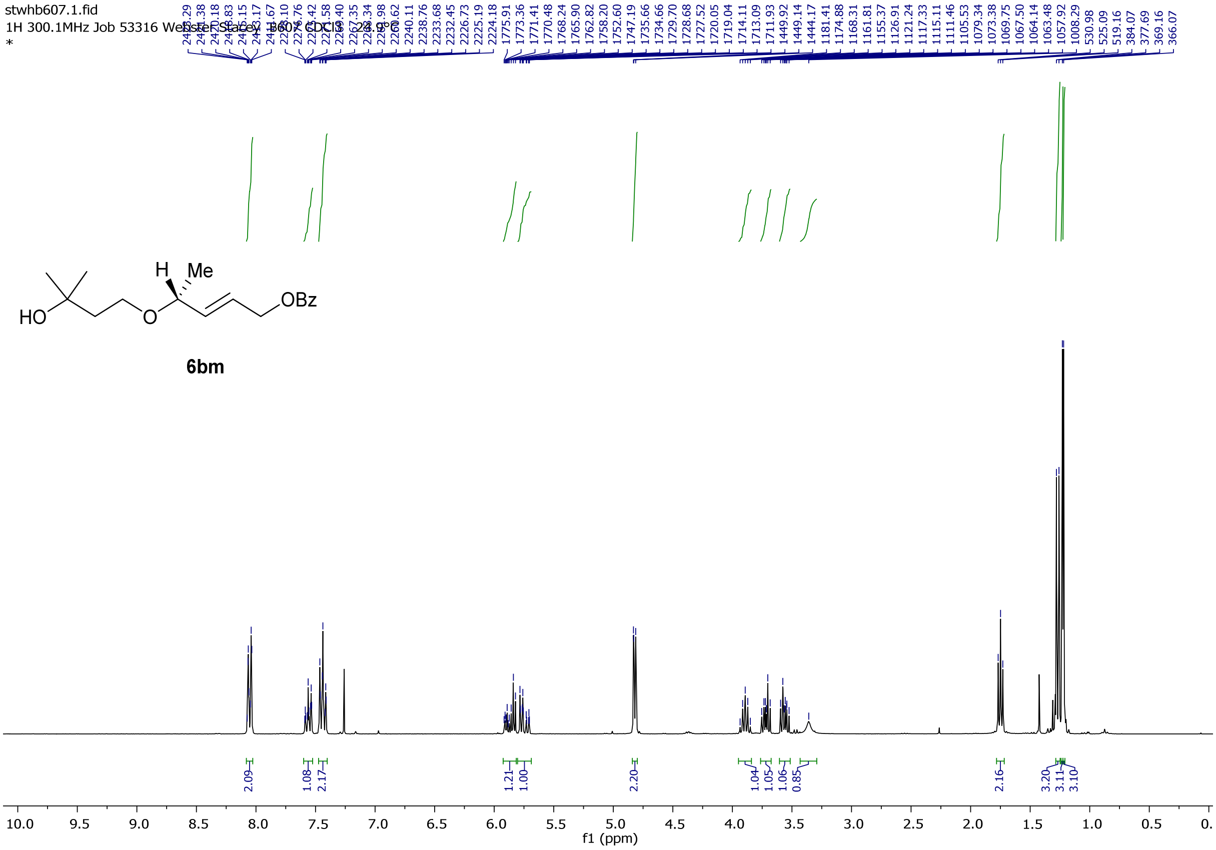
stwhb607.1.fid
1H 300.1MHz Job 53316 Webster Stacey B607 CDCl3
f1 (ppm)
H
Me
HO
O
OBz

6bm

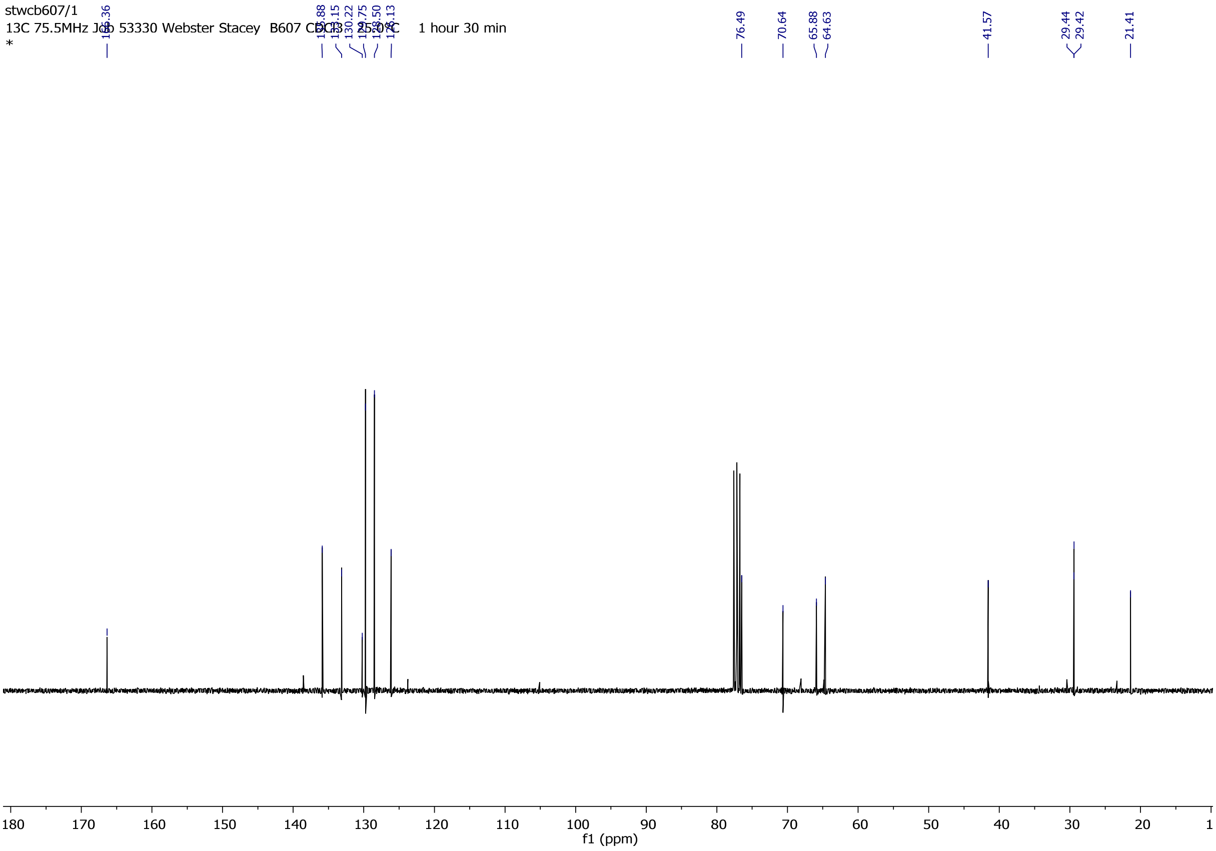
stwcb607/1
13C 75.5MHz Job 53330 Webster Stacey B607 CDCl3 25.0°C 1 hour 30 min
166.36
135.88
133.15
130.22
129.75
128.50
126.13
76.49
70.64
65.88
64.63
41.57
29.44
29.42
21.41
f1 (ppm)

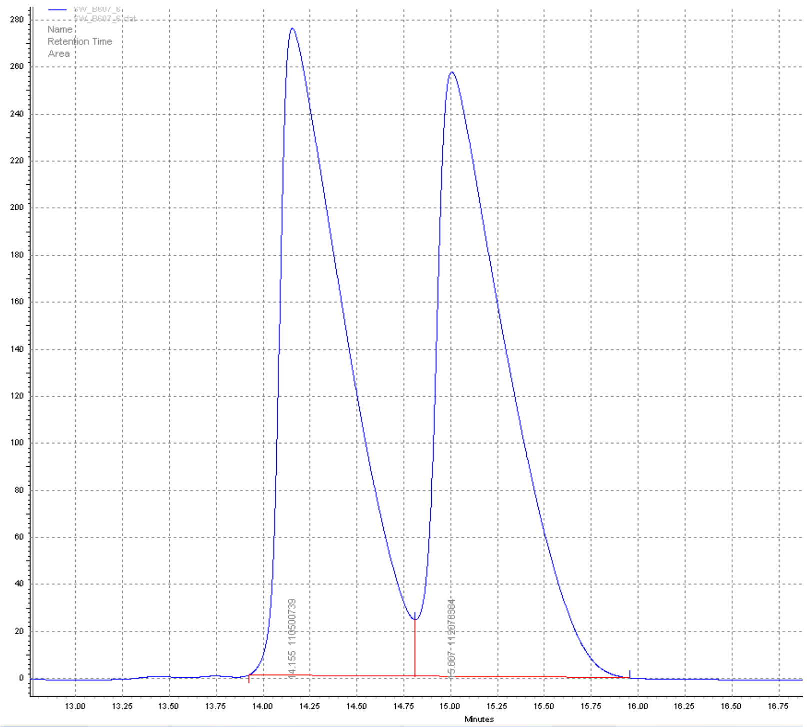

Retention Time	Area	Area %	Height	Height %
14.155	110500739	49.65	4616822	51.70
15.007	112078384	50.35	4312926	48.30
Totals	222579123	100.00	8929748	100.00

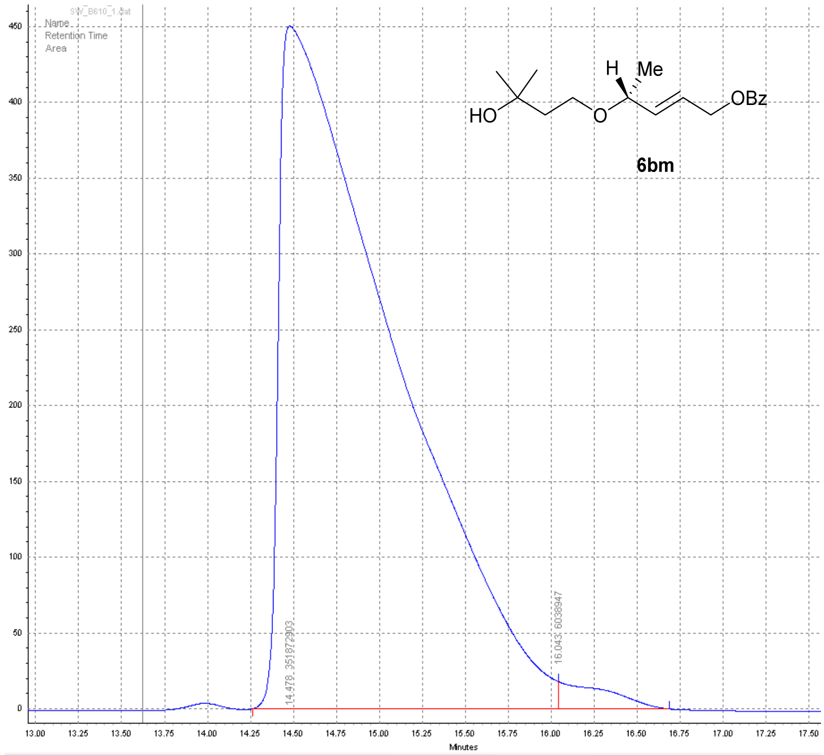

Retention Time	Area	Area %	Height	Height %
14.478	351872903	98.31	7554428	96.17
16.043	6038947	1.69	300737	3.83
Totals	357911850	100.00	7855165	100.00

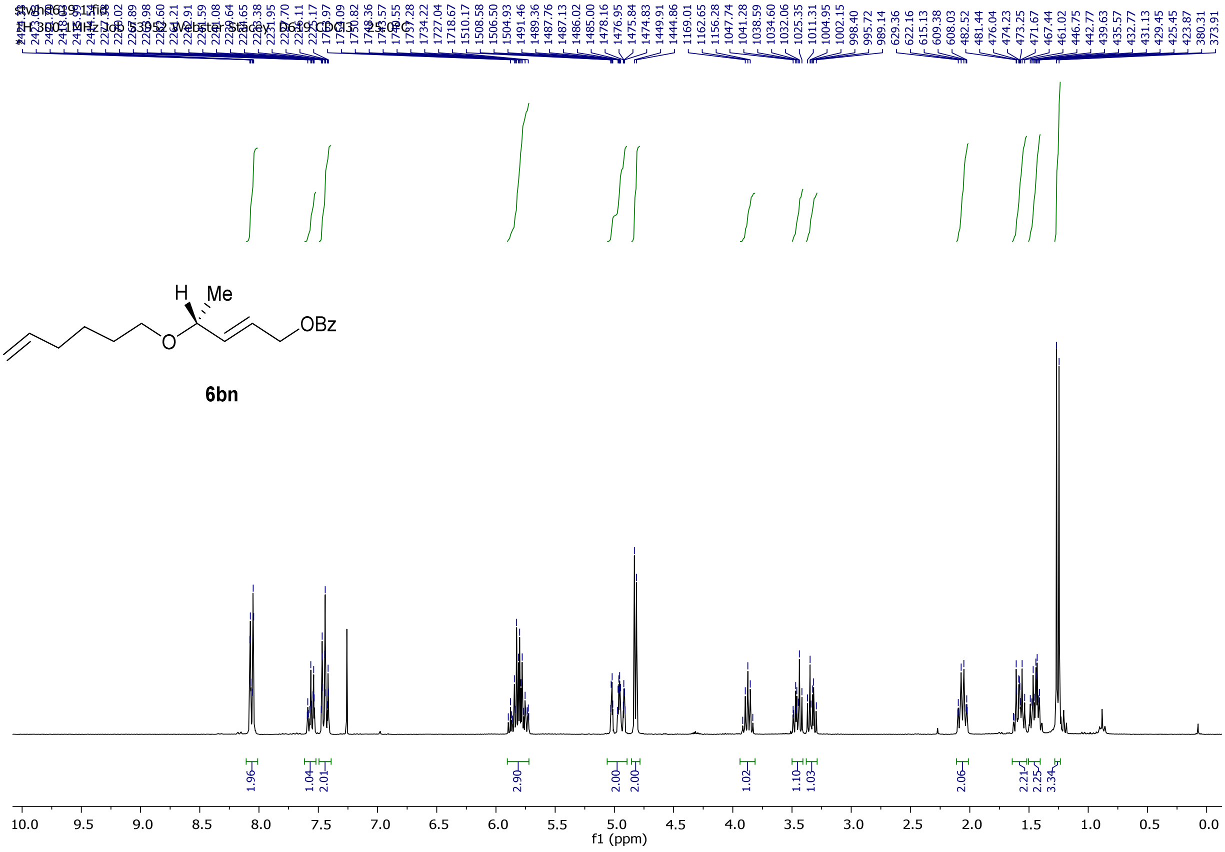

6bn

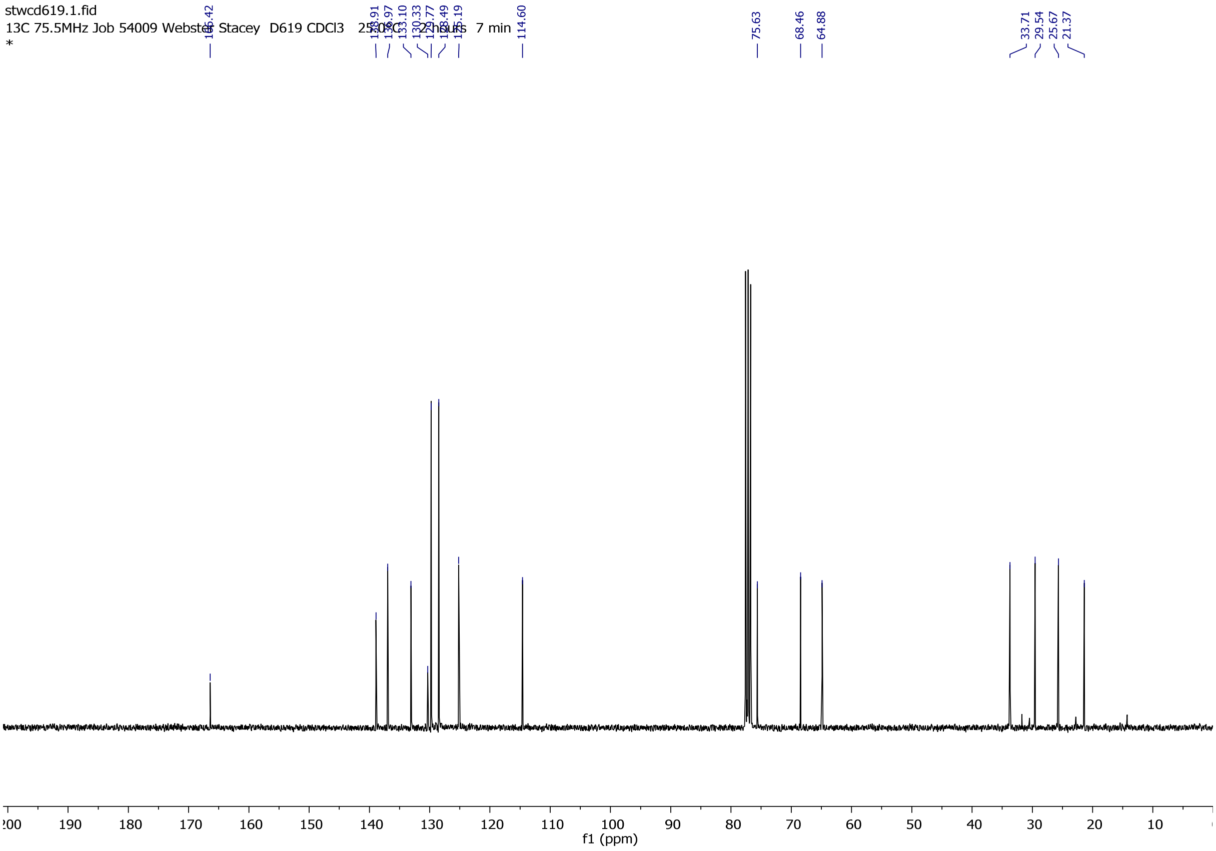

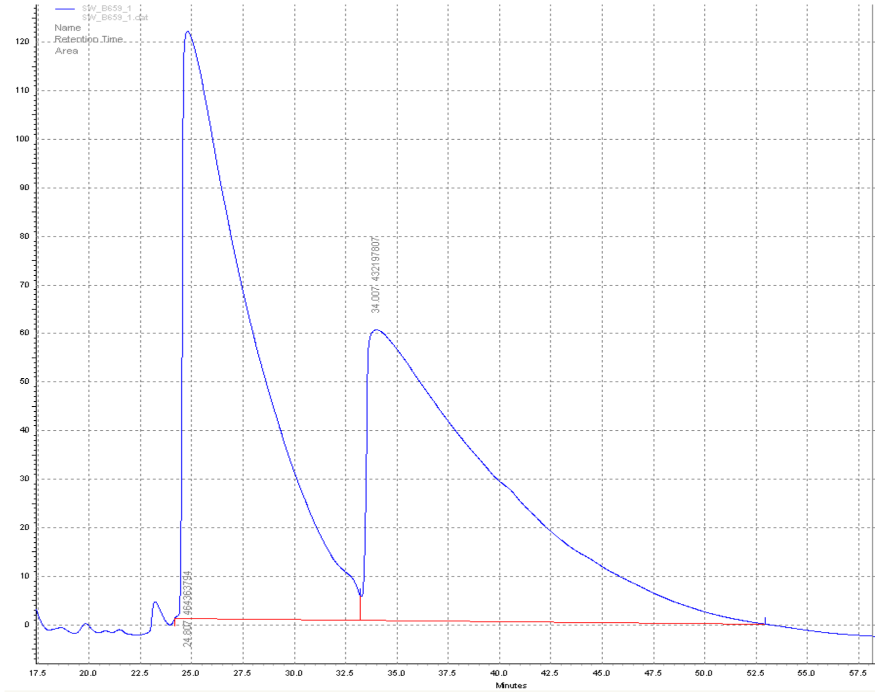

Retention Time	Area	Area %	Height	Height %
24.807	464363794	51.79	2029287	66.90
34.007	432197807	48.21	1004243	33.10
Totals	896561601	100.00	3033530	100.00

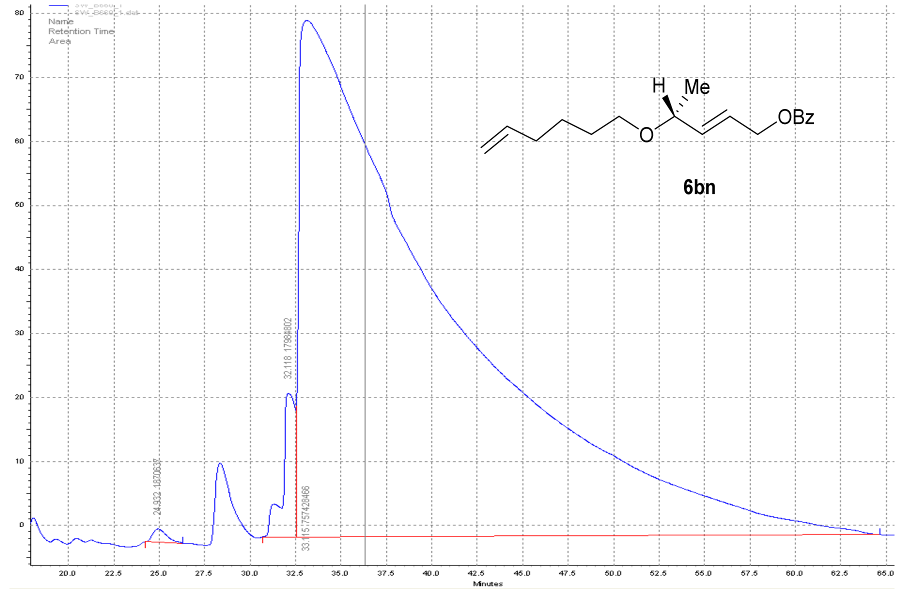

6bn

Retention Time	Area	Area %	Height	Height %
24.932	1870637	0.24	34722	1.97
32.118	17984802	2.31	376115	21.32
33.115	757428466	97.45	1353465	76.71
Totals	777283905	100.00	1764302	100.00

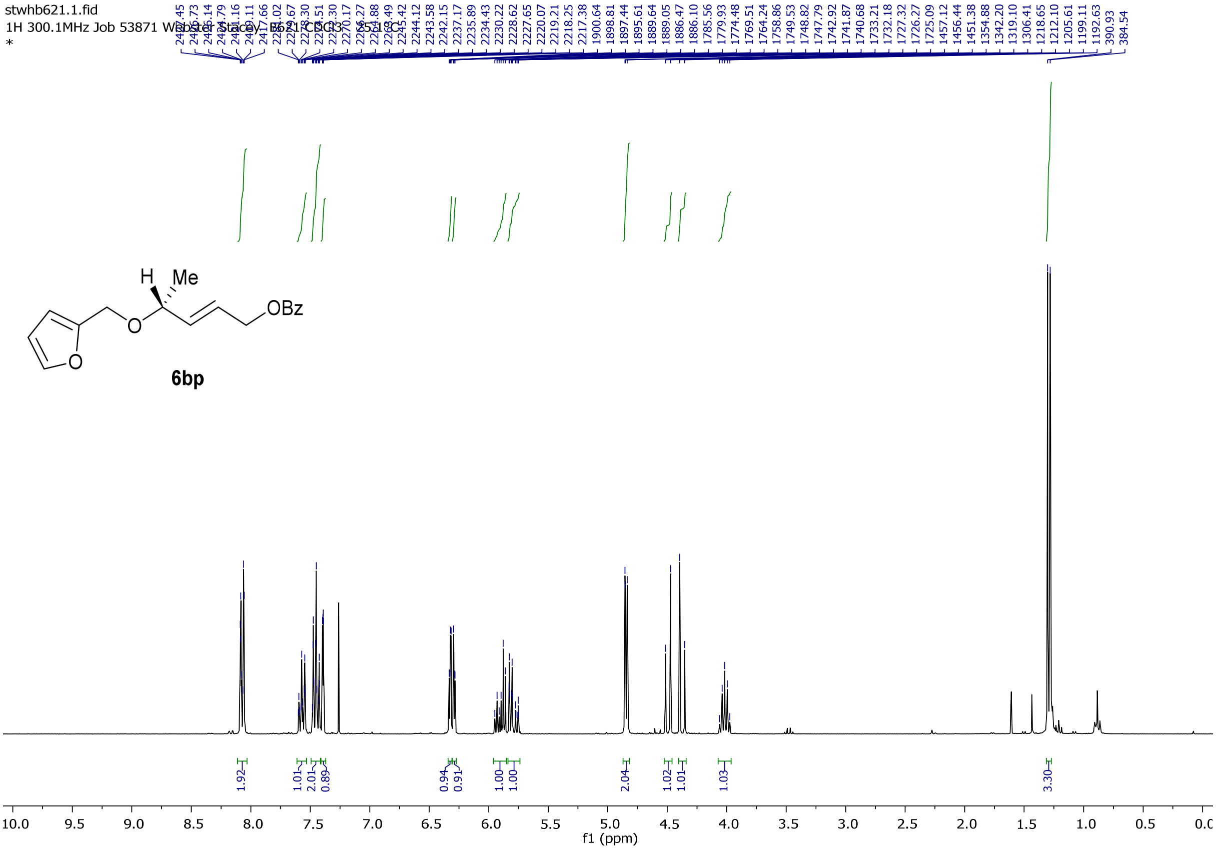
stwhb621.1.fid
1H 300.1MHz Job 53871 Webster Stacey B621 CDCl3 25.0°C
H
Me
OBz
O
O
6bp
f1 (ppm)

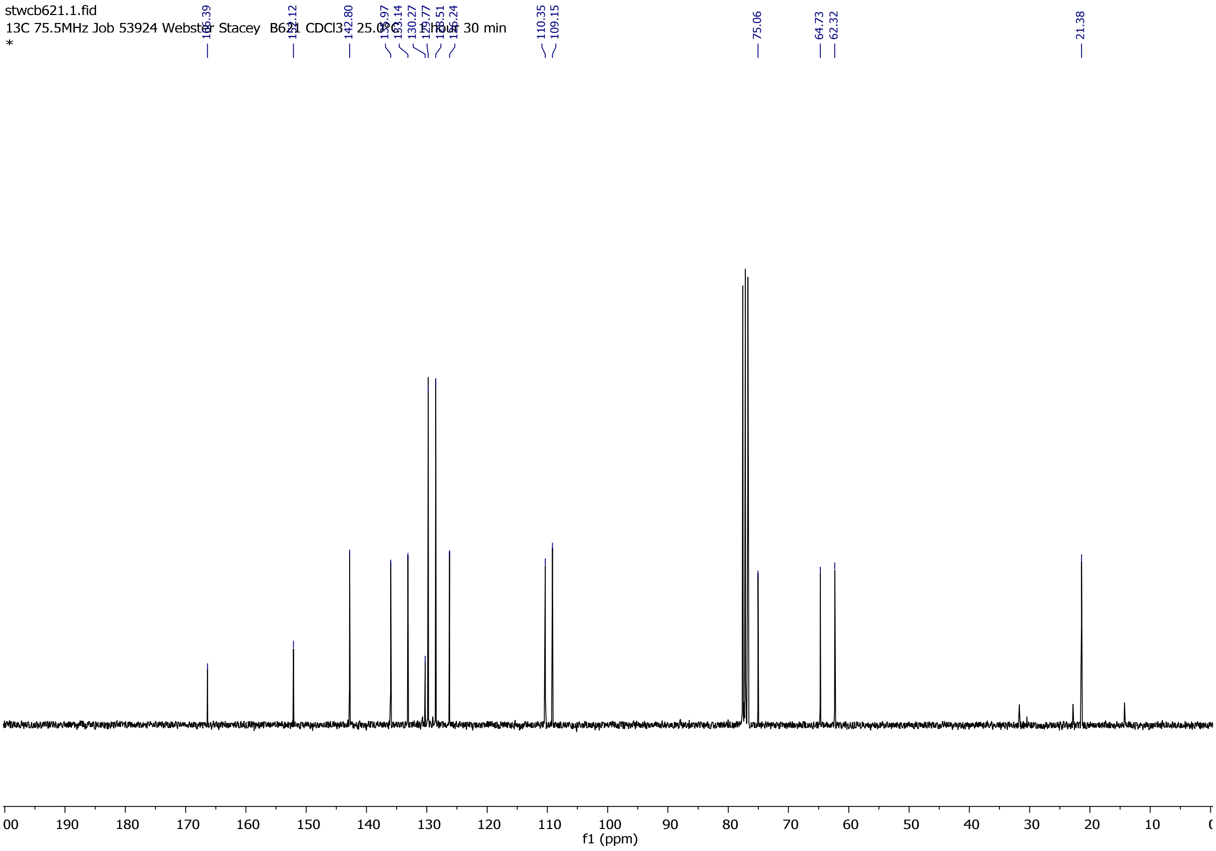
stwcb621.1.fid
13C 75.5MHz Job 53924 Webster Stacey B621 CDCl3 25.0°C 1 hour 30 min
142.80
130.27
110.35
109.15
75.06
64.73
62.32
21.38
f1 (ppm)

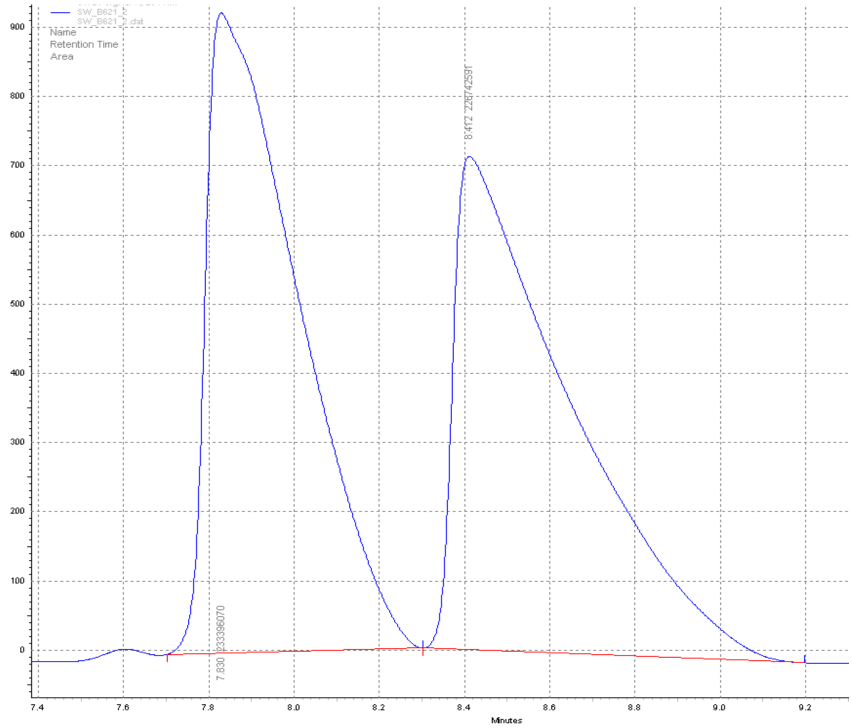

Retention Time	Area	Area %	Height	Height %
7.830	233396070	50.72	15515743	56.47
8.412	226742591	49.28	11961005	43.53
Totals	460138661	100.00	27476748	100.00

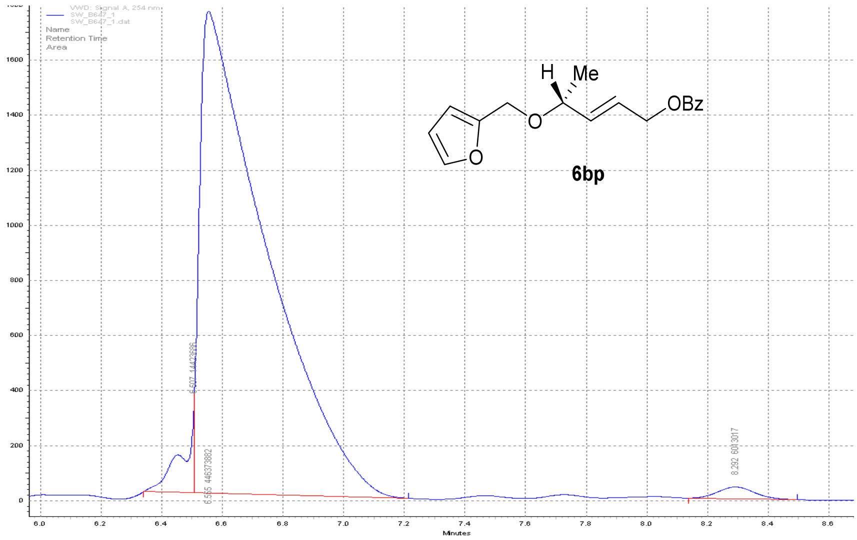

Retention Time	Area	Area %	Height	Height %
6.507	14423686	3.09	5302290	14.98
6.555	446373882	95.62	29370180	83.00
8.292	6013017	1.29	715139	2.02
Totals	466810585	100.00	35387609	100.00

References

[1] G. Barker, D. G. Johnson, P. C. Young, S. A. Macgregor, A.-L. Lee, *Chem. Eur. J.* **2015**, *21*, 13748-13757.

[2] Z. Zhang, R. A. Widenhoefer, *Org. Lett.* **2008**, *10*, 2079-2081.

[3] T. R. Hoye, C. S. Jeffrey, F. Shao, *Nat. Protoc.* **2007**, *2*, 2451-2458.

[4] Y. Yatsumonji, Y. Ishida, A. Tsubouchi, T. Takeda, *Org. Lett.* **2007**, *9*, 4603-4606.

[5] T. R. Hoye, S. E. Erickson, S. L. Erickson-Birkedahl, C. R. H. Hale, E. C. Izgu, M. J. Mayer, P. K. Notz, M. K. Renner, *Org. Lett.* **2010**, *12*, 1768-1771.

[6] X. Huang, T. Cao, L. C. Han, X. G. Jiang, W. L. Lin, J. S. Zhang, S. Ma, *Chem. Commun.* **2015**, *51*, 6956.

[7] A. H. Stoll, S. B. Blakey, *J. Am. Chem. Soc.* **2010**, *132*, 2108-2109.

[8] M. R. Crittall, N. W. G. Fairhurst, D. R. Carbery, *Chem. Commun.* **2012**, *48*, 11181-11183.

[9] R. Gurubrahamam, M. Periasamy, *J. Org. Chem.* **2013**, *78*, 1463-1470.

[10] Z. Zhang, R. A. Widenhoefer, *Org. Lett.* **2008**, *10*, 2079-2081.

[11] L. Lempke, T. Fischer, J. Bell, W. Kraus, K. Rurack, N. Krause, *Org. Biomol. Chem.* **2015**, *13*, 3787-3791.

[12] T. J. Donohoe, J. A. Basutto, J. F. Bower, A. Rathi, *Org. Lett.* **2011**, *13*, 1036-1039.

[13] R. A. Smith, R. L. White, A. Krantz, *J. Med. Chem.* **1988**, *31*, 1558-1566.

[14] B. M. Trost, D. R. Fandrick, D. C. Dinh, *J Am Chem Soc* **2005**, *127*, 14186-14187.

[15] A. G. Myers, B. Zheng, *J. Am. Chem. Soc.* **1996**, *118*, 4492-4493.

[16] X. Pu, J. M. Ready, *J. Am. Chem. Soc.* **2008**, *130*, 10874-10875.

[17] G.-J. Jiang, Q.-H. Zheng, M. Dou, L.-G. Zhuo, W. Meng, Z.-X. Yu, *J. Org. Chem.* **2013**, *78*, 11783-11793.
